# Blood lipids mediate the effects of gut microbiome on endometriosis: a mendelian randomization study

**DOI:** 10.1186/s12944-024-02096-y

**Published:** 2024-04-16

**Authors:** Chang Su, Su Wan, Jin Ding, Guantai Ni, Huafeng Ding

**Affiliations:** 1 Department of Obstetrics and Gynecology, The First Affiliated Hospital of Wannan Medical College, Wuhu, China; 2 Anhui Province Key Laboratory of Non-coding RNA Basic and Clinical Transformation, Wuhu, China

**Keywords:** Mendelian randomization, Gut microbiome, Blood lipid, Endometriosis, Mediation

## Abstract

**Background:**

There is evidence for an association between the gut microbiome and endometriosis. However, their causal relationship and the mediating role of lipid metabolism remain unclear.

**Methods:**

Using genome-wide association study (GWAS) data, we conducted a bidirectional Mendelian randomization (MR) analysis to investigate the causal relationships between gut microbiome and endometriosis. The inverse variance weighted (IVW) method was used as the primary model, with other MR models used for comparison. Sensitivity analysis based on different statistical assumptions was used to evaluate whether the results were robust. A two-step MR analysis was further conducted to explore the mediating effects of lipids, by integrating univariable MR and the multivariate MR method based on the Bayesian model averaging method (MR-BMA).

**Results:**

We identified four possible intestinal bacteria genera associated with the risk of endometriosis through the IVW method, including Eubacterium ruminantium group (odds ratio [OR] = 0.881, 95% CI: 0.795–0.976, *P* = 0.015), Anaerotruncus (OR = 1.252, 95% CI: 1.028–1.525, *P* = 0.025), Olsenella (OR = 1.110, 95% CI: 1.007–1.223, *P* = 0.036), and Oscillospira (OR = 1.215, 95% CI: 1.014–1.456, *P* = 0.035). The further two-step MR analysis identified that the effect of Olsenella on endometriosis was mediated by triglycerides (proportion mediated: 3.3%; 95% CI = 1.5−5.1%).

**Conclusion:**

This MR study found evidence for specific gut microbiomes associated with the risk of endometriosis, which might partially be mediated by triglycerides.

**Supplementary Information:**

The online version contains supplementary material available at 10.1186/s12944-024-02096-y.

## Introduction

Endometriosis is a chronic gynecological disease whose course is strongly influenced by estrogen [1]. It affects a significant proportion of women of reproductive age globally, ranging from 5 to 15% [2]. While traditionally considered benign, endometriosis shares certain characteristics with malignant tumors, including aggressive growth, recurrence, and metastasis [3]. As a result, endometriosis can lead to a reduced quality of life for patients and places a substantial economic burden on society [4]. At present, the etiology and pathogenesis of endometriosis are unclear, which poses a primary challenge to the development of clinical interventions to cure endometriosis [5].

In recent years, the relationship between human microbiota and female reproductive health has garnered significant attention [6, 7], and numerous studies have demonstrated variations in the diversity of gut microbiomes between women with endometriosis and those without [8–10]. Huang et al. reported an increase in the number of harmful *Eggerthella lenta* and *Eubacterium dolichum* in the gut of women with endometriosis and a significant decrease in the number of other protective microorganisms, particularly Clostridia, Ruminococcus, and Lachnospiraceae [8]. Chadchan et al. conducted a study on autograft-induced EMS mice, using antibiotics to inhibit lesion growth and fecal gavage to disrupt the gut microbiome. Their findings revealed that both lesion growth and associated inflammation were restored [11]. Despite these findings, the available evidence does not establish a causal relationship between the gut microbiome and endometriosis. Moreover, the susceptibility of the gut microbiome to various factors complicates the elucidation of their relationship.

Some studies have demonstrated that the increased risk of endometriosis may be related to abnormal lipid metabolism [12, 13]. Epidemiological studies, animal experiments, and Mendelian randomization (MR) Studies have demonstrated an association between gut microbiome and lipids [14–16]. Therefore, blood lipids may be a potential mediator between the gut microbiome and endometriosis.

In this work, we conducted an MR study, aiming to evaluate the causal effect of gut microbiome on the risk of endometriosis, as well as the potential mediating role of lipids. The results might shed light on the etiology of endometriosis and help make strategies for the clinical screening and prevention of endometriosis.

## Methods

### Study design

**Fig. 1:**
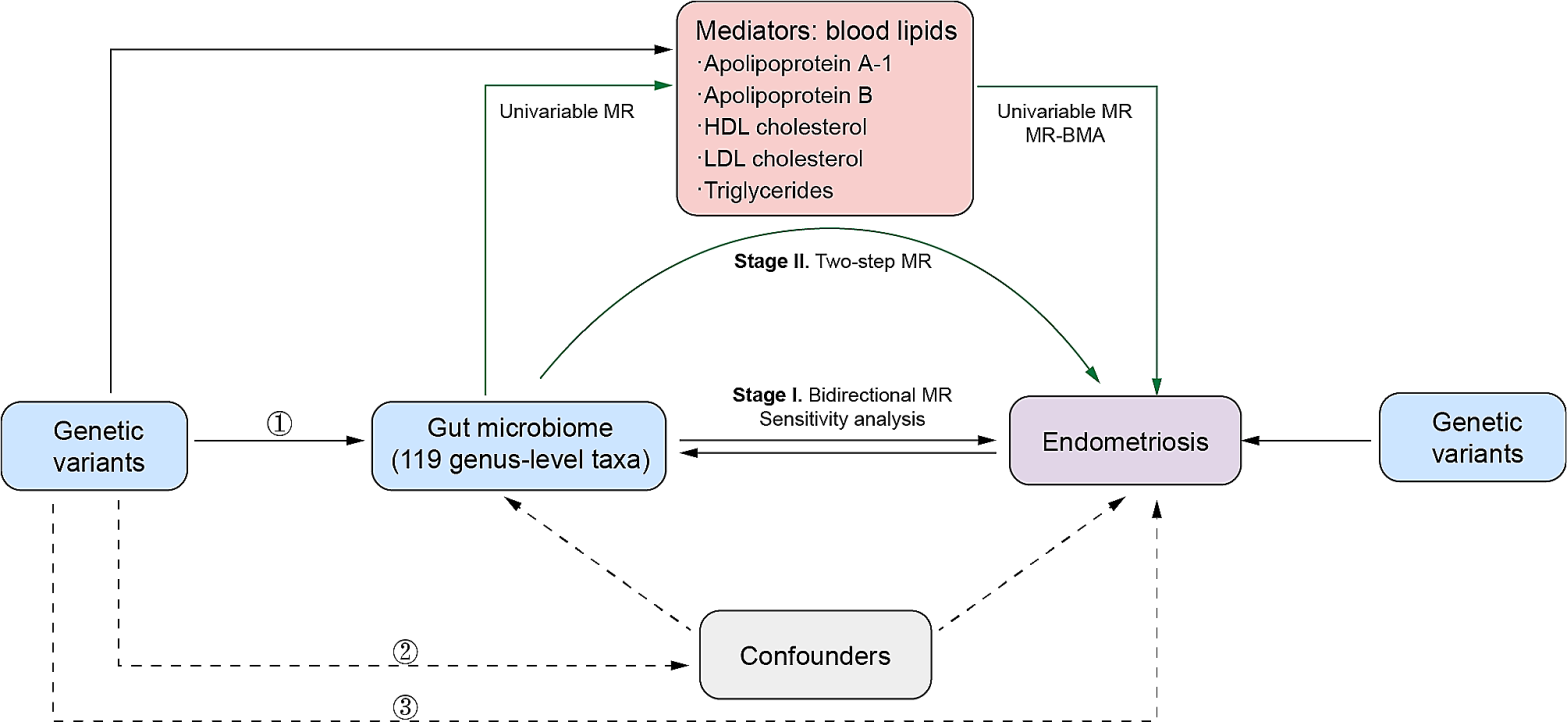
The flowchart of the study based on three assumptions: [1] Instrumental variables are significantly linked to exposure [2], Instrumental variables are not linked to confounders that affect the relationship between exposure and outcome, and [3] Instrumental variables are only linked to outcome through exposure

This work encompassed two stages of analysis. In stage I, we investigated the causal association between gut microbiome and endometriosis through a bidirectional MR. In stage II, we performed a two-step MR to establish a causal pathway from the gut microbiome to endometriosis, containing two analysis steps with lipids acting as mediators. In step 1, we identified the causative lipids in endometriosis by integrating univariable MR and MR based on Bayesian model averaging (MR-BMA). In step 2, we investigated the causal associations between the identified gut microbiota and lipids. Then we calculated the mediating effect of lipids in the association of gut microbiota with endometriosis. Figure 1 presents the study design. The selection of instrumental variables (IVs) was based on three assumptions: [1] IVs should be significantly associated with exposure [2], IVs are not associated with confounders that affect the relationship between exposure and outcome, and [3] IVs should affect the outcome only through the exposure.

### Data source

The genetic IVs for the gut microbiome were obtained from the research conducted by Kurilshikov et al. in the MiBioGen Consortium [17]. The data included the 16s rRNA gene sequencing profiles and whole genome genotyping data of 18,473 individuals from 12 countries, including the United Kingdom, the United States, and the Netherlands. The dataset also encompassed 122,110 genetic variation loci corresponding to 211 gut microbiome taxa at five hierarchical levels: phyla, kingdom, order, family, and genus. For our study, the genus was selected as the lowest classification level, and a total of 131 genera with an average abundance greater than 1% were identified, of which 12 could not be identified [17]. Therefore, we analyzed 119 genus-level taxa. The GWAS for blood lipids was obtained from the study conducted by Richardson et al., containing over 440,000 participants from the UK Biobank [18]. We obtained genetic associations with each lipid trait, including apolipoprotein A-1 (ApoA-1) (*n* = 393,193), apolipoprotein B (ApoB) (*n* = 439,214), high-density lipoprotein (HDL) cholesterol (*n* = 403,943), low-density lipoprotein (LDL) cholesterol (*n* = 440,546), and triglycerides (TG) (*n* = 441,016). The endometriosis GWAS data was derived from FinnGen, which was deposited in the IEU GWAS database (https://gwas.mrcieu.ac.uk/) with the GWAS ID “finn-b-N14_ENDOMETRIOSIS” [19]. Specifically, there were 8,288 cases diagnosed with endometriosis and 68,969 controls, and 16,377,306 single nucleotide polymorphisms (SNPs) were tested. There was no sample overlapping among the GWAS data of the exposure, outcome, or mediators.

### Stage I analysis

In stage I, we evaluated the causal effect of gut microbiome on endometriosis using univariable MR analysis. For the identified gut microbiome, we also conducted a reverse MR analysis to investigate the causal effect of endometriosis on them.

#### IVs selection

We implemented a series of quality-control steps in selecting the genetic IVs associated with the gut microbiome to ensure the precision and effectiveness of the study’s conclusions. First, we extracted the significant IVs for gut microbiome with a threshold of *P* < 1 × 10^− 5^ as a limited number of SNPs available at genome-wide significance cutoff (*P* < 5 × 10^− 8^) [20]. Second, we excluded SNPs exhibiting linkage disequilibrium (LD) with an r^2^ ≥ 0.001 to ensure the included SNPs were independent. Then the SNPs were extracted from the endometriosis dataset and harmonization was performed to align the effect allele and remove palindromic SNPs with intermediate minor allele frequency (maf > 0.42). We finally used the following formula to assess weak instrumental variable bias: F = R^2^ (n-k-1)/k(1-R^2^), where n represents the number of samples, k represents the number of SNPs, and R^2^ signifies the exposure variance. IVs with F-statistics < 10 were excluded [21]. When conducting reverse MR analysis, we used genetic IVs associated with endometriosis at *P* < 5 × 10^− 8^, and the consequent processing steps were as mentioned above. Supplementary Tables S1 and S2 present the characteristics of SNPs included in this study.

#### MR analysis

We tested the bidirectional effects between gut microbiome and endometriosis using several MR models, including inverse variance weighted (IVW), weighted median, simple mode, weighted mode, and MR-Egger regression. Given the IVW method is more efficient than other MR methods, this study used the IVW method as the primary model to test the causal effect [22]. Besides, considering the random-effects model for IVW was more conservative than the fix-effects model when existing heterogeneity, this MR study applied the random-effect model IVW as the major approach.

For sensitivity analysis, we initially assessed heterogeneity using Cochran’s Q test. The Cochran’s Q with *P* < 0.05 indicated that heterogeneity was detected. We then performed the “leave-one-out” analysis by omitting each instrumental SNP in turn. We also used the intercept term of the Egger regression to judge horizontal pleiotropy. The intercept with *P* < 0.05 suggested that horizontal pleiotropy could not be ruled out. Finally, the MR pleiotropy residual sum and outlier (MR-PRESSO) method was applied for testing and calculating the causal estimate following the removal of the identified outliers, once heterogeneity was detected in Cochran’s Q test [23].

In addition, to identify the independent effect of gut microbiome on endometriosis, we further conducted a multivariable MR (MVMR) analysis with the detected gut microbiome included in an MVMR IVW model.

### Stage II analysis

The stage II analysis was predominantly based on a two-step MR analysis containing two steps. In step 1, we first evaluated the causal effects of several lipid traits on endometriosis (β2); in step 2, we evaluated the causal associations between the identified gut microbiome and lipids (β1). Then the mediating effects of lipids were calculated.

#### Step1: evaluating the effect of lipids on endometriosis

To identify potential lipid traits associated with endometriosis, we first conducted an univariable MR analysis. Genetic IVs for several lipid traits, including ApoA-1, ApoB, HDL, LDL, and TG, were extracted with a P threshold at 5 × 10^− 8^. The processing steps for IVs and statistical analyses were as mentioned above. For the detected lipids in univariable MR analysis, we further conducted MR-BMA to prioritize them at the risk of endometriosis. The MR-BMA is the extension of multivariable MR, which allows for the analysis of high-throughput experiments. Zuber et al. found that in a realistic simulation study, MR-BMA could detect true causal risk factors even when the candidate risk factors were highly correlated [24]. Specifically, MR-BMA takes all possible combinations of the covariates into account and generates posterior probability for each combination model. The marginal inclusion probability (MIP) was determined by summing the posterior probabilities of all models, providing a ranking of causal associations with the outcome. Finally, model-averaged causal effects (MACE) were analyzed and the P-value of each lipid was calculated using the permutation method. The P-values were adjusted by several tests through the False Discovery Rate (FDR) procedure [28]. In addition, we also conducted an LD score regression (LDSC) analysis to investigate the genetic correlation among the lipids.

#### Step 2: evaluating the causal associations between the detected microbiome and lipids

Based on the analyses above, gut microbiome and lipids associated with endometriosis were identified. We then conducted a univariable MR analysis to detect levels of blood lipids attributed to gut microbiome, with the statistical methods mentioned above.

#### Calculating the mediating effect and proportion of lipids

The indirect effect mediated by lipids was calculated as β1 × β2, and the proportion of the mediating effect was calculated using β1 × β2 / β3, where β1 is the MR effect of the gut microbiome on lipids, β2 is the MR effect of lipids on endometriosis, and β3 represents the MR effect of the gut microbiome on endometriosis. The delta method was used to analyze 95% confidence intervals (CIs) [25]. MR estimates from MR-PRESSO were used to calculate the mediating effect when existing heterogeneity.

This analysis was performed using the R software (version 4.2.3). The “TwoSampleMR” and “MR-PRESSO” packages were used for statistical analysis. The MR-BMA was conducted based on the R-code deposited in GitHub (https://github.com/mglev1n/mrbma). The MR study was reported according to the MR-STROBE.

## Result

The IVs of 119 bacterial genera are presented in Supplementary Table S1. No weak IVs were included as F-statistics were over 10 for all the SNPs. For binary outcomes (endometriosis), the results were presented as odds ratio (OR); whereas for continuous outcomes (gut microbiome and lipids), the results were presented as beta. The corresponding 95% CIs were presented.

### Causal effects of the gut microbiome on endometriosis

**Fig. 2:**
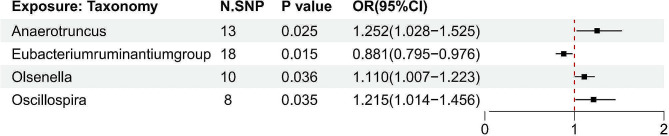
The forest plot shows the causal estimates between gut microbiome and endometriosis. The odds ratio and 95% confidence interval were obtained using the inverse–variance weighted method. OR, odds ratio; CI, confidence interval

**Fig. 3:**
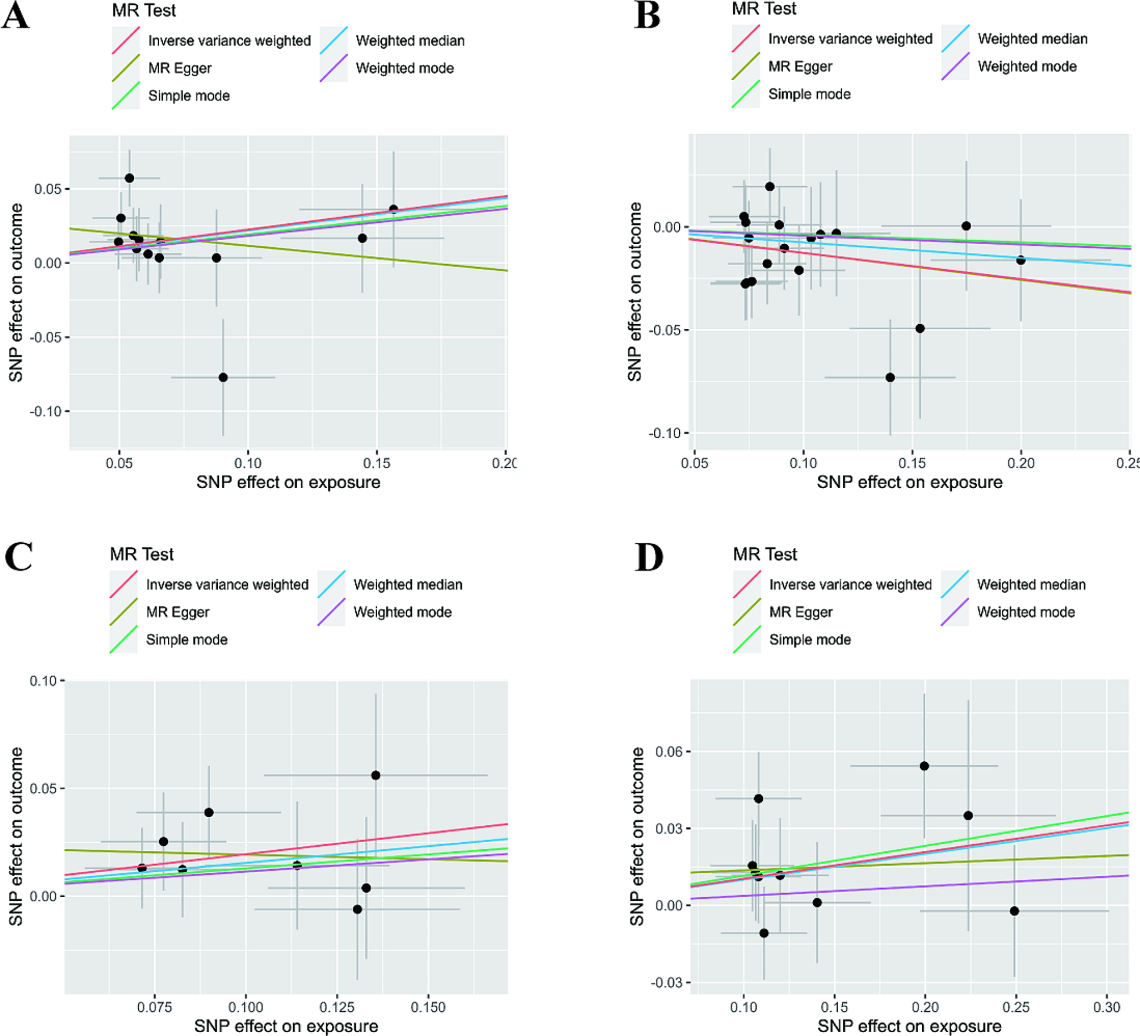
Scatter plots of significant causality between gut microbiome genera and endometriosis. **(A)** Anaerotruncus; **(B)** Eubacteriumruminantiumgroup; **(C)** Oscillospira; **(D)** Olsenella

We identified four possible intestinal bacteria genera through the IVW method (Figs. 2 and 3). Eubacterium ruminantium group exhibited a negative causal relationship with endometriosis (OR = 0.881, 95% CI: 0.795–0.976, *P* = 0.015), whereas the remaining three bacteria genera Anaerotruncus (OR = 1.252, 95% CI: 1.028–1.525, *P* = 0.025), Olsenella (OR = 1.110, 95% CI: 1.007–1.223, *P* = 0.036), and Oscillospira (OR = 1.215, 95% CI: 1.014–1.456, *P* = 0.035) were positively associated with the risk of endometriosis. Similar results were derived from other MR models, including weighted median, MR-Egger regression, simple mode, and weighted mode (Fig. 3).

**Fig. 4:**
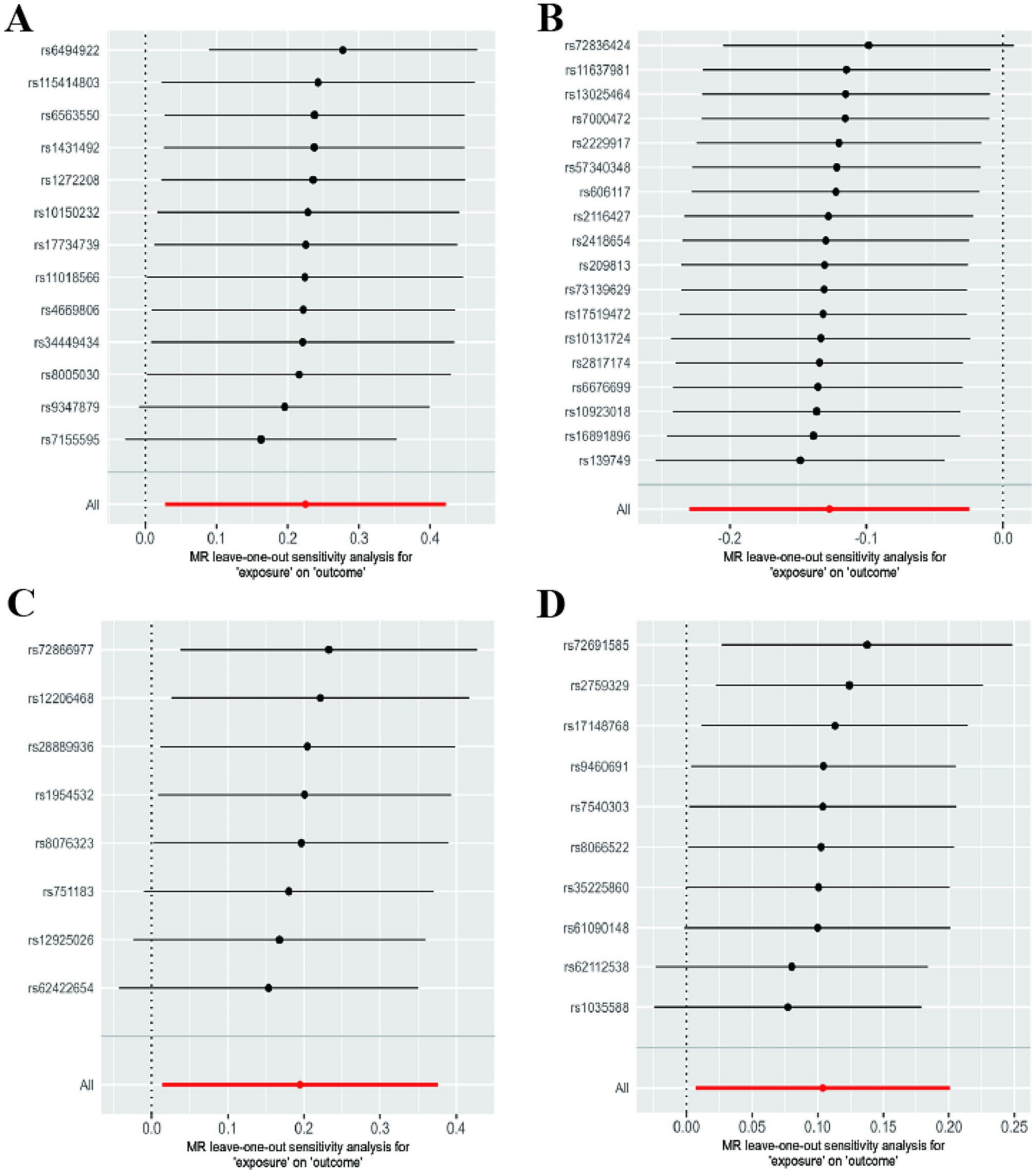
Leave-one-out analysis for the impact of individual SNPs on the association between GM and EMS risk. **(A)** Anaerotruncus; **(B)** Eubacterium ruminantium group; **(C)** Oscillospira; **(D)** Olsenella

Cochran’s IVW Q test indicated no marked heterogeneity among these IVs (Supplementary Table S3). Additionally, the P-values of the MR-Egger regression intercept all exceeded 0.05, suggesting the absence of obvious horizontal pleiotropy. The leave-one-out analysis also indicated that no SNPs altered the causal association signal (Fig. 4). Besides, the MR estimates were further supported by replicative analyses after removing outliers identified in MR-PRESSO (Supplementary Table S3).

Further MVMR analysis showed that Eubacterium ruminantium group (OR = 0.85, 95% CI: 0.77–0.94, *P* = 0.001), Olsenella (OR = 1.10, 95% CI: 1.01–1.20, *P* = 0.03), and Oscillospira (OR = 1.25, 95% CI: 1.08–1.46, *P* = 0.003) remained an independent effect on endometriosis, whereas the effect of Anaerotruncus was slightly attenuated (OR = 1.21, 95% CI: 0.99–1.47, *P* = 0.057).

Reverse MR analysis of the above four gut microbiomes revealed that endometriosis had a suggestive causal effect on Anaerotruncus (Beta = -0.061, 95% CI: -0.120 – -0.002, *P* = 0.041), whereas no bidirectional causal relationship was observed between the remaining three gut microbiome and endometriosis (Supplementary Table S4).

### Causal effect of blood lipids on endometriosis

Univariable MR analysis indicated that among the five included lipid traits, ApoA-1 (OR = 0.893, 95% CI: 0.817–0.975, *P* = 0.013), HDL cholesterol (OR = 0.899, 95% CI: 0.824–0.981, *P* = 0.018) and TG (OR = 1.159, 95% CI: 1.054–1.275, *P* = 0.002) had causal effects on endometriosis. The Cochran’s Q test showed considerable heterogeneity in some MR estimates (Table 1). However, the MR-Egger intercept test indicated no evidence of pleiotropy.

**Table 1:** Mendelian randomization analysis on the causal effect of blood lipids on endometriosis

Exposure	Method	OR	95% CI	P	P_FDR_	Intercept	Intercept P	Cochran P
Apolipoprotein A-1	Inverse variance weighted	0.898	0.818–0.986	0.025	0.041			0.037
	MR Egger	0.879	0.755–1.023	0.097		0.0008	0.725	0.034
	Simple mode	0.756	0.568–1.006	0.056				
	Weighted median	0.948	0.826–1.088	0.447				
	Weighted mode	0.902	0.787–1.032	0.134				
	MR-PRESSO	0.893	0.817–0.975	0.013				0.037
Apolipoprotein B	Inverse variance weighted	1.039	0.934–1.154	0.483	0.603			< 0.001
	MR Egger	0.952	0.827–1.097	0.501		0.005	0.076	< 0.001
	Simple mode	1.083	0.804–1.459	0.601				
	Weighted median	0.993	0.861–1.146	0.925				
	Weighted mode	0.956	0.845–1.082	0.479				
	MR-PRESSO	1.004	0.915–1.103	0.929				< 0.001
HDL cholesterol	Inverse variance weighted	0.892	0.812–0.979	0.016	0.040			< 0.001
	MR Egger	0.948	0.822–1.092	0.458		-0.002	0.266	< 0.001
	Simple mode	0.872	0.652–1.165	0.354				
	Weighted median	0.887	0.777–1.013	0.077				
	Weighted mode	0.893	0.785–1.016	0.087				
	MR-PRESSO	0.899	0.824–0.981	0.018				< 0.001
LDL cholesterol	Inverse variance weighted	1.019	0.904–1.150	0.755	0.755			< 0.001
	MR Egger	0.916	0.769–1.092	0.329		0.005	0.105	< 0.001
	Simple mode	0.948	0.689–1.304	0.741				
	Weighted median	0.942	0.807-1.100	0.454				
	Weighted mode	0.899	0.785–1.029	0.125				
	MR-PRESSO	0.969	0.873–1.075	0.550				< 0.001
Triglycerides	Inverse variance weighted	1.150	1.043–1.267	0.005	0.025			< 0.001
	MR Egger	1.158	1.002–1.337	0.048		-0.0003	0.900	< 0.001
	Simple mode	1.354	0.983–1.866	0.065				
	Weighted median	1.062	0.926–1.218	0.388				
	Weighted mode	1.130	0.990–1.291	0.072				
	MR-PRESSO	1.159	1.054–1.275	0.002				< 0.001

**Fig. 5:**
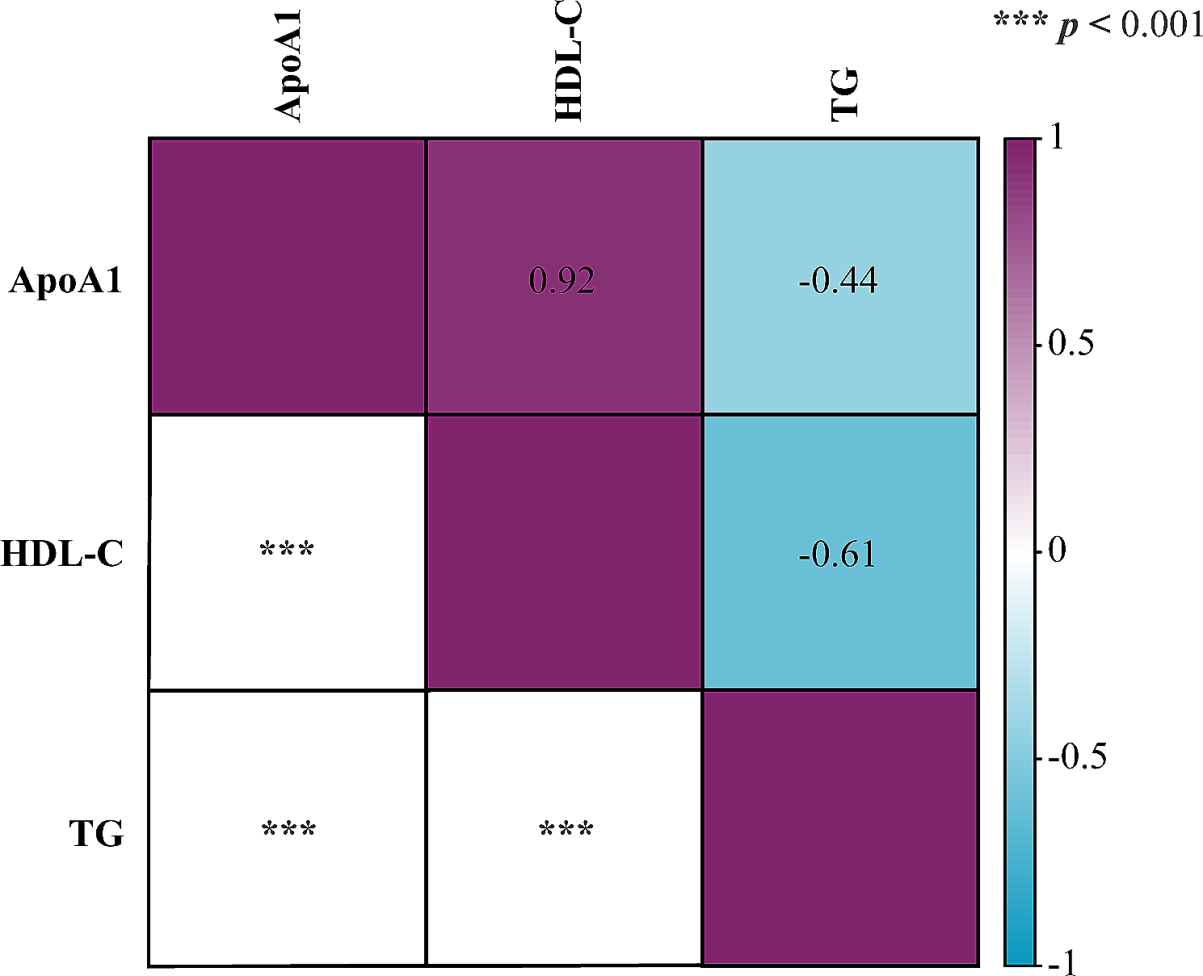
Heat map show the genetic correlation estimates from linkage disequilibrium score regression among selected metabolites. The red indicated a positive, while the blue indicated a negative correlation. The number marked in the upper right part of the figure is the correlation coefficient, and the lower left part is the P values of the LDSC test. ApoA1, apolipoprotein A1; HDL-C, high-density lipoprotein cholesterol; TG, triglycerides

Using MR-BMA, TG was identified as the top-ranked lipid most strongly associated with endometriosis (MIP = 0.839, MACE = 0.117, P_FDR_ = 0.039) (Tables 2 and 3). Through the LDSC, we determined that there were significantly strong genetic correlations among TG, ApoA-1, and HDL cholesterol (Fig. 5).

**Table 2:** Ranking of models for endometriosis according to their posterior probability after exclusion of outlying and influential variants in MR-BMA analysis

Models	Posterior probability	Causal estimate
Triglycerides	0.817	0.139
HDL cholesterol	0.103	-0.092
Apolipoprotein A-1	0.053	-0.077
Apolipoprotein A-1, Triglycerides	0.011	-0.034, 0.128
HDL cholesterol, Triglycerides	0.011	-0.029, 0.122
Apolipoprotein A-1, HDL cholesterol	0.005	0.147, -0.223
Apolipoprotein A-1, HDL cholesterol, Triglycerides	0	-0.092, 0.063, 0.145

**Table 3:** Prioritization of causal blood lipids of endometriosis using the MR-BMA method

Blood lipids	MIP	MACE	Empirical P-value	FDR-adjusted P-value
Triglycerides	0.839	0.117	0.013	0.039
HDL cholesterol	0.119	-0.011	0.984	0.992
Apolipoprotein A-1	0.069	-0.004	0.992	0.992

### Causal effect of gut microbiome on lipid traits

We further conducted a univariable MR analysis to investigate the causal effects of Eubacterium ruminantium group, Anaerotruncus, Olsenella, and Oscillospira on TG, The IVW showed that Olsenella exhibited a causal effect on TG (Beta = 0.023, 95% CI: 0.005–0.041, *P* = 0.011). No causal associations were observed in the other three microbiomes.

### Mediation analysis

**Fig. 6:**
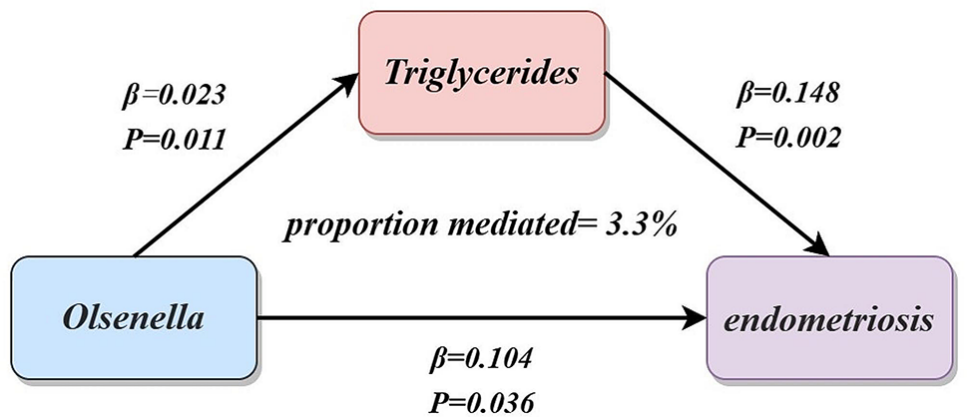
The triglycerides mediated the causal effect of Olsenella on endometriosis. The β value between Olsenella and triglycerides and endometriosis are the MR estimates using the inverse variance weighted method. The β value between triglycerides and endometriosis are the Mendelian randomization estimates using the Mendelian randomization pleiotropy residual sum and outlier method

Resultantly, we analyzed TG as the mediator of the Olsenella-endometriosis pathway (Fig. 6). Using the product of coefficients, we found the mediating effect of TG in the Olsenella-endometriosis pathway was 0.003, with a mediating proportion of 3.3% (95% CI = 1.5 − 5.1%, *P* = 0.048).

## Discussion

In this study, we found that genetic liability for a higher abundance of Eubacterium ruminantium group was associated with a lower risk of endometriosis, whereas genetic liability for a higher abundance of Anaerotruncus, Olsenella, and Oscillospira, was associated with a higher risk of endometriosis. Further mediation analysis revealed that TG partially mediated the detrimental effect of Olsenella on endometriosis. The findings in this MR work shed light on the screening and prevention of endometriosis by examining the gut microbiome. On the one hand, examining fecal bacteria might be a promising strategy to screen populations with a higher risk of endometriosis; on the other hand, regulating the intestinal flora microenvironment might be a novel strategy to prevent endometriosis in the future. In addition, this MR work also implies the involvement of lipid traits (TG) in the gut microbiome-endometriosis pathway, suggesting modulating TG could also be a promising strategy for endometriosis prevention.

Animal studies have demonstrated that gut microbiome plays a crucial pro-inflammatory role in the progression of endometriosis and results in higher levels of IL-1β, TNF-α, IL-6, and TGF-β1 and an increase in macrophages at the lesion site [26]. Short-chain fatty acids, like acetate, propionate, and n-butyrate, are bioactive metabolites produced through the processing of indigestible nutrients by the gut microbiome [27, 28]. Butyrate is the main energy source for colon epithelial cells and plays a crucial role in maintaining the integrity of the intestinal barrier [29, 30]. Research findings indicate that n-butyrate inactivates the pro-growth RAP1 signaling pathway by activating GPR43 and GPR109A, inhibiting histone deacetylase activity and activating the expression of RAP1GAP. This process then inhibits the development of endometriosis lesions and inflammatory cell infiltration [11]. Therefore, once the gut microbiome is disturbed, the immune integrity of the intestinal wall will be decreased, and the permeability will be increased, causing bacterial endotoxins in the intestinal tract to enter the blood and consequently trigger various inflammatory reactions in the body to induce the occurrence of epithelial-mesenchymal transition (EMT) [31–38]. Studies have demonstrated that the pathogenesis of EMT is consistent with the pathogenesis of endometriosis. It has been proven that EMT plays a crucial role in the invasion of ectopic endometrium and is a key step in the successful invasion of ectopic endometrium into the external position of the uterine cavity [39].

PD-1 and PD-L1 are overexpressed in endometriosis, and their overactivation promotes the development of endometriosis [40]. Additionally, studies have demonstrated that the immune factor IL-27 in endometriosis accelerates the development of endometriosis by triggering the production of IL-10 by Th17 and promoting the proliferation and implantation of ectopic lesions [41]. Therefore, the successful implantation and transfer of ectopic endometrium outside the uterus is closely related to immune tolerance, especially the immune tolerance caused by PD-1/PD-L1 and IL-27 overexpression [42–44]. Numerous studies have shown that endometriosis patients exhibit immunological tolerance of T cells, which includes immunosuppression and the depletion of helper T cells or cytotoxic T cell [45, 46]. This lowers the immune clearance ability and promotes the emergence of immune escape [47, 48]. In our study, Olsenella exhibited a causal association with an increased risk of endometriosis. However, studies have demonstrated that Olsenella can enhance the activity of anti-PD-1 antibodies by producing the metabolite inosine to activate antitumor T cells [49]. Therefore, the association between Olsenella and endometriosis deserves further study.

In addition, estrogen metabolism might be another potential mechanism worth discussing. Endometriosis is an estrogen-driven disease, and high estrogen levels are considered to be an important pathogenic factor in endometriosis [50, 51]. The metabolism of estrogen mainly occurs in the liver, which can produce sex hormone-binding globulin (SHBG). Estrogen loses its biological activity after binding to SHBG [52]. Conjugated estrogens are excreted in the bile and enter the intestine, uncoupled by beta-glucuronidase secreted by the gut microbiome, reabsorbed through the intestinal mucosa, and enter the circulatory system through the portal vein [53]. Ervin et al. reported that beta-glucuronidase secreted by the gut microbiome can affect intestinal estrogen metabolism and the growth of estrogen-dependent tumors [54]. An imbalance of the gut microbiome leads to a large production of beta-glucuronidase, causing the levels of circulating estrogen metabolites to increase, stimulating the growth of endometrial tissue, and promoting the occurrence and development of endometriosis [55, 56].

Except for the potential immunomodulatory and estrogen metabolism mechanisms proposed above, our study further identified TG partially mediated the causal association between Olsenella and endometriosis, suggesting blood lipid metabolism might be the underlying mechanism linking Olsenella with an increased risk of endometriosis. Specifically, the role of lipids in mediating the effect of gut microbiome on health outcomes has been investigated in previous studies. For example, Dai et al. found that ApoB accounted for nearly 10% of the effect of species *B. dorei* on heart failure [57]. As mentioned above, we predominantly proposed the mediating role of TG in the pathway from gut microbiome to endometriosis. Despite this, other potential pathways warrant further investigation. Except for TG, previous studies have reported enriched ApoB mRNA Editing Enzyme Catalytic Subunit (APOBEC) signature mutations were higher in endometriosis patients, indicating APOBEC-mediated mutagenesis might drive genomic heterogeneity in endometriosis [58]. In addition, the coagulation function might be another mechanism that should be noted. One GWAS study has reported that LDL and TC were associated with an increased risk of pulmonary embolism, suggesting the lipids traits were involved in coagulation pathways [59]. Another MR study has demonstrated the causal associations between ADAMTS13/vWF and the risk of endometriosis, suggesting the involvement of coagulation factors in endometriosis development [60].

The study had some limitations. First, our study was limited to European patients, and this demographic bias limits our study and its applicability to other ethnic groups [61]. Second, this study was limited to patients diagnosed with ovarian endometriosis and did not consider intestinal endometriosis, peritoneal endometriosis, and pelvic endometriosis. For the above two limitations, further study is needed to expand the investigation to diverse populations and other forms of endometriosis to enhance generalizability when future GWAS data containing various subtypes of endometriosis from different ancestries is available. Third, the current study could not rule out the bias from the complex interaction between gut microbiome and host metabolism. Fourth, given that only summary-level statistics are publicly available, stratification analysis like sex stratification is unavailable. Though the MR design is less likely prone to confounders (including gender and age), future investigation focusing on stratification analysis when stratified GWAS data is available would improve the reliability of the results. Fifth, in this exploratory MR work, we did not conduct multiple testing corrections for p values that might miss out on certain significant genera potentially associated with the occurrence of endometriosis. Instead, we used *P* < 0.05 as the threshold to determine the significant estimates. This strategy might help preliminarily screen potential genera involved in endometriosis development. Sixth, we used a relaxed P threshold to extract IVs for gut microbiome, which has been widely used in previous MR studies. This might be prone to including weak instruments, but the F-statistics for each of the SNPs suggested no weak IVs were included (all F > 10). Seventh, the leave-one-out analysis showed some high influence points driving the overall estimates, which would potentially make the MR results over-estimated. This might be owing to the relatively limited number of SNPs included in the MR work, though a relaxed significance threshold for the associations between SNPs and gut microbiome was used in this work. Further investigation is warranted using gut microbiome GWAS with greater sequencing depth to validate our results. Eighth, our study suggests that a small part of the causal effect of the gut microbiome on endometriosis is mediated by blood lipids. Therefore, further research is needed to quantify other potential mediators. Finally, several issues should be also noted in terms of the inherent nature of the MR approach, including pleiotropy, measurement error in GWAS data, and the assumption that genetic variants accurately reflect the lifetime exposure to microbiome composition or lipid levels.

## Conclusion

Our study shows evidence for gut microbiome involved in endometriosis development and identified TG might partially mediate the effect of Olsenella on endometriosis risk. This study provides new insights into the etiology of endometriosis and might help set out strategies for endometriosis screening and prevention.

### Electronic supplementary material

Below is the link to the electronic supplementary material.

Supplementary Material 1

## Data Availability

The datasets used and/or analysed during the current study are available from the corresponding author on reasonable request.

## Abbreviations

MR: Mendelian randomization
IVW: Inverse variance weighting
MR-PRESSO: Mendelian Randomized Polymorphism Residual and Outlier
MR-BMA: Bayesian model averaging method
OR: Odds ratio
MIP: Marginal inclusion probability
MACE: Model-averaged causal effects
FDR: False Discovery Rate
EMS: Endometriosis
GWAS: Genome-wide association studies
ApoA-1: Apolipoprotein A-1
ApoB: Apolipoprotein B
HDL-C: High-density lipoprotein cholesterol
LDL-C: Low-density lipoprotein cholesterol
TG: Triglycerides
SNP: Single nucleotide polymorphism
LDSC: Linkage disequilibrium score
CI: Confidence interval
GM: Gut microbiome
EMT: Epithelial-mesenchymal transition
SHBG: Sex hormone-binding globulin

## References

[1] Taylor HS, Kotlyar AM, Flores VA (2021). Endometriosis is a chronic systemic disease: clinical challenges and novel innovations. Lancet (London England).

[2] Peiris AN, Chaljub E, Medlock D, Endometriosis (2018). JAMA.

[3] Giudice LC (2010). Clinical practice. Endometr New Engl J Med.

[4] Simoens S, Dunselman G, Dirksen C, Hummelshoj L, Bokor A, Brandes I (2012). The burden of endometriosis: costs and quality of life of women with endometriosis and treated in referral centres. Hum Reprod (Oxford England).

[5] Burney RO, Giudice LC (2012). Pathogenesis and pathophysiology of endometriosis. Fertil Steril.

[6] Martin DH (2012). The microbiota of the vagina and its influence on women’s health and disease. Am J Med Sci.

[7] Nelson DB, Rockwell LC, Prioleau MD, Goetzl L (2016). The role of the bacterial microbiota on reproductive and pregnancy health. Anaerobe.

[8] Huang L, Liu B, Liu Z, Feng W, Liu M, Wang Y (2021). Gut microbiota exceeds cervical microbiota for early diagnosis of endometriosis. Front Cell Infect Microbiol.

[9] Svensson A, Brunkwall L, Roth B, Orho-Melander M, Ohlsson B. Associations Between Endometriosis and Gut Microbiota. Reproductive sciences (Thousand Oaks, Calif). 2021;28(8):2367-77. 10.1007/s43032-021-00506-5 PMC8289757 33660232

[10] Shan J, Ni Z, Cheng W, Zhou L, Zhai D, Sun S (2021). Gut microbiota imbalance and its correlations with hormone and inflammatory factors in patients with stage 3/4 endometriosis. Arch Gynecol Obstet.

[11] Chadchan SB, Cheng M, Parnell LA, Yin Y, Schriefer A, Mysorekar IU (2019). Antibiotic therapy with metronidazole reduces endometriosis disease progression in mice: a potential role for gut microbiota. Hum Reprod (Oxford England).

[12] Peinado FM, Olivas-Martínez A, Iribarne-Durán LM, Ubiña A, León J, Vela-Soria F (2023). Cell cycle, apoptosis, cell differentiation, and lipid metabolism gene expression in endometriotic tissue and exposure to parabens and benzophenones. Sci Total Environ.

[13] Dutta M, Anitha M, Smith PB, Chiaro CR, Maan M, Chaudhury K (2016). Metabolomics reveals altered lipid metabolism in a mouse model of endometriosis. J Proteome Res.

[14] Liu R, Hong J, Xu X, Feng Q, Zhang D, Gu Y (2017). Gut microbiome and serum metabolome alterations in obesity and after weight-loss intervention. Nat Med.

[15] Zhou X, Lian P, Liu H, Wang Y, Zhou M, Feng Z. Causal associations between Gut Microbiota and different types of Dyslipidemia: a two-sample mendelian randomization study. Nutrients. 2023;15(20). 10.3390/nu15204445 PMC10609956 37892520

[16] Liu X, Tong X, Zou Y, Lin X, Zhao H, Tian L (2022). Mendelian randomization analyses support causal relationships between blood metabolites and the gut microbiome. Nat Genet.

[17] Kurilshikov A, Medina-Gomez C, Bacigalupe R, Radjabzadeh D, Wang J, Demirkan A (2021). Large-scale association analyses identify host factors influencing human gut microbiome composition. Nat Genet.

[18] Richardson TG, Sanderson E, Palmer TM, Ala-Korpela M, Ference BA, Davey Smith G (2020). Evaluating the relationship between circulating lipoprotein lipids and apolipoproteins with risk of coronary heart disease: a multivariable mendelian randomisation analysis. PLoS Med.

[19] Garitazelaia A, Rueda-Martínez A, Arauzo R, de Miguel J, Cilleros-Portet A, Marí S et al. A Systematic Two-Sample Mendelian Randomization Analysis Identifies Shared Genetic Origin of Endometriosis and Associated Phenotypes. Life (Basel, Switzerland). 2021;11(1). 10.3390/life11010024 PMC7824623 33401535

[20] Sanna S, van Zuydam NR, Mahajan A, Kurilshikov A, Vich Vila A, Võsa U (2019). Causal relationships among the gut microbiome, short-chain fatty acids and metabolic diseases. Nat Genet.

[21] Burgess S, Thompson SG (2011). Avoiding bias from weak instruments in mendelian randomization studies. Int J Epidemiol.

[22] Burgess S, Butterworth A, Thompson SG (2013). Mendelian randomization analysis with multiple genetic variants using summarized data. Genet Epidemiol.

[23] Verbanck M, Chen CY, Neale B, Do R (2018). Detection of widespread horizontal pleiotropy in causal relationships inferred from mendelian randomization between complex traits and diseases. Nat Genet.

[24] Zuber V, Colijn JM, Klaver C, Burgess S (2020). Selecting likely causal risk factors from high-throughput experiments using multivariable mendelian randomization. Nat Commun.

[25] Thompson JR, Minelli C, Del Greco MF. Mendelian randomization using Public Data from Genetic Consortia. Int J Biostatistics. 2016;12(2). 10.1515/ijb-2015-0074 27092657

[26] Talwar C, Singh V, Kommagani R (2022). The gut microbiota: a double-edged sword in endometriosis†. Biol Reprod.

[27] Li Z, Quan G, Jiang X, Yang Y, Ding X, Zhang D (2018). Effects of metabolites derived from gut microbiota and hosts on pathogens. Front Cell Infect Microbiol.

[28] Kho ZY, Lal SK (2018). The human gut microbiome - A potential Controller of Wellness and Disease. Front Microbiol.

[29] Kim M, Friesen L, Park J, Kim HM, Kim CH (2018). Microbial metabolites, short-chain fatty acids, restrain tissue bacterial load, chronic inflammation, and associated cancer in the colon of mice. Eur J Immunol.

[30] Paradis T, Bègue H, Basmaciyan L, Dalle F, Bon F. Tight junctions as a Key for pathogens Invasion in Intestinal epithelial cells. Int J Mol Sci. 2021;22(5). 10.3390/ijms22052506 PMC7958858 33801524

[31] Dethlefsen L, McFall-Ngai M, Relman DA (2007). An ecological and evolutionary perspective on human-microbe mutualism and disease. Nature.

[32] Turnbaugh PJ, Ley RE, Mahowald MA, Magrini V, Mardis ER, Gordon JI (2006). An obesity-associated gut microbiome with increased capacity for energy harvest. Nature.

[33] Cani PD, Bibiloni R, Knauf C, Waget A, Neyrinck AM, Delzenne NM (2008). Changes in gut microbiota control metabolic endotoxemia-induced inflammation in high-fat diet-induced obesity and diabetes in mice. Diabetes.

[34] Cani PD, Possemiers S, Van de Wiele T, Guiot Y, Everard A, Rottier O (2009). Changes in gut microbiota control inflammation in obese mice through a mechanism involving GLP-2-driven improvement of gut permeability. Gut.

[35] Goodman AL, Kallstrom G, Faith JJ, Reyes A, Moore A, Dantas G (2011). Extensive personal human gut microbiota culture collections characterized and manipulated in gnotobiotic mice. Proc Natl Acad Sci USA.

[36] Cani PD, Amar J, Iglesias MA, Poggi M, Knauf C, Bastelica D (2007). Metabolic endotoxemia initiates obesity and insulin resistance. Diabetes.

[37] Wellen KE, Hotamisligil GS (2005). Inflammation, stress, and diabetes. J Clin Investig.

[38] Ying J, Zhou HY, Liu P, You Q, Kuang F, Shen YN (2018). Aspirin inhibited the metastasis of colon cancer cells by inhibiting the expression of toll-like receptor 4. Cell Bioscience.

[39] Xiong W, Zhang L, Yu L, Xie W, Man Y, Xiong Y (2015). Estradiol promotes cells invasion by activating β-catenin signaling pathway in endometriosis. Reprod (Cambridge England).

[40] Suszczyk D, Skiba W, Zardzewiały W, Pawłowska A, Włodarczyk K, Polak G et al. Clinical value of the PD-1/PD-L1/PD-L2 pathway in patients suffering from Endometriosis. Int J Mol Sci. 2022;23(19). 10.3390/ijms231911607 PMC9570092 36232911

[41] Chang KK, Liu LB, Jin LP, Zhang B, Mei J, Li H (2017). IL-27 triggers IL-10 production in Th17 cells via a c-Maf/RORγt/Blimp-1 signal to promote the progression of endometriosis. Cell Death Dis.

[42] Riella LV, Paterson AM, Sharpe AH, Chandraker A (2012). Role of the PD-1 pathway in the immune response. Am J Transplantation: Official J Am Soc Transplantation Am Soc Transpl Surg.

[43] Okazaki T, Honjo T (2006). The PD-1-PD-L pathway in immunological tolerance. Trends Immunol.

[44] Francisco LM, Sage PT, Sharpe AH (2010). The PD-1 pathway in tolerance and autoimmunity. Immunol Rev.

[45] Wong YC, Tay SS, McCaughan GW, Bowen DG, Bertolino P (2015). Immune outcomes in the liver: is CD8 T cell fate determined by the environment?. J Hepatol.

[46] Horst AK, Neumann K, Diehl L, Tiegs G (2016). Modulation of liver tolerance by conventional and nonconventional antigen-presenting cells and regulatory immune cells. Cell Mol Immunol.

[47] Dong H, Strome SE, Salomao DR, Tamura H, Hirano F, Flies DB (2002). Tumor-associated B7-H1 promotes T-cell apoptosis: a potential mechanism of immune evasion. Nat Med.

[48] Anderson AC, Joller N, Kuchroo VK, Lag-3. Tim-3, and TIGIT: co-inhibitory receptors with Specialized functions in Immune Regulation. Immunity. 2016;44(5):989–1004. 10.1016/j.immuni.2016.05.001 PMC4942846 27192565

[49] Mager LF, Burkhard R, Pett N, Cooke NCA, Brown K, Ramay H (2020). Microbiome-derived inosine modulates response to checkpoint inhibitor immunotherapy. Sci (New York NY).

[50] Galvankar M, Singh N, Modi D (2017). Estrogen is essential but not sufficient to induce endometriosis. J Biosci.

[51] Clemenza S, Vannuccini S, Ruotolo A, Capezzuoli T, Petraglia F (2022). Advances in targeting estrogen synthesis and receptors in patients with endometriosis. Expert Opin Investig Drugs.

[52] Flores R, Shi J, Fuhrman B, Xu X, Veenstra TD, Gail MH (2012). Fecal microbial determinants of fecal and systemic estrogens and estrogen metabolites: a cross-sectional study. J Translational Med.

[53] Hu S, Ding Q, Zhang W, Kang M, Ma J, Zhao L (2023). Gut microbial beta-glucuronidase: a vital regulator in female estrogen metabolism. Gut Microbes.

[54] Ervin SM, Li H, Lim L, Roberts LR, Liang X, Mani S (2019). Gut microbial β-glucuronidases reactivate estrogens as components of the estrobolome that reactivate estrogens. J Biol Chem.

[55] Baker JM, Al-Nakkash L, Herbst-Kralovetz MM (2017). Estrogen-gut microbiome axis: physiological and clinical implications. Maturitas.

[56] Laschke MW, Menger MD (2016). The gut microbiota: a puppet master in the pathogenesis of endometriosis?. Am J Obstet Gynecol.

[57] Dai H, Hou T, Wang Q, Hou Y, Wang T, Zheng J (2023). Causal relationships between the gut microbiome, blood lipids, and heart failure: a mendelian randomization analysis. Eur J Prev Cardiol.

[58] Revathidevi S, Nakaoka H, Suda K, Fujito N, Munirajan AK, Yoshihara K (2022). APOBEC mediated mutagenesis drives genomic heterogeneity in endometriosis. J Hum Genet.

[59] Zhang Z, Li H, Weng H, Zhou G, Chen H, Yang G (2023). Genome-wide association analyses identified novel susceptibility loci for pulmonary embolism among Han Chinese population. BMC Med.

[60] Li Y, Liu H, Ye S, Zhang B, Li X, Yuan J (2023). The effects of coagulation factors on the risk of endometriosis: a mendelian randomization study. BMC Med.

[61] Tan JS, Yan XX, Wu Y, Gao X, Xu XQ, Jiang X (2021). Rare variants in MTHFR predispose to occurrence and recurrence of pulmonary embolism. Int J Cardiol.

