# Supplementary material for: Blood lipids mediate the effects of gut microbiome on endometriosis: a mendelian randomization study
Source: Lipids Health Dis. 2024 Apr 16;23:110. doi: 10.1186/s12944-024-02096-y (PMC11020997; doi:10.1186/s12944-024-02096-y)
Supplement: Supplementary file 1 — Supplementary Material 1 [file 12944_2024_2096_MOESM1_ESM.docx]

Supplementary Material

# Supplementary Tables

|  | **Table S1 Instrumental variables used in MR analysis of the association between** **gut microbiome and endometriosis.** | | | | | | | | | | | | |
| --- | --- | --- | --- | --- | --- | --- | --- | --- | --- | --- | --- | --- | --- |
| **Bacterial taxa (exposure)** | | **SNP** | **Effect allele** | **Other allele** | **EAF** | **Exposure (Bacteria)** | | | | **Outcome (PE)** | | |  |
|  |  |  |  |  |  | **Beta** | **SE** | **F statistics** | **P-value** | **Beta** | **SE** | **P-value** | |
| *Actinomyces* | | rs2715439 | C | T | 0.454 | 0.075 | 0.016 | 20.7 | 6.27E-06 | -0.002 | 0.018 | 0.897 | |
| *Actinomyces* | | rs34583783 | G | T | 0.102 | 0.127 | 0.027 | 22.1 | 4.49E-06 | 0.032 | 0.037 | 0.379 | |
| *Actinomyces* | | rs35011108 | A | G | 0.064 | 0.233 | 0.051 | 22.8 | 6.34E-06 | -0.005 | 0.035 | 0.897 | |
| *Actinomyces* | | rs4073240 | G | A | 0.383 | 0.075 | 0.017 | 19.9 | 7.94E-06 | -0.006 | 0.018 | 0.756 | |
| *Actinomyces* | | rs4146653 | G | A | 0.197 | 0.099 | 0.021 | 23.0 | 4.50E-06 | 0.031 | 0.025 | 0.223 | |
| *Actinomyces* | | rs71315246 | A | G | 0.143 | -0.097 | 0.022 | 17.3 | 9.83E-06 | -0.063 | 0.026 | 0.015 | |
| *Actinomyces* | | rs7915461 | T | C | 0.058 | 0.188 | 0.040 | 20.3 | 5.92E-06 | -0.037 | 0.035 | 0.285 | |
| *Adlercreutzia* | | rs11604400 | C | T | 0.115 | -0.103 | 0.023 | 17.4 | 9.74E-06 | -0.024 | 0.029 | 0.399 | |
| *Adlercreutzia* | | rs13231526 | C | A | 0.064 | 0.143 | 0.031 | 19.7 | 4.81E-06 | 0.044 | 0.032 | 0.174 | |
| *Adlercreutzia* | | rs2717140 | C | T | 0.111 | -0.119 | 0.025 | 23.2 | 2.05E-06 | -0.039 | 0.029 | 0.184 | |
| *Adlercreutzia* | | rs55719207 | G | A | 0.418 | -0.070 | 0.016 | 19.6 | 9.61E-06 | 0.010 | 0.018 | 0.588 | |
| *Adlercreutzia* | | rs6664405 | T | C | 0.144 | -0.095 | 0.021 | 18.1 | 5.23E-06 | 0.021 | 0.025 | 0.401 | |
| *Adlercreutzia* | | rs7680684 | C | T | 0.325 | -0.083 | 0.017 | 25.2 | 9.77E-07 | 0.021 | 0.018 | 0.248 | |
| *Adlercreutzia* | | rs9490822 | C | T | 0.447 | -0.073 | 0.016 | 22.0 | 2.54E-06 | -0.016 | 0.018 | 0.372 | |
| *Adlercreutzia* | | rs9915817 | T | C | 0.329 | 0.075 | 0.017 | 20.5 | 8.22E-06 | 0.019 | 0.019 | 0.324 | |
| *Akkermansia* | | rs111862613 | T | C | 0.166 | 0.091 | 0.020 | 26.8 | 3.39E-06 | 0.004 | 0.024 | 0.869 | |
| *Akkermansia* | | rs117107102 | A | G | 0.045 | 0.204 | 0.043 | 11.8 | 3.01E-06 | -0.027 | 0.041 | 0.509 | |
| *Akkermansia* | | rs11729256 | T | C | 0.239 | 0.075 | 0.015 | 24.3 | 6.58E-07 | 0.003 | 0.023 | 0.891 | |
| *Akkermansia* | | rs12908520 | G | A | 0.456 | 0.062 | 0.013 | 22.5 | 2.26E-06 | 0.018 | 0.018 | 0.303 | |
| *Akkermansia* | | rs2602429 | C | T | 0.222 | 0.075 | 0.016 | 22.4 | 2.72E-06 | 0.005 | 0.020 | 0.804 | |
| *Akkermansia* | | rs4242783 | G | A | 0.287 | 0.069 | 0.015 | 22.5 | 3.00E-06 | -0.001 | 0.020 | 0.947 | |
| *Akkermansia* | | rs4936098 | A | G | 0.340 | 0.065 | 0.014 | 22.5 | 1.10E-06 | 0.010 | 0.018 | 0.579 | |
| *Akkermansia* | | rs61779207 | G | A | 0.178 | -0.076 | 0.017 | 19.6 | 6.32E-06 | -0.005 | 0.021 | 0.828 | |
| *Akkermansia* | | rs74542928 | T | C | 0.079 | 0.113 | 0.024 | 20.6 | 1.48E-06 | 0.041 | 0.041 | 0.323 | |
| *Akkermansia* | | rs9349825 | A | G | 0.279 | -0.070 | 0.015 | 23.7 | 2.60E-06 | -0.038 | 0.023 | 0.092 | |
| *Akkermansia* | | rs941682 | G | A | 0.311 | -0.063 | 0.014 | 20.3 | 9.17E-06 | -0.016 | 0.020 | 0.414 | |
| *Alistipes* | | rs1107244 | G | A | 0.079 | 0.076 | 0.017 | 14.6 | 3.59E-06 | 0.003 | 0.033 | 0.916 | |
| *Alistipes* | | rs11769002 | G | A | 0.401 | -0.053 | 0.011 | 23.6 | 1.45E-06 | -0.013 | 0.018 | 0.478 | |
| *Alistipes* | | rs11958296 | A | G | 0.071 | -0.098 | 0.022 | 20.5 | 9.30E-06 | 0.003 | 0.043 | 0.945 | |
| *Alistipes* | | rs12990744 | C | T | 0.123 | -0.078 | 0.017 | 21.8 | 8.21E-06 | 0.061 | 0.029 | 0.038 | |
| *Alistipes* | | rs1689282 | A | C | 0.340 | -0.052 | 0.011 | 20.8 | 5.28E-06 | -0.017 | 0.019 | 0.353 | |
| *Alistipes* | | rs2290844 | C | T | 0.113 | 0.081 | 0.019 | 23.4 | 9.10E-06 | 0.001 | 0.028 | 0.987 | |
| *Alistipes* | | rs2450745 | A | C | 0.083 | -0.081 | 0.018 | 17.3 | 7.12E-06 | 0.082 | 0.035 | 0.018 | |
| *Alistipes* | | rs2875322 | T | C | 0.207 | -0.058 | 0.013 | 19.4 | 8.78E-06 | -0.025 | 0.023 | 0.292 | |
| *Alistipes* | | rs34417064 | A | G | 0.512 | -0.048 | 0.011 | 20.4 | 7.01E-06 | 0.007 | 0.018 | 0.695 | |
| *Alistipes* | | rs4810359 | A | G | 0.147 | -0.065 | 0.015 | 18.6 | 7.50E-06 | 0.000 | 0.027 | 0.994 | |
| *Alistipes* | | rs62576416 | T | C | 0.398 | 0.049 | 0.011 | 20.4 | 7.50E-06 | -0.015 | 0.018 | 0.403 | |
| *Alistipes* | | rs7129639 | C | A | 0.402 | -0.052 | 0.011 | 23.3 | 1.78E-06 | -0.024 | 0.019 | 0.200 | |
| *Alistipes* | | rs8130320 | A | G | 0.482 | -0.049 | 0.011 | 21.1 | 4.84E-06 | -0.001 | 0.018 | 0.950 | |
| *Allisonella* | | *rs1901739* | T | G | 0.495 | 0.116 | 0.025 | 21.7 | 3.59E-06 | 0.013 | 0.018 | 0.459 | |
| *Allisonella* | | *rs35110698* | T | C | 0.149 | -0.146 | 0.032 | 17.5 | 5.72E-06 | 0.026 | 0.025 | 0.300 | |
| *Allisonella* | | *rs35778461* | C | T | 0.192 | 0.147 | 0.030 | 21.6 | 1.21E-06 | -0.021 | 0.021 | 0.319 | |
| *Allisonella* | | *rs594561* | C | T | 0.441 | 0.112 | 0.025 | 20.1 | 9.41E-06 | 0.048 | 0.018 | 0.006 | |
| *Allisonella* | | *rs602075* | A | G | 0.231 | 0.169 | 0.030 | 32.9 | 3.57E-08 | 0.016 | 0.020 | 0.435 | |
| *Allisonella* | | *rs6742198* | G | A | 0.196 | 0.149 | 0.032 | 22.4 | 3.35E-06 | -0.051 | 0.021 | 0.013 | |
| *Allisonella* | | *rs76904847* | G | A | 0.190 | 0.149 | 0.033 | 21.7 | 6.09E-06 | -0.021 | 0.023 | 0.372 | |
| *Allisonella* | | rs7898615 | T | G | 0.115 | 0.168 | 0.037 | 18.6 | 8.87E-06 | -0.028 | 0.026 | 0.276 | |
| *Alloprevotella* | | rs12675596 | G | T | 0.245 | 0.146 | 0.029 | 23.8 | 9.64E-07 | -0.038 | 0.021 | 0.077 | |
| *Alloprevotella* | | rs2154444 | T | G | 0.223 | 0.138 | 0.031 | 20.0 | 8.37E-06 | -0.013 | 0.020 | 0.521 | |
| *Alloprevotella* | | rs34619204 | G | A | 0.200 | -0.156 | 0.034 | 23.5 | 8.84E-06 | 0.024 | 0.023 | 0.304 | |
| *Alloprevotella* | | rs4364940 | A | G | 0.279 | 0.126 | 0.028 | 19.5 | 8.58E-06 | 0.003 | 0.019 | 0.882 | |
| *Alloprevotella* | | rs4680035 | A | G | 0.546 | -0.120 | 0.026 | 21.5 | 4.99E-06 | -0.033 | 0.018 | 0.068 | |
| *Alloprevotella* | | rs58212166 | A | G | 0.133 | -0.162 | 0.036 | 18.2 | 7.94E-06 | -0.030 | 0.023 | 0.180 | |
| *Anaerofilum* | | rs10794359 | T | C | 0.487 | -0.095 | 0.020 | 22.8 | 2.23E-06 | -0.016 | 0.018 | 0.381 | |
| *Anaerofilum* | | rs1563175 | A | C | 0.444 | 0.092 | 0.020 | 21.4 | 5.54E-06 | -0.024 | 0.018 | 0.179 | |
| *Anaerofilum* | | rs17012738 | T | G | 0.408 | 0.090 | 0.020 | 20.0 | 7.24E-06 | -0.001 | 0.018 | 0.938 | |
| *Anaerofilum* | | rs17096874 | C | T | 0.181 | -0.126 | 0.027 | 24.0 | 2.86E-06 | -0.041 | 0.022 | 0.057 | |
| *Anaerofilum* | | rs356049 | G | A | 0.186 | 0.133 | 0.029 | 26.7 | 6.56E-06 | 0.014 | 0.035 | 0.690 | |
| *Anaerofilum* | | rs4244069 | G | A | 0.113 | -0.147 | 0.033 | 21.9 | 9.81E-06 | 0.019 | 0.027 | 0.480 | |
| *Anaerofilum* | | rs4506496 | G | A | 0.337 | 0.103 | 0.021 | 23.5 | 1.49E-06 | -0.024 | 0.019 | 0.214 | |
| *Anaerofilum* | | rs712981 | A | C | 0.419 | 0.101 | 0.020 | 25.1 | 6.83E-07 | 0.001 | 0.018 | 0.970 | |
| *Anaerofilum* | | rs79598899 | C | T | 0.083 | 0.183 | 0.036 | 23.9 | 3.75E-07 | -0.016 | 0.042 | 0.708 | |
| *Anaerofilum* | | rs816292 | T | C | 0.243 | -0.113 | 0.022 | 23.6 | 2.64E-07 | -0.016 | 0.019 | 0.413 | |
| *Anaerofilum* | | rs9299345 | T | C | 0.117 | -0.136 | 0.030 | 19.5 | 8.04E-06 | 0.033 | 0.029 | 0.259 | |
| *Anaerostipes* | | rs10502061 | A | G | 0.094 | -0.007 | 0.019 | 20.1 | 7.94E-06 | -0.007 | 0.028 | 0.788 | |
| *Anaerostipes* | | rs2014785 | T | C | 0.389 | -0.024 | 0.011 | 20.8 | 4.68E-06 | -0.024 | 0.018 | 0.173 | |
| *Anaerostipes* | | rs2396460 | T | C | 0.439 | -0.003 | 0.011 | 21.9 | 2.91E-06 | -0.003 | 0.018 | 0.874 | |
| *Anaerostipes* | | rs2804244 | A | G | 0.455 | 0.008 | 0.011 | 23.7 | 2.04E-06 | 0.008 | 0.018 | 0.663 | |
| *Anaerostipes* | | rs3900776 | G | A | 0.071 | -0.089 | 0.024 | 23.4 | 2.75E-06 | -0.089 | 0.053 | 0.091 | |
| *Anaerostipes* | | rs60983350 | G | A | 0.435 | -0.061 | 0.012 | 23.5 | 4.42E-06 | -0.061 | 0.019 | 0.001 | |
| *Anaerostipes* | | rs62157625 | T | C | 0.113 | -0.007 | 0.019 | 24.0 | 1.45E-06 | -0.007 | 0.027 | 0.805 | |
| *Anaerostipes* | | rs62215703 | G | A | 0.220 | 0.019 | 0.014 | 22.2 | 1.98E-06 | 0.019 | 0.021 | 0.374 | |
| *Anaerostipes* | | rs6474958 | A | G | 0.403 | 0.007 | 0.011 | 20.4 | 6.74E-06 | 0.007 | 0.019 | 0.725 | |
| *Anaerostipes* | | rs6726833 | C | A | 0.094 | 0.034 | 0.019 | 22.3 | 3.32E-06 | 0.034 | 0.033 | 0.293 | |
| *Anaerostipes* | | rs6854026 | T | C | 0.512 | 0.009 | 0.011 | 21.9 | 3.20E-06 | 0.009 | 0.018 | 0.595 | |
| *Anaerostipes* | | rs7193624 | C | T | 0.149 | -0.038 | 0.015 | 24.2 | 5.35E-07 | -0.038 | 0.032 | 0.228 | |
| *Anaerostipes* | | rs78735375 | A | C | 0.053 | 0.065 | 0.031 | 19.6 | 5.33E-06 | 0.065 | 0.045 | 0.146 | |
| *Anaerotruncus* | | rs10150232 | A | G | 0.254 | 0.057 | 0.012 | 19.6 | 6.68E-06 | 0.010 | 0.022 | 0.663 | |
| *Anaerotruncus* | | rs11018566 | A | G | 0.052 | -0.156 | 0.037 | 15.1 | 6.14E-06 | -0.036 | 0.039 | 0.356 | |
| *Anaerotruncus* | | rs115414803 | A | C | 0.059 | -0.144 | 0.032 | 21.4 | 6.83E-06 | -0.017 | 0.037 | 0.649 | |
| *Anaerotruncus* | | rs1272208 | G | T | 0.280 | -0.061 | 0.013 | 24.4 | 4.28E-06 | -0.006 | 0.021 | 0.773 | |
| *Anaerotruncus* | | rs1431492 | C | T | 0.183 | -0.065 | 0.015 | 21.3 | 7.36E-06 | -0.004 | 0.024 | 0.885 | |
| *Anaerotruncus* | | rs17734739 | T | C | 0.190 | 0.066 | 0.015 | 21.4 | 7.43E-06 | 0.014 | 0.025 | 0.567 | |
| *Anaerotruncus* | | rs34449434 | A | C | 0.456 | -0.050 | 0.011 | 19.8 | 9.85E-06 | -0.014 | 0.019 | 0.443 | |
| *Anaerotruncus* | | rs4669806 | G | T | 0.250 | 0.058 | 0.012 | 20.6 | 2.42E-06 | 0.016 | 0.021 | 0.462 | |
| *Anaerotruncus* | | rs6494922 | A | G | 0.073 | 0.090 | 0.020 | 17.7 | 6.62E-06 | -0.077 | 0.039 | 0.050 | |
| *Anaerotruncus* | | rs6563550 | T | C | 0.079 | 0.088 | 0.018 | 18.5 | 2.35E-07 | 0.003 | 0.033 | 0.918 | |
| *Anaerotruncus* | | rs7155595 | C | A | 0.309 | 0.054 | 0.012 | 20.6 | 7.55E-06 | 0.057 | 0.019 | 0.003 | |
| *Anaerotruncus* | | rs8005030 | C | T | 0.302 | 0.055 | 0.012 | 21.5 | 2.28E-06 | 0.019 | 0.019 | 0.320 | |
| *Anaerotruncus* | | rs9347879 | T | C | 0.460 | 0.051 | 0.011 | 21.1 | 4.22E-06 | 0.030 | 0.018 | 0.084 | |
| *Bacteroides* | | rs11585893 | A | G | 0.148 | -0.074 | 0.015 | 24.7 | 1.80E-06 | -0.007 | 0.021 | 0.732 | |
| *Bacteroides* | | rs13207588 | A | G | 0.233 | -0.059 | 0.013 | 22.3 | 7.49E-06 | 0.051 | 0.022 | 0.022 | |
| *Bacteroides* | | rs1340391 | T | C | 0.192 | -0.059 | 0.013 | 19.4 | 6.73E-06 | 0.000 | 0.026 | 0.998 | |
| *Bacteroides* | | rs17619981 | T | G | 0.066 | 0.088 | 0.019 | 17.0 | 2.69E-06 | 0.001 | 0.026 | 0.966 | |
| *Bacteroides* | | rs2023437 | T | C | 0.124 | -0.078 | 0.017 | 23.8 | 5.02E-06 | 0.017 | 0.027 | 0.529 | |
| *Bacteroides* | | rs66474973 | G | T | 0.115 | 0.081 | 0.016 | 24.0 | 6.81E-07 | 0.038 | 0.029 | 0.184 | |
| *Bacteroides* | | rs66710942 | C | T | 0.416 | 0.049 | 0.011 | 20.6 | 5.86E-06 | 0.014 | 0.018 | 0.413 | |
| *Bacteroides* | | rs6795673 | C | T | 0.412 | 0.054 | 0.011 | 25.1 | 3.38E-07 | -0.036 | 0.018 | 0.041 | |
| *Bacteroides* | | rs9507307 | C | T | 0.190 | 0.060 | 0.013 | 20.1 | 2.13E-06 | -0.019 | 0.021 | 0.353 | |
| *Barnesiella* | | rs11155559 | T | C | 0.095 | 0.096 | 0.021 | 21.1 | 8.92E-06 | -0.038 | 0.030 | 0.206 | |
| *Barnesiella* | | rs113258194 | A | G | 0.102 | 0.099 | 0.021 | 24.1 | 7.31E-06 | -0.038 | 0.030 | 0.202 | |
| *Barnesiella* | | rs12909713 | C | T | 0.469 | -0.055 | 0.012 | 20.2 | 4.95E-06 | -0.019 | 0.018 | 0.287 | |
| *Barnesiella* | | rs13242616 | T | C | 0.370 | -0.058 | 0.012 | 21.3 | 2.29E-06 | -0.019 | 0.019 | 0.309 | |
| *Barnesiella* | | rs199035 | G | A | 0.509 | 0.056 | 0.012 | 21.0 | 3.00E-06 | -0.019 | 0.018 | 0.294 | |
| *Barnesiella* | | rs2276875 | A | G | 0.255 | -0.070 | 0.014 | 24.7 | 4.65E-07 | -0.010 | 0.020 | 0.617 | |
| *Barnesiella* | | rs2428166 | G | A | 0.049 | -0.166 | 0.034 | 34.2 | 8.51E-07 | 0.030 | 0.074 | 0.680 | |
| *Barnesiella* | | rs35177866 | A | G | 0.124 | 0.092 | 0.019 | 24.5 | 2.95E-06 | -0.040 | 0.034 | 0.234 | |
| *Barnesiella* | | rs62251337 | A | G | 0.158 | -0.069 | 0.015 | 17.0 | 4.24E-06 | -0.036 | 0.025 | 0.154 | |
| *Barnesiella* | | rs72684847 | T | C | 0.066 | -0.114 | 0.025 | 21.5 | 6.76E-06 | 0.026 | 0.033 | 0.431 | |
| *Barnesiella* | | rs76181748 | C | T | 0.144 | -0.078 | 0.017 | 20.0 | 6.78E-06 | 0.025 | 0.021 | 0.231 | |
| *Barnesiella* | | rs77455852 | T | G | 0.121 | -0.089 | 0.020 | 22.7 | 3.16E-06 | -0.025 | 0.025 | 0.329 | |
| *Barnesiella* | | rs79795328 | A | G | 0.125 | -0.082 | 0.018 | 19.7 | 4.23E-06 | 0.038 | 0.025 | 0.134 | |
| *Bifidobacterium* | | rs12022129 | G | A | 0.221 | 0.062 | 0.014 | 19.5 | 8.00E-06 | 0.033 | 0.020 | 0.089 | |
| *Bifidobacterium* | | rs182549 | C | T | 0.491 | 0.120 | 0.013 | 106.6 | 1.28E-20 | -0.044 | 0.018 | 0.013 | |
| *Bifidobacterium* | | rs2491158 | G | A | 0.182 | 0.071 | 0.016 | 22.4 | 8.05E-06 | 0.054 | 0.026 | 0.037 | |
| *Bifidobacterium* | | rs2686790 | T | C | 0.115 | 0.071 | 0.016 | 15.1 | 7.50E-06 | 0.003 | 0.025 | 0.912 | |
| *Bifidobacterium* | | rs540489 | T | G | 0.234 | -0.064 | 0.014 | 21.5 | 5.19E-06 | 0.001 | 0.023 | 0.976 | |
| *Bifidobacterium* | | rs55888705 | A | G | 0.387 | 0.055 | 0.012 | 20.9 | 6.67E-06 | 0.030 | 0.020 | 0.119 | |
| *Bifidobacterium* | | rs56108664 | T | C | 0.185 | 0.073 | 0.016 | 23.8 | 2.44E-06 | 0.020 | 0.026 | 0.446 | |
| *Bifidobacterium* | | rs5746486 | T | C | 0.354 | -0.054 | 0.012 | 19.5 | 9.00E-06 | 0.027 | 0.018 | 0.139 | |
| *Bifidobacterium* | | rs62181700 | G | A | 0.281 | -0.062 | 0.013 | 23.3 | 2.17E-06 | 0.024 | 0.020 | 0.236 | |
| *Bifidobacterium* | | rs7322849 | T | C | 0.096 | 0.112 | 0.020 | 32.6 | 1.08E-08 | 0.003 | 0.031 | 0.920 | |
| *Bifidobacterium* | | rs73797465 | T | G | 0.087 | -0.095 | 0.021 | 21.3 | 4.38E-06 | 0.002 | 0.028 | 0.946 | |
| *Bifidobacterium* | | rs75344046 | C | T | 0.050 | 0.232 | 0.051 | 75.7 | 4.86E-06 | -0.041 | 0.042 | 0.325 | |
| *Bifidobacterium* | | rs857444 | C | T | 0.361 | 0.056 | 0.012 | 21.3 | 3.57E-06 | 0.005 | 0.018 | 0.780 | |
| *Bilophila* | | rs11069458 | T | C | 0.190 | -0.068 | 0.016 | 18.4 | 7.72E-06 | -0.044 | 0.023 | 0.054 | |
| *Bilophila* | | rs1241171 | G | A | 0.216 | -0.069 | 0.015 | 20.9 | 4.24E-06 | -0.018 | 0.025 | 0.480 | |
| *Bilophila* | | rs1571225 | C | T | 0.149 | 0.083 | 0.017 | 22.3 | 1.12E-06 | -0.024 | 0.024 | 0.313 | |
| *Bilophila* | | rs1969927 | G | A | 0.380 | 0.056 | 0.013 | 19.3 | 9.07E-06 | -0.009 | 0.019 | 0.649 | |
| *Bilophila* | | rs2728491 | G | T | 0.310 | -0.063 | 0.014 | 21.7 | 6.33E-06 | 0.014 | 0.021 | 0.509 | |
| *Bilophila* | | rs3827020 | C | T | 0.190 | 0.077 | 0.016 | 23.3 | 1.79E-06 | 0.024 | 0.022 | 0.274 | |
| *Bilophila* | | rs4798126 | G | A | 0.150 | 0.073 | 0.017 | 17.6 | 7.15E-06 | -0.030 | 0.022 | 0.174 | |
| *Bilophila* | | rs542415 | T | C | 0.340 | -0.061 | 0.013 | 21.8 | 4.71E-06 | 0.001 | 0.018 | 0.971 | |
| *Bilophila* | | rs60178956 | G | A | 0.235 | -0.062 | 0.014 | 18.0 | 8.06E-06 | -0.017 | 0.021 | 0.423 | |
| *Bilophila* | | rs6793291 | C | A | 0.092 | 0.113 | 0.024 | 27.5 | 3.11E-06 | 0.001 | 0.040 | 0.978 | |
| *Bilophila* | | rs72676854 | T | C | 0.066 | 0.123 | 0.027 | 24.0 | 5.62E-06 | -0.016 | 0.039 | 0.689 | |
| *Bilophila* | | rs7802841 | C | A | 0.351 | 0.067 | 0.014 | 26.3 | 1.77E-06 | 0.021 | 0.019 | 0.283 | |
| *Bilophila* | | rs9899990 | A | G | 0.066 | -0.103 | 0.023 | 16.6 | 9.07E-06 | -0.048 | 0.032 | 0.130 | |
| *Blautia* | | rs11149971 | C | T | 0.054 | 0.118 | 0.023 | 22.9 | 1.04E-06 | 0.075 | 0.038 | 0.052 | |
| *Blautia* | | rs113271346 | C | T | 0.118 | 0.078 | 0.017 | 20.8 | 6.85E-06 | -0.039 | 0.032 | 0.220 | |
| *Blautia* | | rs115043014 | G | A | 0.044 | -0.207 | 0.044 | 58.2 | 5.19E-06 | -0.054 | 0.068 | 0.426 | |
| *Blautia* | | rs117001700 | T | C | 0.044 | 0.196 | 0.044 | 52.6 | 8.84E-06 | 0.029 | 0.074 | 0.700 | |
| *Blautia* | | rs12453000 | C | T | 0.220 | 0.063 | 0.013 | 21.8 | 1.26E-06 | 0.011 | 0.026 | 0.681 | |
| *Blautia* | | rs16892041 | T | C | 0.171 | -0.062 | 0.014 | 17.9 | 8.82E-06 | 0.002 | 0.022 | 0.926 | |
| *Blautia* | | rs2788271 | T | G | 0.165 | -0.058 | 0.013 | 14.9 | 7.16E-06 | -0.037 | 0.023 | 0.114 | |
| *Blautia* | | rs3005511 | A | G | 0.336 | 0.050 | 0.011 | 18.2 | 6.19E-06 | -0.065 | 0.019 | 0.001 | |
| *Blautia* | | rs4926264 | T | C | 0.079 | 0.083 | 0.018 | 16.1 | 5.10E-06 | 0.021 | 0.029 | 0.460 | |
| *Blautia* | | rs67794373 | C | T | 0.214 | 0.060 | 0.012 | 19.8 | 1.00E-06 | 0.013 | 0.021 | 0.533 | |
| *Blautia* | | rs682885 | A | G | 0.417 | -0.049 | 0.011 | 19.3 | 4.49E-06 | 0.023 | 0.019 | 0.229 | |
| *Blautia* | | rs72973581 | A | G | 0.055 | 0.125 | 0.027 | 26.4 | 1.74E-06 | 0.026 | 0.038 | 0.492 | |
| *Blautia* | | rs7860714 | A | G | 0.334 | -0.050 | 0.011 | 18.3 | 4.09E-06 | -0.005 | 0.019 | 0.778 | |
| *Butyricicoccus* | | rs10084203 | A | G | 0.242 | 0.055 | 0.012 | 19.0 | 8.59E-06 | 0.012 | 0.026 | 0.642 | |
| *Butyricicoccus* | | rs12034718 | A | G | 0.165 | 0.070 | 0.016 | 23.2 | 9.58E-06 | -0.022 | 0.021 | 0.308 | |
| *Butyricicoccus* | | rs12585793 | T | C | 0.053 | -0.262 | 0.056 | 118.4 | 5.79E-06 | 0.020 | 0.052 | 0.700 | |
| *Butyricicoccus* | | rs2017189 | G | T | 0.422 | -0.051 | 0.011 | 21.5 | 3.87E-06 | -0.024 | 0.018 | 0.176 | |
| *Butyricicoccus* | | rs4962426 | G | T | 0.198 | 0.061 | 0.014 | 20.5 | 7.38E-06 | -0.050 | 0.022 | 0.025 | |
| *Butyricicoccus* | | rs56221232 | T | C | 0.108 | 0.083 | 0.017 | 22.7 | 7.62E-07 | 0.023 | 0.029 | 0.437 | |
| *Butyricicoccus* | | rs62478070 | T | G | 0.044 | 0.224 | 0.049 | 72.2 | 5.94E-06 | 0.069 | 0.056 | 0.222 | |
| *Butyricicoccus* | | rs7322368 | T | C | 0.085 | 0.082 | 0.018 | 17.7 | 5.52E-06 | -0.048 | 0.031 | 0.115 | |
| *Butyricimonas* | | rs11228830 | A | G | 0.071 | 0.135 | 0.030 | 21.6 | 6.55E-06 | 0.038 | 0.033 | 0.244 | |
| *Butyricimonas* | | rs113054641 | G | A | 0.085 | -0.145 | 0.027 | 29.2 | 1.74E-07 | 0.056 | 0.042 | 0.181 | |
| *Butyricimonas* | | rs12304031 | G | A | 0.129 | -0.086 | 0.020 | 15.1 | 6.70E-06 | 0.011 | 0.027 | 0.677 | |
| *Butyricimonas* | | rs12458763 | A | C | 0.072 | 0.122 | 0.027 | 17.8 | 6.37E-06 | 0.028 | 0.042 | 0.505 | |
| *Butyricimonas* | | rs1862649 | G | A | 0.092 | 0.113 | 0.025 | 19.1 | 4.76E-06 | -0.016 | 0.035 | 0.644 | |
| *Butyricimonas* | | rs2114713 | G | T | 0.451 | 0.063 | 0.014 | 17.5 | 6.88E-06 | -0.025 | 0.018 | 0.157 | |
| *Butyricimonas* | | rs62130338 | G | A | 0.262 | -0.073 | 0.016 | 18.7 | 3.90E-06 | -0.037 | 0.019 | 0.048 | |
| *Butyricimonas* | | rs62390301 | T | C | 0.183 | -0.087 | 0.017 | 20.5 | 7.42E-07 | 0.012 | 0.022 | 0.575 | |
| *Butyricimonas* | | rs7083431 | A | C | 0.355 | 0.070 | 0.014 | 20.4 | 8.85E-07 | 0.032 | 0.020 | 0.106 | |
| *Butyricimonas* | | rs71428626 | G | T | 0.073 | -0.133 | 0.029 | 21.5 | 4.80E-06 | -0.012 | 0.049 | 0.815 | |
| *Butyricimonas* | | rs72814525 | A | G | 0.323 | 0.066 | 0.015 | 17.3 | 8.25E-06 | 0.011 | 0.021 | 0.580 | |
| *Butyricimonas* | | rs78453362 | A | G | 0.058 | -0.149 | 0.033 | 21.9 | 4.06E-06 | -0.014 | 0.056 | 0.805 | |
| *Butyricimonas* | | rs9657374 | C | T | 0.302 | 0.068 | 0.015 | 17.6 | 4.50E-06 | 0.010 | 0.019 | 0.593 | |
| *Butyrivibrio* | | rs1007475 | G | T | 0.294 | 0.118 | 0.026 | 20.3 | 7.92E-06 | -0.001 | 0.020 | 0.963 | |
| *Butyrivibrio* | | rs11761679 | T | C | 0.156 | 0.155 | 0.032 | 22.1 | 2.20E-06 | 0.026 | 0.025 | 0.313 | |
| *Butyrivibrio* | | rs142855850 | A | G | 0.101 | 0.205 | 0.046 | 26.9 | 6.86E-06 | 0.027 | 0.030 | 0.362 | |
| *Butyrivibrio* | | rs16934069 | T | C | 0.199 | -0.134 | 0.030 | 20.0 | 8.86E-06 | -0.026 | 0.023 | 0.260 | |
| *Butyrivibrio* | | rs16941336 | C | T | 0.264 | 0.127 | 0.027 | 22.1 | 1.53E-06 | -0.015 | 0.021 | 0.468 | |
| *Butyrivibrio* | | rs17163238 | G | A | 0.200 | 0.141 | 0.031 | 22.3 | 5.51E-06 | 0.039 | 0.022 | 0.080 | |
| *Butyrivibrio* | | rs4537857 | T | C | 0.292 | -0.125 | 0.026 | 22.5 | 1.80E-06 | -0.018 | 0.019 | 0.338 | |
| *Butyrivibrio* | | rs486484 | A | G | 0.471 | -0.108 | 0.024 | 20.5 | 6.61E-06 | -0.001 | 0.018 | 0.943 | |
| *Butyrivibrio* | | rs4928024 | A | G | 0.098 | -0.175 | 0.039 | 19.0 | 8.19E-06 | 0.016 | 0.023 | 0.487 | |
| *Butyrivibrio* | | rs72723662 | C | T | 0.100 | 0.224 | 0.045 | 31.9 | 7.86E-07 | 0.036 | 0.026 | 0.161 | |
| *Butyrivibrio* | | rs74622183 | A | G | 0.088 | -0.201 | 0.043 | 22.6 | 2.46E-06 | -0.023 | 0.031 | 0.451 | |
| *Butyrivibrio* | | rs77356209 | T | C | 0.048 | 0.217 | 0.048 | 14.9 | 6.66E-06 | 0.015 | 0.042 | 0.730 | |
| *Butyrivibrio* | | rs7752361 | A | G | 0.504 | -0.119 | 0.024 | 24.9 | 7.69E-07 | 0.018 | 0.018 | 0.312 | |
| *Butyrivibrio* | | rs7763512 | G | A | 0.383 | 0.120 | 0.025 | 23.8 | 3.11E-06 | -0.011 | 0.018 | 0.525 | |
| *Butyrivibrio* | | rs9349693 | A | G | 0.311 | 0.118 | 0.026 | 20.9 | 5.55E-06 | -0.007 | 0.019 | 0.712 | |
| *Candidatus Soleaferrea* | | rs10090365 | A | G | 0.559 | -0.083 | 0.018 | 21.2 | 4.17E-06 | 0.035 | 0.018 | 0.050 | |
| *Candidatus Soleaferrea* | | rs10108780 | A | G | 0.267 | -0.093 | 0.020 | 20.8 | 3.64E-06 | 0.021 | 0.020 | 0.305 | |
| *Candidatus Soleaferrea* | | rs10809135 | T | C | 0.491 | 0.083 | 0.018 | 21.5 | 5.47E-06 | 0.006 | 0.018 | 0.727 | |
| *Candidatus Soleaferrea* | | rs36155147 | C | T | 0.362 | 0.105 | 0.024 | 31.4 | 5.41E-06 | -0.012 | 0.019 | 0.519 | |
| *Candidatus Soleaferrea* | | rs4294381 | T | C | 0.180 | 0.112 | 0.023 | 22.9 | 1.37E-06 | -0.024 | 0.024 | 0.328 | |
| *Candidatus Soleaferrea* | | rs4678258 | T | C | 0.276 | 0.099 | 0.022 | 24.0 | 5.53E-06 | 0.004 | 0.021 | 0.843 | |
| *Candidatus Soleaferrea* | | rs6489992 | A | G | 0.385 | -0.084 | 0.019 | 20.6 | 7.89E-06 | -0.003 | 0.018 | 0.888 | |
| *Candidatus Soleaferrea* | | rs6494306 | A | G | 0.197 | -0.097 | 0.021 | 18.3 | 5.80E-06 | -0.007 | 0.019 | 0.701 | |
| *Candidatus Soleaferrea* | | rs7400877 | T | C | 0.251 | -0.095 | 0.021 | 20.9 | 9.29E-06 | 0.040 | 0.021 | 0.061 | |
| *Candidatus Soleaferrea* | | rs9973954 | A | G | 0.306 | 0.089 | 0.020 | 20.8 | 5.95E-06 | 0.008 | 0.018 | 0.650 | |
| *Catenibacterium* | | rs12404911 | C | T | 0.170 | 0.141 | 0.030 | 18.2 | 2.80E-06 | -0.015 | 0.023 | 0.508 | |
| *Catenibacterium* | | rs212393 | G | A | 0.310 | -0.135 | 0.029 | 25.5 | 3.62E-06 | 0.008 | 0.022 | 0.699 | |
| *Catenibacterium* | | rs73128290 | A | G | 0.263 | 0.130 | 0.028 | 21.3 | 4.29E-06 | -0.026 | 0.019 | 0.167 | |
| *Catenibacterium* | | rs7742829 | C | T | 0.420 | 0.114 | 0.025 | 20.7 | 5.61E-06 | -0.007 | 0.018 | 0.687 | |
| *Christensenellaceae (R7 group)* | | rs10461257 | A | G | 0.285 | -0.055 | 0.012 | 19.9 | 6.51E-06 | -0.003 | 0.019 | 0.887 | |
| *Christensenellaceae (R7 group)* | | rs17081797 | A | G | 0.069 | -0.090 | 0.020 | 16.7 | 3.34E-06 | 0.050 | 0.036 | 0.158 | |
| *Christensenellaceae (R7 group)* | | rs60954665 | T | G | 0.510 | 0.050 | 0.011 | 19.9 | 7.13E-06 | -0.031 | 0.018 | 0.078 | |
| *Christensenellaceae (R7 group)* | | rs62132810 | A | G | 0.108 | -0.083 | 0.018 | 21.2 | 5.67E-06 | -0.029 | 0.025 | 0.251 | |
| *Christensenellaceae (R7 group)* | | rs62190261 | A | C | 0.091 | 0.096 | 0.021 | 24.2 | 8.74E-06 | -0.003 | 0.031 | 0.916 | |
| *Christensenellaceae (R7 group)* | | rs62467127 | C | T | 0.045 | 0.114 | 0.025 | 17.8 | 3.25E-06 | 0.018 | 0.056 | 0.748 | |
| *Christensenellaceae (R7 group)* | | rs73952017 | C | T | 0.088 | -0.086 | 0.019 | 19.0 | 8.46E-06 | -0.016 | 0.029 | 0.590 | |
| *Christensenellaceae (R7 group)* | | rs78521377 | C | T | 0.053 | 0.125 | 0.027 | 25.0 | 5.61E-06 | 0.042 | 0.051 | 0.414 | |
| *Christensenellaceae (R7 group)* | | rs79150079 | C | A | 0.072 | 0.122 | 0.027 | 31.5 | 9.42E-06 | -0.006 | 0.035 | 0.858 | |
| *Christensenellaceae (R7 group)* | | rs892686 | A | G | 0.451 | 0.051 | 0.011 | 21.0 | 3.97E-06 | -0.008 | 0.018 | 0.654 | |
| *Clostridium (innocuum group)* | | rs10074000 | T | C | 0.414 | -0.103 | 0.023 | 20.8 | 7.00E-06 | 0.016 | 0.018 | 0.384 | |
| *Clostridium (innocuum group)* | | rs10506058 | A | G | 0.397 | 0.100 | 0.022 | 19.3 | 8.92E-06 | -0.017 | 0.018 | 0.333 | |
| *Clostridium (innocuum group)* | | rs1942371 | G | A | 0.130 | -0.158 | 0.034 | 23.0 | 4.06E-06 | 0.000 | 0.027 | 0.993 | |
| *Clostridium (innocuum group)* | | rs40656 | C | T | 0.189 | 0.143 | 0.031 | 25.4 | 8.62E-06 | -0.037 | 0.022 | 0.093 | |
| *Clostridium (innocuum group)* | | rs4869133 | G | A | 0.089 | -0.181 | 0.041 | 21.4 | 7.24E-06 | 0.022 | 0.023 | 0.347 | |
| *Clostridium (innocuum group)* | | rs61267978 | T | C | 0.124 | 0.147 | 0.032 | 19.1 | 5.59E-06 | -0.004 | 0.027 | 0.885 | |
| *Clostridium (innocuum group)* | | rs6577484 | G | A | 0.148 | 0.160 | 0.036 | 26.4 | 8.41E-06 | -0.017 | 0.029 | 0.559 | |
| *Clostridium (innocuum group)* | | rs6890185 | T | C | 0.311 | 0.113 | 0.023 | 22.4 | 1.12E-06 | 0.004 | 0.019 | 0.845 | |
| *Clostridium (innocuum group)* | | rs77845139 | A | G | 0.295 | -0.115 | 0.026 | 22.4 | 8.41E-06 | -0.026 | 0.021 | 0.213 | |
| *Clostridium sensustricto 1* | | rs11264403 | G | A | 0.040 | -0.139 | 0.033 | 10.8 | 7.76E-06 | 0.016 | 0.033 | 0.635 | |
| *Clostridium sensustricto 1* | | rs115807074 | A | G | 0.050 | -0.227 | 0.049 | 35.7 | 4.32E-06 | -0.158 | 0.086 | 0.065 | |
| *Clostridium sensustricto 1* | | rs116847295 | C | T | 0.076 | 0.110 | 0.025 | 12.3 | 4.58E-06 | -0.011 | 0.026 | 0.681 | |
| *Clostridium sensustricto 1* | | rs12341505 | G | A | 0.111 | 0.081 | 0.018 | 20.3 | 4.82E-06 | 0.004 | 0.030 | 0.903 | |
| *Clostridium sensustricto 1* | | rs2795528 | G | A | 0.049 | -0.184 | 0.039 | 23.0 | 2.72E-06 | 0.008 | 0.038 | 0.846 | |
| *Clostridium sensustricto 1* | | rs2817172 | C | T | 0.413 | 0.058 | 0.012 | 12.0 | 2.77E-06 | -0.009 | 0.018 | 0.635 | |
| *Clostridium sensustricto 1* | | rs550843 | T | C | 0.149 | -0.078 | 0.017 | 11.4 | 2.05E-06 | -0.020 | 0.020 | 0.308 | |
| *Collinsella* | | rs10890671 | T | C | 0.527 | -0.054 | 0.012 | 20.7 | 6.52E-06 | -0.013 | 0.018 | 0.468 | |
| *Collinsella* | | rs11597285 | G | T | 0.425 | -0.054 | 0.012 | 20.3 | 9.38E-06 | -0.004 | 0.018 | 0.839 | |
| *Collinsella* | | rs1496626 | T | C | 0.154 | -0.072 | 0.016 | 19.5 | 6.78E-06 | -0.025 | 0.026 | 0.335 | |
| *Collinsella* | | rs149807560 | C | A | 0.090 | -0.104 | 0.024 | 25.4 | 7.10E-06 | 0.032 | 0.035 | 0.359 | |
| *Collinsella* | | rs2103510 | G | A | 0.131 | 0.079 | 0.017 | 20.2 | 2.42E-06 | 0.033 | 0.027 | 0.234 | |
| *Collinsella* | | rs62448871 | C | A | 0.461 | -0.054 | 0.012 | 20.8 | 6.78E-06 | 0.001 | 0.018 | 0.952 | |
| *Collinsella* | | rs73052258 | G | A | 0.096 | 0.093 | 0.020 | 21.6 | 1.72E-06 | -0.008 | 0.033 | 0.812 | |
| *Collinsella* | | rs75672793 | A | G | 0.071 | -0.109 | 0.024 | 22.3 | 6.14E-06 | -0.036 | 0.042 | 0.390 | |
| *Collinsella* | | rs9541268 | C | A | 0.096 | 0.096 | 0.020 | 23.0 | 8.79E-07 | -0.034 | 0.031 | 0.269 | |
| *Coprobacter* | | rs11532348 | C | T | 0.183 | -0.104 | 0.023 | 20.6 | 5.71E-06 | 0.007 | 0.025 | 0.784 | |
| *Coprobacter* | | rs12684609 | T | C | 0.198 | 0.101 | 0.022 | 20.6 | 6.10E-06 | 0.012 | 0.022 | 0.598 | |
| *Coprobacter* | | rs12996055 | A | C | 0.192 | 0.092 | 0.021 | 16.8 | 8.08E-06 | -0.026 | 0.020 | 0.195 | |
| *Coprobacter* | | rs143662916 | C | T | 0.058 | 0.253 | 0.054 | 44.7 | 3.07E-06 | -0.074 | 0.052 | 0.154 | |
| *Coprobacter* | | rs189356 | G | A | 0.522 | 0.078 | 0.017 | 19.4 | 6.26E-06 | 0.027 | 0.018 | 0.125 | |
| *Coprobacter* | | rs213863 | C | T | 0.309 | -0.089 | 0.019 | 21.5 | 2.35E-06 | 0.002 | 0.018 | 0.923 | |
| *Coprobacter* | | rs305411 | A | G | 0.135 | 0.129 | 0.026 | 24.9 | 1.01E-06 | -0.032 | 0.029 | 0.278 | |
| *Coprobacter* | | rs3828477 | G | T | 0.281 | -0.091 | 0.020 | 21.5 | 2.89E-06 | 0.000 | 0.018 | 0.998 | |
| *Coprobacter* | | rs72821405 | T | C | 0.089 | -0.147 | 0.032 | 22.4 | 4.76E-06 | -0.039 | 0.031 | 0.212 | |
| *Coprobacter* | | rs74919520 | G | A | 0.128 | 0.126 | 0.028 | 22.6 | 5.76E-06 | 0.016 | 0.029 | 0.572 | |
| *Coprococcus 1* | | rs1010560 | C | A | 0.296 | 0.058 | 0.012 | 23.5 | 1.96E-06 | 0.003 | 0.020 | 0.879 | |
| *Coprococcus 1* | | rs12794898 | G | T | 0.094 | 0.090 | 0.020 | 23.4 | 4.92E-06 | -0.038 | 0.027 | 0.156 | |
| *Coprococcus 1* | | rs1519491 | T | C | 0.409 | 0.050 | 0.011 | 20.2 | 8.95E-06 | -0.018 | 0.018 | 0.306 | |
| *Coprococcus 1* | | rs1576241 | A | G | 0.439 | -0.051 | 0.011 | 21.5 | 3.33E-06 | 0.028 | 0.018 | 0.121 | |
| *Coprococcus 1* | | rs1762123 | C | T | 0.072 | -0.089 | 0.020 | 17.7 | 8.01E-06 | 0.015 | 0.029 | 0.614 | |
| *Coprococcus 1* | | rs2907920 | A | G | 0.290 | 0.056 | 0.013 | 21.7 | 7.65E-06 | 0.021 | 0.020 | 0.292 | |
| *Coprococcus 1* | | rs4277593 | G | A | 0.483 | -0.059 | 0.011 | 28.7 | 1.14E-07 | -0.006 | 0.018 | 0.760 | |
| *Coprococcus 1* | | rs56405618 | A | G | 0.111 | -0.090 | 0.019 | 26.6 | 1.57E-06 | -0.012 | 0.028 | 0.673 | |
| *Coprococcus 1* | | rs73031725 | T | C | 0.061 | 0.168 | 0.036 | 53.6 | 1.98E-06 | 0.013 | 0.051 | 0.793 | |
| *Coprococcus 1* | | rs73167075 | T | C | 0.240 | 0.057 | 0.013 | 20.0 | 8.57E-06 | 0.061 | 0.022 | 0.004 | |
| *Coprococcus 1* | | rs74101919 | T | C | 0.187 | -0.072 | 0.014 | 26.3 | 1.03E-06 | 0.000 | 0.028 | 1.000 | |
| *Coprococcus 1* | | rs946513 | C | T | 0.055 | 0.206 | 0.046 | 73.6 | 8.62E-06 | 0.006 | 0.043 | 0.885 | |
| *Coprococcus 2* | | rs10070053 | A | G | 0.408 | 0.059 | 0.014 | 19.6 | 7.65E-06 | 0.001 | 0.018 | 0.962 | |
| *Coprococcus 2* | | rs12634070 | T | C | 0.193 | 0.074 | 0.016 | 19.4 | 9.95E-06 | -0.045 | 0.020 | 0.026 | |
| *Coprococcus 2* | | rs2482516 | C | T | 0.226 | 0.075 | 0.016 | 22.9 | 4.72E-06 | -0.005 | 0.021 | 0.824 | |
| *Coprococcus 2* | | rs35890118 | A | G | 0.317 | -0.067 | 0.015 | 22.0 | 8.26E-06 | -0.001 | 0.020 | 0.958 | |
| *Coprococcus 2* | | rs61823518 | A | C | 0.095 | -0.096 | 0.022 | 18.1 | 6.68E-06 | -0.017 | 0.029 | 0.556 | |
| *Coprococcus 2* | | rs6677933 | C | T | 0.242 | -0.080 | 0.016 | 27.3 | 1.19E-06 | -0.007 | 0.025 | 0.780 | |
| *Coprococcus 2* | | rs72680320 | T | C | 0.414 | -0.065 | 0.014 | 23.5 | 2.27E-06 | 0.010 | 0.019 | 0.609 | |
| *Coprococcus 2* | | rs9426473 | A | G | 0.279 | 0.073 | 0.016 | 24.5 | 6.31E-06 | -0.006 | 0.020 | 0.763 | |
| *Coprococcus3* | | rs10810043 | A | G | 0.390 | 0.052 | 0.012 | 20.0 | 9.27E-06 | 0.020 | 0.019 | 0.284 | |
| *Coprococcus3* | | rs11077359 | T | C | 0.200 | -0.065 | 0.015 | 21.1 | 9.64E-06 | 0.016 | 0.024 | 0.490 | |
| *Coprococcus3* | | rs11080344 | C | T | 0.474 | 0.052 | 0.011 | 21.1 | 4.79E-06 | 0.023 | 0.018 | 0.200 | |
| *Coprococcus3* | | rs13247359 | G | A | 0.480 | 0.051 | 0.011 | 20.8 | 7.33E-06 | -0.020 | 0.018 | 0.256 | |
| *Coprococcus3* | | rs13394391 | C | T | 0.160 | -0.071 | 0.015 | 21.4 | 2.20E-06 | -0.048 | 0.024 | 0.047 | |
| *Coprococcus3* | | rs178271 | T | C | 0.053 | 0.145 | 0.029 | 33.4 | 7.81E-07 | 0.007 | 0.069 | 0.916 | |
| *Coprococcus3* | | rs4575475 | G | A | 0.237 | 0.062 | 0.014 | 22.0 | 7.04E-06 | -0.044 | 0.021 | 0.035 | |
| *Coprococcus3* | | rs7521171 | G | A | 0.338 | -0.060 | 0.013 | 25.2 | 4.32E-06 | -0.003 | 0.019 | 0.888 | |
| *Coprococcus3* | | rs8100692 | T | C | 0.427 | 0.058 | 0.011 | 25.9 | 4.16E-07 | 0.019 | 0.018 | 0.293 | |
| *Defluviitaleaceae (UCG011)* | | rs112893842 | T | C | 0.097 | 0.114 | 0.023 | 18.7 | 1.45E-06 | 0.011 | 0.031 | 0.722 | |
| *Defluviitaleaceae (UCG011)* | | rs1582238 | T | C | 0.372 | 0.081 | 0.017 | 24.8 | 1.57E-06 | -0.012 | 0.018 | 0.524 | |
| *Defluviitaleaceae (UCG011)* | | rs2892880 | G | A | 0.260 | 0.082 | 0.018 | 21.1 | 6.83E-06 | -0.019 | 0.020 | 0.344 | |
| *Defluviitaleaceae (UCG011)* | | rs4344384 | G | T | 0.474 | 0.072 | 0.016 | 21.0 | 4.83E-06 | -0.003 | 0.018 | 0.885 | |
| *Defluviitaleaceae (UCG011)* | | rs4677103 | A | G | 0.171 | 0.098 | 0.020 | 22.2 | 9.60E-07 | -0.027 | 0.023 | 0.249 | |
| *Defluviitaleaceae (UCG011)* | | rs55658617 | T | C | 0.070 | 0.174 | 0.036 | 32.3 | 2.15E-06 | -0.054 | 0.049 | 0.271 | |
| *Defluviitaleaceae (UCG011)* | | rs72731813 | C | T | 0.082 | -0.147 | 0.029 | 26.7 | 4.33E-07 | -0.007 | 0.041 | 0.871 | |
| *Defluviitaleaceae (UCG011)* | | rs9608282 | T | G | 0.066 | 0.143 | 0.030 | 20.5 | 2.52E-06 | -0.015 | 0.049 | 0.759 | |
| *Defluviitaleaceae (UCG011)* | | rs9725395 | A | G | 0.088 | -0.138 | 0.030 | 25.1 | 3.52E-06 | -0.001 | 0.028 | 0.978 | |
| *Desulfovibrio* | | rs12031543 | T | C | 0.076 | -0.127 | 0.028 | 20.9 | 6.55E-06 | 0.041 | 0.026 | 0.109 | |
| *Desulfovibrio* | | rs13066142 | G | A | 0.093 | 0.119 | 0.025 | 22.2 | 3.79E-06 | -0.045 | 0.031 | 0.143 | |
| *Desulfovibrio* | | rs16863365 | A | G | 0.117 | 0.109 | 0.023 | 22.9 | 1.79E-06 | -0.041 | 0.042 | 0.324 | |
| *Desulfovibrio* | | rs2032031 | A | G | 0.473 | -0.065 | 0.015 | 19.8 | 9.14E-06 | 0.010 | 0.018 | 0.583 | |
| *Desulfovibrio* | | rs2590913 | G | A | 0.073 | 0.154 | 0.034 | 29.8 | 6.65E-06 | -0.029 | 0.041 | 0.471 | |
| *Desulfovibrio* | | rs2853179 | C | T | 0.249 | 0.081 | 0.017 | 22.8 | 2.42E-06 | 0.020 | 0.021 | 0.338 | |
| *Desulfovibrio* | | rs4797774 | G | A | 0.069 | 0.213 | 0.047 | 53.6 | 5.64E-06 | 0.000 | 0.044 | 0.993 | |
| *Desulfovibrio* | | rs6580353 | T | C | 0.260 | 0.077 | 0.017 | 21.2 | 4.94E-06 | -0.014 | 0.022 | 0.529 | |
| *Desulfovibrio* | | rs72647089 | T | G | 0.106 | -0.107 | 0.024 | 20.0 | 8.30E-06 | -0.021 | 0.032 | 0.510 | |
| *Desulfovibrio* | | rs7729080 | C | A | 0.289 | -0.070 | 0.016 | 18.8 | 9.96E-06 | -0.024 | 0.019 | 0.216 | |
| *Dialister* | | rs10138457 | T | C | 0.072 | -0.113 | 0.026 | 18.7 | 7.88E-06 | 0.055 | 0.030 | 0.068 | |
| *Dialister* | | rs10938938 | G | A | 0.194 | -0.077 | 0.017 | 20.5 | 7.37E-06 | -0.006 | 0.024 | 0.815 | |
| *Dialister* | | rs11071887 | T | C | 0.263 | 0.066 | 0.015 | 18.7 | 5.91E-06 | 0.011 | 0.019 | 0.545 | |
| *Dialister* | | rs11166701 | G | A | 0.516 | -0.066 | 0.013 | 23.6 | 5.51E-07 | -0.017 | 0.018 | 0.334 | |
| *Dialister* | | rs2314294 | T | C | 0.118 | 0.087 | 0.019 | 17.2 | 8.08E-06 | -0.031 | 0.026 | 0.233 | |
| *Dialister* | | rs2435610 | A | C | 0.260 | 0.065 | 0.014 | 17.7 | 5.93E-06 | -0.008 | 0.020 | 0.692 | |
| *Dialister* | | rs4747450 | C | A | 0.266 | 0.067 | 0.015 | 19.2 | 5.84E-06 | -0.016 | 0.021 | 0.446 | |
| *Dialister* | | rs4753063 | G | A | 0.441 | -0.060 | 0.013 | 19.3 | 4.86E-06 | -0.013 | 0.018 | 0.466 | |
| *Dialister* | | rs75416973 | A | G | 0.226 | 0.073 | 0.016 | 20.3 | 9.46E-06 | 0.027 | 0.021 | 0.201 | |
| *Dialister* | | rs764177 | C | A | 0.338 | -0.060 | 0.014 | 17.8 | 9.61E-06 | -0.014 | 0.018 | 0.445 | |
| *Dialister* | | rs76680460 | G | A | 0.048 | -0.161 | 0.036 | 26.0 | 8.19E-06 | 0.066 | 0.044 | 0.133 | |
| *Dorea* | | rs11150408 | T | G | 0.445 | 0.049 | 0.011 | 20.3 | 7.06E-06 | 0.001 | 0.018 | 0.945 | |
| *Dorea* | | rs12537781 | T | C | 0.230 | -0.056 | 0.013 | 18.8 | 9.15E-06 | -0.003 | 0.021 | 0.872 | |
| *Dorea* | | rs13279148 | G | A | 0.166 | 0.072 | 0.015 | 24.4 | 2.25E-06 | -0.001 | 0.028 | 0.986 | |
| *Dorea* | | rs1899291 | C | T | 0.128 | 0.070 | 0.015 | 18.7 | 4.57E-06 | -0.032 | 0.024 | 0.194 | |
| *Dorea* | | rs3005511 | A | G | 0.336 | 0.052 | 0.011 | 20.5 | 5.29E-06 | -0.065 | 0.019 | 0.001 | |
| *Dorea* | | rs345219 | T | G | 0.376 | -0.050 | 0.011 | 20.0 | 8.80E-06 | -0.006 | 0.018 | 0.738 | |
| *Dorea* | | rs3752849 | G | A | 0.033 | 0.164 | 0.037 | 29.4 | 7.68E-06 | 0.034 | 0.040 | 0.397 | |
| *Dorea* | | rs4793307 | C | T | 0.246 | 0.057 | 0.012 | 21.0 | 4.01E-06 | -0.013 | 0.021 | 0.540 | |
| *Dorea* | | rs62503162 | A | G | 0.088 | -0.097 | 0.019 | 26.1 | 7.47E-07 | 0.011 | 0.043 | 0.802 | |
| *Dorea* | | rs73729431 | C | T | 0.061 | -0.137 | 0.030 | 37.1 | 3.17E-06 | -0.251 | 0.062 | 0.000 | |
| *Eggerthella* | | rs112205261 | T | C | 0.058 | -0.189 | 0.040 | 19.6 | 3.35E-06 | 0.005 | 0.032 | 0.870 | |
| *Eggerthella* | | rs13070736 | A | C | 0.157 | -0.121 | 0.027 | 19.7 | 7.62E-06 | -0.024 | 0.024 | 0.308 | |
| *Eggerthella* | | rs1784446 | G | A | 0.442 | 0.091 | 0.020 | 20.5 | 5.23E-06 | 0.038 | 0.017 | 0.029 | |
| *Eggerthella* | | rs2223081 | G | A | 0.292 | 0.103 | 0.022 | 22.0 | 3.89E-06 | -0.028 | 0.020 | 0.148 | |
| *Eggerthella* | | rs2240838 | A | G | 0.502 | 0.098 | 0.020 | 24.3 | 7.36E-07 | -0.007 | 0.018 | 0.701 | |
| *Eggerthella* | | rs3851328 | T | G | 0.266 | -0.108 | 0.024 | 23.0 | 4.18E-06 | -0.009 | 0.021 | 0.683 | |
| *Eggerthella* | | rs4985746 | G | A | 0.179 | 0.111 | 0.025 | 18.1 | 5.71E-06 | 0.028 | 0.029 | 0.341 | |
| *Eggerthella* | | rs6430926 | C | T | 0.514 | 0.088 | 0.020 | 19.5 | 8.37E-06 | -0.020 | 0.018 | 0.255 | |
| *Eggerthella* | | rs67490567 | T | C | 0.198 | 0.108 | 0.025 | 18.9 | 8.94E-06 | -0.014 | 0.020 | 0.486 | |
| *Eggerthella* | | rs76663501 | C | T | 0.082 | 0.175 | 0.038 | 23.3 | 4.83E-06 | -0.018 | 0.040 | 0.650 | |
| *Eisenbergiella* | | rs11027642 | C | T | 0.124 | 0.129 | 0.028 | 20.3 | 4.92E-06 | 0.010 | 0.025 | 0.679 | |
| *Eisenbergiella* | | rs11079158 | T | C | 0.220 | 0.101 | 0.023 | 19.4 | 7.35E-06 | -0.010 | 0.021 | 0.624 | |
| *Eisenbergiella* | | rs11938607 | T | C | 0.263 | 0.098 | 0.022 | 20.8 | 8.22E-06 | -0.020 | 0.020 | 0.330 | |
| *Eisenbergiella* | | rs12257723 | A | C | 0.339 | -0.095 | 0.021 | 22.8 | 8.85E-06 | 0.000 | 0.019 | 0.994 | |
| *Eisenbergiella* | | rs12710729 | C | A | 0.371 | 0.089 | 0.020 | 20.9 | 9.84E-06 | -0.003 | 0.019 | 0.895 | |
| *Eisenbergiella* | | rs13258851 | A | G | 0.086 | 0.137 | 0.030 | 16.4 | 7.75E-06 | 0.012 | 0.025 | 0.631 | |
| *Eisenbergiella* | | rs1508033 | A | C | 0.350 | 0.092 | 0.020 | 21.4 | 3.23E-06 | -0.010 | 0.019 | 0.611 | |
| *Eisenbergiella* | | rs1553971 | T | G | 0.161 | 0.121 | 0.026 | 22.1 | 5.27E-06 | -0.010 | 0.021 | 0.623 | |
| *Eisenbergiella* | | rs2683098 | C | T | 0.224 | 0.107 | 0.023 | 22.4 | 2.24E-06 | -0.045 | 0.021 | 0.035 | |
| *Eisenbergiella* | | rs3812426 | G | A | 0.208 | 0.106 | 0.022 | 20.9 | 2.72E-06 | 0.020 | 0.024 | 0.421 | |
| *Eisenbergiella* | | rs4462860 | G | A | 0.344 | 0.094 | 0.020 | 22.3 | 4.16E-06 | -0.005 | 0.018 | 0.783 | |
| *Enterorhabdus* | | rs10098492 | T | C | 0.091 | 0.132 | 0.029 | 21.3 | 6.41E-06 | 0.041 | 0.038 | 0.282 | |
| *Enterorhabdus* | | rs114731706 | T | G | 0.084 | 0.182 | 0.038 | 37.6 | 2.17E-06 | -0.052 | 0.050 | 0.298 | |
| *Enterorhabdus* | | rs2051957 | C | T | 0.235 | 0.084 | 0.019 | 18.8 | 8.90E-06 | -0.013 | 0.022 | 0.554 | |
| *Enterorhabdus* | | rs3017103 | A | G | 0.190 | 0.098 | 0.021 | 21.8 | 2.94E-06 | -0.021 | 0.022 | 0.352 | |
| *Enterorhabdus* | | rs73331712 | T | C | 0.043 | 0.262 | 0.055 | 41.5 | 4.85E-06 | 0.082 | 0.044 | 0.061 | |
| *Enterorhabdus* | | rs77655283 | G | A | 0.069 | 0.133 | 0.030 | 16.7 | 5.88E-06 | -0.038 | 0.035 | 0.270 | |
| *Erysipelatoclostridium* | | rs1434153 | G | A | 0.321 | -0.068 | 0.015 | 19.8 | 6.85E-06 | 0.026 | 0.018 | 0.144 | |
| *Erysipelatoclostridium* | | rs16936671 | C | T | 0.136 | -0.097 | 0.022 | 21.3 | 6.04E-06 | 0.031 | 0.025 | 0.220 | |
| *Erysipelatoclostridium* | | rs17804233 | T | C | 0.482 | -0.066 | 0.014 | 21.2 | 4.59E-06 | -0.027 | 0.018 | 0.123 | |
| *Erysipelatoclostridium* | | rs2901723 | C | A | 0.503 | 0.064 | 0.014 | 19.9 | 8.79E-06 | 0.002 | 0.018 | 0.917 | |
| *Erysipelatoclostridium* | | rs340991 | A | G | 0.298 | -0.074 | 0.016 | 22.2 | 3.75E-06 | -0.024 | 0.020 | 0.228 | |
| *Erysipelatoclostridium* | | rs3804326 | A | G | 0.068 | 0.141 | 0.034 | 24.4 | 9.85E-06 | -0.027 | 0.041 | 0.503 | |
| *Erysipelatoclostridium* | | rs45480394 | T | G | 0.362 | -0.069 | 0.015 | 21.1 | 7.66E-06 | 0.020 | 0.018 | 0.272 | |
| *Erysipelatoclostridium* | | rs4697572 | A | G | 0.246 | -0.081 | 0.016 | 23.6 | 7.59E-07 | 0.038 | 0.022 | 0.088 | |
| *Erysipelatoclostridium* | | rs58236560 | G | T | 0.137 | -0.111 | 0.023 | 28.3 | 2.16E-06 | 0.010 | 0.027 | 0.713 | |
| *Erysipelatoclostridium* | | rs61806970 | C | T | 0.065 | 0.143 | 0.032 | 23.8 | 9.09E-06 | -0.061 | 0.035 | 0.081 | |
| *Erysipelatoclostridium* | | rs622418 | A | G | 0.511 | -0.067 | 0.014 | 21.6 | 3.68E-06 | 0.024 | 0.018 | 0.167 | |
| *Erysipelatoclostridium* | | rs6474512 | A | C | 0.451 | 0.067 | 0.014 | 21.5 | 3.02E-06 | -0.007 | 0.018 | 0.714 | |
| *Erysipelatoclostridium* | | rs710230 | T | C | 0.087 | 0.143 | 0.028 | 31.5 | 6.33E-07 | 0.033 | 0.034 | 0.336 | |
| *Erysipelatoclostridium* | | rs7221249 | A | G | 0.547 | 0.084 | 0.014 | 33.9 | 4.31E-09 | 0.010 | 0.018 | 0.569 | |
| *Erysipelatoclostridium* | | rs9590927 | G | A | 0.494 | -0.065 | 0.014 | 20.2 | 6.39E-06 | 0.001 | 0.018 | 0.952 | |
| *Erysipelotrichaceae (UCG003)* | | rs10164067 | T | G | 0.075 | -0.103 | 0.021 | 21.3 | 1.13E-06 | -0.102 | 0.040 | 0.010 | |
| *Erysipelotrichaceae (UCG003)* | | rs11666127 | A | G | 0.171 | -0.072 | 0.016 | 21.2 | 7.90E-06 | -0.022 | 0.023 | 0.338 | |
| *Erysipelotrichaceae (UCG003)* | | rs11994308 | C | T | 0.070 | 0.115 | 0.024 | 24.9 | 1.33E-06 | -0.007 | 0.031 | 0.814 | |
| *Erysipelotrichaceae (UCG003)* | | rs12251396 | A | G | 0.194 | -0.071 | 0.016 | 22.5 | 9.52E-06 | 0.007 | 0.024 | 0.787 | |
| *Erysipelotrichaceae (UCG003)* | | rs17798136 | G | A | 0.062 | 0.159 | 0.035 | 42.2 | 3.24E-06 | -0.043 | 0.036 | 0.231 | |
| *Erysipelotrichaceae (UCG003)* | | rs28568391 | A | G | 0.449 | -0.058 | 0.012 | 24.4 | 6.42E-07 | -0.025 | 0.018 | 0.164 | |
| *Erysipelotrichaceae (UCG003)* | | rs4758231 | G | T | 0.350 | -0.055 | 0.012 | 20.0 | 6.55E-06 | 0.024 | 0.020 | 0.230 | |
| *Erysipelotrichaceae (UCG003)* | | rs59068084 | T | G | 0.422 | 0.056 | 0.012 | 22.5 | 3.12E-06 | -0.026 | 0.018 | 0.146 | |
| *Erysipelotrichaceae (UCG003)* | | rs59104037 | A | G | 0.082 | -0.095 | 0.020 | 19.6 | 4.48E-06 | -0.078 | 0.026 | 0.003 | |
| *Erysipelotrichaceae (UCG003)* | | rs62403464 | T | C | 0.164 | -0.073 | 0.016 | 21.2 | 3.44E-06 | 0.010 | 0.023 | 0.675 | |
| *Erysipelotrichaceae (UCG003)* | | rs6875357 | C | T | 0.047 | 0.166 | 0.035 | 35.3 | 6.70E-06 | -0.040 | 0.046 | 0.382 | |
| *Erysipelotrichaceae (UCG003)* | | rs73074432 | C | T | 0.166 | 0.072 | 0.016 | 20.8 | 9.99E-06 | -0.015 | 0.028 | 0.588 | |
| *Erysipelotrichaceae (UCG003)* | | rs74988980 | G | A | 0.044 | -0.133 | 0.035 | 21.4 | 8.64E-06 | 0.050 | 0.043 | 0.248 | |
| *Erysipelotrichaceae (UCG003)* | | rs75949021 | T | C | 0.046 | -0.170 | 0.037 | 36.3 | 3.58E-06 | 0.046 | 0.045 | 0.303 | |
| *Erysipelotrichaceae (UCG003)* | | rs76502207 | T | C | 0.091 | 0.145 | 0.029 | 50.0 | 6.41E-07 | -0.007 | 0.044 | 0.867 | |
| *Erysipelotrichaceae (UCG003)* | | rs8053479 | A | G | 0.141 | -0.084 | 0.019 | 24.6 | 5.83E-06 | 0.039 | 0.028 | 0.157 | |
| *Escherichia Shigella* | | rs112767262 | T | C | 0.196 | 0.073 | 0.016 | 19.9 | 8.21E-06 | 0.002 | 0.021 | 0.928 | |
| *Escherichia Shigella* | | rs113127095 | A | G | 0.052 | 0.151 | 0.032 | 26.4 | 3.33E-06 | -0.019 | 0.044 | 0.677 | |
| *Escherichia Shigella* | | rs113513883 | A | G | 0.058 | 0.172 | 0.038 | 38.1 | 5.28E-06 | 0.041 | 0.050 | 0.407 | |
| *Escherichia Shigella* | | rs1154904 | A | G | 0.464 | -0.061 | 0.013 | 22.1 | 3.04E-06 | -0.018 | 0.018 | 0.319 | |
| *Escherichia Shigella* | | rs118526 | C | A | 0.393 | -0.059 | 0.014 | 19.9 | 8.00E-06 | 0.004 | 0.019 | 0.825 | |
| *Escherichia Shigella* | | rs2798105 | A | G | 0.118 | -0.101 | 0.022 | 25.0 | 8.25E-06 | 0.007 | 0.029 | 0.826 | |
| *Escherichia Shigella* | | rs4731451 | G | A | 0.372 | -0.061 | 0.014 | 20.5 | 7.47E-06 | -0.002 | 0.019 | 0.904 | |
| *Escherichia Shigella* | | rs57024273 | T | C | 0.318 | 0.063 | 0.014 | 20.1 | 9.70E-06 | 0.036 | 0.020 | 0.076 | |
| *Escherichia Shigella* | | rs592299 | T | C | 0.468 | -0.059 | 0.013 | 20.6 | 4.77E-06 | -0.043 | 0.018 | 0.014 | |
| *Escherichia Shigella* | | rs73208162 | A | G | 0.059 | -0.119 | 0.025 | 18.5 | 2.19E-06 | 0.009 | 0.050 | 0.854 | |
| *Eubacterium (brachy group)* | | rs112617308 | T | C | 0.080 | -0.171 | 0.036 | 17.2 | 2.38E-06 | 0.003 | 0.031 | 0.923 | |
| *Eubacterium (brachy group)* | | rs12151423 | A | G | 0.479 | 0.101 | 0.023 | 20.6 | 9.27E-06 | -0.004 | 0.018 | 0.835 | |
| *Eubacterium (brachy group)* | | rs13139592 | T | C | 0.122 | -0.146 | 0.033 | 18.4 | 7.97E-06 | 0.036 | 0.026 | 0.168 | |
| *Eubacterium (brachy group)* | | rs1384962 | A | G | 0.211 | 0.121 | 0.027 | 19.5 | 6.99E-06 | 0.010 | 0.019 | 0.605 | |
| *Eubacterium (brachy group)* | | rs2913110 | C | T | 0.378 | 0.105 | 0.023 | 20.9 | 4.56E-06 | -0.015 | 0.018 | 0.431 | |
| *Eubacterium (brachy group)* | | rs4862235 | G | A | 0.444 | 0.105 | 0.023 | 21.8 | 3.73E-06 | -0.007 | 0.018 | 0.685 | |
| *Eubacterium (brachy group)* | | rs62348779 | T | C | 0.082 | -0.201 | 0.043 | 24.5 | 3.78E-06 | -0.040 | 0.033 | 0.232 | |
| *Eubacterium (brachy group)* | | rs6591893 | G | A | 0.348 | 0.108 | 0.024 | 21.4 | 7.34E-06 | 0.024 | 0.018 | 0.201 | |
| *Eubacterium (brachy group)* | | rs720439 | A | G | 0.252 | -0.112 | 0.025 | 19.0 | 7.03E-06 | -0.031 | 0.021 | 0.131 | |
| *Eubacterium (brachy group)* | | rs73199919 | T | C | 0.057 | -0.237 | 0.053 | 24.1 | 8.16E-06 | 0.018 | 0.041 | 0.652 | |
| *Eubacterium (coprostanoligenes group)* | | rs1020520 | T | G | 0.204 | -0.059 | 0.013 | 19.7 | 8.89E-06 | -0.018 | 0.025 | 0.473 | |
| *Eubacterium (coprostanoligenes group)* | | rs10444197 | A | G | 0.368 | -0.051 | 0.011 | 20.7 | 5.98E-06 | 0.001 | 0.018 | 0.979 | |
| *Eubacterium (coprostanoligenes group)* | | rs11052069 | T | C | 0.434 | 0.048 | 0.011 | 19.5 | 9.38E-06 | 0.002 | 0.018 | 0.912 | |
| *Eubacterium (coprostanoligenes group)* | | rs11720857 | C | T | 0.177 | 0.063 | 0.014 | 20.1 | 9.26E-06 | -0.015 | 0.023 | 0.504 | |
| *Eubacterium (coprostanoligenes group)* | | rs12906958 | C | T | 0.302 | -0.053 | 0.012 | 20.8 | 4.35E-06 | -0.007 | 0.019 | 0.735 | |
| *Eubacterium (coprostanoligenes group)* | | rs17159861 | C | T | 0.133 | 0.096 | 0.017 | 37.2 | 1.04E-08 | -0.027 | 0.029 | 0.353 | |
| *Eubacterium (coprostanoligenes group)* | | rs2644213 | G | A | 0.237 | 0.054 | 0.012 | 18.2 | 9.86E-06 | 0.032 | 0.019 | 0.095 | |
| *Eubacterium (coprostanoligenes group)* | | rs4076415 | T | G | 0.401 | 0.052 | 0.011 | 22.2 | 1.99E-06 | -0.002 | 0.018 | 0.921 | |
| *Eubacterium (coprostanoligenes group)* | | rs62024432 | C | T | 0.125 | -0.077 | 0.017 | 22.6 | 7.50E-06 | -0.017 | 0.030 | 0.580 | |
| *Eubacterium (coprostanoligenes group)* | | rs6762473 | C | A | 0.357 | 0.052 | 0.011 | 21.7 | 4.26E-06 | 0.029 | 0.018 | 0.114 | |
| *Eubacterium (coprostanoligenes group)* | | rs76898927 | G | A | 0.048 | 0.123 | 0.027 | 23.9 | 4.79E-06 | 0.011 | 0.039 | 0.771 | |
| *Eubacterium (coprostanoligenes group)* | | rs79895140 | T | C | 0.217 | -0.064 | 0.014 | 24.3 | 8.62E-06 | -0.027 | 0.028 | 0.334 | |
| *Eubacterium (coprostanoligenes group)* | | rs9648214 | T | C | 0.118 | -0.083 | 0.016 | 24.9 | 2.52E-07 | -0.003 | 0.032 | 0.928 | |
| *Eubacterium (eligens group)* | | rs182318 | G | A | 0.078 | -0.082 | 0.020 | 14.3 | 8.40E-06 | -0.096 | 0.032 | 0.003 | |
| *Eubacterium (eligens group)* | | rs2200429 | A | G | 0.074 | -0.089 | 0.020 | 15.8 | 5.30E-06 | -0.075 | 0.030 | 0.011 | |
| *Eubacterium (eligens group)* | | rs265534 | T | G | 0.455 | -0.056 | 0.012 | 23.2 | 2.27E-06 | 0.022 | 0.018 | 0.215 | |
| *Eubacterium (eligens group)* | | rs4583233 | A | C | 0.334 | 0.067 | 0.013 | 29.4 | 2.84E-07 | 0.009 | 0.020 | 0.660 | |
| *Eubacterium (eligens group)* | | rs56080211 | C | T | 0.056 | 0.123 | 0.028 | 23.4 | 9.14E-06 | 0.056 | 0.033 | 0.090 | |
| *Eubacterium (eligens group)* | | rs6923695 | T | G | 0.057 | 0.103 | 0.023 | 16.8 | 4.87E-06 | 0.003 | 0.035 | 0.928 | |
| *Eubacterium (fissicatena group)* | | rs10147907 | T | G | 0.069 | 0.172 | 0.040 | 14.6 | 8.27E-06 | 0.059 | 0.033 | 0.077 | |
| *Eubacterium (fissicatena group)* | | rs11818408 | G | A | 0.477 | 0.106 | 0.024 | 21.5 | 8.20E-06 | -0.006 | 0.018 | 0.750 | |
| *Eubacterium (fissicatena group)* | | rs11876297 | T | C | 0.216 | 0.131 | 0.028 | 22.5 | 2.67E-06 | 0.008 | 0.020 | 0.700 | |
| *Eubacterium (fissicatena group)* | | rs151257695 | A | G | 0.081 | 0.210 | 0.045 | 25.1 | 3.10E-06 | 0.034 | 0.034 | 0.327 | |
| *Eubacterium (fissicatena group)* | | rs1768152 | T | C | 0.137 | 0.139 | 0.032 | 17.7 | 8.70E-06 | -0.027 | 0.029 | 0.348 | |
| *Eubacterium (fissicatena group)* | | rs2733072 | G | A | 0.465 | 0.110 | 0.023 | 23.1 | 1.49E-06 | 0.023 | 0.018 | 0.194 | |
| *Eubacterium (fissicatena group)* | | rs3771393 | C | T | 0.284 | 0.131 | 0.027 | 26.9 | 7.38E-07 | -0.039 | 0.022 | 0.081 | |
| *Eubacterium (fissicatena group)* | | rs6934739 | A | G | 0.295 | 0.111 | 0.025 | 19.9 | 9.75E-06 | -0.014 | 0.019 | 0.457 | |
| *Eubacterium (fissicatena group)* | | rs7104872 | G | A | 0.178 | 0.139 | 0.029 | 21.7 | 2.73E-06 | 0.021 | 0.028 | 0.467 | |
| *Eubacterium (hallii group)* | | rs10501370 | C | T | 0.053 | -0.116 | 0.025 | 18.5 | 5.42E-06 | -0.006 | 0.036 | 0.861 | |
| *Eubacterium (hallii group)* | | rs10798999 | C | T | 0.216 | 0.060 | 0.013 | 16.9 | 2.61E-06 | -0.012 | 0.020 | 0.561 | |
| *Eubacterium (hallii group)* | | rs10808115 | A | C | 0.478 | -0.050 | 0.011 | 17.6 | 4.42E-06 | 0.020 | 0.018 | 0.266 | |
| *Eubacterium (hallii group)* | | rs117748144 | T | C | 0.043 | -0.127 | 0.029 | 18.1 | 7.86E-06 | -0.005 | 0.040 | 0.895 | |
| *Eubacterium (hallii group)* | | rs13116360 | T | C | 0.063 | 0.154 | 0.030 | 38.6 | 2.94E-07 | -0.004 | 0.036 | 0.908 | |
| *Eubacterium (hallii group)* | | rs138531890 | A | G | 0.054 | 0.153 | 0.035 | 33.0 | 5.43E-06 | 0.018 | 0.033 | 0.591 | |
| *Eubacterium (hallii group)* | | rs17074066 | T | C | 0.092 | -0.081 | 0.019 | 15.2 | 9.35E-06 | 0.004 | 0.063 | 0.943 | |
| *Eubacterium (hallii group)* | | rs17474256 | G | A | 0.123 | 0.081 | 0.018 | 19.7 | 9.45E-06 | -0.015 | 0.030 | 0.613 | |
| *Eubacterium (hallii group)* | | rs281379 | A | G | 0.460 | -0.050 | 0.011 | 17.2 | 9.33E-06 | 0.033 | 0.018 | 0.064 | |
| *Eubacterium (hallii group)* | | rs28584818 | A | G | 0.069 | 0.126 | 0.027 | 28.2 | 4.43E-06 | 0.012 | 0.033 | 0.716 | |
| *Eubacterium (hallii group)* | | rs60254196 | A | G | 0.541 | -0.052 | 0.011 | 18.8 | 2.70E-06 | 0.012 | 0.018 | 0.484 | |
| *Eubacterium (hallii group)* | | rs630939 | C | T | 0.400 | -0.051 | 0.011 | 17.2 | 9.16E-06 | 0.039 | 0.018 | 0.027 | |
| *Eubacterium (hallii group)* | | rs6550770 | T | C | 0.052 | -0.198 | 0.044 | 53.4 | 4.82E-06 | -0.017 | 0.045 | 0.702 | |
| *Eubacterium (hallii group)* | | rs74018587 | C | T | 0.037 | 0.209 | 0.044 | 42.9 | 3.70E-06 | -0.051 | 0.045 | 0.252 | |
| *Eubacterium (hallii group)* | | rs78056098 | G | T | 0.361 | -0.051 | 0.011 | 16.4 | 8.29E-06 | -0.027 | 0.018 | 0.140 | |
| *Eubacterium (hallii group)* | | rs949971 | T | G | 0.384 | -0.054 | 0.012 | 19.1 | 3.29E-06 | 0.014 | 0.019 | 0.458 | |
| *Eubacterium (nodatum group)* | | rs10263623 | C | T | 0.104 | 0.193 | 0.044 | 21.7 | 8.91E-06 | -0.018 | 0.046 | 0.693 | |
| *Eubacterium (nodatum group)* | | rs10458299 | T | C | 0.081 | -0.188 | 0.042 | 16.1 | 8.37E-06 | -0.025 | 0.034 | 0.448 | |
| *Eubacterium (nodatum group)* | | rs11006576 | A | G | 0.513 | -0.110 | 0.025 | 18.8 | 7.99E-06 | -0.030 | 0.018 | 0.093 | |
| *Eubacterium (nodatum group)* | | rs113893692 | C | T | 0.098 | -0.185 | 0.040 | 18.8 | 5.76E-06 | -0.021 | 0.027 | 0.430 | |
| *Eubacterium (nodatum group)* | | rs34297067 | A | G | 0.139 | -0.187 | 0.034 | 26.0 | 6.60E-08 | 0.030 | 0.025 | 0.232 | |
| *Eubacterium (nodatum group)* | | rs61841040 | G | T | 0.166 | 0.161 | 0.034 | 22.1 | 3.56E-06 | -0.024 | 0.022 | 0.281 | |
| *Eubacterium (nodatum group)* | | rs6818880 | A | G | 0.455 | -0.110 | 0.025 | 18.6 | 7.83E-06 | -0.010 | 0.018 | 0.585 | |
| *Eubacterium (nodatum group)* | | rs77910827 | C | T | 0.103 | 0.202 | 0.041 | 23.4 | 9.05E-07 | 0.002 | 0.028 | 0.937 | |
| *Eubacterium (nodatum group)* | | rs7827125 | C | T | 0.282 | 0.122 | 0.027 | 18.8 | 7.17E-06 | -0.021 | 0.020 | 0.291 | |
| *Eubacterium (nodatum group)* | | rs7880204 | T | C | 0.248 | -0.125 | 0.028 | 18.2 | 6.84E-06 | 0.002 | 0.020 | 0.938 | |
| *Eubacterium (nodatum group)* | | rs9425984 | T | C | 0.239 | -0.130 | 0.029 | 19.1 | 7.21E-06 | -0.036 | 0.021 | 0.091 | |
| *Eubacterium (oxidoreducens group)* | | rs12129908 | C | A | 0.447 | 0.089 | 0.020 | 21.3 | 5.80E-06 | -0.030 | 0.018 | 0.096 | |
| *Eubacterium (oxidoreducens group)* | | rs12423772 | G | T | 0.150 | 0.141 | 0.030 | 27.4 | 2.63E-06 | -0.021 | 0.025 | 0.417 | |
| *Eubacterium (oxidoreducens group)* | | rs2973294 | G | T | 0.421 | 0.092 | 0.020 | 22.4 | 2.39E-06 | 0.017 | 0.018 | 0.328 | |
| *Eubacterium (oxidoreducens group)* | | rs34561138 | G | A | 0.059 | 0.216 | 0.046 | 27.8 | 2.51E-06 | -0.045 | 0.046 | 0.325 | |
| *Eubacterium (oxidoreducens group)* | | rs440215 | C | T | 0.462 | 0.093 | 0.020 | 23.4 | 1.65E-06 | -0.029 | 0.018 | 0.100 | |
| *Eubacterium (rectale group)* | | rs10248854 | C | A | 0.360 | -0.053 | 0.011 | 22.3 | 4.21E-06 | 0.020 | 0.018 | 0.277 | |
| *Eubacterium (rectale group)* | | rs10797540 | A | G | 0.434 | 0.050 | 0.011 | 21.6 | 3.53E-06 | -0.022 | 0.018 | 0.219 | |
| *Eubacterium (rectale group)* | | rs143694765 | T | C | 0.094 | 0.087 | 0.020 | 22.5 | 9.75E-06 | 0.070 | 0.029 | 0.016 | |
| *Eubacterium (rectale group)* | | rs2884897 | A | G | 0.053 | -0.129 | 0.029 | 29.0 | 6.44E-06 | 0.082 | 0.048 | 0.088 | |
| *Eubacterium (rectale group)* | | rs314726 | T | C | 0.457 | 0.053 | 0.011 | 24.1 | 1.38E-06 | 0.013 | 0.018 | 0.451 | |
| *Eubacterium (rectale group)* | | rs35398954 | A | G | 0.121 | -0.090 | 0.017 | 30.1 | 5.40E-07 | 0.027 | 0.024 | 0.261 | |
| *Eubacterium (rectale group)* | | rs59427698 | A | G | 0.203 | -0.058 | 0.013 | 18.6 | 5.37E-06 | -0.009 | 0.022 | 0.682 | |
| *Eubacterium (rectale group)* | | rs62547233 | A | G | 0.307 | 0.054 | 0.012 | 21.3 | 9.90E-06 | 0.003 | 0.019 | 0.897 | |
| *Eubacterium (ruminantium group)* | | rs10131724 | A | C | 0.064 | -0.200 | 0.041 | 24.2 | 2.39E-06 | 0.016 | 0.030 | 0.586 | |
| *Eubacterium (ruminantium group)* | | rs10923018 | G | A | 0.472 | 0.073 | 0.016 | 20.4 | 6.80E-06 | 0.005 | 0.018 | 0.776 | |
| *Eubacterium (ruminantium group)* | | rs11637981 | G | T | 0.484 | -0.073 | 0.016 | 20.7 | 5.44E-06 | 0.028 | 0.018 | 0.117 | |
| *Eubacterium (ruminantium group)* | | rs13025464 | T | C | 0.383 | -0.074 | 0.016 | 19.9 | 6.97E-06 | 0.027 | 0.018 | 0.128 | |
| *Eubacterium (ruminantium group)* | | rs139749 | C | T | 0.342 | -0.085 | 0.017 | 23.6 | 8.59E-07 | -0.020 | 0.019 | 0.296 | |
| *Eubacterium (ruminantium group)* | | rs16891896 | G | A | 0.059 | -0.175 | 0.039 | 16.1 | 2.38E-06 | 0.000 | 0.032 | 0.991 | |
| *Eubacterium (ruminantium group)* | | rs17519472 | C | T | 0.154 | 0.108 | 0.023 | 23.4 | 4.70E-06 | -0.004 | 0.025 | 0.885 | |
| *Eubacterium (ruminantium group)* | | rs209813 | G | A | 0.166 | -0.103 | 0.024 | 21.6 | 9.23E-06 | 0.006 | 0.025 | 0.826 | |
| *Eubacterium (ruminantium group)* | | rs2116427 | A | G | 0.257 | 0.091 | 0.018 | 24.6 | 4.67E-07 | -0.010 | 0.020 | 0.607 | |
| *Eubacterium (ruminantium group)* | | rs2229917 | A | G | 0.067 | 0.154 | 0.032 | 20.7 | 2.16E-06 | -0.049 | 0.044 | 0.261 | |
| *Eubacterium (ruminantium group)* | | rs2418654 | C | T | 0.478 | -0.075 | 0.017 | 20.5 | 6.17E-06 | 0.005 | 0.018 | 0.764 | |
| *Eubacterium (ruminantium group)* | | rs2817174 | C | T | 0.429 | -0.073 | 0.016 | 20.4 | 7.87E-06 | -0.002 | 0.018 | 0.901 | |
| *Eubacterium (ruminantium group)* | | rs57340348 | T | C | 0.215 | -0.098 | 0.021 | 23.7 | 4.93E-06 | 0.021 | 0.022 | 0.336 | |
| *Eubacterium (ruminantium group)* | | rs606117 | A | G | 0.332 | 0.083 | 0.018 | 22.4 | 4.82E-06 | -0.018 | 0.020 | 0.364 | |
| *Eubacterium (ruminantium group)* | | rs6676699 | G | T | 0.222 | -0.089 | 0.020 | 21.0 | 6.38E-06 | -0.001 | 0.019 | 0.963 | |
| *Eubacterium (ruminantium group)* | | rs7000472 | A | G | 0.362 | -0.076 | 0.017 | 20.8 | 4.07E-06 | 0.027 | 0.018 | 0.139 | |
| *Eubacterium (ruminantium group)* | | rs72836424 | C | T | 0.095 | -0.140 | 0.030 | 24.8 | 2.62E-06 | 0.073 | 0.028 | 0.010 | |
| *Eubacterium (ruminantium group)* | | rs73139629 | A | C | 0.132 | -0.115 | 0.025 | 22.3 | 5.36E-06 | 0.003 | 0.031 | 0.916 | |
| *Eubacterium (ventriosum group)* | | rs11617697 | A | G | 0.048 | -0.143 | 0.029 | 21.5 | 7.22E-07 | 0.011 | 0.039 | 0.770 | |
| *Eubacterium (ventriosum group)* | | rs12964517 | G | A | 0.316 | 0.059 | 0.012 | 17.2 | 2.07E-06 | -0.019 | 0.020 | 0.340 | |
| *Eubacterium (ventriosum group)* | | rs13082419 | C | T | 0.170 | -0.072 | 0.016 | 16.6 | 9.56E-06 | 0.004 | 0.028 | 0.885 | |
| *Eubacterium (ventriosum group)* | | rs16884680 | G | T | 0.107 | -0.091 | 0.019 | 18.1 | 1.74E-06 | 0.002 | 0.029 | 0.935 | |
| *Eubacterium (ventriosum group)* | | rs35179274 | C | T | 0.244 | -0.063 | 0.014 | 16.7 | 5.76E-06 | -0.003 | 0.023 | 0.908 | |
| *Eubacterium (ventriosum group)* | | rs3809430 | T | C | 0.348 | -0.055 | 0.012 | 15.7 | 3.55E-06 | 0.003 | 0.019 | 0.878 | |
| *Eubacterium (ventriosum group)* | | rs57199565 | T | C | 0.241 | 0.078 | 0.016 | 25.8 | 7.97E-07 | -0.026 | 0.023 | 0.263 | |
| *Eubacterium (ventriosum group)* | | rs66746423 | C | T | 0.156 | 0.075 | 0.016 | 17.1 | 6.11E-06 | 0.012 | 0.024 | 0.610 | |
| *Eubacterium (ventriosum group)* | | rs6704822 | A | G | 0.116 | 0.074 | 0.017 | 12.9 | 6.62E-06 | -0.035 | 0.026 | 0.172 | |
| *Eubacterium (ventriosum group)* | | rs72783037 | C | A | 0.187 | 0.066 | 0.014 | 15.2 | 6.55E-06 | 0.009 | 0.022 | 0.690 | |
| *Eubacterium (ventriosum group)* | | rs73615400 | T | C | 0.091 | -0.096 | 0.019 | 17.3 | 9.54E-07 | 0.024 | 0.030 | 0.419 | |
| *Eubacterium (ventriosum group)* | | rs73849225 | T | C | 0.080 | 0.098 | 0.022 | 16.0 | 5.21E-06 | -0.015 | 0.032 | 0.640 | |
| *Eubacterium (ventriosum group)* | | rs78250280 | G | A | 0.149 | 0.075 | 0.016 | 16.4 | 3.36E-06 | -0.003 | 0.025 | 0.922 | |
| *Eubacterium (ventriosum group)* | | rs876734 | C | T | 0.289 | -0.062 | 0.013 | 18.1 | 2.89E-06 | 0.016 | 0.020 | 0.409 | |
| *Eubacterium (ventriosum group)* | | rs9316536 | T | G | 0.136 | -0.082 | 0.018 | 18.1 | 7.84E-06 | -0.042 | 0.025 | 0.100 | |
| *Eubacterium (xylanophilum group)* | | rs10140184 | A | C | 0.413 | 0.058 | 0.013 | 20.9 | 4.96E-06 | -0.031 | 0.018 | 0.084 | |
| *Eubacterium (xylanophilum group)* | | rs10917203 | A | C | 0.335 | 0.061 | 0.013 | 21.7 | 3.15E-06 | 0.060 | 0.018 | 0.001 | |
| *Eubacterium (xylanophilum group)* | | rs112176119 | C | T | 0.065 | -0.113 | 0.025 | 20.1 | 3.33E-06 | 0.001 | 0.031 | 0.971 | |
| *Eubacterium (xylanophilum group)* | | rs13239072 | G | A | 0.275 | 0.069 | 0.014 | 24.4 | 1.82E-06 | 0.006 | 0.020 | 0.780 | |
| *Eubacterium (xylanophilum group)* | | rs17830032 | G | A | 0.054 | -0.161 | 0.031 | 34.0 | 2.39E-07 | 0.022 | 0.033 | 0.490 | |
| *Eubacterium (xylanophilum group)* | | rs1999224 | G | T | 0.089 | -0.095 | 0.020 | 18.8 | 3.75E-06 | -0.003 | 0.030 | 0.924 | |
| *Eubacterium (xylanophilum group)* | | rs2012708 | A | G | 0.364 | 0.057 | 0.013 | 19.7 | 6.53E-06 | -0.018 | 0.019 | 0.336 | |
| *Eubacterium (xylanophilum group)* | | rs2213117 | T | G | 0.161 | 0.088 | 0.019 | 27.0 | 4.21E-06 | -0.017 | 0.024 | 0.471 | |
| *Eubacterium (xylanophilum group)* | | rs75586835 | A | G | 0.067 | -0.114 | 0.026 | 21.1 | 9.39E-06 | 0.059 | 0.035 | 0.087 | |
| *Faecalibacterium* | | rs114946999 | C | T | 0.098 | -0.086 | 0.019 | 20.7 | 5.70E-06 | 0.026 | 0.027 | 0.331 | |
| *Faecalibacterium* | | rs11776390 | T | C | 0.090 | -0.078 | 0.017 | 16.7 | 6.40E-06 | 0.015 | 0.035 | 0.674 | |
| *Faecalibacterium* | | rs1271565 | C | T | 0.272 | -0.058 | 0.012 | 23.2 | 1.30E-06 | -0.033 | 0.020 | 0.104 | |
| *Faecalibacterium* | | rs12753492 | A | C | 0.149 | 0.064 | 0.015 | 18.3 | 8.80E-06 | 0.019 | 0.028 | 0.501 | |
| *Faecalibacterium* | | rs2835874 | T | C | 0.070 | -0.087 | 0.020 | 17.1 | 7.54E-06 | -0.045 | 0.048 | 0.345 | |
| *Faecalibacterium* | | rs6910935 | A | G | 0.054 | 0.135 | 0.028 | 23.4 | 1.38E-06 | 0.023 | 0.037 | 0.533 | |
| *Faecalibacterium* | | rs75499067 | C | T | 0.064 | 0.228 | 0.047 | 28.1 | 1.76E-06 | -0.034 | 0.034 | 0.309 | |
| *Faecalibacterium* | | rs79656633 | T | C | 0.105 | 0.146 | 0.032 | 36.5 | 8.14E-06 | 0.022 | 0.029 | 0.464 | |
| *Faecalibacterium* | | rs9536330 | T | C | 0.409 | -0.048 | 0.011 | 20.4 | 5.33E-06 | 0.029 | 0.018 | 0.101 | |
| *Family XIIIAD (3011 group)* | | rs11126423 | C | T | 0.113 | 0.090 | 0.020 | 22.6 | 5.91E-06 | 0.028 | 0.031 | 0.366 | |
| *Family XIIIAD (3011 group)* | | rs11736617 | G | A | 0.120 | -0.076 | 0.017 | 16.8 | 9.02E-06 | 0.007 | 0.039 | 0.859 | |
| *Family XIIIAD (3011 group)* | | rs12812672 | T | C | 0.074 | -0.096 | 0.021 | 17.3 | 2.56E-06 | -0.021 | 0.034 | 0.535 | |
| *Family XIIIAD (3011 group)* | | rs149302 | T | C | 0.213 | -0.065 | 0.014 | 19.2 | 7.48E-06 | 0.009 | 0.021 | 0.661 | |
| *Family XIIIAD (3011 group)* | | rs16840310 | A | G | 0.372 | -0.061 | 0.012 | 23.8 | 6.75E-07 | -0.027 | 0.018 | 0.133 | |
| *Family XIIIAD (3011 group)* | | rs16940167 | C | T | 0.169 | 0.073 | 0.016 | 20.7 | 3.91E-06 | 0.009 | 0.022 | 0.695 | |
| *Family XIIIAD (3011 group)* | | rs17156849 | G | A | 0.063 | -0.113 | 0.025 | 20.6 | 4.19E-06 | 0.077 | 0.037 | 0.039 | |
| *Family XIIIAD (3011 group)* | | rs62029761 | A | G | 0.068 | 0.129 | 0.028 | 28.8 | 3.89E-06 | -0.012 | 0.039 | 0.770 | |
| *Family XIIIAD (3011 group)* | | rs62200412 | C | T | 0.171 | -0.080 | 0.016 | 25.0 | 5.80E-07 | -0.017 | 0.020 | 0.389 | |
| *Family XIIIAD (3011 group)* | | rs72730932 | C | A | 0.164 | -0.090 | 0.018 | 30.5 | 6.89E-07 | -0.022 | 0.030 | 0.475 | |
| *Family XIIIAD (3011 group)* | | rs739451 | C | T | 0.176 | 0.065 | 0.015 | 16.8 | 7.88E-06 | 0.015 | 0.022 | 0.502 | |
| *Family XIIIAD (3011 group)* | | rs9276029 | A | G | 0.168 | -0.081 | 0.019 | 25.3 | 8.93E-06 | 0.006 | 0.024 | 0.811 | |
| *Family XIIIAD (3011 group)* | | rs9837139 | A | G | 0.082 | 0.108 | 0.024 | 23.8 | 8.71E-06 | 0.022 | 0.032 | 0.498 | |
| *Family XIII (UCG001)* | | rs112362903 | A | G | 0.043 | -0.149 | 0.033 | 15.6 | 7.88E-06 | 0.018 | 0.048 | 0.708 | |
| *Family XIII (UCG001)* | | rs12049454 | T | C | 0.301 | -0.065 | 0.013 | 15.2 | 1.17E-06 | 0.013 | 0.018 | 0.488 | |
| *Family XIII (UCG001)* | | rs1426266 | T | C | 0.296 | -0.067 | 0.014 | 15.9 | 1.25E-06 | -0.046 | 0.020 | 0.020 | |
| *Family XIII (UCG001)* | | rs3842897 | G | A | 0.065 | -0.113 | 0.024 | 13.2 | 5.20E-06 | 0.013 | 0.031 | 0.680 | |
| *Family XIII (UCG001)* | | rs62414802 | C | T | 0.298 | -0.061 | 0.013 | 13.5 | 4.29E-06 | -0.020 | 0.020 | 0.330 | |
| *Family XIII (UCG001)* | | rs7119679 | G | A | 0.150 | -0.081 | 0.017 | 14.3 | 3.52E-06 | 0.004 | 0.021 | 0.853 | |
| *Family XIII (UCG001)* | | rs76463770 | A | G | 0.052 | 0.193 | 0.042 | 31.5 | 3.77E-06 | 0.011 | 0.052 | 0.833 | |
| *Family XIII (UCG001)* | | rs8076666 | A | G | 0.136 | 0.089 | 0.020 | 15.9 | 8.02E-06 | -0.004 | 0.027 | 0.895 | |
| *Flavonifractor* | | rs114873521 | C | T | 0.077 | -0.130 | 0.029 | 21.9 | 7.13E-06 | -0.014 | 0.034 | 0.690 | |
| *Flavonifractor* | | rs11811696 | T | C | 0.110 | -0.116 | 0.024 | 24.3 | 2.07E-06 | 0.030 | 0.032 | 0.349 | |
| *Flavonifractor* | | rs12030302 | A | G | 0.491 | -0.069 | 0.014 | 22.0 | 5.61E-07 | 0.020 | 0.018 | 0.250 | |
| *Flavonifractor* | | rs34066017 | A | G | 0.246 | 0.076 | 0.016 | 19.9 | 1.52E-06 | -0.041 | 0.022 | 0.059 | |
| *Flavonifractor* | | rs806808 | T | C | 0.476 | 0.067 | 0.014 | 20.4 | 1.18E-06 | -0.003 | 0.018 | 0.875 | |
| *Fusicatenibacter* | | rs10439674 | A | G | 0.202 | -0.057 | 0.013 | 18.3 | 7.68E-06 | -0.010 | 0.022 | 0.639 | |
| *Fusicatenibacter* | | rs167879 | C | T | 0.167 | -0.066 | 0.015 | 21.1 | 5.87E-06 | 0.000 | 0.025 | 0.998 | |
| *Fusicatenibacter* | | rs1864685 | A | C | 0.465 | -0.049 | 0.011 | 21.2 | 4.96E-06 | 0.019 | 0.018 | 0.300 | |
| *Fusicatenibacter* | | rs2025938 | G | A | 0.077 | -0.097 | 0.021 | 23.0 | 2.99E-06 | 0.062 | 0.036 | 0.085 | |
| *Fusicatenibacter* | | rs206581 | A | G | 0.209 | -0.057 | 0.013 | 18.6 | 8.96E-06 | -0.003 | 0.021 | 0.905 | |
| *Fusicatenibacter* | | rs2132128 | G | A | 0.165 | -0.077 | 0.016 | 28.6 | 1.08E-06 | -0.012 | 0.029 | 0.674 | |
| *Fusicatenibacter* | | rs3303 | T | C | 0.077 | -0.095 | 0.020 | 22.4 | 3.94E-06 | 0.010 | 0.036 | 0.786 | |
| *Fusicatenibacter* | | rs4378146 | A | C | 0.242 | -0.062 | 0.013 | 24.3 | 7.20E-07 | 0.001 | 0.020 | 0.964 | |
| *Fusicatenibacter* | | rs60254196 | A | G | 0.541 | -0.049 | 0.011 | 21.0 | 5.47E-06 | 0.012 | 0.018 | 0.484 | |
| *Fusicatenibacter* | | rs62187631 | T | C | 0.140 | -0.071 | 0.016 | 21.2 | 4.55E-06 | 0.040 | 0.022 | 0.074 | |
| *Fusicatenibacter* | | rs62353480 | A | G | 0.171 | -0.070 | 0.015 | 24.3 | 1.57E-06 | 0.024 | 0.024 | 0.300 | |
| *Fusicatenibacter* | | rs6515626 | G | A | 0.062 | 0.142 | 0.031 | 40.4 | 7.29E-06 | 0.024 | 0.035 | 0.503 | |
| *Fusicatenibacter* | | rs704418 | T | C | 0.186 | 0.074 | 0.015 | 28.8 | 7.77E-07 | -0.014 | 0.027 | 0.604 | |
| *Fusicatenibacter* | | rs73103914 | A | G | 0.178 | -0.060 | 0.013 | 18.2 | 8.30E-06 | -0.031 | 0.025 | 0.204 | |
| *Fusicatenibacter* | | rs792108 | T | C | 0.354 | -0.051 | 0.011 | 20.6 | 8.50E-06 | 0.036 | 0.018 | 0.045 | |
| *Fusicatenibacter* | | rs8028026 | A | G | 0.092 | -0.079 | 0.018 | 18.1 | 8.06E-06 | 0.044 | 0.031 | 0.147 | |
| *Fusicatenibacter* | | rs8063430 | T | C | 0.060 | -0.104 | 0.022 | 21.1 | 4.93E-06 | -0.025 | 0.040 | 0.535 | |
| *Fusicatenibacter* | | rs9905659 | G | A | 0.188 | -0.062 | 0.014 | 20.2 | 7.31E-06 | -0.025 | 0.023 | 0.265 | |
| *Gordonibacter* | | rs13412653 | A | C | 0.354 | 0.108 | 0.024 | 19.6 | 8.61E-06 | 0.000 | 0.018 | 0.985 | |
| *Gordonibacter* | | rs16955299 | G | A | 0.084 | -0.196 | 0.043 | 21.9 | 6.37E-06 | 0.045 | 0.030 | 0.130 | |
| *Gordonibacter* | | rs322296 | G | A | 0.119 | 0.179 | 0.038 | 24.9 | 4.02E-06 | 0.002 | 0.034 | 0.948 | |
| *Gordonibacter* | | rs35042269 | C | A | 0.189 | -0.180 | 0.040 | 37.0 | 8.11E-06 | 0.003 | 0.028 | 0.927 | |
| *Gordonibacter* | | rs3765837 | T | G | 0.079 | -0.191 | 0.043 | 19.5 | 7.17E-06 | -0.024 | 0.034 | 0.485 | |
| *Gordonibacter* | | rs4596722 | A | G | 0.476 | 0.103 | 0.023 | 19.6 | 9.06E-06 | -0.001 | 0.018 | 0.951 | |
| *Gordonibacter* | | rs61934597 | C | T | 0.076 | -0.172 | 0.039 | 15.4 | 8.37E-06 | 0.028 | 0.036 | 0.438 | |
| *Gordonibacter* | | rs71545975 | A | G | 0.173 | -0.154 | 0.034 | 25.1 | 7.04E-06 | 0.004 | 0.023 | 0.864 | |
| *Gordonibacter* | | rs72714787 | C | A | 0.104 | 0.181 | 0.038 | 22.8 | 1.43E-06 | 0.038 | 0.026 | 0.141 | |
| *Gordonibacter* | | rs72939513 | A | G | 0.054 | -0.214 | 0.049 | 17.2 | 7.98E-06 | -0.043 | 0.040 | 0.286 | |
| *Gordonibacter* | | rs7294633 | C | T | 0.351 | 0.129 | 0.025 | 28.0 | 3.44E-07 | -0.021 | 0.020 | 0.295 | |
| *Gordonibacter* | | rs768830 | G | A | 0.129 | 0.150 | 0.033 | 18.7 | 7.76E-06 | 0.036 | 0.024 | 0.139 | |
| *Haemophilus* | | rs10781340 | G | A | 0.166 | 0.095 | 0.020 | 22.6 | 4.32E-06 | -0.026 | 0.027 | 0.334 | |
| *Haemophilus* | | rs111582866 | G | A | 0.101 | -0.124 | 0.026 | 25.5 | 1.27E-06 | 0.035 | 0.031 | 0.267 | |
| *Haemophilus* | | rs35509 | G | A | 0.079 | 0.128 | 0.027 | 21.6 | 2.01E-06 | -0.054 | 0.044 | 0.213 | |
| *Haemophilus* | | rs4822728 | T | C | 0.437 | 0.071 | 0.015 | 22.2 | 3.48E-06 | -0.008 | 0.018 | 0.660 | |
| *Haemophilus* | | rs76022354 | C | T | 0.033 | 0.245 | 0.051 | 34.4 | 1.83E-06 | 0.009 | 0.041 | 0.829 | |
| *Haemophilus* | | rs78909003 | T | C | 0.050 | -0.246 | 0.050 | 52.1 | 1.67E-06 | 0.026 | 0.039 | 0.499 | |
| *Haemophilus* | | rs9328464 | T | C | 0.492 | 0.072 | 0.015 | 23.7 | 1.42E-06 | -0.010 | 0.018 | 0.580 | |
| *Haemophilus* | | rs9382510 | C | T | 0.246 | -0.094 | 0.017 | 29.4 | 7.12E-08 | 0.007 | 0.020 | 0.745 | |
| *Haemophilus* | | rs9895850 | T | C | 0.050 | -0.193 | 0.042 | 31.9 | 2.14E-06 | -0.018 | 0.043 | 0.682 | |
| *Holdemanella* | | rs12513188 | G | A | 0.222 | 0.090 | 0.020 | 21.7 | 4.65E-06 | 0.038 | 0.020 | 0.056 | |
| *Holdemanella* | | rs17586763 | T | C | 0.018 | -0.227 | 0.051 | 13.9 | 7.72E-06 | 0.021 | 0.040 | 0.601 | |
| *Holdemanella* | | rs1926302 | G | A | 0.153 | -0.108 | 0.023 | 23.2 | 7.50E-06 | -0.024 | 0.021 | 0.266 | |
| *Holdemanella* | | rs34187114 | C | A | 0.166 | -0.105 | 0.023 | 23.2 | 5.13E-06 | 0.021 | 0.028 | 0.454 | |
| *Holdemanella* | | rs35228298 | G | A | 0.247 | 0.093 | 0.020 | 24.9 | 7.30E-06 | -0.013 | 0.024 | 0.598 | |
| *Holdemanella* | | rs4541991 | T | C | 0.236 | -0.093 | 0.019 | 23.8 | 2.10E-06 | 0.030 | 0.019 | 0.109 | |
| *Holdemanella* | | rs607782 | T | C | 0.382 | -0.085 | 0.017 | 26.5 | 7.19E-07 | -0.016 | 0.018 | 0.380 | |
| *Holdemanella* | | rs62113381 | T | C | 0.159 | -0.105 | 0.023 | 22.8 | 5.54E-06 | -0.064 | 0.026 | 0.014 | |
| *Holdemanella* | | rs73011279 | T | C | 0.208 | -0.096 | 0.020 | 23.4 | 1.36E-06 | -0.018 | 0.021 | 0.382 | |
| *Holdemanella* | | rs75764681 | T | C | 0.063 | -0.283 | 0.060 | 72.7 | 1.94E-06 | -0.054 | 0.043 | 0.208 | |
| *Holdemanella* | | rs8113760 | G | A | 0.352 | 0.079 | 0.017 | 21.9 | 4.62E-06 | -0.020 | 0.019 | 0.284 | |
| *Holdemania* | | rs10885477 | T | C | 0.062 | -0.135 | 0.030 | 18.4 | 8.60E-06 | 0.138 | 0.040 | 0.001 | |
| *Holdemania* | | rs11080063 | G | A | 0.466 | -0.067 | 0.015 | 19.2 | 6.67E-06 | -0.019 | 0.018 | 0.298 | |
| *Holdemania* | | rs111745969 | A | G | 0.080 | 0.121 | 0.027 | 18.6 | 3.71E-06 | -0.028 | 0.026 | 0.275 | |
| *Holdemania* | | rs113593397 | A | G | 0.102 | -0.129 | 0.028 | 26.6 | 9.36E-06 | 0.022 | 0.030 | 0.452 | |
| *Holdemania* | | rs116500994 | G | T | 0.052 | -0.138 | 0.029 | 16.1 | 2.34E-06 | -0.040 | 0.041 | 0.338 | |
| *Holdemania* | | rs12701617 | A | G | 0.458 | -0.066 | 0.015 | 18.9 | 9.52E-06 | 0.015 | 0.017 | 0.395 | |
| *Holdemania* | | rs1867876 | T | C | 0.294 | 0.084 | 0.016 | 25.7 | 2.74E-07 | -0.024 | 0.019 | 0.205 | |
| *Holdemania* | | rs4146507 | C | T | 0.222 | 0.079 | 0.018 | 19.0 | 7.23E-06 | -0.020 | 0.021 | 0.321 | |
| *Holdemania* | | rs73139538 | G | A | 0.053 | -0.149 | 0.033 | 19.2 | 7.77E-06 | 0.054 | 0.052 | 0.299 | |
| *Holdemania* | | rs77293403 | A | G | 0.046 | 0.165 | 0.034 | 20.6 | 1.77E-06 | 0.010 | 0.048 | 0.840 | |
| *Holdemania* | | rs80149660 | C | T | 0.059 | -0.233 | 0.052 | 52.3 | 6.04E-06 | 0.008 | 0.043 | 0.856 | |
| *Holdemania* | | rs9500080 | C | T | 0.235 | 0.093 | 0.018 | 26.9 | 4.09E-07 | -0.009 | 0.023 | 0.696 | |
| *Holdemania* | | rs9529719 | T | C | 0.325 | 0.074 | 0.016 | 20.9 | 5.97E-06 | -0.010 | 0.019 | 0.603 | |
| *Holdemania* | | rs967319 | T | C | 0.247 | 0.079 | 0.018 | 20.1 | 8.38E-06 | 0.008 | 0.021 | 0.687 | |
| *Howardella* | | rs10048062 | C | T | 0.107 | -0.147 | 0.034 | 15.9 | 8.59E-06 | -0.012 | 0.031 | 0.700 | |
| *Howardella* | | rs12452946 | A | G | 0.501 | -0.106 | 0.023 | 21.4 | 3.80E-06 | -0.011 | 0.018 | 0.519 | |
| *Howardella* | | rs1484873 | A | G | 0.069 | -0.228 | 0.046 | 25.4 | 2.56E-06 | -0.068 | 0.025 | 0.006 | |
| *Howardella* | | rs17167098 | G | A | 0.113 | -0.169 | 0.035 | 22.0 | 1.12E-06 | -0.028 | 0.026 | 0.275 | |
| *Howardella* | | rs2154047 | C | A | 0.068 | -0.193 | 0.042 | 17.8 | 9.97E-06 | -0.029 | 0.031 | 0.358 | |
| *Howardella* | | rs36081916 | T | C | 0.111 | -0.181 | 0.040 | 24.8 | 4.70E-06 | 0.032 | 0.031 | 0.307 | |
| *Howardella* | | rs3791893 | A | G | 0.123 | 0.147 | 0.034 | 17.8 | 9.50E-06 | 0.011 | 0.025 | 0.676 | |
| *Howardella* | | rs609430 | T | G | 0.339 | -0.112 | 0.024 | 21.5 | 3.34E-06 | 0.042 | 0.018 | 0.023 | |
| *Howardella* | | rs672217 | G | A | 0.141 | 0.164 | 0.035 | 25.0 | 3.52E-06 | -0.023 | 0.022 | 0.301 | |
| *Hungatella* | | rs10044993 | C | A | 0.117 | 0.140 | 0.032 | 17.0 | 8.07E-06 | -0.009 | 0.032 | 0.776 | |
| *Hungatella* | | rs13128780 | T | C | 0.145 | -0.150 | 0.031 | 23.5 | 1.75E-06 | -0.001 | 0.023 | 0.969 | |
| *Hungatella* | | rs13249325 | T | G | 0.367 | -0.100 | 0.023 | 19.6 | 9.69E-06 | 0.005 | 0.018 | 0.797 | |
| *Hungatella* | | rs17092615 | G | A | 0.124 | 0.152 | 0.034 | 21.3 | 7.38E-06 | 0.037 | 0.026 | 0.145 | |
| *Hungatella* | | rs72759041 | G | T | 0.235 | -0.126 | 0.028 | 24.1 | 3.86E-06 | 0.027 | 0.022 | 0.216 | |
| *Intestinibacter* | | rs10805326 | G | A | 0.278 | 0.078 | 0.014 | 29.8 | 3.55E-08 | 0.105 | 0.165 | 0.524 | |
| *Intestinibacter* | | rs11109097 | C | T | 0.312 | 0.062 | 0.014 | 20.6 | 5.49E-06 | -0.005 | 0.018 | 0.778 | |
| *Intestinibacter* | | rs118030283 | G | A | 0.038 | -0.152 | 0.032 | 20.7 | 2.67E-06 | 0.009 | 0.042 | 0.840 | |
| *Intestinibacter* | | rs16938435 | T | C | 0.061 | -0.112 | 0.024 | 17.7 | 1.80E-06 | -0.027 | 0.030 | 0.357 | |
| *Intestinibacter* | | rs2098844 | C | T | 0.425 | -0.058 | 0.013 | 19.9 | 6.79E-06 | -0.056 | 0.018 | 0.002 | |
| *Intestinibacter* | | rs2702387 | A | G | 0.352 | 0.061 | 0.013 | 20.8 | 4.26E-06 | 0.015 | 0.018 | 0.405 | |
| *Intestinibacter* | | rs4327025 | G | A | 0.195 | -0.081 | 0.015 | 25.4 | 1.64E-07 | -0.017 | 0.023 | 0.445 | |
| *Intestinibacter* | | rs447950 | A | G | 0.299 | 0.063 | 0.014 | 20.4 | 5.64E-06 | -0.009 | 0.018 | 0.604 | |
| *Intestinibacter* | | rs478972 | T | C | 0.060 | -0.143 | 0.030 | 28.1 | 1.82E-06 | 0.021 | 0.030 | 0.486 | |
| *Intestinibacter* | | rs6062862 | A | G | 0.094 | 0.092 | 0.020 | 18.0 | 6.68E-06 | -0.015 | 0.032 | 0.637 | |
| *Intestinibacter* | | rs62430350 | T | C | 0.058 | 0.151 | 0.035 | 30.6 | 6.84E-06 | -0.150 | 0.047 | 0.002 | |
| *Intestinibacter* | | rs68093214 | C | T | 0.258 | 0.066 | 0.015 | 20.7 | 9.26E-06 | -0.024 | 0.021 | 0.239 | |
| *Intestinibacter* | | rs6875660 | C | T | 0.123 | 0.089 | 0.019 | 21.1 | 3.06E-06 | -0.008 | 0.037 | 0.838 | |
| *Intestinibacter* | | rs893394 | G | A | 0.420 | 0.058 | 0.013 | 20.4 | 7.85E-06 | -0.019 | 0.018 | 0.283 | |
| *Intestinibacter* | | rs9348442 | C | T | 0.088 | 0.099 | 0.022 | 19.3 | 6.26E-06 | 0.011 | 0.027 | 0.677 | |
| *Intestinimonas* | | rs10262702 | T | C | 0.129 | 0.092 | 0.019 | 23.0 | 2.06E-06 | -0.016 | 0.027 | 0.558 | |
| *Intestinimonas* | | rs11258178 | A | G | 0.415 | 0.066 | 0.013 | 25.7 | 6.98E-07 | 0.011 | 0.018 | 0.531 | |
| *Intestinimonas* | | rs12226153 | A | G | 0.062 | -0.151 | 0.031 | 32.0 | 5.12E-07 | 0.029 | 0.075 | 0.699 | |
| *Intestinimonas* | | rs17067892 | C | T | 0.077 | 0.107 | 0.025 | 19.7 | 6.38E-06 | 0.021 | 0.031 | 0.507 | |
| *Intestinimonas* | | rs1859797 | G | A | 0.504 | 0.060 | 0.013 | 22.1 | 4.12E-06 | -0.002 | 0.018 | 0.927 | |
| *Intestinimonas* | | rs2276760 | A | G | 0.220 | -0.069 | 0.015 | 19.5 | 7.84E-06 | -0.004 | 0.021 | 0.858 | |
| *Intestinimonas* | | rs2731794 | C | T | 0.060 | 0.121 | 0.026 | 19.8 | 1.92E-06 | -0.081 | 0.049 | 0.094 | |
| *Intestinimonas* | | rs2930225 | G | T | 0.258 | 0.073 | 0.015 | 24.7 | 1.35E-06 | 0.039 | 0.021 | 0.063 | |
| *Intestinimonas* | | rs4113676 | A | C | 0.062 | -0.219 | 0.049 | 67.1 | 7.42E-06 | -0.070 | 0.074 | 0.343 | |
| *Intestinimonas* | | rs4784055 | T | C | 0.056 | -0.175 | 0.039 | 39.2 | 8.72E-07 | 0.056 | 0.040 | 0.162 | |
| *Intestinimonas* | | rs62240188 | G | A | 0.069 | 0.130 | 0.027 | 26.2 | 2.20E-06 | -0.006 | 0.030 | 0.837 | |
| *Intestinimonas* | | rs6934519 | C | T | 0.275 | 0.069 | 0.015 | 23.2 | 8.57E-06 | 0.011 | 0.020 | 0.588 | |
| *Intestinimonas* | | rs716604 | A | G | 0.188 | 0.082 | 0.017 | 24.7 | 8.57E-07 | -0.012 | 0.021 | 0.580 | |
| *Intestinimonas* | | rs7170984 | T | C | 0.290 | -0.066 | 0.014 | 21.6 | 2.98E-06 | 0.005 | 0.020 | 0.792 | |
| *Intestinimonas* | | rs72982915 | C | T | 0.046 | 0.183 | 0.040 | 35.5 | 4.91E-06 | 0.000 | 0.038 | 0.992 | |
| *Intestinimonas* | | rs9823439 | T | C | 0.441 | -0.058 | 0.013 | 20.2 | 9.86E-06 | -0.034 | 0.018 | 0.054 | |
| *Lachnoclostridium* | | rs1031599 | G | T | 0.085 | -0.079 | 0.018 | 16.7 | 6.31E-06 | -0.031 | 0.036 | 0.386 | |
| *Lachnoclostridium* | | rs12566975 | T | C | 0.493 | -0.047 | 0.011 | 19.1 | 9.57E-06 | 0.002 | 0.018 | 0.912 | |
| *Lachnoclostridium* | | rs1528479 | G | A | 0.370 | -0.050 | 0.011 | 20.1 | 9.64E-06 | 0.037 | 0.018 | 0.039 | |
| *Lachnoclostridium* | | rs1997204 | T | C | 0.053 | -0.108 | 0.024 | 20.3 | 5.97E-06 | 0.047 | 0.042 | 0.260 | |
| *Lachnoclostridium* | | rs2385421 | A | G | 0.104 | 0.075 | 0.018 | 18.1 | 7.14E-06 | 0.034 | 0.027 | 0.211 | |
| *Lachnoclostridium* | | rs3821998 | C | A | 0.089 | -0.086 | 0.019 | 21.0 | 6.72E-06 | -0.013 | 0.029 | 0.651 | |
| *Lachnoclostridium* | | rs4738679 | G | A | 0.365 | -0.052 | 0.011 | 21.8 | 4.42E-06 | -0.006 | 0.018 | 0.756 | |
| *Lachnoclostridium* | | rs6112314 | A | C | 0.397 | -0.056 | 0.011 | 26.3 | 2.43E-07 | 0.043 | 0.019 | 0.022 | |
| *Lachnoclostridium* | | rs615997 | T | C | 0.499 | 0.051 | 0.011 | 22.8 | 2.03E-06 | -0.002 | 0.018 | 0.893 | |
| *Lachnoclostridium* | | rs62285313 | A | G | 0.086 | 0.086 | 0.018 | 20.3 | 1.58E-06 | -0.014 | 0.030 | 0.627 | |
| *Lachnoclostridium* | | rs72829893 | G | T | 0.066 | 0.117 | 0.027 | 29.5 | 5.58E-06 | -0.030 | 0.029 | 0.303 | |
| *Lachnoclostridium* | | rs78068103 | A | G | 0.092 | 0.089 | 0.019 | 22.7 | 3.67E-06 | -0.019 | 0.027 | 0.489 | |
| *Lachnoclostridium* | | rs789029 | C | T | 0.189 | -0.064 | 0.014 | 22.0 | 3.75E-06 | -0.038 | 0.025 | 0.127 | |
| *Lachnospira* | | rs13157098 | A | G | 0.167 | -0.077 | 0.016 | 26.2 | 5.99E-07 | 0.001 | 0.024 | 0.968 | |
| *Lachnospira* | | rs159484 | G | A | 0.113 | 0.079 | 0.018 | 20.3 | 6.68E-06 | -0.008 | 0.034 | 0.803 | |
| *Lachnospira* | | rs2520509 | A | G | 0.318 | 0.052 | 0.012 | 18.7 | 7.42E-06 | 0.001 | 0.019 | 0.949 | |
| *Lachnospira* | | rs4686798 | T | C | 0.386 | 0.053 | 0.011 | 21.4 | 2.74E-06 | 0.003 | 0.018 | 0.866 | |
| *Lachnospira* | | rs4923324 | G | A | 0.239 | -0.062 | 0.013 | 22.1 | 2.44E-06 | -0.003 | 0.024 | 0.902 | |
| *Lachnospira* | | rs56791201 | T | C | 0.457 | 0.052 | 0.011 | 21.3 | 2.93E-06 | -0.021 | 0.018 | 0.245 | |
| *Lachnospiraceae (FCS020 group)* | | rs10093861 | G | A | 0.498 | -0.057 | 0.012 | 22.3 | 3.06E-06 | -0.007 | 0.018 | 0.705 | |
| *Lachnospiraceae (FCS020 group)* | | rs1254846 | G | A | 0.092 | 0.106 | 0.023 | 25.8 | 5.60E-06 | 0.036 | 0.026 | 0.170 | |
| *Lachnospiraceae (FCS020 group)* | | rs1363769 | T | C | 0.033 | -0.201 | 0.045 | 35.3 | 1.58E-06 | -0.026 | 0.050 | 0.608 | |
| *Lachnospiraceae (FCS020 group)* | | rs2322265 | C | T | 0.256 | -0.067 | 0.014 | 23.3 | 5.21E-06 | 0.010 | 0.020 | 0.627 | |
| *Lachnospiraceae (FCS020 group)* | | rs2862811 | T | C | 0.454 | 0.056 | 0.012 | 21.8 | 3.92E-06 | 0.063 | 0.019 | 0.001 | |
| *Lachnospiraceae (FCS020 group)* | | rs35035870 | T | C | 0.036 | -0.191 | 0.041 | 34.6 | 2.62E-06 | -0.001 | 0.045 | 0.976 | |
| *Lachnospiraceae (FCS020 group)* | | rs3999074 | G | T | 0.478 | -0.055 | 0.012 | 20.9 | 6.55E-06 | -0.043 | 0.018 | 0.014 | |
| *Lachnospiraceae (FCS020 group)* | | rs4452603 | T | G | 0.286 | 0.060 | 0.014 | 20.6 | 8.98E-06 | 0.014 | 0.020 | 0.491 | |
| *Lachnospiraceae (FCS020 group)* | | rs7249113 | G | A | 0.281 | 0.068 | 0.013 | 25.8 | 3.72E-07 | -0.006 | 0.019 | 0.747 | |
| *Lachnospiraceae (FCS020 group)* | | rs72793667 | A | G | 0.084 | -0.117 | 0.025 | 28.9 | 1.63E-06 | 0.051 | 0.045 | 0.262 | |
| *Lachnospiraceae (FCS020 group)* | | rs9308097 | A | G | 0.452 | 0.055 | 0.012 | 21.0 | 7.47E-06 | 0.002 | 0.018 | 0.909 | |
| *Lachnospiraceae (FCS020 group)* | | rs9788306 | C | T | 0.284 | -0.063 | 0.013 | 22.1 | 1.39E-06 | 0.004 | 0.019 | 0.835 | |
| *Lachnospiraceae (NC2004 group)* | | rs117467633 | T | C | 0.063 | -0.170 | 0.038 | 17.2 | 9.13E-06 | 0.041 | 0.045 | 0.360 | |
| *Lachnospiraceae (NC2004 group)* | | rs12127733 | G | A | 0.178 | 0.115 | 0.025 | 19.8 | 3.11E-06 | 0.002 | 0.023 | 0.927 | |
| *Lachnospiraceae (NC2004 group)* | | rs12208226 | C | A | 0.106 | -0.155 | 0.034 | 23.2 | 9.75E-06 | 0.006 | 0.028 | 0.838 | |
| *Lachnospiraceae (NC2004 group)* | | rs12863463 | G | A | 0.055 | -0.156 | 0.035 | 12.9 | 6.04E-06 | -0.014 | 0.033 | 0.659 | |
| *Lachnospiraceae (NC2004 group)* | | rs17067076 | G | A | 0.077 | -0.155 | 0.035 | 17.2 | 5.61E-06 | 0.000 | 0.028 | 0.990 | |
| *Lachnospiraceae (NC2004 group)* | | rs1928659 | T | C | 0.250 | 0.103 | 0.023 | 20.1 | 6.17E-06 | 0.023 | 0.022 | 0.290 | |
| *Lachnospiraceae (NC2004 group)* | | rs1929743 | T | C | 0.312 | 0.084 | 0.019 | 15.3 | 9.06E-06 | -0.009 | 0.019 | 0.619 | |
| *Lachnospiraceae (NC2004 group)* | | rs3756315 | A | G | 0.339 | -0.088 | 0.019 | 17.8 | 3.33E-06 | -0.003 | 0.019 | 0.865 | |
| *Lachnospiraceae (NC2004 group)* | | rs6116753 | G | A | 0.240 | 0.099 | 0.021 | 18.4 | 2.92E-06 | 0.005 | 0.023 | 0.818 | |
| *Lachnospiraceae (ND3007 group)* | | rs2861203 | G | A | 0.319 | 0.057 | 0.013 | 21.8 | 7.37E-06 | 0.009 | 0.019 | 0.646 | |
| *Lachnospiraceae (ND3007 group)* | | rs72776675 | T | C | 0.175 | -0.065 | 0.015 | 18.5 | 8.72E-06 | 0.008 | 0.023 | 0.733 | |
| *Lachnospiraceae (ND3007 group)* | | rs9932954 | A | G | 0.428 | -0.056 | 0.012 | 23.7 | 1.25E-06 | 0.015 | 0.019 | 0.421 | |
| *Lachnospiraceae (NK4A136 group)* | | rs10952110 | G | T | 0.448 | 0.049 | 0.011 | 19.7 | 9.08E-06 | -0.011 | 0.011 | 0.552 | |
| *Lachnospiraceae (NK4A136 group)* | | rs11263806 | A | G | 0.278 | -0.052 | 0.012 | 18.6 | 5.07E-06 | 0.003 | 0.012 | 0.884 | |
| *Lachnospiraceae (NK4A136 group)* | | rs12611395 | A | G | 0.090 | -0.090 | 0.020 | 22.3 | 5.83E-06 | -0.014 | 0.020 | 0.629 | |
| *Lachnospiraceae (NK4A136 group)* | | rs160061 | A | G | 0.468 | 0.051 | 0.011 | 22.1 | 2.12E-06 | -0.013 | 0.011 | 0.472 | |
| *Lachnospiraceae (NK4A136 group)* | | rs28540839 | A | C | 0.487 | 0.051 | 0.011 | 21.7 | 9.34E-06 | 0.017 | 0.011 | 0.326 | |
| *Lachnospiraceae (NK4A136 group)* | | rs2880566 | T | C | 0.188 | 0.060 | 0.013 | 18.4 | 5.61E-06 | -0.026 | 0.013 | 0.288 | |
| *Lachnospiraceae (NK4A136 group)* | | rs4955932 | T | C | 0.396 | -0.049 | 0.011 | 19.4 | 7.05E-06 | -0.024 | 0.011 | 0.193 | |
| *Lachnospiraceae (NK4A136 group)* | | rs59805249 | T | C | 0.091 | 0.094 | 0.021 | 24.2 | 9.45E-06 | 0.013 | 0.021 | 0.673 | |
| *Lachnospiraceae (NK4A136 group)* | | rs68104925 | T | C | 0.371 | -0.055 | 0.012 | 23.6 | 2.37E-06 | 0.021 | 0.012 | 0.278 | |
| *Lachnospiraceae (NK4A136 group)* | | rs7073658 | T | G | 0.405 | -0.050 | 0.011 | 20.2 | 5.27E-06 | -0.016 | 0.011 | 0.372 | |
| *Lachnospiraceae (NK4A136 group)* | | rs73044693 | A | G | 0.067 | -0.108 | 0.023 | 24.2 | 3.57E-06 | -0.009 | 0.023 | 0.798 | |
| *Lachnospiraceae (NK4A136 group)* | | rs7616165 | G | T | 0.037 | -0.231 | 0.048 | 63.4 | 2.77E-06 | 0.090 | 0.048 | 0.103 | |
| *Lachnospiraceae (NK4A136 group)* | | rs76193507 | A | G | 0.057 | -0.230 | 0.050 | 95.2 | 2.93E-06 | 0.010 | 0.050 | 0.762 | |
| *Lachnospiraceae (NK4A136 group)* | | rs7832116 | A | G | 0.146 | -0.071 | 0.015 | 21.4 | 3.57E-06 | -0.015 | 0.015 | 0.565 | |
| *Lachnospiraceae (NK4A136 group)* | | rs954878 | A | G | 0.450 | -0.052 | 0.011 | 22.5 | 1.78E-06 | 0.007 | 0.011 | 0.693 | |
| *Lachnospiraceae (UCG001)* | | rs12131224 | C | T | 0.116 | 0.117 | 0.026 | 28.7 | 7.40E-06 | -0.046 | 0.028 | 0.103 | |
| *Lachnospiraceae (UCG001)* | | rs2050911 | G | A | 0.304 | 0.075 | 0.015 | 24.3 | 1.11E-06 | -0.027 | 0.019 | 0.138 | |
| *Lachnospiraceae (UCG001)* | | rs2371284 | T | C | 0.223 | -0.076 | 0.017 | 20.4 | 7.56E-06 | 0.003 | 0.021 | 0.894 | |
| *Lachnospiraceae (UCG001)* | | rs437876 | T | C | 0.373 | 0.078 | 0.014 | 29.3 | 7.17E-08 | 0.020 | 0.018 | 0.280 | |
| *Lachnospiraceae (UCG001)* | | rs4981345 | T | C | 0.351 | -0.068 | 0.015 | 21.5 | 6.09E-06 | -0.021 | 0.019 | 0.261 | |
| *Lachnospiraceae (UCG001)* | | rs573933 | T | C | 0.102 | -0.108 | 0.023 | 21.8 | 3.11E-06 | 0.023 | 0.029 | 0.429 | |
| *Lachnospiraceae (UCG001)* | | rs62496417 | T | G | 0.271 | -0.075 | 0.017 | 22.5 | 5.88E-06 | 0.044 | 0.021 | 0.041 | |
| *Lachnospiraceae (UCG001)* | | rs7341608 | T | C | 0.190 | -0.078 | 0.018 | 19.3 | 9.48E-06 | -0.009 | 0.026 | 0.720 | |
| *Lachnospiraceae (UCG001)* | | rs74034332 | G | A | 0.046 | 0.168 | 0.038 | 25.1 | 3.33E-06 | 0.063 | 0.036 | 0.085 | |
| *Lachnospiraceae (UCG001)* | | rs78848836 | A | G | 0.084 | -0.119 | 0.026 | 22.0 | 3.38E-06 | -0.044 | 0.029 | 0.131 | |
| *Lachnospiraceae (UCG001)* | | rs8104225 | A | G | 0.149 | 0.089 | 0.020 | 20.5 | 8.04E-06 | -0.014 | 0.021 | 0.516 | |
| *Lachnospiraceae (UCG001)* | | rs9403580 | C | T | 0.109 | 0.108 | 0.023 | 23.0 | 3.47E-06 | -0.007 | 0.026 | 0.784 | |
| *Lachnospiraceae (UCG001)* | | rs985416 | C | T | 0.206 | 0.097 | 0.018 | 31.3 | 1.46E-07 | -0.008 | 0.023 | 0.716 | |
| *Lachnospiraceae (UCG004)* | | rs11128180 | A | G | 0.224 | 0.065 | 0.014 | 21.1 | 4.52E-06 | 0.011 | 0.021 | 0.610 | |
| *Lachnospiraceae (UCG004)* | | rs12072562 | T | C | 0.052 | 0.133 | 0.030 | 25.2 | 7.07E-06 | -0.031 | 0.046 | 0.496 | |
| *Lachnospiraceae (UCG004)* | | rs12673420 | G | A | 0.441 | 0.055 | 0.012 | 22.0 | 2.98E-06 | -0.001 | 0.018 | 0.966 | |
| *Lachnospiraceae (UCG004)* | | rs12747809 | G | A | 0.340 | -0.062 | 0.013 | 25.2 | 8.65E-07 | 0.006 | 0.020 | 0.779 | |
| *Lachnospiraceae (UCG004)* | | rs12894272 | A | G | 0.300 | 0.058 | 0.013 | 20.5 | 4.34E-06 | 0.044 | 0.019 | 0.017 | |
| *Lachnospiraceae (UCG004)* | | rs233486 | A | G | 0.124 | -0.080 | 0.018 | 20.1 | 6.28E-06 | 0.025 | 0.025 | 0.323 | |
| *Lachnospiraceae (UCG004)* | | rs2444793 | C | T | 0.470 | -0.054 | 0.012 | 21.2 | 4.77E-06 | -0.029 | 0.018 | 0.109 | |
| *Lachnospiraceae (UCG004)* | | rs2726805 | A | G | 0.409 | 0.055 | 0.012 | 21.1 | 6.30E-06 | -0.009 | 0.018 | 0.624 | |
| *Lachnospiraceae (UCG004)* | | rs2882478 | G | A | 0.455 | -0.058 | 0.012 | 23.9 | 1.21E-06 | 0.016 | 0.018 | 0.369 | |
| *Lachnospiraceae (UCG004)* | | rs35182105 | A | G | 0.073 | -0.110 | 0.024 | 23.5 | 4.87E-06 | -0.021 | 0.039 | 0.596 | |
| *Lachnospiraceae (UCG004)* | | rs6656451 | C | T | 0.439 | -0.054 | 0.012 | 21.1 | 5.57E-06 | -0.005 | 0.018 | 0.794 | |
| *Lachnospiraceae (UCG004)* | | rs7629954 | A | G | 0.069 | 0.108 | 0.024 | 21.8 | 5.77E-06 | 0.018 | 0.043 | 0.670 | |
| *Lachnospiraceae (UCG008)* | | rs10741777 | T | C | 0.261 | -0.097 | 0.019 | 25.8 | 7.69E-07 | 0.021 | 0.019 | 0.282 | |
| *Lachnospiraceae (UCG008)* | | rs10793103 | C | T | 0.304 | 0.097 | 0.018 | 28.3 | 9.35E-08 | 0.030 | 0.018 | 0.092 | |
| *Lachnospiraceae (UCG008)* | | rs10801803 | G | A | 0.162 | -0.117 | 0.024 | 26.2 | 1.40E-06 | 0.011 | 0.025 | 0.661 | |
| *Lachnospiraceae (UCG008)* | | rs13024781 | T | C | 0.503 | -0.080 | 0.017 | 22.4 | 2.29E-06 | 0.016 | 0.018 | 0.363 | |
| *Lachnospiraceae (UCG008)* | | rs57091572 | A | G | 0.140 | -0.110 | 0.024 | 20.7 | 2.86E-06 | 0.000 | 0.026 | 0.992 | |
| *Lachnospiraceae (UCG008)* | | rs57254474 | G | A | 0.241 | 0.089 | 0.020 | 20.2 | 6.92E-06 | -0.003 | 0.022 | 0.874 | |
| *Lachnospiraceae (UCG008)* | | rs61944774 | A | G | 0.058 | 0.180 | 0.039 | 24.7 | 6.34E-06 | -0.019 | 0.039 | 0.624 | |
| *Lachnospiraceae (UCG008)* | | rs62277846 | C | T | 0.236 | 0.102 | 0.021 | 26.5 | 1.59E-06 | 0.018 | 0.022 | 0.433 | |
| *Lachnospiraceae (UCG008)* | | rs67078837 | T | C | 0.458 | -0.085 | 0.017 | 25.0 | 7.68E-07 | -0.016 | 0.018 | 0.370 | |
| *Lachnospiraceae (UCG008)* | | rs75356640 | G | A | 0.089 | 0.137 | 0.030 | 21.1 | 9.83E-06 | 0.003 | 0.027 | 0.903 | |
| *Lachnospiraceae (UCG008)* | | rs955844 | A | C | 0.200 | 0.112 | 0.023 | 28.3 | 1.81E-06 | -0.006 | 0.026 | 0.830 | |
| *Lachnospiraceae (UCG010)* | | rs10414815 | T | C | 0.119 | 0.105 | 0.023 | 26.6 | 4.24E-06 | 0.048 | 0.042 | 0.257 | |
| *Lachnospiraceae (UCG010)* | | rs11192447 | A | G | 0.075 | 0.127 | 0.024 | 25.6 | 4.69E-07 | 0.100 | 0.040 | 0.013 | |
| *Lachnospiraceae (UCG010)* | | rs12346653 | C | T | 0.274 | 0.066 | 0.014 | 19.9 | 2.70E-06 | -0.028 | 0.022 | 0.193 | |
| *Lachnospiraceae (UCG010)* | | rs17730011 | G | A | 0.219 | -0.070 | 0.016 | 19.5 | 7.85E-06 | -0.007 | 0.021 | 0.757 | |
| *Lachnospiraceae (UCG010)* | | rs2833528 | C | T | 0.361 | -0.056 | 0.013 | 16.9 | 9.92E-06 | -0.031 | 0.018 | 0.091 | |
| *Lachnospiraceae (UCG010)* | | rs336138 | G | T | 0.163 | 0.078 | 0.017 | 19.2 | 7.48E-06 | -0.026 | 0.028 | 0.338 | |
| *Lachnospiraceae (UCG010)* | | rs4576377 | A | C | 0.384 | -0.057 | 0.013 | 17.9 | 7.63E-06 | -0.005 | 0.018 | 0.775 | |
| *Lachnospiraceae (UCG010)* | | rs72894957 | G | A | 0.048 | 0.222 | 0.049 | 52.1 | 5.68E-06 | -0.066 | 0.059 | 0.263 | |
| *Lachnospiraceae (UCG010)* | | rs74315802 | G | T | 0.121 | 0.087 | 0.018 | 18.6 | 3.19E-06 | -0.022 | 0.023 | 0.332 | |
| *Lachnospiraceae (UCG010)* | | rs9981767 | A | C | 0.316 | 0.066 | 0.013 | 21.5 | 9.96E-07 | 0.007 | 0.020 | 0.735 | |
| *Lactobacillus* | | rs12693845 | C | T | 0.357 | -0.081 | 0.018 | 20.8 | 8.96E-06 | 0.019 | 0.018 | 0.303 | |
| *Lactobacillus* | | rs1530559 | G | A | 0.568 | 0.080 | 0.018 | 22.1 | 4.93E-06 | -0.042 | 0.018 | 0.018 | |
| *Lactobacillus* | | rs16861661 | G | A | 0.050 | -0.183 | 0.038 | 22.1 | 1.28E-06 | -0.048 | 0.036 | 0.178 | |
| *Lactobacillus* | | rs62314653 | C | A | 0.063 | 0.188 | 0.039 | 28.9 | 2.24E-06 | 0.028 | 0.037 | 0.454 | |
| *Lactobacillus* | | rs7399658 | G | A | 0.216 | -0.107 | 0.022 | 27.1 | 3.12E-06 | 0.002 | 0.023 | 0.927 | |
| *Lactobacillus* | | rs75127669 | C | A | 0.071 | 0.140 | 0.031 | 17.9 | 6.83E-06 | -0.016 | 0.034 | 0.647 | |
| *Lactobacillus* | | rs768253 | T | G | 0.391 | -0.079 | 0.017 | 20.8 | 4.25E-06 | -0.034 | 0.018 | 0.057 | |
| *Lactobacillus* | | rs77478751 | A | G | 0.054 | -0.220 | 0.048 | 34.3 | 7.33E-06 | -0.022 | 0.028 | 0.416 | |
| *Lactobacillus* | | rs921925 | A | C | 0.262 | 0.099 | 0.020 | 26.2 | 9.72E-07 | -0.005 | 0.021 | 0.801 | |
| *Lactococcus* | | rs10417872 | T | G | 0.258 | 0.118 | 0.025 | 21.9 | 1.29E-06 | -0.007 | 0.019 | 0.731 | |
| *Lactococcus* | | rs123059 | T | C | 0.222 | -0.137 | 0.027 | 26.3 | 1.27E-06 | 0.012 | 0.021 | 0.564 | |
| *Lactococcus* | | rs12621813 | G | A | 0.282 | 0.108 | 0.024 | 19.4 | 6.61E-06 | -0.002 | 0.020 | 0.923 | |
| *Lactococcus* | | rs17168302 | G | A | 0.066 | 0.192 | 0.042 | 18.4 | 6.29E-06 | -0.017 | 0.029 | 0.547 | |
| *Lactococcus* | | rs2293361 | C | T | 0.065 | -0.199 | 0.043 | 19.5 | 1.40E-06 | 0.055 | 0.039 | 0.162 | |
| *Lactococcus* | | rs4766997 | C | T | 0.296 | 0.115 | 0.024 | 22.3 | 2.06E-06 | -0.014 | 0.018 | 0.419 | |
| *Lactococcus* | | rs55910161 | C | T | 0.135 | 0.146 | 0.031 | 20.4 | 2.36E-06 | -0.011 | 0.028 | 0.686 | |
| *Lactococcus* | | rs6674304 | C | T | 0.069 | 0.201 | 0.044 | 21.0 | 6.18E-06 | -0.013 | 0.046 | 0.778 | |
| *Lactococcus* | | rs7992246 | T | C | 0.347 | 0.104 | 0.023 | 20.1 | 4.45E-06 | -0.007 | 0.018 | 0.689 | |
| *Marvinbryantia* | | rs11620597 | T | C | 0.053 | 0.119 | 0.027 | 15.7 | 7.80E-06 | -0.069 | 0.057 | 0.224 | |
| *Marvinbryantia* | | rs1187983 | C | T | 0.122 | -0.094 | 0.019 | 20.8 | 2.02E-06 | -0.019 | 0.029 | 0.521 | |
| *Marvinbryantia* | | rs146541147 | G | A | 0.071 | 0.119 | 0.027 | 20.5 | 6.86E-06 | -0.060 | 0.049 | 0.223 | |
| *Marvinbryantia* | | rs2724813 | A | G | 0.201 | -0.084 | 0.017 | 25.1 | 6.28E-07 | -0.008 | 0.021 | 0.708 | |
| *Marvinbryantia* | | rs2842896 | C | T | 0.528 | -0.065 | 0.013 | 23.2 | 7.25E-07 | 0.010 | 0.018 | 0.579 | |
| *Marvinbryantia* | | rs2863363 | A | G | 0.368 | 0.063 | 0.014 | 20.7 | 3.11E-06 | 0.056 | 0.020 | 0.006 | |
| *Marvinbryantia* | | rs3125832 | A | C | 0.262 | 0.068 | 0.015 | 19.7 | 5.03E-06 | -0.054 | 0.021 | 0.011 | |
| *Marvinbryantia* | | rs61884471 | G | A | 0.085 | 0.124 | 0.025 | 26.5 | 1.01E-06 | 0.034 | 0.028 | 0.226 | |
| *Marvinbryantia* | | rs72948274 | A | C | 0.058 | -0.126 | 0.027 | 19.2 | 3.26E-06 | -0.037 | 0.036 | 0.302 | |
| *Marvinbryantia* | | rs8006832 | G | T | 0.111 | -0.095 | 0.022 | 19.8 | 6.58E-06 | -0.001 | 0.031 | 0.979 | |
| *Methanobrevibacter* | | rs10202904 | T | G | 0.467 | -0.113 | 0.024 | 22.8 | 3.09E-06 | -0.005 | 0.018 | 0.802 | |
| *Methanobrevibacter* | | rs1334944 | T | C | 0.285 | 0.115 | 0.026 | 19.5 | 7.61E-06 | -0.012 | 0.020 | 0.529 | |
| *Methanobrevibacter* | | rs4802933 | A | G | 0.178 | -0.136 | 0.031 | 19.4 | 9.74E-06 | -0.013 | 0.021 | 0.524 | |
| *Methanobrevibacter* | | rs6776814 | T | C | 0.090 | -0.189 | 0.042 | 21.0 | 8.05E-06 | -0.015 | 0.062 | 0.803 | |
| *Methanobrevibacter* | | rs76029318 | T | C | 0.065 | 0.223 | 0.045 | 21.6 | 1.08E-06 | 0.000 | 0.037 | 0.997 | |
| *Methanobrevibacter* | | rs894996 | C | A | 0.072 | 0.214 | 0.046 | 22.0 | 3.82E-06 | 0.040 | 0.035 | 0.250 | |
| *Odoribacter* | | rs10093869 | A | G | 0.376 | -0.058 | 0.013 | 22.3 | 3.67E-06 | -0.021 | 0.018 | 0.234 | |
| *Odoribacter* | | rs10423795 | C | T | 0.407 | 0.055 | 0.012 | 20.8 | 6.58E-06 | 0.044 | 0.018 | 0.014 | |
| *Odoribacter* | | rs28417404 | A | G | 0.169 | -0.073 | 0.016 | 21.1 | 3.68E-06 | -0.053 | 0.030 | 0.075 | |
| *Odoribacter* | | rs4793970 | A | G | 0.301 | -0.058 | 0.013 | 19.9 | 6.03E-06 | 0.014 | 0.018 | 0.464 | |
| *Odoribacter* | | rs6856150 | G | A | 0.118 | 0.088 | 0.019 | 23.1 | 6.06E-06 | 0.019 | 0.027 | 0.490 | |
| *Odoribacter* | | rs74553962 | T | G | 0.063 | 0.121 | 0.026 | 24.7 | 9.49E-06 | -0.042 | 0.034 | 0.213 | |
| *Odoribacter* | | rs77779484 | G | A | 0.059 | -0.133 | 0.027 | 28.0 | 6.56E-07 | 0.003 | 0.037 | 0.926 | |
| *Olsenella* | | rs1035588 | A | G | 0.383 | -0.108 | 0.024 | 20.8 | 4.86E-06 | -0.042 | 0.018 | 0.022 | |
| *Olsenella* | | rs17148768 | G | A | 0.212 | 0.140 | 0.030 | 24.2 | 2.20E-06 | 0.001 | 0.024 | 0.964 | |
| *Olsenella* | | rs2759329 | G | A | 0.340 | -0.111 | 0.024 | 20.8 | 3.43E-06 | 0.011 | 0.018 | 0.553 | |
| *Olsenella* | | rs35225860 | A | G | 0.057 | -0.224 | 0.048 | 19.5 | 3.87E-06 | -0.035 | 0.045 | 0.437 | |
| *Olsenella* | | rs61090148 | A | G | 0.423 | -0.105 | 0.023 | 20.1 | 6.44E-06 | -0.016 | 0.018 | 0.382 | |
| *Olsenella* | | rs62112538 | C | T | 0.081 | -0.199 | 0.041 | 21.5 | 1.19E-06 | -0.054 | 0.028 | 0.054 | |
| *Olsenella* | | rs72691585 | C | A | 0.069 | -0.249 | 0.052 | 24.6 | 2.95E-06 | 0.002 | 0.026 | 0.932 | |
| *Olsenella* | | rs7540303 | C | T | 0.422 | 0.108 | 0.024 | 21.4 | 5.32E-06 | 0.011 | 0.018 | 0.539 | |
| *Olsenella* | | rs8066522 | G | A | 0.347 | -0.107 | 0.024 | 19.3 | 9.70E-06 | -0.013 | 0.019 | 0.496 | |
| *Olsenella* | | rs9460691 | C | A | 0.217 | 0.120 | 0.027 | 18.3 | 7.28E-06 | 0.012 | 0.022 | 0.602 | |
| *Oscillibacter* | | rs11627628 | T | C | 0.073 | 0.144 | 0.029 | 23.7 | 1.01E-06 | -0.059 | 0.033 | 0.074 | |
| *Oscillibacter* | | rs11990279 | T | C | 0.240 | -0.082 | 0.018 | 21.1 | 4.94E-06 | -0.031 | 0.025 | 0.216 | |
| *Oscillibacter* | | rs12649930 | T | G | 0.094 | 0.122 | 0.026 | 21.5 | 4.09E-06 | 0.081 | 0.029 | 0.005 | |
| *Oscillibacter* | | rs133832 | A | C | 0.313 | -0.080 | 0.016 | 23.2 | 1.15E-06 | -0.028 | 0.020 | 0.160 | |
| *Oscillibacter* | | rs16866406 | A | G | 0.139 | 0.099 | 0.021 | 19.9 | 3.08E-06 | -0.003 | 0.024 | 0.910 | |
| *Oscillibacter* | | rs16934185 | A | G | 0.104 | -0.130 | 0.028 | 26.7 | 4.38E-06 | -0.042 | 0.029 | 0.149 | |
| *Oscillibacter* | | rs234108 | A | G | 0.480 | 0.075 | 0.015 | 23.9 | 9.16E-07 | -0.014 | 0.018 | 0.420 | |
| *Oscillibacter* | | rs36095275 | C | T | 0.362 | -0.075 | 0.016 | 22.2 | 1.40E-06 | -0.028 | 0.018 | 0.117 | |
| *Oscillibacter* | | rs4506202 | A | G | 0.516 | -0.071 | 0.015 | 21.5 | 3.21E-06 | -0.032 | 0.018 | 0.069 | |
| *Oscillibacter* | | rs61883564 | A | G | 0.124 | -0.101 | 0.022 | 19.0 | 3.39E-06 | 0.025 | 0.026 | 0.334 | |
| *Oscillibacter* | | rs75453768 | G | T | 0.087 | 0.122 | 0.027 | 20.0 | 5.35E-06 | 0.004 | 0.030 | 0.892 | |
| *Oscillibacter* | | rs761240 | T | G | 0.067 | -0.177 | 0.039 | 33.0 | 2.04E-06 | 0.027 | 0.041 | 0.508 | |
| *Oscillibacter* | | rs9393920 | A | G | 0.435 | -0.074 | 0.015 | 23.2 | 9.92E-07 | -0.013 | 0.019 | 0.480 | |
| *Oscillospira* | | rs12206468 | G | A | 0.064 | -0.133 | 0.027 | 20.2 | 1.04E-06 | -0.004 | 0.033 | 0.909 | |
| *Oscillospira* | | rs12925026 | T | C | 0.073 | 0.136 | 0.031 | 20.3 | 9.31E-06 | 0.056 | 0.038 | 0.140 | |
| *Oscillospira* | | rs1954532 | T | C | 0.209 | -0.083 | 0.018 | 22.2 | 2.27E-06 | -0.012 | 0.022 | 0.579 | |
| *Oscillospira* | | rs28889936 | A | C | 0.108 | 0.114 | 0.025 | 24.6 | 3.37E-06 | 0.014 | 0.030 | 0.632 | |
| *Oscillospira* | | rs62422654 | C | T | 0.147 | 0.090 | 0.020 | 19.8 | 6.47E-06 | 0.039 | 0.022 | 0.072 | |
| *Oscillospira* | | rs72866977 | A | C | 0.070 | -0.131 | 0.028 | 20.1 | 5.63E-06 | 0.006 | 0.033 | 0.852 | |
| *Oscillospira* | | rs751183 | T | C | 0.246 | -0.077 | 0.017 | 21.9 | 6.85E-06 | -0.025 | 0.023 | 0.268 | |
| *Oscillospira* | | rs8076323 | A | G | 0.258 | 0.072 | 0.016 | 20.0 | 5.61E-06 | 0.013 | 0.019 | 0.487 | |
| *Oxalobacter* | | rs10464997 | G | A | 0.153 | 0.138 | 0.029 | 23.0 | 3.30E-06 | -0.013 | 0.023 | 0.553 | |
| *Oxalobacter* | | rs11108500 | A | G | 0.077 | -0.199 | 0.043 | 26.2 | 3.74E-06 | 0.016 | 0.031 | 0.618 | |
| *Oxalobacter* | | rs111966731 | T | C | 0.072 | 0.213 | 0.047 | 28.2 | 7.30E-06 | -0.025 | 0.031 | 0.428 | |
| *Oxalobacter* | | rs12002250 | A | C | 0.060 | 0.217 | 0.047 | 24.7 | 1.42E-06 | 0.064 | 0.044 | 0.143 | |
| *Oxalobacter* | | rs1569853 | T | C | 0.138 | -0.138 | 0.030 | 21.2 | 3.65E-06 | -0.048 | 0.027 | 0.077 | |
| *Oxalobacter* | | rs36057338 | G | T | 0.076 | 0.208 | 0.042 | 28.2 | 8.80E-07 | -0.046 | 0.049 | 0.356 | |
| *Oxalobacter* | | rs3862635 | C | T | 0.079 | -0.172 | 0.039 | 20.0 | 9.19E-06 | 0.021 | 0.030 | 0.488 | |
| *Oxalobacter* | | rs4428215 | G | A | 0.260 | 0.130 | 0.024 | 30.6 | 7.51E-08 | 0.014 | 0.020 | 0.494 | |
| *Oxalobacter* | | rs6000536 | C | T | 0.211 | -0.131 | 0.025 | 26.7 | 2.06E-07 | 0.012 | 0.023 | 0.620 | |
| *Oxalobacter* | | rs6993398 | G | A | 0.153 | 0.127 | 0.028 | 19.6 | 7.13E-06 | 0.031 | 0.023 | 0.172 | |
| *Oxalobacter* | | rs736744 | C | T | 0.416 | 0.118 | 0.021 | 31.6 | 2.57E-08 | -0.003 | 0.018 | 0.885 | |
| *Parabacteroides* | | rs115602804 | G | A | 0.067 | 0.103 | 0.022 | 21.7 | 1.93E-06 | 0.022 | 0.029 | 0.448 | |
| *Parabacteroides* | | rs4236095 | G | A | 0.125 | 0.076 | 0.016 | 20.9 | 1.93E-06 | 0.019 | 0.029 | 0.506 | |
| *Parabacteroides* | | rs60884758 | C | T | 0.165 | -0.070 | 0.014 | 22.3 | 5.71E-07 | -0.016 | 0.023 | 0.479 | |
| *Parabacteroides* | | rs6657302 | T | C | 0.052 | -0.105 | 0.023 | 17.6 | 9.76E-06 | 0.064 | 0.036 | 0.076 | |
| *Parabacteroides* | | rs7298818 | C | T | 0.077 | 0.089 | 0.020 | 18.3 | 8.54E-06 | -0.010 | 0.029 | 0.744 | |
| *Paraprevotella* | | rs10842464 | T | C | 0.419 | -0.076 | 0.017 | 20.1 | 6.60E-06 | -0.009 | 0.019 | 0.654 | |
| *Paraprevotella* | | rs140997932 | T | C | 0.065 | -0.162 | 0.035 | 22.9 | 2.11E-06 | 0.033 | 0.039 | 0.394 | |
| *Paraprevotella* | | rs145020347 | A | G | 0.139 | -0.125 | 0.026 | 26.8 | 4.03E-06 | -0.020 | 0.025 | 0.417 | |
| *Paraprevotella* | | rs17109926 | A | G | 0.188 | -0.099 | 0.022 | 21.5 | 6.75E-06 | 0.056 | 0.020 | 0.004 | |
| *Paraprevotella* | | rs17785622 | A | G | 0.060 | 0.248 | 0.052 | 49.8 | 1.93E-06 | 0.013 | 0.046 | 0.782 | |
| *Paraprevotella* | | rs2081023 | A | G | 0.102 | -0.123 | 0.024 | 19.9 | 2.64E-07 | 0.020 | 0.025 | 0.437 | |
| *Paraprevotella* | | rs3008582 | T | C | 0.132 | 0.106 | 0.023 | 18.4 | 4.36E-06 | -0.032 | 0.022 | 0.154 | |
| *Paraprevotella* | | rs3801748 | G | A | 0.346 | 0.078 | 0.017 | 19.8 | 5.20E-06 | -0.010 | 0.018 | 0.583 | |
| *Paraprevotella* | | rs4756632 | G | T | 0.093 | -0.139 | 0.029 | 23.5 | 3.82E-06 | -0.001 | 0.026 | 0.966 | |
| *Paraprevotella* | | rs4767113 | C | T | 0.287 | 0.088 | 0.018 | 23.0 | 2.14E-06 | -0.014 | 0.019 | 0.446 | |
| *Paraprevotella* | | rs7240324 | T | G | 0.191 | -0.102 | 0.023 | 23.3 | 5.96E-06 | -0.014 | 0.020 | 0.492 | |
| *Paraprevotella* | | rs9602779 | A | C | 0.173 | -0.107 | 0.022 | 23.5 | 6.93E-07 | 0.001 | 0.021 | 0.948 | |
| *Paraprevotella* | | rs9900242 | A | G | 0.361 | -0.085 | 0.018 | 24.2 | 1.14E-06 | 0.028 | 0.018 | 0.131 | |
| *Parasutterella* | | rs10899911 | A | G | 0.272 | -0.072 | 0.015 | 23.3 | 1.15E-06 | -0.004 | 0.021 | 0.839 | |
| *Parasutterella* | | rs11715853 | G | A | 0.314 | -0.066 | 0.015 | 21.6 | 6.23E-06 | -0.019 | 0.019 | 0.325 | |
| *Parasutterella* | | rs2090816 | A | C | 0.157 | 0.084 | 0.018 | 21.4 | 2.90E-06 | 0.047 | 0.023 | 0.042 | |
| *Parasutterella* | | rs35055552 | T | C | 0.078 | 0.110 | 0.024 | 19.6 | 3.35E-06 | 0.043 | 0.026 | 0.090 | |
| *Parasutterella* | | rs55877868 | A | C | 0.096 | -0.104 | 0.023 | 21.7 | 2.87E-06 | 0.020 | 0.029 | 0.493 | |
| *Parasutterella* | | rs62273907 | A | G | 0.050 | 0.229 | 0.050 | 56.9 | 5.88E-06 | 0.006 | 0.035 | 0.861 | |
| *Parasutterella* | | rs6809952 | G | A | 0.273 | -0.068 | 0.015 | 21.3 | 8.13E-06 | 0.015 | 0.020 | 0.457 | |
| *Parasutterella* | | rs6828768 | C | T | 0.500 | 0.064 | 0.013 | 23.1 | 1.78E-06 | 0.007 | 0.018 | 0.674 | |
| *Parasutterella* | | rs7303158 | C | T | 0.442 | 0.065 | 0.013 | 23.5 | 1.33E-06 | 0.004 | 0.018 | 0.816 | |
| *Parasutterella* | | rs7311004 | T | C | 0.376 | -0.062 | 0.014 | 20.4 | 5.92E-06 | 0.016 | 0.018 | 0.374 | |
| *Parasutterella* | | rs7572229 | G | A | 0.490 | 0.066 | 0.013 | 25.0 | 6.32E-07 | 0.007 | 0.018 | 0.687 | |
| *Parasutterella* | | rs78383039 | T | C | 0.067 | -0.146 | 0.030 | 30.4 | 1.57E-06 | 0.022 | 0.044 | 0.617 | |
| *Parasutterella* | | rs8039785 | T | G | 0.452 | 0.062 | 0.013 | 21.6 | 3.62E-06 | -0.016 | 0.018 | 0.377 | |
| *Parasutterella* | | rs823424 | G | A | 0.210 | -0.071 | 0.016 | 19.2 | 4.95E-06 | 0.007 | 0.020 | 0.739 | |
| *Peptococcus* | | rs10031059 | T | C | 0.221 | -0.121 | 0.023 | 28.0 | 1.24E-07 | 0.050 | 0.021 | 0.015 | |
| *Peptococcus* | | rs11001941 | G | A | 0.068 | -0.196 | 0.039 | 26.8 | 1.33E-06 | 0.028 | 0.031 | 0.363 | |
| *Peptococcus* | | rs12069354 | C | T | 0.067 | 0.168 | 0.038 | 19.4 | 9.28E-06 | -0.011 | 0.037 | 0.765 | |
| *Peptococcus* | | rs2054133 | G | A | 0.474 | 0.090 | 0.019 | 22.2 | 2.14E-06 | 0.017 | 0.019 | 0.371 | |
| *Peptococcus* | | rs36121075 | A | G | 0.128 | -0.141 | 0.031 | 24.5 | 6.99E-06 | -0.062 | 0.023 | 0.008 | |
| *Peptococcus* | | rs413827 | G | A | 0.213 | 0.110 | 0.024 | 22.6 | 3.30E-06 | -0.024 | 0.021 | 0.245 | |
| *Peptococcus* | | rs5770862 | T | C | 0.067 | 0.162 | 0.036 | 18.1 | 3.22E-06 | 0.019 | 0.030 | 0.519 | |
| *Peptococcus* | | rs6918730 | G | A | 0.097 | 0.135 | 0.029 | 17.8 | 1.15E-06 | -0.061 | 0.037 | 0.099 | |
| *Peptococcus* | | rs7033353 | T | G | 0.420 | 0.090 | 0.019 | 22.0 | 2.22E-06 | 0.022 | 0.018 | 0.216 | |
| *Peptococcus* | | rs72850165 | T | C | 0.108 | -0.134 | 0.030 | 19.3 | 5.74E-06 | 0.016 | 0.033 | 0.620 | |
| *Peptococcus* | | rs74592222 | G | A | 0.117 | 0.138 | 0.030 | 21.9 | 8.55E-06 | 0.011 | 0.028 | 0.687 | |
| *Peptococcus* | | rs77681628 | C | T | 0.059 | 0.200 | 0.039 | 24.6 | 2.69E-07 | -0.024 | 0.032 | 0.464 | |
| *Phascolarctobacterium* | | rs11929846 | T | C | 0.210 | -0.070 | 0.016 | 17.9 | 8.88E-06 | -0.002 | 0.021 | 0.927 | |
| *Phascolarctobacterium* | | rs12618201 | A | G | 0.347 | 0.064 | 0.014 | 20.8 | 3.38E-06 | -0.019 | 0.018 | 0.278 | |
| *Phascolarctobacterium* | | rs1264476 | T | G | 0.200 | 0.077 | 0.017 | 21.0 | 4.30E-06 | -0.004 | 0.023 | 0.854 | |
| *Phascolarctobacterium* | | rs28525131 | G | A | 0.094 | -0.119 | 0.027 | 26.8 | 8.23E-06 | -0.016 | 0.041 | 0.701 | |
| *Phascolarctobacterium* | | rs56069061 | G | A | 0.101 | -0.111 | 0.023 | 25.1 | 1.87E-06 | -0.022 | 0.036 | 0.540 | |
| *Phascolarctobacterium* | | rs56157888 | A | C | 0.170 | 0.095 | 0.019 | 28.7 | 1.09E-06 | -0.017 | 0.021 | 0.428 | |
| *Phascolarctobacterium* | | rs74540770 | G | A | 0.065 | -0.121 | 0.026 | 19.7 | 3.60E-06 | -0.005 | 0.032 | 0.887 | |
| *Phascolarctobacterium* | | rs75882962 | T | C | 0.167 | 0.097 | 0.019 | 29.1 | 3.19E-07 | -0.040 | 0.026 | 0.130 | |
| *Phascolarctobacterium* | | rs7982713 | G | A | 0.229 | 0.073 | 0.016 | 20.7 | 9.72E-06 | 0.016 | 0.020 | 0.420 | |
| *Prevotella 7* | | rs118038478 | A | G | 0.076 | 0.206 | 0.047 | 19.6 | 7.85E-06 | 0.013 | 0.035 | 0.708 | |
| *Prevotella 7* | | rs12124567 | A | G | 0.262 | -0.121 | 0.028 | 18.9 | 9.49E-06 | 0.033 | 0.022 | 0.134 | |
| *Prevotella 7* | | rs12195431 | T | C | 0.079 | 0.197 | 0.044 | 18.5 | 8.73E-06 | 0.033 | 0.030 | 0.269 | |
| *Prevotella 7* | | rs2240542 | C | T | 0.258 | 0.121 | 0.026 | 18.5 | 4.84E-06 | -0.016 | 0.020 | 0.413 | |
| *Prevotella 7* | | rs2918132 | C | T | 0.368 | -0.115 | 0.025 | 20.3 | 6.42E-06 | -0.026 | 0.018 | 0.155 | |
| *Prevotella 7* | | rs430270 | A | C | 0.201 | 0.139 | 0.030 | 20.6 | 2.87E-06 | 0.012 | 0.023 | 0.588 | |
| *Prevotella 7* | | rs57404562 | C | A | 0.180 | 0.155 | 0.032 | 23.7 | 6.22E-07 | 0.008 | 0.026 | 0.775 | |
| *Prevotella 7* | | rs79263163 | A | C | 0.185 | -0.144 | 0.032 | 20.7 | 7.51E-06 | -0.011 | 0.022 | 0.618 | |
| *Prevotella 7* | | rs9426434 | T | C | 0.273 | -0.124 | 0.028 | 20.1 | 9.72E-06 | -0.005 | 0.019 | 0.771 | |
| *Prevotella 7* | | rs9608249 | A | G | 0.171 | -0.158 | 0.034 | 23.5 | 2.07E-06 | -0.010 | 0.028 | 0.733 | |
| *Prevotella 7* | | rs9959718 | G | A | 0.250 | 0.133 | 0.028 | 22.0 | 1.90E-06 | 0.003 | 0.022 | 0.887 | |
| *Prevotella 9* | | rs111509883 | T | C | 0.085 | 0.171 | 0.035 | 31.7 | 1.24E-06 | -0.005 | 0.028 | 0.852 | |
| *Prevotella 9* | | rs11685699 | C | T | 0.083 | -0.141 | 0.030 | 25.3 | 2.03E-06 | 0.043 | 0.033 | 0.192 | |
| *Prevotella 9* | | rs117271932 | A | G | 0.047 | 0.208 | 0.044 | 18.9 | 2.82E-06 | -0.038 | 0.038 | 0.317 | |
| *Prevotella 9* | | rs12648235 | T | C | 0.157 | 0.079 | 0.018 | 16.8 | 7.39E-06 | 0.019 | 0.021 | 0.360 | |
| *Prevotella 9* | | rs1304512 | G | A | 0.219 | 0.076 | 0.017 | 19.4 | 5.29E-06 | -0.004 | 0.020 | 0.835 | |
| *Prevotella 9* | | rs2104588 | T | C | 0.119 | 0.106 | 0.024 | 21.7 | 8.13E-06 | -0.069 | 0.038 | 0.068 | |
| *Prevotella 9* | | rs2495052 | A | G | 0.179 | 0.084 | 0.019 | 20.3 | 8.97E-06 | -0.028 | 0.025 | 0.252 | |
| *Prevotella 9* | | rs2683313 | A | G | 0.308 | -0.072 | 0.015 | 23.0 | 1.69E-06 | -0.015 | 0.019 | 0.427 | |
| *Prevotella 9* | | rs4968431 | G | T | 0.364 | 0.064 | 0.014 | 19.5 | 8.58E-06 | -0.007 | 0.018 | 0.691 | |
| *Prevotella 9* | | rs7237249 | C | T | 0.135 | -0.082 | 0.018 | 16.3 | 8.93E-06 | 0.024 | 0.023 | 0.284 | |
| *Prevotella 9* | | rs72815774 | T | C | 0.071 | -0.176 | 0.039 | 23.4 | 8.78E-06 | 0.054 | 0.039 | 0.170 | |
| *Prevotella 9* | | rs746764 | T | C | 0.173 | -0.092 | 0.019 | 22.6 | 2.04E-06 | -0.016 | 0.021 | 0.448 | |
| *Prevotella 9* | | rs7976209 | T | C | 0.167 | -0.087 | 0.020 | 21.7 | 7.28E-06 | -0.022 | 0.024 | 0.368 | |
| *Prevotella 9* | | rs9428102 | A | G | 0.237 | -0.078 | 0.018 | 22.5 | 4.62E-06 | -0.002 | 0.021 | 0.919 | |
| *Prevotella 9* | | rs9613013 | G | A | 0.145 | 0.092 | 0.020 | 20.5 | 6.10E-06 | 0.001 | 0.027 | 0.968 | |
| *Rikenellaceae (RC9gut group)* | | rs12501673 | A | G | 0.325 | 0.116 | 0.026 | 19.9 | 6.29E-06 | 0.014 | 0.020 | 0.493 | |
| *Rikenellaceae (RC9gut group)* | | rs17032291 | T | C | 0.141 | -0.170 | 0.037 | 23.4 | 6.61E-06 | 0.003 | 0.027 | 0.926 | |
| *Rikenellaceae (RC9gut group)* | | rs17582787 | A | G | 0.129 | -0.158 | 0.034 | 18.8 | 3.55E-06 | 0.008 | 0.024 | 0.730 | |
| *Rikenellaceae (RC9gut group)* | | rs2074881 | T | C | 0.179 | -0.142 | 0.032 | 19.9 | 9.45E-06 | 0.015 | 0.026 | 0.555 | |
| *Rikenellaceae (RC9gut group)* | | rs2900503 | G | T | 0.171 | -0.172 | 0.033 | 28.3 | 1.55E-07 | 0.027 | 0.024 | 0.254 | |
| *Rikenellaceae (RC9gut group)* | | rs2998141 | T | C | 0.222 | -0.136 | 0.029 | 21.5 | 4.42E-06 | 0.022 | 0.021 | 0.287 | |
| *Rikenellaceae (RC9gut group)* | | rs4270579 | G | A | 0.304 | -0.118 | 0.027 | 19.8 | 5.46E-06 | -0.009 | 0.019 | 0.638 | |
| *Rikenellaceae (RC9gut group)* | | rs4717843 | G | T | 0.362 | -0.119 | 0.026 | 22.1 | 4.72E-06 | -0.002 | 0.018 | 0.906 | |
| *Rikenellaceae (RC9gut group)* | | rs7712231 | A | G | 0.165 | 0.156 | 0.035 | 22.6 | 7.97E-06 | 0.050 | 0.026 | 0.054 | |
| *Rikenellaceae (RC9gut group)* | | rs80309088 | G | A | 0.129 | 0.174 | 0.038 | 22.9 | 4.56E-06 | -0.018 | 0.027 | 0.506 | |
| *Rikenellaceae (RC9gut group)* | | rs9887954 | G | A | 0.436 | -0.115 | 0.025 | 21.8 | 4.81E-06 | 0.052 | 0.018 | 0.003 | |
| *Romboutsia* | | rs10279978 | A | G | 0.348 | -0.062 | 0.013 | 24.9 | 1.17E-06 | -0.056 | 0.019 | 0.003 | |
| *Romboutsia* | | rs11221428 | T | C | 0.155 | -0.073 | 0.016 | 19.6 | 6.49E-06 | -0.025 | 0.021 | 0.231 | |
| *Romboutsia* | | rs16843578 | C | T | 0.083 | -0.088 | 0.020 | 16.4 | 5.08E-06 | 0.046 | 0.039 | 0.237 | |
| *Romboutsia* | | rs28603357 | T | C | 0.064 | -0.215 | 0.047 | 78.2 | 8.52E-06 | -0.056 | 0.063 | 0.371 | |
| *Romboutsia* | | rs34302036 | A | G | 0.415 | 0.055 | 0.012 | 20.8 | 5.88E-06 | 0.006 | 0.018 | 0.742 | |
| *Romboutsia* | | rs61841503 | G | A | 0.137 | 0.093 | 0.017 | 28.9 | 4.00E-08 | 0.002 | 0.026 | 0.953 | |
| *Romboutsia* | | rs62504452 | A | G | 0.166 | -0.071 | 0.016 | 19.8 | 4.66E-06 | 0.019 | 0.025 | 0.460 | |
| *Romboutsia* | | rs7109293 | A | G | 0.092 | 0.092 | 0.021 | 20.1 | 6.98E-06 | -0.035 | 0.028 | 0.206 | |
| *Romboutsia* | | rs75200530 | T | G | 0.047 | -0.191 | 0.042 | 45.9 | 5.07E-06 | -0.030 | 0.052 | 0.570 | |
| *Romboutsia* | | rs75987356 | G | A | 0.069 | -0.130 | 0.028 | 30.4 | 6.71E-06 | 0.038 | 0.033 | 0.252 | |
| *Romboutsia* | | rs77702691 | A | G | 0.102 | -0.094 | 0.021 | 23.2 | 7.37E-06 | -0.006 | 0.031 | 0.858 | |
| *Romboutsia* | | rs9389266 | T | G | 0.169 | 0.072 | 0.016 | 20.8 | 9.38E-06 | 0.039 | 0.023 | 0.093 | |
| *Romboutsia* | | rs9567264 | C | T | 0.320 | 0.058 | 0.013 | 20.7 | 5.76E-06 | -0.017 | 0.019 | 0.368 | |
| *Roseburia* | | rs12740451 | T | C | 0.125 | 0.070 | 0.015 | 19.0 | 7.34E-06 | 0.012 | 0.026 | 0.655 | |
| *Roseburia* | | rs147990086 | A | G | 0.191 | -0.058 | 0.013 | 18.5 | 8.93E-06 | 0.004 | 0.023 | 0.880 | |
| *Roseburia* | | rs16910295 | T | C | 0.061 | -0.098 | 0.021 | 18.2 | 2.91E-06 | 0.026 | 0.039 | 0.506 | |
| *Roseburia* | | rs2160994 | T | C | 0.380 | 0.055 | 0.011 | 25.0 | 9.70E-07 | 0.053 | 0.019 | 0.005 | |
| *Roseburia* | | rs2943022 | T | C | 0.460 | 0.049 | 0.011 | 21.7 | 4.11E-06 | 0.027 | 0.018 | 0.126 | |
| *Roseburia* | | rs302266 | T | C | 0.120 | -0.078 | 0.017 | 21.8 | 8.13E-06 | 0.000 | 0.026 | 0.997 | |
| *Roseburia* | | rs329182 | T | C | 0.157 | 0.069 | 0.015 | 22.6 | 5.90E-06 | -0.038 | 0.024 | 0.116 | |
| *Roseburia* | | rs55858165 | A | C | 0.078 | 0.179 | 0.040 | 25.1 | 9.99E-06 | -0.009 | 0.046 | 0.850 | |
| *Roseburia* | | rs57466170 | C | T | 0.087 | 0.074 | 0.017 | 15.1 | 8.30E-06 | 0.050 | 0.034 | 0.139 | |
| *Roseburia* | | rs6445851 | G | A | 0.401 | -0.050 | 0.011 | 21.2 | 3.53E-06 | 0.027 | 0.018 | 0.134 | |
| *Roseburia* | | rs6930661 | C | T | 0.076 | -0.096 | 0.020 | 21.9 | 2.48E-06 | -0.015 | 0.037 | 0.687 | |
| *Roseburia* | | rs75326254 | C | T | 0.064 | -0.105 | 0.023 | 20.7 | 7.50E-06 | -0.045 | 0.037 | 0.233 | |
| *Roseburia* | | rs78753150 | A | C | 0.059 | 0.097 | 0.021 | 16.9 | 9.98E-06 | -0.014 | 0.029 | 0.635 | |
| *Roseburia* | | rs9300744 | C | T | 0.239 | -0.059 | 0.013 | 22.5 | 4.75E-06 | -0.016 | 0.023 | 0.492 | |
| *Ruminiclostridium 5* | | rs10827477 | A | G | 0.315 | -0.055 | 0.012 | 22.5 | 2.19E-06 | -0.030 | 0.018 | 0.101 | |
| *Ruminiclostridium 5* | | rs113753996 | T | C | 0.137 | 0.082 | 0.017 | 27.8 | 3.99E-06 | -0.034 | 0.023 | 0.136 | |
| *Ruminiclostridium 5* | | rs1223978 | T | C | 0.480 | 0.048 | 0.011 | 20.4 | 8.16E-06 | 0.010 | 0.018 | 0.568 | |
| *Ruminiclostridium 5* | | rs1492620 | T | C | 0.081 | -0.083 | 0.018 | 17.8 | 3.53E-06 | -0.028 | 0.026 | 0.296 | |
| *Ruminiclostridium 5* | | rs2482038 | C | A | 0.438 | 0.052 | 0.011 | 23.1 | 1.70E-06 | -0.002 | 0.018 | 0.909 | |
| *Ruminiclostridium 5* | | rs2791343 | T | C | 0.340 | 0.052 | 0.011 | 20.9 | 5.54E-06 | 0.003 | 0.018 | 0.878 | |
| *Ruminiclostridium 5* | | rs2833828 | G | A | 0.453 | 0.049 | 0.011 | 20.7 | 6.82E-06 | 0.012 | 0.018 | 0.487 | |
| *Ruminiclostridium 5* | | rs4955951 | A | G | 0.092 | -0.071 | 0.017 | 14.9 | 9.96E-06 | -0.016 | 0.027 | 0.560 | |
| *Ruminiclostridium 5* | | rs6121460 | G | A | 0.081 | 0.093 | 0.020 | 22.4 | 2.64E-06 | -0.038 | 0.033 | 0.246 | |
| *Ruminiclostridium 5* | | rs79968837 | A | G | 0.069 | -0.095 | 0.019 | 20.1 | 1.15E-06 | 0.014 | 0.040 | 0.734 | |
| *Ruminiclostridium 5* | | rs8053158 | A | G | 0.120 | -0.074 | 0.016 | 20.2 | 5.90E-06 | -0.017 | 0.027 | 0.525 | |
| *Ruminiclostridium 6* | | rs10829821 | T | C | 0.086 | -0.098 | 0.022 | 19.1 | 3.47E-06 | -0.015 | 0.031 | 0.615 | |
| *Ruminiclostridium 6* | | rs116969552 | A | G | 0.036 | -0.167 | 0.038 | 24.6 | 9.16E-06 | 0.032 | 0.053 | 0.549 | |
| *Ruminiclostridium 6* | | rs11992182 | A | C | 0.288 | 0.063 | 0.014 | 20.6 | 4.65E-06 | 0.021 | 0.021 | 0.320 | |
| *Ruminiclostridium 6* | | rs2548459 | C | T | 0.470 | 0.055 | 0.012 | 19.7 | 6.40E-06 | 0.038 | 0.018 | 0.035 | |
| *Ruminiclostridium 6* | | rs35362464 | C | A | 0.151 | 0.072 | 0.017 | 17.0 | 8.99E-06 | -0.040 | 0.025 | 0.116 | |
| *Ruminiclostridium 6* | | rs61060922 | T | G | 0.045 | 0.159 | 0.032 | 27.7 | 1.09E-06 | -0.008 | 0.049 | 0.863 | |
| *Ruminiclostridium 6* | | rs663262 | T | C | 0.052 | -0.135 | 0.031 | 22.9 | 3.39E-06 | -0.006 | 0.049 | 0.900 | |
| *Ruminiclostridium 6* | | rs67479537 | T | C | 0.080 | 0.119 | 0.026 | 26.6 | 9.30E-06 | -0.059 | 0.041 | 0.152 | |
| *Ruminiclostridium 6* | | rs71414120 | T | G | 0.077 | 0.201 | 0.041 | 73.4 | 1.08E-06 | 0.023 | 0.040 | 0.569 | |
| *Ruminiclostridium 6* | | rs72991535 | T | G | 0.060 | 0.136 | 0.030 | 26.4 | 4.95E-06 | 0.101 | 0.047 | 0.034 | |
| *Ruminiclostridium 6* | | rs73176030 | T | C | 0.318 | 0.059 | 0.013 | 19.1 | 7.29E-06 | 0.044 | 0.020 | 0.025 | |
| *Ruminiclostridium 6* | | rs77193512 | A | G | 0.167 | 0.074 | 0.015 | 19.3 | 1.30E-06 | -0.007 | 0.020 | 0.728 | |
| *Ruminiclostridium 6* | | rs792058 | G | A | 0.427 | 0.055 | 0.013 | 19.3 | 8.58E-06 | -0.008 | 0.018 | 0.654 | |
| *Ruminiclostridium 6* | | rs79968172 | G | A | 0.056 | 0.116 | 0.024 | 18.2 | 1.66E-06 | -0.021 | 0.037 | 0.565 | |
| *Ruminiclostridium 6* | | rs9555756 | A | C | 0.124 | -0.080 | 0.018 | 18.0 | 7.10E-06 | -0.015 | 0.032 | 0.625 | |
| *Ruminiclostridium 9* | | rs12040548 | G | T | 0.311 | 0.057 | 0.012 | 23.3 | 3.15E-06 | 0.004 | 0.020 | 0.852 | |
| *Ruminiclostridium 9* | | rs6082461 | A | C | 0.207 | 0.059 | 0.013 | 18.9 | 4.87E-06 | -0.023 | 0.022 | 0.291 | |
| *Ruminiclostridium 9* | | rs7137760 | C | T | 0.415 | 0.051 | 0.011 | 21.0 | 7.07E-06 | 0.000 | 0.018 | 0.998 | |
| *Ruminiclostridium 9* | | rs74303178 | T | C | 0.320 | 0.053 | 0.012 | 20.7 | 7.92E-06 | -0.019 | 0.019 | 0.323 | |
| *Ruminiclostridium 9* | | rs78191726 | T | C | 0.091 | 0.094 | 0.021 | 24.6 | 7.58E-06 | -0.086 | 0.034 | 0.012 | |
| *Ruminiclostridium 9* | | rs918449 | A | G | 0.070 | -0.095 | 0.020 | 19.6 | 2.56E-06 | -0.027 | 0.034 | 0.429 | |
| *Ruminiclostridium 9* | | rs9522712 | T | C | 0.145 | 0.070 | 0.015 | 20.3 | 4.66E-06 | 0.002 | 0.025 | 0.942 | |
| *Ruminiclostridium 9* | | rs9809789 | C | T | 0.168 | -0.072 | 0.016 | 24.1 | 8.72E-06 | 0.022 | 0.023 | 0.327 | |
| *Ruminococcaceae (NK4A214 group)* | | rs11241747 | C | T | 0.391 | 0.053 | 0.012 | 20.1 | 6.59E-06 | -0.006 | 0.019 | 0.774 | |
| *Ruminococcaceae (NK4A214 group)* | | rs11586410 | G | A | 0.134 | -0.086 | 0.017 | 25.7 | 3.66E-07 | -0.020 | 0.025 | 0.419 | |
| *Ruminococcaceae (NK4A214 group)* | | rs12642039 | T | C | 0.366 | -0.055 | 0.012 | 21.1 | 3.43E-06 | -0.028 | 0.018 | 0.130 | |
| *Ruminococcaceae (NK4A214 group)* | | rs12731 | A | G | 0.451 | -0.053 | 0.012 | 20.5 | 4.87E-06 | 0.012 | 0.018 | 0.510 | |
| *Ruminococcaceae (NK4A214 group)* | | rs13087692 | T | G | 0.305 | 0.057 | 0.013 | 20.8 | 8.69E-06 | -0.003 | 0.019 | 0.884 | |
| *Ruminococcaceae (NK4A214 group)* | | rs136761 | G | A | 0.429 | -0.059 | 0.012 | 25.1 | 8.15E-07 | -0.019 | 0.018 | 0.304 | |
| *Ruminococcaceae (NK4A214 group)* | | rs147475196 | A | G | 0.078 | -0.134 | 0.030 | 38.0 | 4.72E-06 | 0.033 | 0.029 | 0.254 | |
| *Ruminococcaceae (NK4A214 group)* | | rs35559912 | T | C | 0.066 | -0.093 | 0.020 | 15.6 | 4.89E-06 | 0.049 | 0.027 | 0.068 | |
| *Ruminococcaceae (NK4A214 group)* | | rs4814689 | C | T | 0.067 | -0.108 | 0.023 | 21.6 | 4.55E-06 | 0.022 | 0.042 | 0.607 | |
| *Ruminococcaceae (NK4A214 group)* | | rs5994253 | A | G | 0.146 | -0.081 | 0.016 | 24.4 | 2.35E-07 | -0.018 | 0.025 | 0.472 | |
| *Ruminococcaceae (NK4A214 group)* | | rs62027366 | T | C | 0.230 | 0.062 | 0.014 | 19.9 | 6.58E-06 | 0.005 | 0.022 | 0.832 | |
| *Ruminococcaceae (NK4A214 group)* | | rs6681678 | C | T | 0.050 | -0.100 | 0.024 | 14.1 | 9.05E-06 | 0.031 | 0.049 | 0.529 | |
| *Ruminococcaceae (NK4A214 group)* | | rs7573569 | T | C | 0.072 | 0.108 | 0.023 | 22.9 | 3.23E-06 | -0.008 | 0.037 | 0.830 | |
| *Ruminococcaceae (UCG002)* | | rs10916131 | C | T | 0.138 | -0.069 | 0.015 | 19.6 | 2.87E-06 | -0.001 | 0.024 | 0.967 | |
| *Ruminococcaceae (UCG002)* | | rs10927423 | C | A | 0.183 | -0.071 | 0.015 | 26.1 | 8.50E-07 | -0.046 | 0.023 | 0.044 | |
| *Ruminococcaceae (UCG002)* | | rs10964441 | G | A | 0.053 | -0.149 | 0.034 | 38.0 | 7.45E-06 | -0.034 | 0.029 | 0.242 | |
| *Ruminococcaceae (UCG002)* | | rs113147300 | A | G | 0.132 | -0.076 | 0.016 | 22.6 | 7.69E-06 | 0.027 | 0.026 | 0.293 | |
| *Ruminococcaceae (UCG002)* | | rs11607472 | A | G | 0.107 | -0.078 | 0.018 | 20.0 | 7.19E-06 | 0.029 | 0.035 | 0.412 | |
| *Ruminococcaceae (UCG002)* | | rs116974815 | C | A | 0.058 | -0.190 | 0.040 | 67.2 | 2.03E-06 | -0.069 | 0.035 | 0.051 | |
| *Ruminococcaceae (UCG002)* | | rs11750293 | G | T | 0.281 | -0.058 | 0.012 | 23.1 | 1.76E-06 | -0.011 | 0.018 | 0.551 | |
| *Ruminococcaceae (UCG002)* | | rs12463378 | A | G | 0.418 | -0.052 | 0.011 | 22.7 | 2.96E-06 | 0.008 | 0.019 | 0.691 | |
| *Ruminococcaceae (UCG002)* | | rs15256 | C | T | 0.087 | 0.073 | 0.017 | 14.5 | 9.46E-06 | 0.031 | 0.027 | 0.254 | |
| *Ruminococcaceae (UCG002)* | | rs55793120 | T | C | 0.051 | 0.137 | 0.027 | 31.1 | 4.81E-07 | 0.067 | 0.037 | 0.070 | |
| *Ruminococcaceae (UCG002)* | | rs56030423 | G | A | 0.089 | -0.098 | 0.022 | 26.7 | 6.30E-06 | 0.022 | 0.034 | 0.520 | |
| *Ruminococcaceae (UCG002)* | | rs57079348 | T | G | 0.121 | -0.077 | 0.017 | 21.4 | 7.22E-06 | -0.018 | 0.038 | 0.629 | |
| *Ruminococcaceae (UCG002)* | | rs6542556 | A | G | 0.345 | 0.051 | 0.011 | 20.1 | 7.86E-06 | -0.046 | 0.018 | 0.011 | |
| *Ruminococcaceae (UCG002)* | | rs6793778 | C | T | 0.262 | -0.056 | 0.013 | 20.7 | 9.81E-06 | 0.000 | 0.020 | 0.995 | |
| *Ruminococcaceae (UCG002)* | | rs7120052 | A | C | 0.176 | 0.062 | 0.014 | 19.4 | 1.97E-06 | 0.035 | 0.022 | 0.119 | |
| *Ruminococcaceae (UCG002)* | | rs7155595 | C | A | 0.309 | 0.057 | 0.012 | 23.7 | 1.15E-06 | 0.057 | 0.019 | 0.003 | |
| *Ruminococcaceae (UCG002)* | | rs7249614 | A | G | 0.385 | -0.049 | 0.011 | 19.7 | 9.07E-06 | -0.006 | 0.018 | 0.758 | |
| *Ruminococcaceae (UCG002)* | | rs7342369 | C | A | 0.323 | -0.053 | 0.012 | 20.9 | 5.66E-06 | 0.017 | 0.020 | 0.391 | |
| *Ruminococcaceae (UCG002)* | | rs76847269 | A | G | 0.057 | 0.164 | 0.036 | 49.0 | 5.17E-06 | 0.006 | 0.056 | 0.912 | |
| *Ruminococcaceae (UCG002)* | | rs77564310 | A | C | 0.179 | -0.071 | 0.014 | 25.6 | 3.29E-07 | -0.002 | 0.022 | 0.936 | |
| *Ruminococcaceae (UCG002)* | | rs79016051 | C | T | 0.091 | -0.089 | 0.019 | 22.2 | 2.34E-06 | 0.026 | 0.026 | 0.324 | |
| *Ruminococcaceae (UCG002)* | | rs882348 | A | G | 0.106 | -0.080 | 0.018 | 20.8 | 5.45E-06 | 0.006 | 0.027 | 0.833 | |
| *Ruminococcaceae (UCG003)* | | rs10490280 | C | T | 0.218 | -0.067 | 0.014 | 21.6 | 4.16E-06 | 0.004 | 0.023 | 0.854 | |
| *Ruminococcaceae (UCG003)* | | rs11243416 | T | C | 0.101 | -0.093 | 0.019 | 21.9 | 1.67E-06 | 0.083 | 0.035 | 0.017 | |
| *Ruminococcaceae (UCG003)* | | rs11613919 | G | T | 0.213 | 0.073 | 0.016 | 24.9 | 1.63E-06 | -0.020 | 0.021 | 0.333 | |
| *Ruminococcaceae (UCG003)* | | rs16959793 | A | C | 0.280 | -0.063 | 0.013 | 22.1 | 2.22E-06 | 0.001 | 0.018 | 0.967 | |
| *Ruminococcaceae (UCG003)* | | rs2523124 | T | C | 0.398 | -0.055 | 0.012 | 20.1 | 5.78E-06 | -0.016 | 0.018 | 0.382 | |
| *Ruminococcaceae (UCG003)* | | rs3013089 | G | A | 0.385 | -0.055 | 0.012 | 20.2 | 4.38E-06 | -0.024 | 0.018 | 0.186 | |
| *Ruminococcaceae (UCG003)* | | rs4452755 | A | C | 0.305 | -0.063 | 0.013 | 23.9 | 3.29E-06 | 0.004 | 0.019 | 0.833 | |
| *Ruminococcaceae (UCG003)* | | rs4532474 | G | A | 0.149 | 0.077 | 0.017 | 21.1 | 4.82E-06 | 0.003 | 0.024 | 0.887 | |
| *Ruminococcaceae (UCG003)* | | rs646327 | G | A | 0.468 | 0.059 | 0.012 | 24.0 | 7.83E-07 | 0.039 | 0.018 | 0.031 | |
| *Ruminococcaceae (UCG003)* | | rs6759615 | A | G | 0.070 | 0.103 | 0.020 | 19.1 | 7.86E-07 | -0.010 | 0.030 | 0.747 | |
| *Ruminococcaceae (UCG003)* | | rs73341549 | T | C | 0.051 | -0.170 | 0.032 | 39.0 | 1.51E-07 | -0.020 | 0.037 | 0.585 | |
| *Ruminococcaceae (UCG003)* | | rs78720113 | A | G | 0.070 | -0.115 | 0.025 | 24.2 | 7.59E-06 | -0.034 | 0.033 | 0.295 | |
| *Ruminococcaceae (UCG004)* | | rs10976229 | T | G | 0.130 | 0.096 | 0.021 | 20.4 | 7.04E-06 | 0.001 | 0.026 | 0.958 | |
| *Ruminococcaceae (UCG004)* | | rs11961899 | G | A | 0.278 | -0.071 | 0.016 | 18.1 | 9.18E-06 | -0.005 | 0.020 | 0.784 | |
| *Ruminococcaceae (UCG004)* | | rs12125734 | G | T | 0.093 | 0.134 | 0.026 | 25.9 | 2.09E-07 | 0.046 | 0.030 | 0.131 | |
| *Ruminococcaceae (UCG004)* | | rs2248146 | T | C | 0.344 | 0.069 | 0.015 | 19.0 | 8.20E-06 | 0.013 | 0.018 | 0.495 | |
| *Ruminococcaceae (UCG004)* | | rs3800154 | A | C | 0.263 | -0.080 | 0.018 | 20.0 | 6.12E-06 | 0.021 | 0.020 | 0.295 | |
| *Ruminococcaceae (UCG004)* | | rs511258 | G | A | 0.187 | -0.076 | 0.016 | 20.3 | 4.52E-06 | 0.014 | 0.022 | 0.532 | |
| *Ruminococcaceae (UCG004)* | | rs550351 | A | C | 0.424 | 0.079 | 0.018 | 27.8 | 9.43E-06 | -0.001 | 0.018 | 0.951 | |
| *Ruminococcaceae (UCG004)* | | rs6769553 | A | G | 0.260 | 0.085 | 0.016 | 26.9 | 7.91E-08 | -0.033 | 0.020 | 0.100 | |
| *Ruminococcaceae (UCG004)* | | rs7569771 | A | G | 0.247 | -0.076 | 0.017 | 17.9 | 8.12E-06 | 0.021 | 0.020 | 0.302 | |
| *Ruminococcaceae (UCG004)* | | rs872501 | G | A | 0.099 | 0.116 | 0.026 | 27.4 | 5.81E-06 | 0.007 | 0.033 | 0.829 | |
| *Ruminococcaceae (UCG004)* | | rs9818949 | G | T | 0.203 | 0.086 | 0.019 | 23.1 | 5.39E-06 | 0.002 | 0.022 | 0.942 | |
| *Ruminococcaceae (UCG005)* | | rs10873449 | T | C | 0.169 | 0.065 | 0.014 | 19.4 | 4.11E-06 | 0.011 | 0.022 | 0.636 | |
| *Ruminococcaceae (UCG005)* | | rs10937802 | G | A | 0.097 | 0.076 | 0.017 | 16.2 | 8.17E-06 | -0.030 | 0.027 | 0.276 | |
| *Ruminococcaceae (UCG005)* | | rs10950694 | T | C | 0.424 | 0.058 | 0.011 | 26.3 | 4.30E-07 | -0.014 | 0.018 | 0.449 | |
| *Ruminococcaceae (UCG005)* | | rs114279581 | A | G | 0.048 | -0.147 | 0.032 | 31.5 | 3.22E-06 | 0.003 | 0.033 | 0.936 | |
| *Ruminococcaceae (UCG005)* | | rs12288512 | A | G | 0.180 | 0.067 | 0.014 | 21.1 | 3.10E-06 | -0.030 | 0.021 | 0.144 | |
| *Ruminococcaceae (UCG005)* | | rs12458218 | T | C | 0.177 | 0.068 | 0.014 | 21.5 | 2.41E-06 | 0.003 | 0.023 | 0.893 | |
| *Ruminococcaceae (UCG005)* | | rs2893871 | G | A | 0.152 | -0.074 | 0.016 | 22.5 | 3.54E-06 | -0.015 | 0.028 | 0.594 | |
| *Ruminococcaceae (UCG005)* | | rs34781347 | G | A | 0.060 | 0.189 | 0.039 | 64.5 | 6.05E-07 | -0.064 | 0.034 | 0.060 | |
| *Ruminococcaceae (UCG005)* | | rs55793120 | T | C | 0.051 | 0.122 | 0.028 | 22.9 | 7.37E-06 | 0.067 | 0.037 | 0.070 | |
| *Ruminococcaceae (UCG005)* | | rs72776570 | C | A | 0.083 | 0.087 | 0.020 | 18.5 | 5.36E-06 | -0.035 | 0.030 | 0.234 | |
| *Ruminococcaceae (UCG005)* | | rs7449320 | C | A | 0.221 | 0.060 | 0.013 | 19.9 | 4.81E-06 | 0.012 | 0.021 | 0.566 | |
| *Ruminococcaceae (UCG005)* | | rs7555878 | A | G | 0.280 | 0.059 | 0.013 | 22.4 | 2.81E-06 | -0.023 | 0.020 | 0.250 | |
| *Ruminococcaceae (UCG005)* | | rs7586445 | G | A | 0.126 | 0.078 | 0.018 | 21.8 | 8.81E-06 | -0.016 | 0.026 | 0.534 | |
| *Ruminococcaceae (UCG005)* | | rs898577 | T | C | 0.055 | -0.123 | 0.029 | 25.2 | 7.46E-06 | -0.011 | 0.038 | 0.768 | |
| *Ruminococcaceae (UCG009)* | | rs113006825 | T | C | 0.180 | -0.093 | 0.021 | 18.3 | 7.98E-06 | -0.012 | 0.022 | 0.591 | |
| *Ruminococcaceae (UCG009)* | | rs12508214 | C | T | 0.351 | -0.077 | 0.017 | 19.6 | 4.75E-06 | 0.018 | 0.019 | 0.351 | |
| *Ruminococcaceae (UCG009)* | | rs138460696 | A | G | 0.106 | 0.139 | 0.032 | 26.5 | 9.81E-06 | -0.007 | 0.033 | 0.832 | |
| *Ruminococcaceae (UCG009)* | | rs1550196 | G | A | 0.117 | 0.131 | 0.026 | 25.5 | 1.13E-06 | -0.012 | 0.029 | 0.684 | |
| *Ruminococcaceae (UCG009)* | | rs2058609 | A | G | 0.314 | 0.082 | 0.017 | 20.6 | 3.12E-06 | 0.004 | 0.020 | 0.834 | |
| *Ruminococcaceae (UCG009)* | | rs2192926 | A | G | 0.237 | -0.089 | 0.019 | 20.6 | 4.88E-06 | -0.006 | 0.019 | 0.752 | |
| *Ruminococcaceae (UCG009)* | | rs4079028 | C | T | 0.194 | 0.092 | 0.020 | 18.8 | 3.28E-06 | 0.006 | 0.021 | 0.783 | |
| *Ruminococcaceae (UCG009)* | | rs4708333 | T | G | 0.302 | -0.084 | 0.017 | 21.4 | 1.56E-06 | 0.032 | 0.019 | 0.080 | |
| *Ruminococcaceae (UCG009)* | | rs6952765 | G | A | 0.461 | 0.073 | 0.017 | 19.1 | 8.13E-06 | -0.032 | 0.019 | 0.092 | |
| *Ruminococcaceae (UCG009)* | | rs758191 | T | G | 0.064 | 0.177 | 0.038 | 26.8 | 9.01E-06 | 0.035 | 0.030 | 0.235 | |
| *Ruminococcaceae (UCG009)* | | rs78410648 | A | G | 0.097 | 0.121 | 0.028 | 18.5 | 9.67E-06 | -0.032 | 0.028 | 0.265 | |
| *Ruminococcaceae (UCG009)* | | rs9558661 | T | C | 0.187 | -0.090 | 0.020 | 17.6 | 7.01E-06 | -0.019 | 0.022 | 0.376 | |
| *Ruminococcaceae (UCG010)* | | rs12597105 | G | A | 0.277 | 0.067 | 0.014 | 22.4 | 4.87E-06 | 0.013 | 0.023 | 0.563 | |
| *Ruminococcaceae (UCG010)* | | rs2820282 | A | C | 0.452 | -0.059 | 0.013 | 21.6 | 2.85E-06 | 0.001 | 0.018 | 0.960 | |
| *Ruminococcaceae (UCG010)* | | rs682403 | A | G | 0.476 | -0.059 | 0.012 | 21.5 | 2.37E-06 | -0.014 | 0.018 | 0.431 | |
| *Ruminococcaceae (UCG010)* | | rs6958419 | C | T | 0.489 | -0.059 | 0.012 | 21.3 | 2.84E-06 | 0.033 | 0.018 | 0.060 | |
| *Ruminococcaceae (UCG010)* | | rs73218807 | G | A | 0.058 | -0.166 | 0.037 | 37.4 | 6.43E-06 | -0.022 | 0.032 | 0.493 | |
| *Ruminococcaceae (UCG010)* | | rs7441445 | C | T | 0.435 | -0.057 | 0.013 | 19.8 | 6.80E-06 | 0.019 | 0.018 | 0.273 | |
| *Ruminococcaceae (UCG011)* | | rs10274562 | C | T | 0.352 | 0.111 | 0.024 | 20.5 | 6.50E-06 | 0.024 | 0.018 | 0.183 | |
| *Ruminococcaceae (UCG011)* | | rs12636310 | G | A | 0.218 | 0.133 | 0.028 | 21.9 | 2.81E-06 | 0.028 | 0.020 | 0.167 | |
| *Ruminococcaceae (UCG011)* | | rs12724320 | C | T | 0.275 | -0.121 | 0.025 | 21.3 | 1.52E-06 | -0.007 | 0.018 | 0.683 | |
| *Ruminococcaceae (UCG011)* | | rs1416041 | A | C | 0.134 | -0.182 | 0.034 | 28.3 | 7.04E-08 | 0.012 | 0.022 | 0.578 | |
| *Ruminococcaceae (UCG011)* | | rs2729556 | C | T | 0.499 | -0.109 | 0.023 | 21.7 | 3.19E-06 | 0.009 | 0.018 | 0.609 | |
| *Ruminococcaceae (UCG011)* | | rs4490371 | T | C | 0.331 | -0.112 | 0.025 | 20.2 | 7.75E-06 | 0.001 | 0.018 | 0.939 | |
| *Ruminococcaceae (UCG011)* | | rs79113084 | C | T | 0.155 | -0.152 | 0.032 | 22.2 | 2.06E-06 | -0.021 | 0.029 | 0.477 | |
| *Ruminococcaceae (UCG011)* | | rs9729514 | A | G | 0.102 | 0.185 | 0.039 | 23.0 | 2.37E-06 | 0.029 | 0.030 | 0.344 | |
| *Ruminococcaceae (UCG013)* | | rs11581881 | C | T | 0.181 | 0.066 | 0.014 | 20.4 | 4.73E-06 | -0.013 | 0.021 | 0.021 | |
| *Ruminococcaceae (UCG013)* | | rs12189346 | G | A | 0.163 | 0.068 | 0.015 | 20.2 | 1.68E-06 | 0.006 | 0.022 | 0.022 | |
| *Ruminococcaceae (UCG013)* | | rs12336782 | T | C | 0.082 | -0.086 | 0.019 | 17.3 | 8.60E-06 | -0.014 | 0.033 | 0.033 | |
| *Ruminococcaceae (UCG013)* | | rs12485353 | G | A | 0.179 | -0.061 | 0.013 | 17.1 | 4.19E-06 | -0.002 | 0.020 | 0.020 | |
| *Ruminococcaceae (UCG013)* | | rs12781711 | C | T | 0.313 | -0.066 | 0.012 | 29.2 | 2.55E-08 | -0.009 | 0.020 | 0.020 | |
| *Ruminococcaceae (UCG013)* | | rs16918863 | A | C | 0.061 | 0.111 | 0.024 | 22.3 | 4.16E-06 | -0.011 | 0.036 | 0.036 | |
| *Ruminococcaceae (UCG013)* | | rs2730183 | G | A | 0.464 | -0.049 | 0.011 | 18.7 | 8.44E-06 | 0.050 | 0.018 | 0.018 | |
| *Ruminococcaceae (UCG013)* | | rs4385846 | G | T | 0.207 | 0.060 | 0.013 | 18.5 | 6.46E-06 | 0.022 | 0.022 | 0.022 | |
| *Ruminococcaceae (UCG013)* | | rs75088940 | T | C | 0.093 | -0.094 | 0.020 | 23.7 | 2.55E-06 | 0.025 | 0.035 | 0.035 | |
| *Ruminococcaceae (UCG013)* | | rs76973485 | G | T | 0.037 | 0.195 | 0.042 | 42.5 | 3.35E-06 | 0.035 | 0.045 | 0.045 | |
| *Ruminococcaceae (UCG013)* | | rs7784330 | G | A | 0.389 | -0.050 | 0.011 | 18.6 | 8.16E-06 | 0.006 | 0.018 | 0.018 | |
| *Ruminococcaceae (UCG013)* | | rs9313055 | T | C | 0.069 | 0.105 | 0.023 | 22.2 | 9.55E-06 | -0.030 | 0.032 | 0.032 | |
| *Ruminococcaceae (UCG014)* | | rs10495392 | C | T | 0.119 | -0.082 | 0.019 | 19.9 | 9.96E-06 | -0.004 | 0.034 | 0.896 | |
| *Ruminococcaceae (UCG014)* | | rs10791168 | A | G | 0.184 | -0.066 | 0.015 | 18.5 | 9.76E-06 | 0.014 | 0.023 | 0.531 | |
| *Ruminococcaceae (UCG014)* | | rs10941294 | C | T | 0.069 | -0.122 | 0.026 | 26.5 | 2.40E-06 | -0.098 | 0.039 | 0.011 | |
| *Ruminococcaceae (UCG014)* | | rs115777838 | T | C | 0.054 | -0.188 | 0.039 | 50.3 | 4.62E-07 | 0.004 | 0.028 | 0.877 | |
| *Ruminococcaceae (UCG014)* | | rs12638134 | T | G | 0.390 | 0.058 | 0.012 | 22.5 | 1.21E-06 | 0.001 | 0.018 | 0.965 | |
| *Ruminococcaceae (UCG014)* | | rs34402072 | C | T | 0.192 | -0.069 | 0.016 | 20.4 | 9.80E-06 | -0.028 | 0.025 | 0.256 | |
| *Ruminococcaceae (UCG014)* | | rs56105232 | G | A | 0.073 | 0.139 | 0.030 | 36.4 | 2.91E-06 | -0.041 | 0.037 | 0.267 | |
| *Ruminococcaceae (UCG014)* | | rs72809222 | T | C | 0.252 | 0.067 | 0.014 | 23.7 | 2.41E-06 | -0.032 | 0.022 | 0.142 | |
| *Ruminococcaceae (UCG014)* | | rs73186226 | G | A | 0.088 | -0.099 | 0.022 | 22.0 | 6.72E-06 | -0.050 | 0.034 | 0.141 | |
| *Ruminococcaceae (UCG014)* | | rs853612 | A | G | 0.403 | -0.053 | 0.012 | 18.7 | 9.75E-06 | 0.031 | 0.018 | 0.080 | |
| *Ruminococcaceae (UCG014)* | | rs995642 | C | T | 0.326 | 0.060 | 0.013 | 22.1 | 1.90E-06 | 0.008 | 0.021 | 0.693 | |
| *Ruminococcus 1* | | rs10167839 | A | G | 0.344 | 0.052 | 0.012 | 20.2 | 8.09E-06 | -0.012 | 0.018 | 0.511 | |
| *Ruminococcus 1* | | rs11783695 | G | T | 0.151 | -0.073 | 0.016 | 23.0 | 4.73E-06 | -0.054 | 0.024 | 0.027 | |
| *Ruminococcus 1* | | rs17781867 | C | T | 0.092 | 0.100 | 0.021 | 27.9 | 1.96E-06 | -0.025 | 0.035 | 0.462 | |
| *Ruminococcus 1* | | rs3819978 | C | T | 0.059 | -0.115 | 0.026 | 24.3 | 8.74E-06 | -0.048 | 0.033 | 0.140 | |
| *Ruminococcus 1* | | rs6105066 | T | C | 0.250 | -0.061 | 0.013 | 22.9 | 5.06E-06 | 0.025 | 0.020 | 0.204 | |
| *Ruminococcus 1* | | rs6493760 | C | T | 0.397 | 0.054 | 0.012 | 22.8 | 3.38E-06 | 0.008 | 0.018 | 0.676 | |
| *Ruminococcus 1* | | rs7117576 | A | G | 0.089 | 0.083 | 0.017 | 18.5 | 6.48E-07 | -0.023 | 0.031 | 0.461 | |
| *Ruminococcus 1* | | rs7583465 | C | T | 0.377 | 0.053 | 0.011 | 21.7 | 2.56E-06 | -0.002 | 0.018 | 0.912 | |
| *Ruminococcus 1* | | rs78572139 | G | A | 0.071 | 0.125 | 0.028 | 34.1 | 5.23E-06 | 0.013 | 0.029 | 0.669 | |
| *Ruminococcus 1* | | rs78613526 | G | A | 0.043 | 0.167 | 0.037 | 38.2 | 5.11E-06 | -0.062 | 0.040 | 0.124 | |
| *Ruminococcus 2* | | rs12406309 | A | C | 0.191 | -0.063 | 0.014 | 18.9 | 9.79E-06 | -0.025 | 0.021 | 0.244 | |
| *Ruminococcus 2* | | rs12986628 | C | T | 0.213 | 0.067 | 0.014 | 22.8 | 2.14E-06 | -0.023 | 0.022 | 0.306 | |
| *Ruminococcus 2* | | rs1819812 | G | T | 0.116 | 0.084 | 0.018 | 22.4 | 5.28E-06 | 0.008 | 0.040 | 0.842 | |
| *Ruminococcus 2* | | rs2368224 | T | G | 0.048 | 0.200 | 0.044 | 55.7 | 3.63E-06 | 0.031 | 0.041 | 0.446 | |
| *Ruminococcus 2* | | rs2846589 | G | T | 0.454 | 0.052 | 0.012 | 20.8 | 7.59E-06 | -0.018 | 0.018 | 0.320 | |
| *Ruminococcus 2* | | rs2997412 | A | G | 0.280 | -0.057 | 0.012 | 20.0 | 4.22E-06 | 0.028 | 0.020 | 0.153 | |
| *Ruminococcus 2* | | rs4400279 | A | G | 0.364 | 0.055 | 0.012 | 21.2 | 5.80E-06 | 0.042 | 0.019 | 0.024 | |
| *Ruminococcus 2* | | rs4799823 | C | T | 0.139 | 0.084 | 0.018 | 25.8 | 5.40E-06 | -0.015 | 0.023 | 0.523 | |
| *Ruminococcus 2* | | rs55707116 | C | A | 0.102 | 0.087 | 0.019 | 21.1 | 8.01E-06 | -0.021 | 0.034 | 0.539 | |
| *Ruminococcus 2* | | rs58681734 | A | G | 0.151 | 0.072 | 0.016 | 20.6 | 4.18E-06 | -0.031 | 0.022 | 0.157 | |
| *Ruminococcus 2* | | rs61791565 | T | C | 0.369 | -0.052 | 0.012 | 19.6 | 6.79E-06 | -0.008 | 0.018 | 0.636 | |
| *Ruminococcus 2* | | rs75140805 | T | G | 0.158 | 0.084 | 0.018 | 28.6 | 3.95E-06 | -0.012 | 0.023 | 0.605 | |
| *Ruminococcus 2* | | rs7635831 | G | A | 0.293 | 0.062 | 0.013 | 24.3 | 1.98E-06 | 0.005 | 0.018 | 0.785 | |
| *Ruminococcus 2* | | rs7693984 | G | A | 0.083 | -0.103 | 0.024 | 24.6 | 9.42E-06 | 0.006 | 0.043 | 0.895 | |
| *Ruminococcus 2* | | rs78120384 | A | G | 0.073 | -0.193 | 0.039 | 77.2 | 3.31E-07 | -0.063 | 0.030 | 0.039 | |
| *Ruminococcus (gauvreauii group)* | | rs10931481 | G | A | 0.311 | 0.061 | 0.013 | 21.4 | 3.38E-06 | -0.019 | 0.019 | 0.338 | |
| *Ruminococcus (gauvreauii group)* | | rs12079579 | A | G | 0.073 | 0.096 | 0.021 | 16.5 | 5.04E-06 | 0.056 | 0.032 | 0.080 | |
| *Ruminococcus (gauvreauii group)* | | rs12539819 | C | T | 0.095 | 0.111 | 0.024 | 28.3 | 4.49E-06 | -0.010 | 0.035 | 0.771 | |
| *Ruminococcus (gauvreauii group)* | | rs1391597 | C | T | 0.381 | 0.059 | 0.012 | 22.0 | 1.86E-06 | 0.001 | 0.018 | 0.973 | |
| *Ruminococcus (gauvreauii group)* | | rs2047242 | A | G | 0.297 | -0.068 | 0.013 | 25.6 | 2.46E-07 | 0.034 | 0.021 | 0.102 | |
| *Ruminococcus (gauvreauii group)* | | rs2105937 | A | G | 0.334 | 0.058 | 0.013 | 20.1 | 5.10E-06 | 0.040 | 0.019 | 0.032 | |
| *Ruminococcus (gauvreauii group)* | | rs2166943 | A | C | 0.438 | 0.057 | 0.012 | 21.2 | 5.28E-06 | -0.005 | 0.018 | 0.800 | |
| *Ruminococcus (gauvreauii group)* | | rs289410 | G | A | 0.268 | -0.065 | 0.014 | 22.6 | 2.27E-06 | -0.016 | 0.020 | 0.428 | |
| *Ruminococcus (gauvreauii group)* | | rs431418 | A | G | 0.119 | -0.095 | 0.021 | 25.3 | 5.54E-06 | -0.006 | 0.030 | 0.853 | |
| *Ruminococcus (gauvreauii group)* | | rs71386687 | T | G | 0.060 | 0.121 | 0.024 | 22.0 | 2.91E-07 | 0.001 | 0.028 | 0.967 | |
| *Ruminococcus (gauvreauii group)* | | rs73802842 | C | A | 0.143 | 0.074 | 0.017 | 17.8 | 7.48E-06 | 0.006 | 0.021 | 0.775 | |
| *Ruminococcus (gauvreauii group)* | | rs9870933 | A | G | 0.409 | 0.062 | 0.013 | 25.0 | 8.49E-07 | -0.025 | 0.018 | 0.164 | |
| *Ruminococcus (gnavus group)* | | rs11597105 | A | G | 0.112 | 0.115 | 0.025 | 16.6 | 6.95E-06 | -0.037 | 0.022 | 0.096 | |
| *Ruminococcus (gnavus group)* | | rs11864644 | T | C | 0.071 | -0.140 | 0.032 | 16.3 | 5.01E-06 | 0.031 | 0.028 | 0.264 | |
| *Ruminococcus (gnavus group)* | | rs12136548 | C | T | 0.291 | 0.090 | 0.020 | 21.3 | 3.10E-06 | -0.036 | 0.019 | 0.065 | |
| *Ruminococcus (gnavus group)* | | rs12989336 | G | A | 0.305 | -0.085 | 0.019 | 19.3 | 7.12E-06 | 0.022 | 0.020 | 0.269 | |
| *Ruminococcus (gnavus group)* | | rs13163520 | G | A | 0.181 | -0.127 | 0.023 | 30.6 | 5.61E-08 | -0.039 | 0.023 | 0.082 | |
| *Ruminococcus (gnavus group)* | | rs2909242 | C | A | 0.386 | -0.091 | 0.018 | 24.9 | 7.41E-07 | 0.004 | 0.019 | 0.815 | |
| *Ruminococcus (gnavus group)* | | rs3124783 | A | G | 0.120 | -0.116 | 0.025 | 18.1 | 2.67E-06 | 0.029 | 0.026 | 0.269 | |
| *Ruminococcus (gnavus group)* | | rs4388134 | C | T | 0.246 | -0.090 | 0.020 | 19.3 | 9.12E-06 | -0.035 | 0.020 | 0.072 | |
| *Ruminococcus (gnavus group)* | | rs62167033 | T | C | 0.071 | 0.185 | 0.040 | 28.6 | 3.50E-06 | -0.023 | 0.044 | 0.602 | |
| *Ruminococcus (gnavus group)* | | rs78399089 | T | C | 0.083 | 0.144 | 0.033 | 20.0 | 6.63E-06 | -0.009 | 0.028 | 0.741 | |
| *Ruminococcus (gnavus group)* | | rs934940 | A | C | 0.185 | -0.105 | 0.023 | 21.1 | 2.74E-06 | 0.004 | 0.026 | 0.889 | |
| *Ruminococcus (gnavus group)* | | rs9872758 | T | C | 0.477 | 0.085 | 0.018 | 22.8 | 1.66E-06 | -0.005 | 0.018 | 0.776 | |
| *Ruminococcus (torques group)* | | rs10967781 | C | A | 0.318 | 0.051 | 0.011 | 19.9 | 8.37E-06 | -0.010 | 0.019 | 0.608 | |
| *Ruminococcus (torques group)* | | rs12434631 | A | G | 0.140 | 0.075 | 0.015 | 23.4 | 2.77E-06 | 0.006 | 0.029 | 0.823 | |
| *Ruminococcus (torques group)* | | rs1475330 | T | C | 0.301 | 0.052 | 0.012 | 20.0 | 8.13E-06 | -0.012 | 0.021 | 0.564 | |
| *Ruminococcus (torques group)* | | rs35866622 | T | C | 0.440 | -0.061 | 0.011 | 31.9 | 2.21E-08 | 0.038 | 0.019 | 0.041 | |
| *Ruminococcus (torques group)* | | rs4073731 | T | C | 0.176 | 0.065 | 0.014 | 22.0 | 4.05E-06 | 0.001 | 0.024 | 0.983 | |
| *Ruminococcus (torques group)* | | rs77034621 | T | G | 0.053 | -0.152 | 0.034 | 19.7 | 6.07E-06 | 0.107 | 0.067 | 0.110 | |
| *Ruminococcus (torques group)* | | rs8080469 | G | A | 0.519 | 0.049 | 0.011 | 21.4 | 3.50E-06 | 0.014 | 0.018 | 0.420 | |
| *Ruminococcus (torques group)* | | rs8141465 | A | G | 0.543 | 0.048 | 0.011 | 20.4 | 9.65E-06 | 0.007 | 0.018 | 0.720 | |
| *Sellimonas* | | rs113379006 | T | C | 0.168 | -0.163 | 0.036 | 23.5 | 7.21E-06 | 0.006 | 0.023 | 0.810 | |
| *Sellimonas* | | rs13417181 | T | C | 0.216 | 0.167 | 0.034 | 29.8 | 7.62E-07 | 0.023 | 0.021 | 0.280 | |
| *Sellimonas* | | rs2016057 | A | C | 0.391 | -0.126 | 0.026 | 24.0 | 1.03E-06 | 0.010 | 0.018 | 0.593 | |
| *Sellimonas* | | rs2187447 | A | C | 0.061 | 0.243 | 0.053 | 21.4 | 3.98E-06 | -0.042 | 0.037 | 0.255 | |
| *Sellimonas* | | rs2371572 | A | C | 0.549 | 0.127 | 0.025 | 25.5 | 4.46E-07 | 0.006 | 0.018 | 0.753 | |
| *Sellimonas* | | rs41816 | A | G | 0.252 | 0.132 | 0.029 | 20.9 | 8.39E-06 | 0.020 | 0.019 | 0.294 | |
| *Sellimonas* | | rs4600608 | A | G | 0.239 | -0.137 | 0.030 | 21.7 | 4.95E-06 | -0.002 | 0.022 | 0.945 | |
| *Sellimonas* | | rs553697 | T | C | 0.162 | -0.154 | 0.034 | 20.4 | 6.13E-06 | 0.030 | 0.023 | 0.186 | |
| *Sellimonas* | | rs56203279 | T | C | 0.330 | -0.124 | 0.027 | 21.6 | 3.72E-06 | 0.006 | 0.019 | 0.755 | |
| *Senegalimassilia* | | rs10036909 | C | T | 0.070 | 0.186 | 0.040 | 22.4 | 8.05E-06 | -0.035 | 0.045 | 0.431 | |
| *Senegalimassilia* | | rs11787826 | C | A | 0.428 | 0.081 | 0.017 | 16.2 | 2.63E-06 | -0.005 | 0.018 | 0.789 | |
| *Senegalimassilia* | | rs1990708 | A | C | 0.126 | -0.110 | 0.025 | 13.3 | 8.91E-06 | -0.001 | 0.032 | 0.985 | |
| *Senegalimassilia* | | rs2017373 | C | T | 0.374 | 0.078 | 0.018 | 14.3 | 9.50E-06 | -0.006 | 0.018 | 0.730 | |
| *Senegalimassilia* | | rs7225245 | G | A | 0.458 | 0.079 | 0.017 | 15.6 | 4.18E-06 | -0.009 | 0.018 | 0.616 | |
| *Slackia* | | rs10409783 | A | G | 0.283 | 0.095 | 0.021 | 21.9 | 7.70E-06 | -0.016 | 0.020 | 0.410 | |
| *Slackia* | | rs12440440 | A | G | 0.309 | 0.090 | 0.019 | 20.7 | 2.63E-06 | -0.013 | 0.019 | 0.487 | |
| *Slackia* | | rs16894137 | C | T | 0.143 | -0.123 | 0.026 | 22.0 | 2.71E-06 | -0.014 | 0.026 | 0.604 | |
| *Slackia* | | rs35156985 | T | C | 0.064 | -0.156 | 0.035 | 17.2 | 8.06E-06 | -0.063 | 0.044 | 0.153 | |
| *Slackia* | | rs4492265 | A | G | 0.310 | -0.091 | 0.019 | 20.9 | 2.41E-06 | -0.003 | 0.019 | 0.891 | |
| *Slackia* | | rs8901 | C | T | 0.371 | 0.093 | 0.019 | 24.3 | 6.07E-07 | 0.041 | 0.019 | 0.032 | |
| *Streptococcus* | | rs10028567 | C | T | 0.082 | -0.092 | 0.019 | 20.7 | 7.30E-06 | -0.042 | 0.027 | 0.114 | |
| *Streptococcus* | | rs10448310 | A | G | 0.430 | -0.052 | 0.011 | 21.5 | 3.31E-06 | 0.004 | 0.018 | 0.846 | |
| *Streptococcus* | | rs11110281 | T | C | 0.068 | -0.138 | 0.023 | 39.0 | 2.58E-09 | -0.028 | 0.041 | 0.498 | |
| *Streptococcus* | | rs11720390 | G | A | 0.075 | 0.107 | 0.023 | 25.8 | 3.59E-06 | 0.034 | 0.036 | 0.354 | |
| *Streptococcus* | | rs11764382 | A | G | 0.191 | -0.070 | 0.014 | 24.4 | 1.29E-06 | -0.102 | 0.025 | 0.000 | |
| *Streptococcus* | | rs17708276 | A | G | 0.128 | -0.079 | 0.017 | 23.0 | 3.04E-06 | -0.004 | 0.030 | 0.891 | |
| *Streptococcus* | | rs1918540 | G | A | 0.253 | 0.060 | 0.013 | 21.9 | 2.44E-06 | 0.040 | 0.023 | 0.076 | |
| *Streptococcus* | | rs2370083 | G | T | 0.089 | -0.082 | 0.019 | 17.6 | 9.75E-06 | 0.029 | 0.036 | 0.426 | |
| *Streptococcus* | | rs4968759 | A | G | 0.426 | -0.052 | 0.011 | 21.2 | 3.78E-06 | 0.026 | 0.018 | 0.139 | |
| *Streptococcus* | | rs57646748 | G | A | 0.068 | -0.091 | 0.020 | 17.0 | 5.48E-06 | 0.018 | 0.045 | 0.688 | |
| *Streptococcus* | | rs6806351 | T | C | 0.209 | -0.063 | 0.014 | 21.7 | 4.94E-06 | 0.028 | 0.021 | 0.192 | |
| *Streptococcus* | | rs71481756 | T | G | 0.099 | 0.093 | 0.021 | 25.4 | 6.51E-06 | -0.021 | 0.036 | 0.562 | |
| *Streptococcus* | | rs7916711 | A | G | 0.065 | 0.103 | 0.022 | 20.9 | 2.72E-06 | 0.017 | 0.025 | 0.515 | |
| *Streptococcus* | | rs9903102 | C | A | 0.164 | -0.071 | 0.016 | 22.5 | 4.18E-06 | 0.008 | 0.022 | 0.707 | |
| *Subdoligranulum* | | rs10065321 | T | C | 0.368 | -0.051 | 0.011 | 21.5 | 2.10E-06 | 0.003 | 0.018 | 0.867 | |
| *Subdoligranulum* | | rs10497836 | C | T | 0.280 | -0.052 | 0.012 | 19.5 | 8.38E-06 | 0.006 | 0.022 | 0.779 | |
| *Subdoligranulum* | | rs1667315 | G | A | 0.437 | 0.049 | 0.011 | 20.4 | 6.72E-06 | 0.016 | 0.018 | 0.369 | |
| *Subdoligranulum* | | rs2114677 | C | T | 0.056 | -0.104 | 0.023 | 20.1 | 2.72E-06 | 0.025 | 0.027 | 0.353 | |
| *Subdoligranulum* | | rs2171249 | C | T | 0.074 | 0.107 | 0.023 | 27.4 | 4.51E-06 | 0.053 | 0.034 | 0.121 | |
| *Subdoligranulum* | | rs35940633 | G | A | 0.363 | -0.051 | 0.011 | 21.3 | 4.22E-06 | -0.001 | 0.019 | 0.952 | |
| *Subdoligranulum* | | rs3761728 | T | G | 0.243 | -0.054 | 0.012 | 19.1 | 3.87E-06 | 0.034 | 0.020 | 0.092 | |
| *Subdoligranulum* | | rs4347804 | A | G | 0.042 | 0.166 | 0.036 | 38.8 | 2.18E-06 | -0.107 | 0.050 | 0.031 | |
| *Subdoligranulum* | | rs6555306 | T | C | 0.122 | -0.074 | 0.016 | 20.7 | 2.81E-06 | 0.015 | 0.025 | 0.561 | |
| *Subdoligranulum* | | rs75158211 | T | C | 0.111 | -0.072 | 0.016 | 18.2 | 7.52E-06 | 0.006 | 0.025 | 0.816 | |
| *Subdoligranulum* | | rs76528319 | G | T | 0.040 | -0.143 | 0.031 | 27.6 | 7.41E-06 | 0.036 | 0.032 | 0.256 | |
| *Sutterella* | | rs1145877 | A | G | 0.159 | -0.074 | 0.016 | 19.2 | 7.20E-06 | 0.023 | 0.025 | 0.370 | |
| *Sutterella* | | rs11591622 | T | G | 0.216 | -0.069 | 0.015 | 21.3 | 6.50E-06 | -0.036 | 0.024 | 0.127 | |
| *Sutterella* | | rs13173038 | A | G | 0.221 | -0.072 | 0.015 | 23.5 | 2.73E-06 | -0.003 | 0.020 | 0.869 | |
| *Sutterella* | | rs143438747 | T | C | 0.055 | -0.146 | 0.031 | 29.2 | 3.28E-06 | 0.038 | 0.033 | 0.257 | |
| *Sutterella* | | rs2050185 | G | A | 0.378 | 0.058 | 0.013 | 20.6 | 7.97E-06 | 0.032 | 0.018 | 0.078 | |
| *Sutterella* | | rs2321387 | G | A | 0.530 | -0.059 | 0.012 | 23.2 | 1.87E-06 | -0.031 | 0.018 | 0.081 | |
| *Sutterella* | | rs2613606 | C | T | 0.394 | -0.056 | 0.012 | 19.6 | 7.20E-06 | 0.001 | 0.018 | 0.942 | |
| *Sutterella* | | rs607327 | C | T | 0.377 | 0.058 | 0.013 | 20.8 | 6.63E-06 | -0.015 | 0.018 | 0.409 | |
| *Sutterella* | | rs62501473 | G | A | 0.229 | 0.069 | 0.015 | 22.6 | 5.52E-06 | -0.004 | 0.020 | 0.824 | |
| *Sutterella* | | rs7499539 | A | G | 0.323 | 0.062 | 0.013 | 22.1 | 2.36E-06 | -0.030 | 0.020 | 0.134 | |
| *Sutterella* | | rs7638039 | T | C | 0.233 | 0.065 | 0.014 | 19.7 | 8.66E-06 | -0.011 | 0.020 | 0.603 | |
| *Sutterella* | | rs9350083 | T | G | 0.299 | -0.059 | 0.013 | 19.6 | 8.23E-06 | 0.012 | 0.018 | 0.521 | |
| *Terrisporobacter* | | rs1883097 | C | T | 0.056 | 0.226 | 0.045 | 23.9 | 4.16E-07 | 0.010 | 0.046 | 0.826 | |
| *Terrisporobacter* | | rs2569953 | A | C | 0.420 | -0.078 | 0.017 | 13.0 | 8.95E-06 | 0.013 | 0.018 | 0.472 | |
| *Terrisporobacter* | | rs2872237 | C | A | 0.434 | -0.081 | 0.018 | 14.4 | 3.97E-06 | 0.022 | 0.018 | 0.218 | |
| *Terrisporobacter* | | rs58405430 | G | T | 0.115 | 0.135 | 0.030 | 16.4 | 7.94E-06 | -0.058 | 0.037 | 0.117 | |
| *Terrisporobacter* | | rs7184125 | T | C | 0.233 | 0.091 | 0.021 | 13.2 | 8.48E-06 | -0.022 | 0.020 | 0.277 | |
| *Turicibacter* | | rs11054680 | T | C | 0.096 | -0.105 | 0.023 | 17.1 | 2.31E-06 | 0.017 | 0.023 | 0.463 | |
| *Turicibacter* | | rs11666533 | C | T | 0.080 | -0.112 | 0.025 | 16.3 | 7.37E-06 | 0.001 | 0.032 | 0.973 | |
| *Turicibacter* | | rs12603364 | T | C | 0.107 | 0.111 | 0.023 | 21.1 | 8.67E-07 | 0.023 | 0.024 | 0.346 | |
| *Turicibacter* | | rs149744580 | A | G | 0.053 | 0.170 | 0.032 | 25.8 | 7.01E-08 | 0.009 | 0.039 | 0.829 | |
| *Turicibacter* | | rs2834977 | T | C | 0.173 | -0.096 | 0.021 | 23.6 | 3.96E-06 | 0.026 | 0.024 | 0.288 | |
| *Turicibacter* | | rs2952020 | G | A | 0.223 | -0.076 | 0.017 | 17.8 | 5.63E-06 | -0.001 | 0.021 | 0.979 | |
| *Turicibacter* | | rs3734633 | G | A | 0.097 | -0.121 | 0.027 | 23.0 | 5.32E-06 | 0.043 | 0.038 | 0.258 | |
| *Turicibacter* | | rs4869133 | G | A | 0.089 | 0.131 | 0.027 | 24.9 | 2.55E-06 | 0.022 | 0.023 | 0.347 | |
| *Turicibacter* | | rs55756211 | T | C | 0.116 | -0.115 | 0.024 | 24.4 | 2.81E-06 | 0.002 | 0.034 | 0.945 | |
| *Turicibacter* | | rs7199484 | G | A | 0.332 | -0.073 | 0.016 | 21.2 | 5.77E-06 | 0.005 | 0.019 | 0.792 | |
| *Tyzzerella 3* | | rs10898797 | C | T | 0.157 | 0.122 | 0.027 | 21.4 | 8.85E-06 | -0.035 | 0.028 | 0.208 | |
| *Tyzzerella 3* | | rs112102233 | A | G | 0.067 | -0.216 | 0.048 | 31.5 | 6.18E-06 | 0.094 | 0.042 | 0.025 | |
| *Tyzzerella 3* | | rs1232220 | G | T | 0.126 | -0.144 | 0.032 | 24.7 | 7.91E-06 | -0.028 | 0.030 | 0.344 | |
| *Tyzzerella 3* | | rs17706273 | T | C | 0.152 | -0.140 | 0.027 | 27.5 | 5.88E-07 | 0.018 | 0.033 | 0.578 | |
| *Tyzzerella 3* | | rs191093 | G | A | 0.080 | 0.159 | 0.035 | 20.0 | 6.76E-06 | -0.001 | 0.029 | 0.987 | |
| *Tyzzerella 3* | | rs4904512 | T | C | 0.199 | -0.117 | 0.025 | 23.6 | 3.09E-06 | 0.005 | 0.026 | 0.856 | |
| *Tyzzerella 3* | | rs55799124 | A | G | 0.207 | -0.114 | 0.024 | 23.2 | 1.34E-06 | -0.017 | 0.020 | 0.390 | |
| *Tyzzerella 3* | | rs67476743 | T | G | 0.285 | 0.132 | 0.022 | 38.6 | 3.74E-09 | 0.008 | 0.020 | 0.694 | |
| *Tyzzerella 3* | | rs6920448 | C | T | 0.115 | -0.141 | 0.031 | 22.0 | 4.15E-06 | 0.020 | 0.030 | 0.504 | |
| *Tyzzerella 3* | | rs7019909 | T | C | 0.101 | 0.144 | 0.030 | 20.5 | 1.76E-06 | 0.031 | 0.027 | 0.247 | |
| *Tyzzerella 3* | | rs7333521 | T | C | 0.043 | -0.207 | 0.045 | 19.0 | 4.88E-06 | -0.094 | 0.050 | 0.057 | |
| *Tyzzerella 3* | | rs75091807 | G | T | 0.091 | -0.185 | 0.038 | 30.5 | 1.71E-06 | -0.021 | 0.037 | 0.567 | |
| *Tyzzerella 3* | | rs7561370 | T | C | 0.122 | 0.131 | 0.029 | 20.0 | 1.52E-06 | -0.014 | 0.025 | 0.564 | |
| *Veillonella* | | rs1882878 | A | G | 0.306 | -0.077 | 0.016 | 21.2 | 2.98E-06 | -0.002 | 0.019 | 0.922 | |
| *Veillonella* | | rs2013594 | T | C | 0.358 | -0.072 | 0.016 | 20.1 | 3.42E-06 | 0.008 | 0.018 | 0.649 | |
| *Veillonella* | | rs55807413 | A | G | 0.097 | 0.107 | 0.024 | 17.1 | 5.51E-06 | 0.011 | 0.029 | 0.695 | |
| *Veillonella* | | rs62376424 | C | T | 0.334 | -0.076 | 0.016 | 21.8 | 3.65E-06 | -0.039 | 0.019 | 0.040 | |
| *Veillonella* | | rs6656807 | A | G | 0.425 | 0.070 | 0.015 | 20.4 | 5.50E-06 | 0.026 | 0.018 | 0.147 | |
| *Veillonella* | | rs742016 | A | G | 0.424 | -0.069 | 0.015 | 19.5 | 4.66E-06 | 0.014 | 0.019 | 0.450 | |
| *Victivallis* | | rs11899949 | G | A | 0.277 | 0.131 | 0.028 | 22.4 | 2.77E-06 | -0.001 | 0.019 | 0.942 | |
| *Victivallis* | | rs12512543 | A | C | 0.121 | -0.178 | 0.037 | 22.0 | 2.54E-06 | -0.014 | 0.031 | 0.657 | |
| *Victivallis* | | rs173120 | T | C | 0.246 | 0.134 | 0.029 | 21.8 | 7.65E-06 | -0.026 | 0.022 | 0.239 | |
| *Victivallis* | | rs1882775 | A | G | 0.224 | -0.138 | 0.031 | 21.8 | 8.73E-06 | -0.028 | 0.023 | 0.215 | |
| *Victivallis* | | rs2546432 | T | C | 0.466 | -0.111 | 0.025 | 20.1 | 9.93E-06 | 0.009 | 0.018 | 0.604 | |
| *Victivallis* | | rs342302 | A | G | 0.159 | -0.153 | 0.035 | 20.5 | 8.16E-06 | 0.040 | 0.026 | 0.116 | |
| *Victivallis* | | rs4764863 | G | A | 0.500 | 0.122 | 0.025 | 24.3 | 8.22E-07 | -0.025 | 0.018 | 0.156 | |
| *Victivallis* | | rs4895919 | T | C | 0.482 | -0.117 | 0.025 | 22.4 | 2.75E-06 | -0.024 | 0.018 | 0.170 | |
| *Victivallis* | | rs56349194 | A | G | 0.160 | -0.159 | 0.032 | 22.2 | 6.26E-07 | -0.025 | 0.026 | 0.332 | |
| *Victivallis* | | rs911666 | T | C | 0.305 | -0.119 | 0.026 | 19.6 | 7.65E-06 | -0.011 | 0.019 | 0.567 | |

|  | **Table S2 Instrumental variables used in MR analysis of the association between blood lipids and endometriosis.** | | | | | | | | | | | | |
| --- | --- | --- | --- | --- | --- | --- | --- | --- | --- | --- | --- | --- | --- |
| **exposure** | | **SNP** | **Effect allele** | **Other allele** | **EAF** | **Exposure (Bacteria)** | | | | **Outcome (PE)** | | |  |
|  |  |  |  |  |  | **Beta** | **SE** | **F statistics** | **P-value** | **Beta** | **SE** | **P-value** | |
| Apolipoprotein A-1 | | rs10023962 | G | T | 0.817 | 0.018 | 0.003 | 36.7 | 3.80E-12 | -0.035 | 0.027 | 0.198 | |
| Apolipoprotein A-1 | | rs10170658 | T | G | 0.223 | -0.013 | 0.002 | 24.1 | 1.70E-08 | 0.006 | 0.020 | 0.752 | |
| Apolipoprotein A-1 | | rs1037117 | A | G | 0.255 | 0.016 | 0.002 | 38.7 | 9.50E-13 | 0.017 | 0.019 | 0.391 | |
| Apolipoprotein A-1 | | rs10404380 | A | C | 0.605 | -0.026 | 0.002 | 125.1 | 1.60E-37 | 0.030 | 0.018 | 0.089 | |
| Apolipoprotein A-1 | | rs1047891 | A | C | 0.316 | -0.026 | 0.002 | 111.9 | 3.30E-34 | -0.010 | 0.019 | 0.607 | |
| Apolipoprotein A-1 | | rs10489044 | G | A | 0.199 | -0.018 | 0.002 | 42.2 | 1.00E-13 | 0.038 | 0.025 | 0.120 | |
| Apolipoprotein A-1 | | rs10504477 | C | T | 0.412 | -0.012 | 0.002 | 28.0 | 1.20E-09 | 0.005 | 0.018 | 0.782 | |
| Apolipoprotein A-1 | | rs1055582 | T | C | 0.505 | 0.016 | 0.002 | 47.6 | 2.40E-15 | -0.021 | 0.018 | 0.240 | |
| Apolipoprotein A-1 | | rs10745954 | G | A | 0.480 | -0.014 | 0.002 | 40.5 | 2.50E-13 | -0.014 | 0.018 | 0.429 | |
| Apolipoprotein A-1 | | rs10748165 | T | C | 0.504 | 0.011 | 0.002 | 23.8 | 2.10E-08 | -0.031 | 0.018 | 0.076 | |
| Apolipoprotein A-1 | | rs10750766 | A | C | 0.710 | -0.015 | 0.002 | 35.4 | 7.80E-12 | 0.006 | 0.020 | 0.771 | |
| Apolipoprotein A-1 | | rs10752898 | C | T | 0.558 | 0.013 | 0.002 | 30.5 | 1.90E-10 | -0.027 | 0.018 | 0.134 | |
| Apolipoprotein A-1 | | rs10774439 | A | G | 0.815 | 0.016 | 0.003 | 32.1 | 1.30E-10 | -0.021 | 0.021 | 0.305 | |
| Apolipoprotein A-1 | | rs10798615 | G | T | 0.533 | -0.015 | 0.002 | 45.9 | 6.20E-15 | 0.001 | 0.018 | 0.953 | |
| Apolipoprotein A-1 | | rs1086056 | G | T | 0.845 | -0.019 | 0.003 | 36.7 | 3.70E-12 | 0.022 | 0.023 | 0.336 | |
| Apolipoprotein A-1 | | rs10876447 | A | G | 0.154 | -0.019 | 0.003 | 38.5 | 9.30E-13 | 0.050 | 0.026 | 0.056 | |
| Apolipoprotein A-1 | | rs10883451 | C | T | 0.496 | 0.022 | 0.002 | 96.0 | 1.80E-29 | -0.020 | 0.018 | 0.267 | |
| Apolipoprotein A-1 | | rs10917383 | G | A | 0.875 | 0.020 | 0.003 | 35.7 | 5.70E-12 | -0.028 | 0.028 | 0.307 | |
| Apolipoprotein A-1 | | rs11021232 | C | T | 0.181 | -0.016 | 0.003 | 31.6 | 1.30E-10 | -0.017 | 0.023 | 0.457 | |
| Apolipoprotein A-1 | | rs11045172 | C | A | 0.198 | 0.022 | 0.002 | 61.7 | 2.70E-19 | -0.004 | 0.021 | 0.850 | |
| Apolipoprotein A-1 | | rs11057390 | G | T | 0.295 | 0.019 | 0.002 | 59.1 | 1.20E-18 | 0.006 | 0.021 | 0.756 | |
| Apolipoprotein A-1 | | rs11065987 | G | A | 0.416 | -0.014 | 0.002 | 37.9 | 1.50E-12 | -0.028 | 0.018 | 0.119 | |
| Apolipoprotein A-1 | | rs11067231 | A | C | 0.524 | 0.022 | 0.002 | 94.9 | 3.60E-29 | 0.017 | 0.018 | 0.324 | |
| Apolipoprotein A-1 | | rs11089620 | G | C | 0.189 | -0.032 | 0.003 | 124.5 | 1.70E-37 | -0.002 | 0.019 | 0.920 | |
| Apolipoprotein A-1 | | rs111849006 | A | G | 0.181 | 0.015 | 0.003 | 25.2 | 1.70E-08 | -0.013 | 0.024 | 0.588 | |
| Apolipoprotein A-1 | | rs11216920 | G | A | 0.084 | 0.025 | 0.004 | 38.0 | 1.60E-12 | -0.013 | 0.032 | 0.684 | |
| Apolipoprotein A-1 | | rs11226108 | C | G | 0.192 | -0.015 | 0.002 | 26.2 | 4.20E-09 | -0.007 | 0.023 | 0.770 | |
| Apolipoprotein A-1 | | rs112928559 | T | C | 0.040 | -0.037 | 0.005 | 40.7 | 2.60E-12 | 0.004 | 0.075 | 0.957 | |
| Apolipoprotein A-1 | | rs113017476 | A | G | 0.039 | 0.031 | 0.005 | 28.6 | 1.00E-09 | 0.110 | 0.059 | 0.060 | |
| Apolipoprotein A-1 | | rs1132274 | A | C | 0.154 | -0.029 | 0.003 | 84.9 | 3.20E-26 | 0.025 | 0.026 | 0.326 | |
| Apolipoprotein A-1 | | rs11429307 | G | GT | 0.809 | 0.022 | 0.003 | 60.5 | 5.00E-19 | -0.064 | 0.026 | 0.012 | |
| Apolipoprotein A-1 | | rs11481448 | A | AT | 0.497 | 0.011 | 0.002 | 25.9 | 5.90E-09 | -0.014 | 0.018 | 0.443 | |
| Apolipoprotein A-1 | | rs116006942 | A | G | 0.061 | -0.028 | 0.004 | 36.0 | 9.40E-12 | 0.041 | 0.045 | 0.356 | |
| Apolipoprotein A-1 | | rs11632618 | A | G | 0.070 | 0.108 | 0.004 | 595.6 | 1.10E-173 | 0.005 | 0.041 | 0.912 | |
| Apolipoprotein A-1 | | rs11641548 | C | A | 0.440 | -0.013 | 0.002 | 33.1 | 3.60E-11 | -0.013 | 0.019 | 0.505 | |
| Apolipoprotein A-1 | | rs1168124 | T | C | 0.651 | 0.043 | 0.002 | 324.8 | 1.20E-95 | 0.008 | 0.020 | 0.691 | |
| Apolipoprotein A-1 | | rs11691486 | C | T | 0.240 | 0.014 | 0.002 | 28.7 | 8.30E-10 | -0.020 | 0.021 | 0.330 | |
| Apolipoprotein A-1 | | rs117399007 | T | C | 0.043 | -0.029 | 0.005 | 27.3 | 2.50E-09 | -0.050 | 0.042 | 0.236 | |
| Apolipoprotein A-1 | | rs11754773 | G | A | 0.093 | -0.040 | 0.003 | 107.1 | 2.70E-32 | 0.069 | 0.025 | 0.006 | |
| Apolipoprotein A-1 | | rs117739035 | T | G | 0.036 | -0.031 | 0.005 | 25.4 | 6.60E-09 | 0.003 | 0.039 | 0.929 | |
| Apolipoprotein A-1 | | rs117853493 | A | G | 0.015 | 0.057 | 0.008 | 37.5 | 1.70E-12 | -0.019 | 0.056 | 0.741 | |
| Apolipoprotein A-1 | | rs11870735 | T | C | 0.181 | -0.015 | 0.003 | 27.1 | 2.20E-09 | -0.023 | 0.026 | 0.380 | |
| Apolipoprotein A-1 | | rs12229372 | C | T | 0.103 | 0.030 | 0.003 | 64.2 | 2.40E-19 | -0.010 | 0.025 | 0.674 | |
| Apolipoprotein A-1 | | rs12273363 | C | T | 0.206 | -0.020 | 0.002 | 50.1 | 3.90E-16 | -0.030 | 0.021 | 0.144 | |
| Apolipoprotein A-1 | | rs12436555 | A | G | 0.173 | -0.015 | 0.003 | 24.2 | 1.80E-08 | 0.045 | 0.028 | 0.109 | |
| Apolipoprotein A-1 | | rs12462109 | T | C | 0.287 | -0.013 | 0.002 | 28.9 | 6.40E-10 | 0.007 | 0.019 | 0.719 | |
| Apolipoprotein A-1 | | rs1260326 | C | T | 0.605 | -0.026 | 0.002 | 125.3 | 4.50E-38 | -0.009 | 0.018 | 0.633 | |
| Apolipoprotein A-1 | | rs12740374 | T | G | 0.221 | 0.044 | 0.002 | 265.8 | 1.10E-78 | -0.015 | 0.021 | 0.490 | |
| Apolipoprotein A-1 | | rs12898210 | G | T | 0.122 | -0.049 | 0.003 | 202.9 | 6.40E-60 | 0.010 | 0.029 | 0.720 | |
| Apolipoprotein A-1 | | rs12943517 | A | G | 0.311 | -0.012 | 0.002 | 23.7 | 2.40E-08 | -0.003 | 0.019 | 0.891 | |
| Apolipoprotein A-1 | | rs12987470 | T | A | 0.295 | 0.012 | 0.002 | 23.7 | 2.20E-08 | 0.055 | 0.019 | 0.003 | |
| Apolipoprotein A-1 | | rs13059175 | T | C | 0.300 | 0.015 | 0.002 | 36.3 | 3.60E-12 | 0.006 | 0.022 | 0.800 | |
| Apolipoprotein A-1 | | rs13107325 | T | C | 0.075 | -0.072 | 0.004 | 281.1 | 1.70E-82 | 0.080 | 0.075 | 0.290 | |
| Apolipoprotein A-1 | | rs13108218 | G | A | 0.615 | -0.013 | 0.002 | 33.1 | 5.30E-11 | -0.012 | 0.019 | 0.538 | |
| Apolipoprotein A-1 | | rs1318175 | T | C | 0.160 | -0.078 | 0.003 | 644.8 | 3.40E-186 | 0.029 | 0.023 | 0.209 | |
| Apolipoprotein A-1 | | rs13326165 | G | A | 0.795 | -0.020 | 0.002 | 48.9 | 8.00E-16 | 0.013 | 0.023 | 0.560 | |
| Apolipoprotein A-1 | | rs13379043 | C | T | 0.280 | 0.022 | 0.002 | 75.3 | 1.50E-22 | -0.021 | 0.022 | 0.341 | |
| Apolipoprotein A-1 | | rs13402475 | G | C | 0.815 | -0.024 | 0.003 | 68.3 | 1.00E-20 | -0.016 | 0.019 | 0.406 | |
| Apolipoprotein A-1 | | rs1395221 | T | G | 0.398 | -0.013 | 0.002 | 33.2 | 3.90E-11 | 0.027 | 0.018 | 0.129 | |
| Apolipoprotein A-1 | | rs1400362 | C | T | 0.741 | 0.013 | 0.002 | 23.6 | 3.30E-08 | 0.027 | 0.021 | 0.204 | |
| Apolipoprotein A-1 | | rs1406982 | A | T | 0.314 | -0.014 | 0.002 | 34.5 | 1.40E-11 | -0.008 | 0.019 | 0.653 | |
| Apolipoprotein A-1 | | rs141368429 | T | C | 0.056 | -0.057 | 0.005 | 134.8 | 1.90E-35 | 0.089 | 0.057 | 0.115 | |
| Apolipoprotein A-1 | | rs144033177 | C | A | 0.016 | -0.049 | 0.008 | 29.9 | 8.90E-10 | -0.101 | 0.071 | 0.150 | |
| Apolipoprotein A-1 | | rs144311893 | T | C | 0.022 | 0.123 | 0.007 | 254.7 | 1.10E-70 | 0.091 | 0.103 | 0.378 | |
| Apolipoprotein A-1 | | rs144475339 | C | G | 0.011 | 0.058 | 0.010 | 27.8 | 2.90E-08 | 0.035 | 0.101 | 0.733 | |
| Apolipoprotein A-1 | | rs1446585 | G | A | 0.244 | 0.019 | 0.002 | 50.2 | 9.20E-17 | -0.027 | 0.018 | 0.139 | |
| Apolipoprotein A-1 | | rs145947882 | C | A | 0.026 | -0.136 | 0.006 | 372.1 | 6.90E-105 | 0.027 | 0.051 | 0.597 | |
| Apolipoprotein A-1 | | rs147772065 | C | G | 0.035 | 0.031 | 0.005 | 26.1 | 6.00E-09 | -0.021 | 0.057 | 0.713 | |
| Apolipoprotein A-1 | | rs148827772 | G | A | 0.023 | -0.057 | 0.007 | 56.6 | 3.70E-16 | 0.066 | 0.059 | 0.263 | |
| Apolipoprotein A-1 | | rs150237291 | C | T | 0.022 | 0.049 | 0.007 | 41.1 | 2.30E-13 | 0.003 | 0.052 | 0.951 | |
| Apolipoprotein A-1 | | rs150483923 | A | C | 0.226 | 0.020 | 0.002 | 53.6 | 5.30E-17 | -0.013 | 0.023 | 0.567 | |
| Apolipoprotein A-1 | | rs150844304 | C | A | 0.026 | -0.075 | 0.006 | 112.8 | 2.00E-34 | 0.117 | 0.124 | 0.344 | |
| Apolipoprotein A-1 | | rs1540687 | T | A | 0.674 | 0.012 | 0.002 | 23.5 | 2.80E-08 | 0.063 | 0.018 | 0.001 | |
| Apolipoprotein A-1 | | rs1601933 | T | C | 0.467 | -0.084 | 0.002 | 1393.6 | 1.00E-200 | -0.007 | 0.018 | 0.679 | |
| Apolipoprotein A-1 | | rs1661052 | A | G | 0.908 | 0.029 | 0.003 | 56.4 | 6.20E-18 | 0.008 | 0.034 | 0.819 | |
| Apolipoprotein A-1 | | rs17008972 | A | G | 0.124 | 0.019 | 0.003 | 31.7 | 1.00E-10 | 0.004 | 0.022 | 0.859 | |
| Apolipoprotein A-1 | | rs17138358 | C | G | 0.399 | -0.026 | 0.002 | 129.1 | 6.60E-39 | 0.014 | 0.019 | 0.453 | |
| Apolipoprotein A-1 | | rs17326656 | T | G | 0.239 | -0.018 | 0.002 | 47.3 | 3.10E-15 | -0.036 | 0.024 | 0.127 | |
| Apolipoprotein A-1 | | rs174566 | G | A | 0.350 | -0.035 | 0.002 | 218.1 | 1.00E-64 | 0.003 | 0.018 | 0.877 | |
| Apolipoprotein A-1 | | rs17883513 | G | A | 0.034 | 0.030 | 0.005 | 23.4 | 2.70E-08 | 0.004 | 0.048 | 0.929 | |
| Apolipoprotein A-1 | | rs1789896 | A | G | 0.513 | 0.015 | 0.002 | 43.8 | 2.60E-14 | 0.010 | 0.018 | 0.563 | |
| Apolipoprotein A-1 | | rs1800961 | T | C | 0.031 | -0.149 | 0.006 | 526.2 | 1.30E-152 | -0.002 | 0.042 | 0.961 | |
| Apolipoprotein A-1 | | rs1852922 | A | G | 0.690 | 0.012 | 0.002 | 25.7 | 6.40E-09 | 0.015 | 0.019 | 0.430 | |
| Apolipoprotein A-1 | | rs1862205 | A | G | 0.405 | 0.011 | 0.002 | 23.2 | 3.20E-08 | 0.020 | 0.018 | 0.257 | |
| Apolipoprotein A-1 | | rs188502504 | C | T | 0.036 | -0.029 | 0.005 | 23.4 | 3.00E-08 | 0.032 | 0.035 | 0.359 | |
| Apolipoprotein A-1 | | rs1919309 | C | T | 0.509 | 0.012 | 0.002 | 26.9 | 2.80E-09 | 0.033 | 0.018 | 0.071 | |
| Apolipoprotein A-1 | | rs1970811 | C | T | 0.458 | -0.012 | 0.002 | 26.3 | 4.00E-09 | 0.021 | 0.018 | 0.244 | |
| Apolipoprotein A-1 | | rs2066714 | C | T | 0.129 | 0.054 | 0.003 | 259.9 | 7.90E-77 | -0.010 | 0.030 | 0.746 | |
| Apolipoprotein A-1 | | rs2068888 | A | G | 0.450 | 0.013 | 0.002 | 35.1 | 9.80E-12 | -0.007 | 0.018 | 0.697 | |
| Apolipoprotein A-1 | | rs2071379 | G | A | 0.595 | -0.021 | 0.002 | 87.1 | 8.10E-27 | -0.008 | 0.018 | 0.663 | |
| Apolipoprotein A-1 | | rs2111705 | A | G | 0.544 | -0.015 | 0.002 | 43.5 | 3.70E-14 | 0.036 | 0.018 | 0.040 | |
| Apolipoprotein A-1 | | rs2159935 | A | G | 0.490 | 0.011 | 0.002 | 25.7 | 5.60E-09 | -0.005 | 0.018 | 0.768 | |
| Apolipoprotein A-1 | | rs2245095 | T | C | 0.089 | -0.026 | 0.003 | 41.7 | 1.10E-13 | 0.028 | 0.025 | 0.263 | |
| Apolipoprotein A-1 | | rs2247355 | T | C | 0.183 | 0.018 | 0.003 | 36.9 | 3.00E-12 | -0.008 | 0.020 | 0.684 | |
| Apolipoprotein A-1 | | rs2256720 | C | T | 0.444 | 0.012 | 0.002 | 28.8 | 7.40E-10 | -0.003 | 0.019 | 0.875 | |
| Apolipoprotein A-1 | | rs2269434 | C | T | 0.345 | 0.035 | 0.002 | 213.1 | 1.70E-63 | 0.010 | 0.018 | 0.592 | |
| Apolipoprotein A-1 | | rs2281719 | T | C | 0.612 | 0.053 | 0.002 | 533.9 | 1.50E-155 | -0.024 | 0.018 | 0.176 | |
| Apolipoprotein A-1 | | rs2294915 | T | C | 0.232 | -0.020 | 0.002 | 55.9 | 1.00E-17 | -0.007 | 0.020 | 0.709 | |
| Apolipoprotein A-1 | | rs2297409 | A | G | 0.195 | -0.037 | 0.002 | 165.5 | 1.50E-49 | 0.007 | 0.027 | 0.789 | |
| Apolipoprotein A-1 | | rs2298214 | A | C | 0.577 | -0.013 | 0.002 | 33.2 | 4.70E-11 | 0.006 | 0.018 | 0.718 | |
| Apolipoprotein A-1 | | rs2298624 | T | C | 0.132 | 0.032 | 0.003 | 92.3 | 2.00E-28 | -0.017 | 0.023 | 0.458 | |
| Apolipoprotein A-1 | | rs2302263 | T | C | 0.089 | -0.029 | 0.003 | 51.6 | 1.50E-16 | -0.056 | 0.026 | 0.034 | |
| Apolipoprotein A-1 | | rs2305696 | T | C | 0.486 | -0.018 | 0.002 | 66.7 | 3.90E-21 | 0.048 | 0.018 | 0.007 | |
| Apolipoprotein A-1 | | rs235314 | T | C | 0.532 | -0.021 | 0.002 | 90.3 | 1.50E-27 | 0.004 | 0.018 | 0.804 | |
| Apolipoprotein A-1 | | rs2362541 | G | T | 0.508 | -0.011 | 0.002 | 24.9 | 9.10E-09 | 0.026 | 0.018 | 0.146 | |
| Apolipoprotein A-1 | | rs2395158 | G | A | 0.169 | -0.034 | 0.003 | 131.1 | 1.00E-39 | 0.002 | 0.027 | 0.949 | |
| Apolipoprotein A-1 | | rs2419605 | G | A | 0.148 | -0.031 | 0.003 | 97.0 | 2.20E-29 | -0.016 | 0.026 | 0.554 | |
| Apolipoprotein A-1 | | rs2424993 | C | G | 0.698 | -0.016 | 0.002 | 43.2 | 4.90E-14 | -0.023 | 0.019 | 0.227 | |
| Apolipoprotein A-1 | | rs2494747 | T | G | 0.616 | -0.032 | 0.002 | 193.3 | 4.30E-57 | 0.009 | 0.018 | 0.616 | |
| Apolipoprotein A-1 | | rs2520096 | G | A | 0.270 | 0.014 | 0.002 | 30.6 | 2.30E-10 | 0.032 | 0.018 | 0.078 | |
| Apolipoprotein A-1 | | rs2540951 | G | A | 0.376 | 0.012 | 0.002 | 27.1 | 2.00E-09 | -0.002 | 0.019 | 0.900 | |
| Apolipoprotein A-1 | | rs2544654 | T | G | 0.715 | 0.013 | 0.002 | 26.4 | 3.70E-09 | -0.001 | 0.021 | 0.970 | |
| Apolipoprotein A-1 | | rs254559 | A | C | 0.405 | -0.017 | 0.002 | 55.3 | 1.40E-17 | 0.025 | 0.018 | 0.172 | |
| Apolipoprotein A-1 | | rs2642438 | G | A | 0.703 | 0.029 | 0.002 | 139.5 | 3.30E-42 | -0.008 | 0.019 | 0.697 | |
| Apolipoprotein A-1 | | rs267738 | G | T | 0.219 | 0.034 | 0.002 | 159.2 | 7.50E-48 | 0.019 | 0.022 | 0.390 | |
| Apolipoprotein A-1 | | rs2726112 | G | A | 0.454 | -0.014 | 0.002 | 37.4 | 2.00E-12 | -0.026 | 0.018 | 0.148 | |
| Apolipoprotein A-1 | | rs2740488 | C | A | 0.266 | -0.080 | 0.002 | 993.9 | 1.00E-200 | 0.014 | 0.022 | 0.528 | |
| Apolipoprotein A-1 | | rs2803619 | C | G | 0.725 | -0.037 | 0.002 | 220.6 | 2.10E-65 | -0.017 | 0.019 | 0.363 | |
| Apolipoprotein A-1 | | rs2804894 | A | G | 0.735 | 0.016 | 0.002 | 40.5 | 4.80E-13 | 0.010 | 0.021 | 0.631 | |
| Apolipoprotein A-1 | | rs28362901 | A | C | 0.088 | -0.025 | 0.003 | 38.5 | 1.00E-12 | -0.030 | 0.028 | 0.275 | |
| Apolipoprotein A-1 | | rs286965 | C | T | 0.632 | -0.016 | 0.002 | 47.0 | 3.20E-15 | 0.007 | 0.018 | 0.694 | |
| Apolipoprotein A-1 | | rs28824216 | T | C | 0.022 | 0.038 | 0.007 | 24.0 | 2.10E-08 | -0.114 | 0.095 | 0.232 | |
| Apolipoprotein A-1 | | rs2925979 | C | T | 0.700 | 0.029 | 0.002 | 134.5 | 6.10E-41 | -0.013 | 0.019 | 0.490 | |
| Apolipoprotein A-1 | | rs2943645 | T | C | 0.647 | -0.034 | 0.002 | 206.2 | 1.80E-61 | 0.034 | 0.018 | 0.060 | |
| Apolipoprotein A-1 | | rs2963468 | G | A | 0.235 | -0.017 | 0.002 | 42.7 | 9.00E-14 | 0.003 | 0.026 | 0.900 | |
| Apolipoprotein A-1 | | rs2965169 | C | A | 0.389 | 0.019 | 0.002 | 66.3 | 7.60E-21 | 0.015 | 0.019 | 0.414 | |
| Apolipoprotein A-1 | | rs2972166 | A | G | 0.275 | 0.015 | 0.002 | 35.2 | 1.10E-11 | -0.010 | 0.019 | 0.593 | |
| Apolipoprotein A-1 | | rs2999141 | C | A | 0.276 | -0.012 | 0.002 | 23.1 | 4.90E-08 | -0.017 | 0.020 | 0.399 | |
| Apolipoprotein A-1 | | rs326 | G | A | 0.294 | 0.083 | 0.002 | 1119.9 | 1.00E-200 | 0.002 | 0.020 | 0.913 | |
| Apolipoprotein A-1 | | rs33042 | A | G | 0.246 | 0.017 | 0.002 | 40.5 | 2.50E-13 | -0.006 | 0.020 | 0.761 | |
| Apolipoprotein A-1 | | rs34045894 | A | G | 0.156 | -0.015 | 0.003 | 22.6 | 4.80E-08 | -0.014 | 0.023 | 0.546 | |
| Apolipoprotein A-1 | | rs34138141 | T | G | 0.281 | -0.018 | 0.002 | 52.0 | 1.30E-16 | -0.024 | 0.021 | 0.262 | |
| Apolipoprotein A-1 | | rs34180494 | C | A | 0.274 | -0.013 | 0.002 | 25.9 | 4.80E-09 | -0.019 | 0.018 | 0.298 | |
| Apolipoprotein A-1 | | rs34397747 | C | T | 0.084 | -0.039 | 0.004 | 93.9 | 2.20E-28 | -0.063 | 0.033 | 0.060 | |
| Apolipoprotein A-1 | | rs34642857 | C | T | 0.251 | -0.013 | 0.002 | 26.3 | 4.50E-09 | 0.006 | 0.023 | 0.800 | |
| Apolipoprotein A-1 | | rs34767118 | G | A | 0.326 | 0.017 | 0.002 | 49.5 | 8.40E-16 | -0.008 | 0.019 | 0.665 | |
| Apolipoprotein A-1 | | rs34955778 | C | T | 0.419 | -0.012 | 0.002 | 29.4 | 3.70E-10 | 0.006 | 0.018 | 0.741 | |
| Apolipoprotein A-1 | | rs35135293 | T | C | 0.519 | -0.022 | 0.002 | 92.5 | 2.70E-28 | -0.015 | 0.018 | 0.407 | |
| Apolipoprotein A-1 | | rs351862 | T | C | 0.115 | 0.018 | 0.003 | 27.0 | 2.60E-09 | -0.053 | 0.023 | 0.019 | |
| Apolipoprotein A-1 | | rs35639749 | A | AC | 0.987 | -0.052 | 0.009 | 27.3 | 6.60E-09 | 0.013 | 0.110 | 0.905 | |
| Apolipoprotein A-1 | | rs35745599 | T | C | 0.239 | 0.028 | 0.002 | 115.5 | 4.00E-35 | -0.036 | 0.021 | 0.095 | |
| Apolipoprotein A-1 | | rs35909200 | G | T | 0.157 | 0.019 | 0.003 | 37.3 | 1.70E-12 | -0.029 | 0.024 | 0.222 | |
| Apolipoprotein A-1 | | rs36024006 | A | G | 0.452 | -0.018 | 0.002 | 61.7 | 1.60E-19 | -0.013 | 0.018 | 0.454 | |
| Apolipoprotein A-1 | | rs36096231 | T | C | 0.072 | -0.021 | 0.004 | 22.8 | 4.00E-08 | -0.006 | 0.049 | 0.899 | |
| Apolipoprotein A-1 | | rs367070 | G | A | 0.225 | 0.039 | 0.002 | 209.6 | 8.40E-62 | 0.005 | 0.019 | 0.792 | |
| Apolipoprotein A-1 | | rs367677 | G | A | 0.240 | 0.017 | 0.002 | 43.8 | 4.10E-14 | -0.019 | 0.020 | 0.328 | |
| Apolipoprotein A-1 | | rs3732356 | T | G | 0.934 | -0.037 | 0.004 | 65.6 | 2.70E-20 | 0.028 | 0.035 | 0.416 | |
| Apolipoprotein A-1 | | rs3740688 | T | G | 0.545 | 0.016 | 0.002 | 47.5 | 2.60E-15 | 0.025 | 0.020 | 0.204 | |
| Apolipoprotein A-1 | | rs3747973 | G | A | 0.593 | 0.015 | 0.002 | 41.9 | 9.90E-14 | -0.002 | 0.018 | 0.910 | |
| Apolipoprotein A-1 | | rs3749748 | T | C | 0.247 | 0.022 | 0.002 | 71.3 | 4.30E-22 | 0.013 | 0.024 | 0.596 | |
| Apolipoprotein A-1 | | rs3768321 | T | G | 0.197 | -0.042 | 0.002 | 222.3 | 6.20E-66 | 0.007 | 0.024 | 0.780 | |
| Apolipoprotein A-1 | | rs3798233 | C | A | 0.402 | 0.018 | 0.002 | 61.1 | 2.30E-19 | -0.019 | 0.018 | 0.288 | |
| Apolipoprotein A-1 | | rs3802548 | A | T | 0.240 | 0.034 | 0.002 | 166.7 | 9.40E-50 | -0.007 | 0.020 | 0.729 | |
| Apolipoprotein A-1 | | rs3818716 | T | C | 0.365 | -0.013 | 0.002 | 29.5 | 5.20E-10 | -0.017 | 0.019 | 0.363 | |
| Apolipoprotein A-1 | | rs3828960 | G | A | 0.207 | -0.015 | 0.002 | 27.5 | 1.70E-09 | -0.002 | 0.021 | 0.911 | |
| Apolipoprotein A-1 | | rs3915932 | C | G | 0.415 | 0.016 | 0.002 | 47.8 | 2.20E-15 | -0.034 | 0.018 | 0.061 | |
| Apolipoprotein A-1 | | rs4052908 | AATT | A | 0.636 | 0.015 | 0.002 | 43.5 | 4.60E-14 | -0.030 | 0.018 | 0.088 | |
| Apolipoprotein A-1 | | rs41272086 | A | G | 0.106 | -0.037 | 0.003 | 103.6 | 1.40E-31 | 0.033 | 0.029 | 0.260 | |
| Apolipoprotein A-1 | | rs4239651 | C | T | 0.794 | 0.026 | 0.002 | 89.7 | 1.40E-27 | -0.010 | 0.022 | 0.638 | |
| Apolipoprotein A-1 | | rs429358 | C | T | 0.154 | -0.103 | 0.003 | 1082.9 | 1.00E-200 | -0.008 | 0.023 | 0.719 | |
| Apolipoprotein A-1 | | rs4330777 | A | G | 0.475 | -0.017 | 0.002 | 53.7 | 2.80E-17 | 0.067 | 0.018 | 0.000 | |
| Apolipoprotein A-1 | | rs4441609 | C | T | 0.626 | 0.011 | 0.002 | 22.6 | 4.40E-08 | -0.039 | 0.018 | 0.028 | |
| Apolipoprotein A-1 | | rs4599108 | T | C | 0.488 | 0.014 | 0.002 | 38.8 | 1.40E-12 | 0.014 | 0.018 | 0.450 | |
| Apolipoprotein A-1 | | rs4632228 | T | G | 0.209 | -0.020 | 0.002 | 51.4 | 1.60E-16 | 0.009 | 0.021 | 0.679 | |
| Apolipoprotein A-1 | | rs4660586 | T | C | 0.739 | 0.013 | 0.002 | 24.0 | 1.80E-08 | -0.002 | 0.019 | 0.906 | |
| Apolipoprotein A-1 | | rs4676609 | T | C | 0.196 | 0.015 | 0.002 | 27.7 | 1.60E-09 | 0.038 | 0.021 | 0.067 | |
| Apolipoprotein A-1 | | rs472629 | A | G | 0.333 | -0.018 | 0.002 | 58.3 | 1.30E-18 | -0.002 | 0.022 | 0.917 | |
| Apolipoprotein A-1 | | rs4762756 | C | T | 0.736 | 0.014 | 0.002 | 30.0 | 4.60E-10 | 0.029 | 0.022 | 0.203 | |
| Apolipoprotein A-1 | | rs4784709 | A | T | 0.959 | -0.062 | 0.005 | 117.6 | 7.40E-36 | 0.049 | 0.065 | 0.445 | |
| Apolipoprotein A-1 | | rs4795386 | G | A | 0.724 | 0.026 | 0.002 | 102.9 | 5.10E-31 | 0.011 | 0.020 | 0.601 | |
| Apolipoprotein A-1 | | rs4807462 | T | A | 0.360 | -0.011 | 0.002 | 23.8 | 2.50E-08 | 0.011 | 0.018 | 0.534 | |
| Apolipoprotein A-1 | | rs4820346 | G | C | 0.693 | 0.013 | 0.002 | 28.4 | 1.10E-09 | -0.008 | 0.018 | 0.659 | |
| Apolipoprotein A-1 | | rs4875043 | C | A | 0.217 | -0.015 | 0.002 | 30.7 | 2.90E-10 | -0.007 | 0.021 | 0.741 | |
| Apolipoprotein A-1 | | rs4883201 | G | A | 0.102 | -0.025 | 0.003 | 46.4 | 5.30E-15 | -0.041 | 0.027 | 0.133 | |
| Apolipoprotein A-1 | | rs4930352 | T | G | 0.494 | 0.017 | 0.002 | 55.0 | 5.30E-17 | 0.016 | 0.018 | 0.368 | |
| Apolipoprotein A-1 | | rs49675 | A | G | 0.098 | 0.021 | 0.003 | 31.4 | 1.30E-10 | 0.031 | 0.036 | 0.387 | |
| Apolipoprotein A-1 | | rs532436 | A | G | 0.185 | 0.030 | 0.003 | 109.8 | 1.70E-33 | 0.100 | 0.022 | 0.000 | |
| Apolipoprotein A-1 | | rs55710224 | A | G | 0.499 | 0.012 | 0.002 | 29.0 | 6.10E-10 | -0.011 | 0.018 | 0.543 | |
| Apolipoprotein A-1 | | rs557933 | C | A | 0.520 | 0.019 | 0.002 | 71.2 | 3.20E-22 | 0.025 | 0.018 | 0.160 | |
| Apolipoprotein A-1 | | rs55801554 | A | C | 0.247 | 0.014 | 0.002 | 27.6 | 1.70E-09 | -0.005 | 0.021 | 0.827 | |
| Apolipoprotein A-1 | | rs55812947 | C | T | 0.129 | -0.016 | 0.003 | 22.9 | 4.00E-08 | -0.013 | 0.034 | 0.694 | |
| Apolipoprotein A-1 | | rs559355 | T | A | 0.157 | -0.045 | 0.003 | 207.7 | 8.80E-62 | 0.022 | 0.021 | 0.289 | |
| Apolipoprotein A-1 | | rs57274629 | G | A | 0.358 | 0.015 | 0.002 | 40.7 | 3.30E-13 | 0.021 | 0.018 | 0.225 | |
| Apolipoprotein A-1 | | rs58473820 | T | C | 0.378 | 0.021 | 0.002 | 79.6 | 1.20E-24 | -0.001 | 0.018 | 0.950 | |
| Apolipoprotein A-1 | | rs59104589 | T | C | 0.358 | 0.017 | 0.002 | 52.4 | 7.60E-17 | 0.024 | 0.018 | 0.189 | |
| Apolipoprotein A-1 | | rs59347135 | G | C | 0.046 | -0.081 | 0.005 | 225.2 | 1.40E-63 | -0.020 | 0.044 | 0.655 | |
| Apolipoprotein A-1 | | rs6057911 | G | C | 0.131 | 0.019 | 0.003 | 31.0 | 2.00E-10 | 0.010 | 0.023 | 0.669 | |
| Apolipoprotein A-1 | | rs6062510 | C | G | 0.674 | -0.015 | 0.002 | 40.1 | 3.50E-13 | -0.018 | 0.020 | 0.362 | |
| Apolipoprotein A-1 | | rs61352607 | T | G | 0.241 | 0.028 | 0.002 | 110.5 | 1.10E-33 | 0.040 | 0.021 | 0.052 | |
| Apolipoprotein A-1 | | rs613808 | G | A | 0.715 | -0.085 | 0.002 | 1159.4 | 1.00E-200 | 0.004 | 0.018 | 0.828 | |
| Apolipoprotein A-1 | | rs61596977 | T | C | 0.140 | -0.016 | 0.003 | 22.9 | 3.80E-08 | 0.001 | 0.024 | 0.975 | |
| Apolipoprotein A-1 | | rs61805076 | C | T | 0.333 | -0.015 | 0.002 | 41.2 | 1.50E-13 | 0.013 | 0.019 | 0.488 | |
| Apolipoprotein A-1 | | rs61980899 | C | T | 0.538 | -0.014 | 0.002 | 38.3 | 1.50E-12 | 0.006 | 0.018 | 0.733 | |
| Apolipoprotein A-1 | | rs62114506 | C | G | 0.262 | 0.014 | 0.002 | 30.0 | 2.70E-10 | -0.006 | 0.019 | 0.771 | |
| Apolipoprotein A-1 | | rs62135193 | T | C | 0.541 | -0.011 | 0.002 | 22.9 | 4.20E-08 | -0.023 | 0.018 | 0.195 | |
| Apolipoprotein A-1 | | rs6224 | T | G | 0.474 | 0.013 | 0.002 | 30.9 | 1.80E-10 | -0.021 | 0.018 | 0.252 | |
| Apolipoprotein A-1 | | rs62369502 | C | T | 0.076 | -0.021 | 0.004 | 25.5 | 6.80E-09 | -0.014 | 0.037 | 0.702 | |
| Apolipoprotein A-1 | | rs62405458 | T | C | 0.183 | -0.015 | 0.003 | 25.7 | 6.50E-09 | 0.020 | 0.021 | 0.343 | |
| Apolipoprotein A-1 | | rs6448429 | T | C | 0.166 | -0.022 | 0.003 | 53.7 | 6.40E-17 | 0.013 | 0.025 | 0.591 | |
| Apolipoprotein A-1 | | rs6469605 | T | C | 0.568 | 0.035 | 0.002 | 236.1 | 1.00E-69 | -0.031 | 0.019 | 0.102 | |
| Apolipoprotein A-1 | | rs6505176 | G | T | 0.332 | -0.017 | 0.002 | 53.0 | 8.60E-17 | -0.029 | 0.019 | 0.126 | |
| Apolipoprotein A-1 | | rs6557267 | T | C | 0.404 | -0.012 | 0.002 | 26.8 | 2.80E-09 | 0.016 | 0.018 | 0.379 | |
| Apolipoprotein A-1 | | rs6705285 | T | G | 0.609 | 0.011 | 0.002 | 23.3 | 1.70E-08 | -0.008 | 0.019 | 0.679 | |
| Apolipoprotein A-1 | | rs676210 | A | G | 0.205 | 0.060 | 0.002 | 463.5 | 8.71E-136 | -0.021 | 0.020 | 0.283 | |
| Apolipoprotein A-1 | | rs6765484 | T | C | 0.473 | 0.020 | 0.002 | 81.9 | 2.50E-25 | -0.037 | 0.018 | 0.037 | |
| Apolipoprotein A-1 | | rs6772763 | C | T | 0.565 | 0.013 | 0.002 | 32.1 | 8.50E-11 | -0.006 | 0.018 | 0.756 | |
| Apolipoprotein A-1 | | rs6807935 | G | A | 0.343 | -0.013 | 0.002 | 28.5 | 8.20E-10 | -0.008 | 0.018 | 0.664 | |
| Apolipoprotein A-1 | | rs681869 | T | C | 0.704 | -0.017 | 0.002 | 44.8 | 1.50E-14 | 0.031 | 0.020 | 0.133 | |
| Apolipoprotein A-1 | | rs686030 | A | C | 0.859 | 0.056 | 0.003 | 303.7 | 4.70E-89 | -0.012 | 0.027 | 0.653 | |
| Apolipoprotein A-1 | | rs6977416 | A | G | 0.334 | -0.016 | 0.002 | 43.5 | 5.70E-14 | -0.001 | 0.018 | 0.972 | |
| Apolipoprotein A-1 | | rs698927 | C | A | 0.184 | 0.025 | 0.003 | 71.1 | 2.80E-22 | 0.028 | 0.023 | 0.220 | |
| Apolipoprotein A-1 | | rs7032795 | C | T | 0.494 | 0.015 | 0.002 | 41.9 | 1.30E-13 | 0.016 | 0.018 | 0.363 | |
| Apolipoprotein A-1 | | rs7036107 | G | A | 0.511 | -0.011 | 0.002 | 24.9 | 1.80E-08 | 0.007 | 0.018 | 0.714 | |
| Apolipoprotein A-1 | | rs7136506 | C | T | 0.216 | -0.032 | 0.002 | 135.0 | 2.10E-39 | -0.012 | 0.021 | 0.562 | |
| Apolipoprotein A-1 | | rs7147511 | T | C | 0.495 | -0.015 | 0.002 | 42.2 | 1.50E-13 | -0.005 | 0.018 | 0.776 | |
| Apolipoprotein A-1 | | rs7170463 | G | A | 0.311 | 0.016 | 0.002 | 44.1 | 2.20E-14 | 0.008 | 0.018 | 0.665 | |
| Apolipoprotein A-1 | | rs7216643 | T | C | 0.224 | -0.022 | 0.002 | 68.3 | 2.90E-21 | -0.005 | 0.021 | 0.808 | |
| Apolipoprotein A-1 | | rs7238484 | T | G | 0.268 | -0.021 | 0.002 | 70.5 | 5.30E-22 | -0.036 | 0.022 | 0.102 | |
| Apolipoprotein A-1 | | rs7251640 | C | T | 0.194 | 0.015 | 0.002 | 28.3 | 1.30E-09 | -0.010 | 0.021 | 0.653 | |
| Apolipoprotein A-1 | | rs72729623 | T | C | 0.143 | -0.015 | 0.003 | 22.7 | 4.40E-08 | 0.011 | 0.023 | 0.633 | |
| Apolipoprotein A-1 | | rs72761606 | C | T | 0.131 | -0.016 | 0.003 | 23.3 | 2.80E-08 | 0.006 | 0.024 | 0.806 | |
| Apolipoprotein A-1 | | rs72926946 | A | C | 0.296 | -0.015 | 0.002 | 39.0 | 6.20E-13 | -0.006 | 0.020 | 0.749 | |
| Apolipoprotein A-1 | | rs72959041 | A | G | 0.049 | -0.036 | 0.005 | 47.9 | 4.20E-15 | 0.073 | 0.036 | 0.045 | |
| Apolipoprotein A-1 | | rs7304603 | C | T | 0.538 | -0.013 | 0.002 | 31.7 | 1.10E-10 | 0.023 | 0.018 | 0.214 | |
| Apolipoprotein A-1 | | rs73052033 | C | T | 0.185 | -0.018 | 0.003 | 40.3 | 3.20E-13 | 0.010 | 0.024 | 0.670 | |
| Apolipoprotein A-1 | | rs7305678 | G | T | 0.866 | -0.022 | 0.003 | 43.8 | 5.40E-14 | 0.014 | 0.026 | 0.595 | |
| Apolipoprotein A-1 | | rs73216701 | G | A | 0.430 | 0.016 | 0.002 | 52.3 | 9.40E-17 | -0.003 | 0.018 | 0.876 | |
| Apolipoprotein A-1 | | rs737338 | T | C | 0.035 | -0.108 | 0.005 | 311.4 | 2.30E-91 | 0.004 | 0.036 | 0.920 | |
| Apolipoprotein A-1 | | rs74025321 | G | A | 0.074 | -0.030 | 0.004 | 48.2 | 1.10E-15 | -0.006 | 0.035 | 0.864 | |
| Apolipoprotein A-1 | | rs74500135 | C | T | 0.010 | 0.074 | 0.010 | 41.5 | 1.10E-12 | -0.076 | 0.171 | 0.656 | |
| Apolipoprotein A-1 | | rs7503353 | T | G | 0.532 | -0.014 | 0.002 | 35.8 | 7.30E-12 | 0.003 | 0.018 | 0.850 | |
| Apolipoprotein A-1 | | rs75104038 | A | G | 0.061 | -0.039 | 0.004 | 69.1 | 1.30E-21 | 0.126 | 0.049 | 0.011 | |
| Apolipoprotein A-1 | | rs75152587 | T | G | 0.013 | -0.080 | 0.009 | 63.0 | 1.20E-19 | -0.080 | 0.082 | 0.327 | |
| Apolipoprotein A-1 | | rs75246752 | C | G | 0.013 | 0.050 | 0.009 | 24.7 | 1.00E-08 | 0.076 | 0.160 | 0.635 | |
| Apolipoprotein A-1 | | rs75265117 | G | C | 0.120 | 0.032 | 0.003 | 83.0 | 1.20E-25 | -0.013 | 0.029 | 0.670 | |
| Apolipoprotein A-1 | | rs75406471 | A | G | 0.154 | -0.025 | 0.003 | 62.3 | 1.30E-19 | -0.020 | 0.025 | 0.437 | |
| Apolipoprotein A-1 | | rs76213248 | T | C | 0.410 | 0.021 | 0.002 | 86.4 | 1.80E-26 | 0.015 | 0.018 | 0.417 | |
| Apolipoprotein A-1 | | rs76428106 | C | T | 0.013 | -0.061 | 0.009 | 37.7 | 1.30E-11 | 0.072 | 0.083 | 0.384 | |
| Apolipoprotein A-1 | | rs76456334 | C | T | 0.040 | -0.037 | 0.005 | 40.3 | 7.40E-13 | -0.096 | 0.049 | 0.051 | |
| Apolipoprotein A-1 | | rs76962725 | A | G | 0.037 | -0.030 | 0.005 | 25.0 | 1.30E-08 | 0.044 | 0.068 | 0.518 | |
| Apolipoprotein A-1 | | rs7700617 | A | C | 0.497 | -0.013 | 0.002 | 31.9 | 9.90E-11 | 0.020 | 0.018 | 0.283 | |
| Apolipoprotein A-1 | | rs77960347 | G | A | 0.013 | 0.336 | 0.009 | 1167.6 | 1.00E-200 | -0.017 | 0.101 | 0.870 | |
| Apolipoprotein A-1 | | rs78058190 | A | G | 0.050 | -0.066 | 0.005 | 165.3 | 2.60E-39 | 0.032 | 0.032 | 0.318 | |
| Apolipoprotein A-1 | | rs7817574 | C | T | 0.185 | 0.036 | 0.003 | 155.5 | 7.40E-47 | -0.012 | 0.020 | 0.574 | |
| Apolipoprotein A-1 | | rs78296522 | A | C | 0.045 | 0.068 | 0.005 | 158.1 | 9.00E-47 | 0.009 | 0.044 | 0.832 | |
| Apolipoprotein A-1 | | rs78807370 | A | G | 0.148 | 0.016 | 0.003 | 25.2 | 8.60E-09 | 0.035 | 0.026 | 0.174 | |
| Apolipoprotein A-1 | | rs78965095 | T | C | 0.020 | 0.044 | 0.007 | 29.8 | 3.50E-10 | -0.029 | 0.073 | 0.692 | |
| Apolipoprotein A-1 | | rs7896518 | G | A | 0.428 | 0.015 | 0.002 | 45.7 | 1.60E-14 | -0.005 | 0.018 | 0.794 | |
| Apolipoprotein A-1 | | rs79506257 | A | G | 0.048 | 0.028 | 0.005 | 28.6 | 1.20E-09 | 0.022 | 0.032 | 0.486 | |
| Apolipoprotein A-1 | | rs7952521 | A | G | 0.106 | 0.027 | 0.003 | 54.2 | 2.80E-16 | -0.008 | 0.028 | 0.777 | |
| Apolipoprotein A-1 | | rs79984435 | A | G | 0.092 | -0.086 | 0.003 | 482.7 | 4.20E-142 | 0.013 | 0.030 | 0.663 | |
| Apolipoprotein A-1 | | rs80236739 | G | A | 0.050 | -0.032 | 0.005 | 38.7 | 8.20E-13 | -0.007 | 0.054 | 0.895 | |
| Apolipoprotein A-1 | | rs8028785 | C | T | 0.183 | 0.015 | 0.003 | 25.1 | 7.70E-09 | -0.017 | 0.023 | 0.474 | |
| Apolipoprotein A-1 | | rs8086351 | G | C | 0.824 | 0.096 | 0.003 | 1057.7 | 1.00E-200 | 0.003 | 0.023 | 0.883 | |
| Apolipoprotein A-1 | | rs8098618 | T | C | 0.479 | 0.012 | 0.002 | 26.1 | 5.10E-09 | 0.004 | 0.018 | 0.832 | |
| Apolipoprotein A-1 | | rs8103728 | G | C | 0.670 | 0.023 | 0.002 | 90.9 | 7.30E-28 | 0.012 | 0.019 | 0.522 | |
| Apolipoprotein A-1 | | rs851132 | C | G | 0.592 | -0.025 | 0.002 | 115.9 | 4.10E-35 | 0.019 | 0.018 | 0.291 | |
| Apolipoprotein A-1 | | rs921919 | A | G | 0.669 | -0.037 | 0.002 | 240.3 | 2.50E-68 | 0.001 | 0.018 | 0.956 | |
| Apolipoprotein A-1 | | rs9347737 | G | A | 0.428 | -0.011 | 0.002 | 23.8 | 2.90E-08 | -0.030 | 0.018 | 0.091 | |
| Apolipoprotein A-1 | | rs9426827 | C | T | 0.478 | 0.017 | 0.002 | 57.2 | 2.80E-18 | 0.003 | 0.018 | 0.873 | |
| Apolipoprotein A-1 | | rs9471972 | A | G | 0.535 | 0.027 | 0.002 | 141.4 | 1.80E-42 | -0.013 | 0.018 | 0.474 | |
| Apolipoprotein A-1 | | rs9604045 | T | G | 0.254 | 0.015 | 0.002 | 35.4 | 5.80E-11 | -0.046 | 0.024 | 0.051 | |
| Apolipoprotein A-1 | | rs9608972 | C | T | 0.241 | -0.017 | 0.002 | 44.1 | 2.80E-14 | 0.008 | 0.022 | 0.727 | |
| Apolipoprotein A-1 | | rs9647335 | T | A | 0.192 | 0.032 | 0.002 | 122.1 | 8.60E-37 | -0.024 | 0.027 | 0.370 | |
| Apolipoprotein A-1 | | rs976002 | G | A | 0.245 | 0.023 | 0.002 | 77.6 | 4.40E-24 | -0.013 | 0.021 | 0.532 | |
| Apolipoprotein A-1 | | rs9817452 | T | G | 0.388 | 0.017 | 0.002 | 57.1 | 5.20E-18 | 0.029 | 0.019 | 0.128 | |
| Apolipoprotein A-1 | | rs9977268 | T | C | 0.199 | -0.018 | 0.002 | 41.6 | 1.50E-13 | 0.029 | 0.024 | 0.226 | |
| Apolipoprotein A-1 | | rs9987289 | G | A | 0.909 | 0.085 | 0.003 | 474.4 | 1.00E-138 | -0.005 | 0.026 | 0.860 | |
| Apolipoprotein A-1 | | rs9989419 | G | A | 0.606 | 0.115 | 0.002 | 2499.7 | 1.00E-200 | -0.036 | 0.018 | 0.049 | |
| Apolipoprotein B | | rs1003533 | T | C | 0.188 | -0.020 | 0.003 | 53.9 | 5.30E-14 | -0.004 | 0.021 | 0.866 | |
| Apolipoprotein B | | rs10087526 | G | T | 0.199 | 0.019 | 0.003 | 49.2 | 6.20E-13 | 0.027 | 0.021 | 0.205 | |
| Apolipoprotein B | | rs10151436 | T | A | 0.112 | -0.019 | 0.003 | 31.4 | 9.80E-09 | 0.051 | 0.029 | 0.078 | |
| Apolipoprotein B | | rs10201242 | A | G | 0.074 | -0.023 | 0.004 | 30.7 | 1.20E-08 | 0.034 | 0.038 | 0.374 | |
| Apolipoprotein B | | rs10448340 | G | T | 0.320 | -0.017 | 0.002 | 55.6 | 2.00E-14 | 0.005 | 0.019 | 0.780 | |
| Apolipoprotein B | | rs10794579 | C | T | 0.575 | 0.014 | 0.002 | 40.5 | 6.80E-11 | 0.006 | 0.018 | 0.731 | |
| Apolipoprotein B | | rs10832963 | G | T | 0.744 | 0.022 | 0.002 | 80.6 | 3.80E-20 | -0.008 | 0.018 | 0.666 | |
| Apolipoprotein B | | rs10953298 | T | C | 0.236 | -0.017 | 0.002 | 46.3 | 3.70E-12 | -0.045 | 0.021 | 0.030 | |
| Apolipoprotein B | | rs11047939 | A | G | 0.225 | 0.015 | 0.003 | 35.8 | 1.00E-09 | -0.005 | 0.019 | 0.796 | |
| Apolipoprotein B | | rs11057397 | T | C | 0.337 | -0.018 | 0.002 | 66.1 | 7.80E-17 | -0.005 | 0.019 | 0.777 | |
| Apolipoprotein B | | rs11057837 | T | C | 0.103 | 0.022 | 0.003 | 38.4 | 2.50E-10 | 0.003 | 0.027 | 0.927 | |
| Apolipoprotein B | | rs11065384 | C | T | 0.690 | -0.022 | 0.002 | 88.4 | 4.90E-22 | -0.039 | 0.018 | 0.033 | |
| Apolipoprotein B | | rs11099097 | T | C | 0.291 | -0.016 | 0.002 | 47.5 | 1.80E-12 | 0.000 | 0.019 | 0.987 | |
| Apolipoprotein B | | rs111928762 | G | A | 0.040 | 0.052 | 0.005 | 89.9 | 3.20E-22 | -0.018 | 0.037 | 0.635 | |
| Apolipoprotein B | | rs112220485 | C | T | 0.086 | 0.021 | 0.004 | 28.9 | 3.90E-08 | -0.054 | 0.037 | 0.140 | |
| Apolipoprotein B | | rs112758337 | A | G | 0.186 | -0.021 | 0.003 | 60.0 | 2.10E-15 | -0.045 | 0.024 | 0.063 | |
| Apolipoprotein B | | rs113177823 | A | G | 0.054 | -0.041 | 0.005 | 76.4 | 7.20E-19 | 0.012 | 0.037 | 0.746 | |
| Apolipoprotein B | | rs114165349 | C | G | 0.023 | 0.090 | 0.007 | 162.1 | 7.70E-39 | -0.071 | 0.047 | 0.128 | |
| Apolipoprotein B | | rs11568318 | A | C | 0.066 | 0.029 | 0.004 | 45.1 | 4.20E-12 | -0.063 | 0.051 | 0.217 | |
| Apolipoprotein B | | rs115692156 | G | A | 0.009 | -0.112 | 0.012 | 95.8 | 5.10E-21 | 0.060 | 0.056 | 0.282 | |
| Apolipoprotein B | | rs11591147 | T | G | 0.017 | -0.346 | 0.008 | 1810.2 | 1.00E-200 | 0.113 | 0.047 | 0.016 | |
| Apolipoprotein B | | rs11601507 | A | C | 0.069 | 0.041 | 0.004 | 97.4 | 1.10E-24 | -0.025 | 0.033 | 0.445 | |
| Apolipoprotein B | | rs116093870 | C | T | 0.068 | 0.026 | 0.004 | 38.1 | 2.30E-10 | 0.030 | 0.046 | 0.519 | |
| Apolipoprotein B | | rs11621792 | T | C | 0.453 | 0.021 | 0.002 | 96.7 | 1.30E-23 | -0.027 | 0.018 | 0.138 | |
| Apolipoprotein B | | rs116734477 | T | C | 0.041 | -0.063 | 0.005 | 138.0 | 2.50E-33 | -0.015 | 0.051 | 0.772 | |
| Apolipoprotein B | | rs11673631 | C | G | 0.043 | 0.039 | 0.005 | 55.5 | 3.20E-14 | -0.008 | 0.043 | 0.859 | |
| Apolipoprotein B | | rs11709868 | T | G | 0.297 | -0.016 | 0.002 | 44.4 | 9.50E-12 | -0.046 | 0.020 | 0.023 | |
| Apolipoprotein B | | rs117733303 | G | A | 0.018 | 0.086 | 0.008 | 118.7 | 3.60E-29 | -0.057 | 0.084 | 0.498 | |
| Apolipoprotein B | | rs118039278 | A | G | 0.079 | 0.087 | 0.004 | 480.5 | 2.20E-111 | -0.040 | 0.042 | 0.340 | |
| Apolipoprotein B | | rs11901691 | A | C | 0.577 | 0.014 | 0.002 | 43.6 | 1.50E-11 | -0.022 | 0.018 | 0.210 | |
| Apolipoprotein B | | rs12046278 | C | T | 0.345 | -0.016 | 0.002 | 48.5 | 6.40E-13 | 0.019 | 0.018 | 0.283 | |
| Apolipoprotein B | | rs12054451 | G | T | 0.259 | 0.016 | 0.002 | 42.9 | 2.10E-11 | 0.004 | 0.021 | 0.855 | |
| Apolipoprotein B | | rs12078100 | G | C | 0.623 | 0.015 | 0.002 | 47.8 | 1.20E-12 | 0.015 | 0.020 | 0.456 | |
| Apolipoprotein B | | rs12208357 | T | C | 0.070 | 0.063 | 0.004 | 227.2 | 1.00E-53 | 0.012 | 0.038 | 0.758 | |
| Apolipoprotein B | | rs1229984 | C | T | 0.973 | 0.039 | 0.006 | 34.5 | 1.10E-09 | -0.202 | 0.120 | 0.093 | |
| Apolipoprotein B | | rs12469941 | T | C | 0.401 | 0.013 | 0.002 | 38.0 | 1.50E-10 | -0.036 | 0.018 | 0.042 | |
| Apolipoprotein B | | rs12471768 | C | T | 0.704 | 0.014 | 0.002 | 35.4 | 8.80E-10 | -0.019 | 0.021 | 0.368 | |
| Apolipoprotein B | | rs1250258 | T | C | 0.737 | 0.014 | 0.002 | 34.1 | 1.90E-09 | -0.035 | 0.022 | 0.109 | |
| Apolipoprotein B | | rs12597418 | A | G | 0.368 | 0.018 | 0.002 | 67.8 | 2.90E-17 | -0.028 | 0.018 | 0.129 | |
| Apolipoprotein B | | rs1260326 | C | T | 0.605 | -0.050 | 0.002 | 516.3 | 1.90E-121 | -0.009 | 0.018 | 0.633 | |
| Apolipoprotein B | | rs12603290 | C | T | 0.515 | -0.029 | 0.002 | 182.6 | 9.80E-44 | 0.013 | 0.018 | 0.444 | |
| Apolipoprotein B | | rs12691088 | A | G | 0.021 | 0.246 | 0.008 | 1092.0 | 1.00E-200 | 0.071 | 0.050 | 0.154 | |
| Apolipoprotein B | | rs1277762 | T | C | 0.843 | 0.017 | 0.003 | 34.2 | 2.20E-09 | -0.001 | 0.022 | 0.950 | |
| Apolipoprotein B | | rs12916 | C | T | 0.400 | 0.055 | 0.002 | 637.3 | 5.20E-148 | 0.005 | 0.018 | 0.798 | |
| Apolipoprotein B | | rs13076933 | G | T | 0.259 | -0.018 | 0.002 | 56.3 | 1.80E-14 | -0.018 | 0.020 | 0.379 | |
| Apolipoprotein B | | rs13108218 | G | A | 0.615 | -0.023 | 0.002 | 109.5 | 1.60E-26 | -0.012 | 0.019 | 0.538 | |
| Apolipoprotein B | | rs13230111 | G | A | 0.492 | -0.014 | 0.002 | 42.0 | 2.80E-11 | 0.021 | 0.018 | 0.240 | |
| Apolipoprotein B | | rs13247874 | T | C | 0.197 | -0.022 | 0.003 | 64.8 | 1.40E-16 | -0.012 | 0.023 | 0.616 | |
| Apolipoprotein B | | rs13379043 | C | T | 0.280 | -0.014 | 0.002 | 36.6 | 1.10E-09 | -0.021 | 0.022 | 0.341 | |
| Apolipoprotein B | | rs13389219 | T | C | 0.393 | -0.016 | 0.002 | 54.5 | 2.60E-14 | -0.003 | 0.019 | 0.862 | |
| Apolipoprotein B | | rs1358980 | T | C | 0.483 | 0.015 | 0.002 | 48.7 | 1.10E-12 | 0.030 | 0.018 | 0.086 | |
| Apolipoprotein B | | rs13702 | C | T | 0.288 | -0.030 | 0.002 | 156.8 | 7.80E-38 | 0.001 | 0.020 | 0.963 | |
| Apolipoprotein B | | rs138026891 | T | C | 0.011 | -0.069 | 0.010 | 43.4 | 1.60E-11 | 0.079 | 0.070 | 0.257 | |
| Apolipoprotein B | | rs138354 | C | T | 0.535 | -0.012 | 0.002 | 31.8 | 6.90E-09 | -0.031 | 0.018 | 0.082 | |
| Apolipoprotein B | | rs138692741 | T | C | 0.036 | 0.071 | 0.006 | 155.0 | 3.00E-36 | 0.007 | 0.050 | 0.890 | |
| Apolipoprotein B | | rs141469619 | G | A | 0.010 | 0.079 | 0.011 | 53.1 | 1.60E-12 | 0.043 | 0.144 | 0.763 | |
| Apolipoprotein B | | rs143020224 | G | C | 0.119 | -0.165 | 0.003 | 2522.1 | 1.00E-200 | 0.028 | 0.029 | 0.331 | |
| Apolipoprotein B | | rs144926613 | T | C | 0.672 | -0.015 | 0.002 | 41.1 | 4.20E-11 | -0.009 | 0.019 | 0.654 | |
| Apolipoprotein B | | rs145730801 | C | T | 0.044 | 0.037 | 0.005 | 52.3 | 3.60E-13 | 0.083 | 0.133 | 0.535 | |
| Apolipoprotein B | | rs146534110 | T | G | 0.013 | 0.067 | 0.009 | 51.4 | 1.80E-13 | -0.054 | 0.119 | 0.652 | |
| Apolipoprotein B | | rs147539187 | G | C | 0.074 | -0.023 | 0.004 | 31.0 | 1.10E-08 | -0.030 | 0.058 | 0.611 | |
| Apolipoprotein B | | rs148150904 | TTAAAG | T | 0.190 | 0.016 | 0.003 | 34.2 | 1.60E-09 | 0.011 | 0.020 | 0.569 | |
| Apolipoprotein B | | rs148601586 | G | C | 0.013 | 0.184 | 0.009 | 388.8 | 5.90E-89 | -0.053 | 0.165 | 0.748 | |
| Apolipoprotein B | | rs148933445 | A | G | 0.022 | -0.573 | 0.008 | 6174.1 | 1.00E-200 | -0.023 | 0.062 | 0.707 | |
| Apolipoprotein B | | rs150474434 | A | G | 0.101 | -0.034 | 0.003 | 94.2 | 2.80E-23 | -0.035 | 0.033 | 0.281 | |
| Apolipoprotein B | | rs150503754 | C | T | 0.015 | -0.052 | 0.009 | 34.8 | 2.40E-09 | 0.023 | 0.050 | 0.647 | |
| Apolipoprotein B | | rs150820726 | T | A | 0.010 | 0.072 | 0.011 | 46.4 | 1.10E-11 | 0.039 | 0.043 | 0.366 | |
| Apolipoprotein B | | rs1556562 | T | G | 0.790 | 0.016 | 0.002 | 37.3 | 1.10E-10 | -0.016 | 0.021 | 0.442 | |
| Apolipoprotein B | | rs1561139 | T | G | 0.424 | -0.013 | 0.002 | 37.1 | 4.20E-10 | -0.024 | 0.019 | 0.210 | |
| Apolipoprotein B | | rs17036085 | G | A | 0.012 | -0.068 | 0.009 | 49.6 | 4.00E-13 | -0.067 | 0.045 | 0.133 | |
| Apolipoprotein B | | rs17050272 | A | G | 0.409 | -0.024 | 0.002 | 126.8 | 3.80E-31 | -0.002 | 0.018 | 0.894 | |
| Apolipoprotein B | | rs174564 | G | A | 0.349 | -0.045 | 0.002 | 405.3 | 1.00E-94 | 0.000 | 0.018 | 0.987 | |
| Apolipoprotein B | | rs17476364 | C | T | 0.108 | -0.021 | 0.003 | 37.2 | 4.00E-10 | 0.035 | 0.037 | 0.342 | |
| Apolipoprotein B | | rs17569873 | T | C | 0.201 | 0.018 | 0.003 | 47.5 | 1.60E-12 | -0.024 | 0.026 | 0.345 | |
| Apolipoprotein B | | rs1801689 | C | A | 0.031 | 0.065 | 0.006 | 108.7 | 9.90E-27 | 0.118 | 0.089 | 0.181 | |
| Apolipoprotein B | | rs183130 | T | C | 0.324 | -0.048 | 0.002 | 440.0 | 1.30E-102 | -0.031 | 0.020 | 0.112 | |
| Apolipoprotein B | | rs188608977 | T | G | 0.011 | 0.062 | 0.010 | 35.5 | 2.10E-09 | -0.071 | 0.081 | 0.383 | |
| Apolipoprotein B | | rs1888488 | T | C | 0.565 | 0.017 | 0.002 | 65.1 | 1.50E-16 | -0.012 | 0.019 | 0.533 | |
| Apolipoprotein B | | rs200046586 | C | CA | 0.981 | 0.412 | 0.008 | 2764.7 | 1.00E-200 | -0.019 | 0.070 | 0.793 | |
| Apolipoprotein B | | rs2043085 | C | T | 0.612 | -0.019 | 0.002 | 78.1 | 1.20E-19 | 0.002 | 0.018 | 0.893 | |
| Apolipoprotein B | | rs2068888 | A | G | 0.451 | -0.024 | 0.002 | 127.1 | 5.20E-31 | -0.007 | 0.018 | 0.697 | |
| Apolipoprotein B | | rs2073547 | G | A | 0.184 | 0.031 | 0.003 | 128.4 | 1.10E-31 | 0.028 | 0.019 | 0.135 | |
| Apolipoprotein B | | rs2137234 | C | T | 0.195 | 0.015 | 0.003 | 32.2 | 5.70E-09 | 0.008 | 0.025 | 0.755 | |
| Apolipoprotein B | | rs2160994 | C | T | 0.647 | 0.017 | 0.002 | 59.8 | 2.10E-15 | -0.053 | 0.019 | 0.005 | |
| Apolipoprotein B | | rs2199048 | G | A | 0.314 | -0.013 | 0.002 | 33.1 | 3.80E-09 | 0.004 | 0.021 | 0.858 | |
| Apolipoprotein B | | rs2238162 | T | C | 0.523 | -0.023 | 0.002 | 112.4 | 1.50E-27 | -0.012 | 0.018 | 0.484 | |
| Apolipoprotein B | | rs224391 | C | G | 0.227 | -0.017 | 0.002 | 47.1 | 2.00E-12 | -0.022 | 0.021 | 0.305 | |
| Apolipoprotein B | | rs2256814 | A | G | 0.198 | 0.015 | 0.003 | 29.4 | 2.90E-08 | 0.013 | 0.022 | 0.557 | |
| Apolipoprotein B | | rs2287622 | G | A | 0.603 | -0.020 | 0.002 | 87.1 | 7.70E-22 | -0.027 | 0.018 | 0.117 | |
| Apolipoprotein B | | rs2446066 | T | G | 0.175 | 0.016 | 0.003 | 30.8 | 1.20E-08 | 0.023 | 0.024 | 0.336 | |
| Apolipoprotein B | | rs2519093 | T | C | 0.185 | 0.041 | 0.003 | 223.3 | 3.90E-53 | 0.102 | 0.022 | 0.000 | |
| Apolipoprotein B | | rs2618566 | T | G | 0.660 | -0.030 | 0.002 | 181.1 | 2.00E-43 | -0.008 | 0.019 | 0.664 | |
| Apolipoprotein B | | rs2737263 | T | G | 0.280 | -0.019 | 0.002 | 60.8 | 1.20E-15 | 0.029 | 0.019 | 0.129 | |
| Apolipoprotein B | | rs2738447 | C | A | 0.593 | 0.044 | 0.002 | 404.0 | 9.90E-97 | -0.013 | 0.018 | 0.481 | |
| Apolipoprotein B | | rs2761311 | T | C | 0.572 | 0.012 | 0.002 | 31.4 | 1.00E-08 | 0.011 | 0.018 | 0.524 | |
| Apolipoprotein B | | rs278981 | C | T | 0.758 | 0.015 | 0.002 | 35.4 | 6.10E-10 | -0.012 | 0.021 | 0.575 | |
| Apolipoprotein B | | rs2807854 | C | T | 0.671 | 0.016 | 0.002 | 51.5 | 1.40E-13 | -0.001 | 0.018 | 0.980 | |
| Apolipoprotein B | | rs28406917 | T | C | 0.428 | 0.013 | 0.002 | 38.6 | 2.00E-10 | -0.008 | 0.018 | 0.639 | |
| Apolipoprotein B | | rs28601761 | G | C | 0.419 | -0.073 | 0.002 | 1128.6 | 1.00E-200 | -0.018 | 0.018 | 0.324 | |
| Apolipoprotein B | | rs28814720 | G | A | 0.520 | 0.012 | 0.002 | 30.2 | 4.30E-08 | 0.015 | 0.018 | 0.406 | |
| Apolipoprotein B | | rs3127580 | T | C | 0.155 | 0.040 | 0.003 | 188.4 | 3.00E-45 | -0.005 | 0.026 | 0.838 | |
| Apolipoprotein B | | rs34042070 | G | C | 0.188 | 0.049 | 0.003 | 327.6 | 1.90E-76 | 0.023 | 0.022 | 0.301 | |
| Apolipoprotein B | | rs35081008 | T | C | 0.148 | -0.031 | 0.003 | 107.3 | 7.80E-27 | 0.014 | 0.026 | 0.587 | |
| Apolipoprotein B | | rs35583283 | C | G | 0.155 | 0.016 | 0.003 | 29.6 | 2.30E-08 | -0.001 | 0.022 | 0.974 | |
| Apolipoprotein B | | rs35764292 | G | A | 0.040 | 0.051 | 0.005 | 88.4 | 1.30E-21 | 0.062 | 0.055 | 0.261 | |
| Apolipoprotein B | | rs35882350 | G | A | 0.261 | 0.014 | 0.002 | 31.1 | 1.00E-08 | 0.027 | 0.020 | 0.169 | |
| Apolipoprotein B | | rs35980001 | G | GC | 0.787 | -0.024 | 0.003 | 87.9 | 1.40E-21 | -0.018 | 0.021 | 0.375 | |
| Apolipoprotein B | | rs3746337 | T | C | 0.489 | 0.016 | 0.002 | 53.7 | 5.10E-14 | 0.004 | 0.018 | 0.813 | |
| Apolipoprotein B | | rs3780181 | G | A | 0.068 | -0.026 | 0.004 | 37.4 | 4.70E-10 | -0.037 | 0.038 | 0.342 | |
| Apolipoprotein B | | rs3822855 | T | G | 0.402 | 0.015 | 0.002 | 46.5 | 2.40E-12 | -0.019 | 0.018 | 0.288 | |
| Apolipoprotein B | | rs3823376 | T | C | 0.502 | 0.016 | 0.002 | 59.2 | 2.50E-15 | 0.006 | 0.018 | 0.758 | |
| Apolipoprotein B | | rs3860846 | T | C | 0.275 | 0.014 | 0.002 | 33.6 | 4.10E-09 | 0.011 | 0.021 | 0.580 | |
| Apolipoprotein B | | rs4052908 | AATT | A | 0.636 | -0.013 | 0.002 | 32.2 | 7.00E-09 | -0.030 | 0.018 | 0.088 | |
| Apolipoprotein B | | rs4307732 | A | G | 0.106 | 0.048 | 0.003 | 193.2 | 5.80E-46 | 0.013 | 0.024 | 0.577 | |
| Apolipoprotein B | | rs4470903 | G | C | 0.215 | 0.035 | 0.003 | 176.9 | 2.60E-42 | 0.005 | 0.022 | 0.835 | |
| Apolipoprotein B | | rs454715 | G | T | 0.583 | -0.024 | 0.002 | 118.3 | 2.40E-29 | -0.051 | 0.020 | 0.009 | |
| Apolipoprotein B | | rs45537841 | T | C | 0.181 | -0.016 | 0.003 | 32.9 | 4.10E-09 | 0.031 | 0.022 | 0.166 | |
| Apolipoprotein B | | rs4671050 | T | G | 0.316 | -0.020 | 0.002 | 73.7 | 1.00E-18 | 0.023 | 0.018 | 0.212 | |
| Apolipoprotein B | | rs4687614 | A | G | 0.945 | 0.027 | 0.005 | 33.0 | 3.70E-09 | 0.025 | 0.047 | 0.585 | |
| Apolipoprotein B | | rs4689088 | A | G | 0.619 | 0.012 | 0.002 | 32.2 | 5.90E-09 | 0.003 | 0.018 | 0.871 | |
| Apolipoprotein B | | rs472495 | T | G | 0.649 | 0.041 | 0.002 | 332.7 | 1.30E-78 | -0.019 | 0.018 | 0.275 | |
| Apolipoprotein B | | rs478975 | A | G | 0.643 | 0.014 | 0.002 | 40.3 | 7.90E-11 | 0.001 | 0.018 | 0.960 | |
| Apolipoprotein B | | rs4803818 | T | C | 0.023 | 0.042 | 0.007 | 34.4 | 1.30E-09 | 0.060 | 0.044 | 0.174 | |
| Apolipoprotein B | | rs4935356 | A | T | 0.244 | 0.025 | 0.003 | 97.9 | 6.60E-22 | -0.044 | 0.042 | 0.290 | |
| Apolipoprotein B | | rs546240 | T | C | 0.620 | -0.012 | 0.002 | 29.2 | 3.10E-08 | -0.007 | 0.018 | 0.685 | |
| Apolipoprotein B | | rs556107 | T | C | 0.523 | 0.036 | 0.002 | 281.8 | 1.30E-66 | 0.028 | 0.018 | 0.117 | |
| Apolipoprotein B | | rs55637835 | T | C | 0.121 | -0.019 | 0.003 | 33.0 | 6.10E-09 | 0.066 | 0.030 | 0.025 | |
| Apolipoprotein B | | rs55714927 | T | C | 0.190 | -0.032 | 0.003 | 140.3 | 5.10E-34 | -0.045 | 0.020 | 0.025 | |
| Apolipoprotein B | | rs55831924 | T | C | 0.361 | 0.018 | 0.002 | 67.6 | 4.80E-17 | 0.037 | 0.019 | 0.053 | |
| Apolipoprotein B | | rs581080 | C | G | 0.819 | 0.019 | 0.003 | 44.9 | 6.40E-12 | -0.017 | 0.025 | 0.509 | |
| Apolipoprotein B | | rs58148580 | T | C | 0.110 | 0.021 | 0.003 | 39.7 | 9.60E-11 | -0.061 | 0.028 | 0.029 | |
| Apolipoprotein B | | rs59328596 | A | G | 0.148 | -0.021 | 0.003 | 48.9 | 6.60E-13 | 0.052 | 0.023 | 0.023 | |
| Apolipoprotein B | | rs597808 | G | A | 0.516 | 0.022 | 0.002 | 103.2 | 2.40E-25 | 0.031 | 0.018 | 0.077 | |
| Apolipoprotein B | | rs6072279 | A | G | 0.474 | 0.029 | 0.002 | 187.2 | 8.70E-45 | -0.022 | 0.018 | 0.218 | |
| Apolipoprotein B | | rs6073958 | C | T | 0.199 | 0.042 | 0.003 | 250.6 | 2.50E-59 | -0.039 | 0.023 | 0.092 | |
| Apolipoprotein B | | rs6129620 | A | T | 0.338 | -0.025 | 0.002 | 126.0 | 8.70E-30 | -0.008 | 0.019 | 0.669 | |
| Apolipoprotein B | | rs61754230 | T | C | 0.020 | 0.043 | 0.007 | 31.8 | 6.80E-09 | -0.131 | 0.112 | 0.244 | |
| Apolipoprotein B | | rs62119267 | C | A | 0.022 | -0.335 | 0.007 | 2156.8 | 1.00E-200 | 0.024 | 0.092 | 0.790 | |
| Apolipoprotein B | | rs62122481 | A | C | 0.377 | 0.084 | 0.002 | 1464.4 | 1.00E-200 | -0.005 | 0.019 | 0.804 | |
| Apolipoprotein B | | rs6426328 | T | G | 0.490 | 0.012 | 0.002 | 32.7 | 4.00E-09 | 0.006 | 0.018 | 0.753 | |
| Apolipoprotein B | | rs6475606 | T | C | 0.484 | -0.017 | 0.002 | 65.9 | 7.20E-17 | -0.080 | 0.018 | 0.000 | |
| Apolipoprotein B | | rs6560499 | A | G | 0.576 | -0.012 | 0.002 | 31.7 | 9.00E-09 | 0.030 | 0.018 | 0.105 | |
| Apolipoprotein B | | rs6602909 | C | T | 0.328 | 0.021 | 0.002 | 89.0 | 4.60E-22 | 0.021 | 0.018 | 0.266 | |
| Apolipoprotein B | | rs6657811 | T | A | 0.130 | -0.128 | 0.003 | 1626.0 | 1.00E-200 | 0.001 | 0.029 | 0.974 | |
| Apolipoprotein B | | rs6667939 | T | C | 0.719 | 0.014 | 0.002 | 35.3 | 1.20E-09 | -0.001 | 0.019 | 0.946 | |
| Apolipoprotein B | | rs6689611 | A | G | 0.012 | -0.132 | 0.010 | 188.3 | 3.90E-44 | -0.074 | 0.174 | 0.670 | |
| Apolipoprotein B | | rs67038483 | T | C | 0.045 | 0.028 | 0.005 | 29.2 | 3.00E-08 | 0.038 | 0.032 | 0.240 | |
| Apolipoprotein B | | rs6874202 | C | T | 0.634 | 0.031 | 0.002 | 198.6 | 1.60E-47 | 0.039 | 0.019 | 0.039 | |
| Apolipoprotein B | | rs6940814 | G | A | 0.587 | -0.020 | 0.002 | 84.7 | 3.00E-21 | 0.011 | 0.018 | 0.536 | |
| Apolipoprotein B | | rs7012637 | A | G | 0.474 | 0.020 | 0.002 | 87.7 | 1.30E-21 | 0.013 | 0.020 | 0.524 | |
| Apolipoprotein B | | rs7108486 | C | T | 0.024 | -0.043 | 0.007 | 37.4 | 5.20E-10 | -0.007 | 0.060 | 0.903 | |
| Apolipoprotein B | | rs71311871 | G | A | 0.083 | -0.026 | 0.004 | 46.4 | 2.90E-12 | 0.062 | 0.032 | 0.057 | |
| Apolipoprotein B | | rs7249565 | A | G | 0.415 | 0.015 | 0.002 | 45.2 | 2.90E-12 | -0.006 | 0.018 | 0.742 | |
| Apolipoprotein B | | rs72631343 | G | C | 0.129 | -0.028 | 0.003 | 76.7 | 2.30E-19 | 0.025 | 0.025 | 0.327 | |
| Apolipoprotein B | | rs72663045 | G | T | 0.021 | 0.042 | 0.007 | 32.0 | 8.00E-09 | -0.063 | 0.067 | 0.346 | |
| Apolipoprotein B | | rs72823013 | A | G | 0.126 | -0.023 | 0.003 | 50.6 | 3.20E-13 | -0.056 | 0.030 | 0.063 | |
| Apolipoprotein B | | rs72848251 | A | G | 0.195 | 0.035 | 0.003 | 168.0 | 1.50E-38 | -0.011 | 0.028 | 0.695 | |
| Apolipoprotein B | | rs73075609 | T | C | 0.027 | 0.049 | 0.006 | 54.1 | 6.20E-14 | 0.063 | 0.092 | 0.498 | |
| Apolipoprotein B | | rs74454529 | A | G | 0.060 | 0.028 | 0.004 | 39.5 | 1.30E-10 | -0.006 | 0.032 | 0.858 | |
| Apolipoprotein B | | rs75331444 | A | G | 0.066 | -0.090 | 0.004 | 440.1 | 2.00E-103 | 0.050 | 0.032 | 0.114 | |
| Apolipoprotein B | | rs7569317 | C | T | 0.531 | 0.018 | 0.002 | 71.5 | 2.60E-18 | 0.009 | 0.018 | 0.618 | |
| Apolipoprotein B | | rs7590687 | C | T | 0.923 | -0.043 | 0.004 | 115.6 | 3.40E-27 | 0.015 | 0.035 | 0.669 | |
| Apolipoprotein B | | rs7603427 | T | C | 0.533 | 0.014 | 0.002 | 40.3 | 6.60E-11 | 0.005 | 0.018 | 0.796 | |
| Apolipoprotein B | | rs76186504 | T | C | 0.025 | -0.116 | 0.007 | 290.7 | 3.30E-69 | 0.054 | 0.067 | 0.418 | |
| Apolipoprotein B | | rs7707394 | A | G | 0.357 | 0.035 | 0.002 | 250.8 | 1.40E-59 | -0.015 | 0.018 | 0.409 | |
| Apolipoprotein B | | rs77083979 | A | G | 0.133 | -0.019 | 0.003 | 34.7 | 2.00E-09 | -0.025 | 0.031 | 0.406 | |
| Apolipoprotein B | | rs7734476 | A | G | 0.550 | 0.020 | 0.002 | 86.5 | 1.20E-21 | 0.027 | 0.018 | 0.122 | |
| Apolipoprotein B | | rs7746081 | A | G | 0.304 | -0.023 | 0.002 | 96.4 | 6.70E-24 | 0.037 | 0.018 | 0.044 | |
| Apolipoprotein B | | rs77498041 | A | G | 0.191 | -0.016 | 0.003 | 35.9 | 8.50E-10 | 0.023 | 0.023 | 0.307 | |
| Apolipoprotein B | | rs77542162 | G | A | 0.022 | 0.116 | 0.007 | 259.8 | 2.50E-61 | -0.128 | 0.105 | 0.223 | |
| Apolipoprotein B | | rs79149284 | T | C | 0.038 | 0.034 | 0.005 | 36.5 | 3.20E-10 | 0.031 | 0.050 | 0.539 | |
| Apolipoprotein B | | rs79220007 | C | T | 0.076 | -0.054 | 0.004 | 178.8 | 4.20E-43 | -0.080 | 0.046 | 0.086 | |
| Apolipoprotein B | | rs8016418 | A | T | 0.375 | 0.020 | 0.002 | 80.1 | 4.60E-20 | 0.007 | 0.018 | 0.680 | |
| Apolipoprotein B | | rs80276949 | A | G | 0.023 | 0.042 | 0.007 | 34.2 | 2.10E-09 | 0.181 | 0.080 | 0.024 | |
| Apolipoprotein B | | rs8107974 | T | A | 0.076 | -0.091 | 0.004 | 508.3 | 4.50E-121 | -0.034 | 0.036 | 0.346 | |
| Apolipoprotein B | | rs9297994 | A | G | 0.665 | -0.029 | 0.002 | 166.0 | 6.20E-40 | 0.001 | 0.018 | 0.938 | |
| Apolipoprotein B | | rs9482772 | C | T | 0.448 | 0.018 | 0.002 | 68.2 | 2.20E-17 | 0.095 | 0.018 | 0.000 | |
| Apolipoprotein B | | rs9496567 | A | G | 0.244 | -0.019 | 0.002 | 58.7 | 3.80E-15 | 0.010 | 0.022 | 0.637 | |
| Apolipoprotein B | | rs9616822 | A | G | 0.351 | 0.016 | 0.002 | 50.8 | 2.70E-13 | 0.001 | 0.018 | 0.951 | |
| Apolipoprotein B | | rs964184 | C | G | 0.867 | -0.077 | 0.003 | 594.9 | 2.50E-138 | -0.048 | 0.025 | 0.055 | |
| Apolipoprotein B | | rs969075 | C | T | 0.665 | 0.014 | 0.002 | 37.3 | 4.40E-10 | -0.030 | 0.020 | 0.132 | |
| Apolipoprotein B | | rs9834932 | G | A | 0.089 | -0.032 | 0.004 | 71.5 | 3.90E-18 | -0.004 | 0.032 | 0.890 | |
| Apolipoprotein B | | rs9884390 | C | T | 0.234 | 0.023 | 0.002 | 85.1 | 7.80E-21 | -0.015 | 0.021 | 0.480 | |
| Apolipoprotein B | | rs9894946 | G | A | 0.841 | -0.017 | 0.003 | 34.5 | 3.10E-09 | -0.025 | 0.024 | 0.299 | |
| HDL cholesterol | | rs10031010 | A | G | 0.185 | 0.014 | 0.002 | 23.2 | 2.10E-08 | -0.022 | 0.020 | 0.292 | |
| HDL cholesterol | | rs10053349 | C | T | 0.390 | 0.012 | 0.002 | 27.7 | 7.90E-10 | -0.022 | 0.018 | 0.209 | |
| HDL cholesterol | | rs10108282 | A | T | 0.206 | 0.017 | 0.002 | 37.5 | 6.30E-13 | 0.015 | 0.023 | 0.515 | |
| HDL cholesterol | | rs10162642 | A | G | 0.212 | -0.048 | 0.002 | 309.8 | 4.60E-93 | 0.017 | 0.025 | 0.492 | |
| HDL cholesterol | | rs10233430 | C | T | 0.427 | -0.020 | 0.002 | 83.0 | 2.10E-26 | 0.024 | 0.018 | 0.173 | |
| HDL cholesterol | | rs1045241 | T | C | 0.271 | 0.016 | 0.002 | 43.0 | 2.60E-14 | 0.039 | 0.019 | 0.038 | |
| HDL cholesterol | | rs1047891 | A | C | 0.316 | -0.019 | 0.002 | 62.4 | 2.20E-20 | -0.010 | 0.019 | 0.607 | |
| HDL cholesterol | | rs10504477 | C | T | 0.412 | -0.015 | 0.002 | 44.4 | 6.10E-15 | 0.005 | 0.018 | 0.782 | |
| HDL cholesterol | | rs10513801 | G | T | 0.137 | -0.030 | 0.003 | 88.0 | 6.70E-28 | -0.019 | 0.029 | 0.506 | |
| HDL cholesterol | | rs1055582 | T | C | 0.505 | 0.014 | 0.002 | 39.7 | 2.10E-13 | -0.021 | 0.018 | 0.240 | |
| HDL cholesterol | | rs10750766 | A | C | 0.710 | -0.019 | 0.002 | 57.4 | 9.20E-19 | 0.006 | 0.020 | 0.771 | |
| HDL cholesterol | | rs10774439 | A | G | 0.815 | 0.021 | 0.002 | 51.3 | 1.40E-16 | -0.021 | 0.021 | 0.305 | |
| HDL cholesterol | | rs10786114 | T | C | 0.875 | 0.024 | 0.003 | 50.4 | 1.20E-16 | -0.006 | 0.025 | 0.819 | |
| HDL cholesterol | | rs1083470 | A | G | 0.616 | 0.012 | 0.002 | 25.6 | 3.40E-09 | 0.001 | 0.018 | 0.945 | |
| HDL cholesterol | | rs11009262 | T | G | 0.057 | -0.023 | 0.004 | 23.8 | 1.20E-08 | 0.008 | 0.036 | 0.819 | |
| HDL cholesterol | | rs11021232 | C | T | 0.181 | -0.017 | 0.002 | 33.5 | 1.70E-11 | -0.017 | 0.023 | 0.457 | |
| HDL cholesterol | | rs11045171 | G | A | 0.198 | 0.028 | 0.002 | 103.1 | 4.30E-32 | -0.004 | 0.021 | 0.850 | |
| HDL cholesterol | | rs111363680 | T | C | 0.031 | 0.033 | 0.006 | 26.4 | 3.10E-08 | 0.179 | 0.075 | 0.016 | |
| HDL cholesterol | | rs11171710 | A | G | 0.448 | -0.011 | 0.002 | 26.3 | 2.80E-09 | 0.005 | 0.018 | 0.763 | |
| HDL cholesterol | | rs112001035 | A | G | 0.060 | -0.047 | 0.004 | 99.9 | 3.30E-30 | 0.028 | 0.032 | 0.391 | |
| HDL cholesterol | | rs11218738 | A | G | 0.249 | 0.023 | 0.002 | 83.2 | 1.40E-26 | -0.005 | 0.021 | 0.812 | |
| HDL cholesterol | | rs112233856 | G | A | 0.029 | -0.053 | 0.006 | 62.5 | 8.00E-20 | 0.000 | 0.039 | 0.994 | |
| HDL cholesterol | | rs112350227 | T | C | 0.028 | -0.032 | 0.006 | 22.1 | 4.10E-08 | -0.143 | 0.089 | 0.106 | |
| HDL cholesterol | | rs11239536 | A | T | 0.241 | 0.029 | 0.002 | 122.2 | 5.50E-38 | -0.006 | 0.020 | 0.769 | |
| HDL cholesterol | | rs11254464 | C | T | 0.423 | 0.013 | 0.002 | 32.1 | 3.90E-11 | 0.002 | 0.018 | 0.908 | |
| HDL cholesterol | | rs1132274 | A | C | 0.154 | -0.022 | 0.003 | 49.9 | 1.60E-16 | 0.025 | 0.026 | 0.326 | |
| HDL cholesterol | | rs113740515 | A | G | 0.209 | 0.038 | 0.002 | 191.0 | 1.20E-58 | -0.046 | 0.023 | 0.042 | |
| HDL cholesterol | | rs113966472 | A | G | 0.032 | 0.032 | 0.006 | 26.7 | 4.80E-09 | 0.003 | 0.046 | 0.949 | |
| HDL cholesterol | | rs114165349 | C | G | 0.023 | -0.081 | 0.006 | 119.9 | 3.90E-37 | -0.071 | 0.047 | 0.128 | |
| HDL cholesterol | | rs11429307 | G | GT | 0.809 | 0.031 | 0.002 | 118.5 | 9.70E-37 | -0.064 | 0.026 | 0.012 | |
| HDL cholesterol | | rs115912456 | G | A | 0.041 | 0.028 | 0.005 | 25.2 | 4.50E-09 | -0.030 | 0.066 | 0.654 | |
| HDL cholesterol | | rs116006942 | A | G | 0.061 | -0.030 | 0.004 | 41.2 | 1.30E-13 | 0.041 | 0.045 | 0.356 | |
| HDL cholesterol | | rs11614202 | G | A | 0.842 | 0.023 | 0.003 | 55.8 | 3.00E-18 | 0.020 | 0.025 | 0.408 | |
| HDL cholesterol | | rs11631178 | C | T | 0.104 | 0.021 | 0.003 | 33.6 | 1.30E-11 | -0.029 | 0.029 | 0.321 | |
| HDL cholesterol | | rs11640494 | A | G | 0.457 | -0.015 | 0.002 | 47.2 | 6.50E-16 | -0.018 | 0.019 | 0.348 | |
| HDL cholesterol | | rs11664369 | T | C | 0.267 | -0.023 | 0.002 | 83.5 | 1.80E-26 | -0.036 | 0.022 | 0.103 | |
| HDL cholesterol | | rs1168114 | G | A | 0.652 | 0.016 | 0.002 | 44.2 | 7.50E-15 | -0.002 | 0.020 | 0.904 | |
| HDL cholesterol | | rs116843064 | A | G | 0.019 | 0.206 | 0.007 | 652.0 | 3.40E-195 | -0.056 | 0.055 | 0.303 | |
| HDL cholesterol | | rs116857878 | T | C | 0.020 | 0.048 | 0.007 | 37.6 | 4.50E-12 | 0.171 | 0.095 | 0.072 | |
| HDL cholesterol | | rs11688682 | C | G | 0.271 | 0.015 | 0.002 | 34.7 | 2.80E-11 | 0.018 | 0.020 | 0.374 | |
| HDL cholesterol | | rs11704977 | T | C | 0.150 | 0.015 | 0.003 | 22.3 | 4.30E-08 | -0.026 | 0.022 | 0.232 | |
| HDL cholesterol | | rs117230571 | G | A | 0.077 | -0.027 | 0.004 | 40.7 | 2.20E-13 | 0.030 | 0.048 | 0.531 | |
| HDL cholesterol | | rs117291242 | T | C | 0.037 | -0.032 | 0.005 | 28.4 | 4.50E-10 | -0.006 | 0.039 | 0.889 | |
| HDL cholesterol | | rs117762989 | T | C | 0.043 | -0.027 | 0.005 | 24.1 | 9.30E-09 | 0.050 | 0.086 | 0.560 | |
| HDL cholesterol | | rs117847213 | G | A | 0.041 | 0.030 | 0.005 | 27.6 | 1.20E-09 | -0.116 | 0.066 | 0.081 | |
| HDL cholesterol | | rs12046972 | C | T | 0.564 | -0.015 | 0.002 | 43.6 | 1.00E-14 | 0.021 | 0.018 | 0.237 | |
| HDL cholesterol | | rs12205778 | A | G | 0.259 | 0.016 | 0.002 | 40.8 | 8.60E-14 | -0.023 | 0.021 | 0.277 | |
| HDL cholesterol | | rs12229011 | T | C | 0.097 | -0.026 | 0.003 | 48.0 | 7.80E-16 | -0.004 | 0.034 | 0.909 | |
| HDL cholesterol | | rs1225053 | C | T | 0.264 | -0.015 | 0.002 | 35.5 | 3.90E-12 | -0.009 | 0.020 | 0.652 | |
| HDL cholesterol | | rs1240820 | A | G | 0.293 | 0.013 | 0.002 | 29.1 | 3.50E-10 | 0.018 | 0.019 | 0.356 | |
| HDL cholesterol | | rs12411732 | A | G | 0.146 | -0.031 | 0.003 | 94.0 | 6.80E-29 | -0.014 | 0.026 | 0.585 | |
| HDL cholesterol | | rs12462109 | T | C | 0.287 | -0.016 | 0.002 | 42.1 | 3.80E-14 | 0.007 | 0.019 | 0.719 | |
| HDL cholesterol | | rs12475332 | G | T | 0.261 | 0.013 | 0.002 | 25.9 | 2.50E-09 | 0.014 | 0.021 | 0.527 | |
| HDL cholesterol | | rs12575456 | A | G | 0.322 | 0.045 | 0.002 | 350.8 | 1.40E-106 | 0.014 | 0.019 | 0.454 | |
| HDL cholesterol | | rs12686780 | T | C | 0.175 | -0.016 | 0.003 | 30.5 | 1.20E-10 | 0.000 | 0.022 | 0.987 | |
| HDL cholesterol | | rs1270076 | G | A | 0.777 | 0.014 | 0.002 | 28.8 | 3.50E-10 | 0.004 | 0.022 | 0.873 | |
| HDL cholesterol | | rs12705595 | A | G | 0.373 | 0.011 | 0.002 | 23.1 | 2.50E-08 | 0.002 | 0.018 | 0.909 | |
| HDL cholesterol | | rs12740374 | T | G | 0.221 | 0.029 | 0.002 | 115.9 | 2.20E-36 | -0.015 | 0.021 | 0.490 | |
| HDL cholesterol | | rs12740811 | G | A | 0.094 | -0.018 | 0.003 | 23.4 | 1.60E-08 | -0.025 | 0.032 | 0.425 | |
| HDL cholesterol | | rs12781812 | T | G | 0.401 | 0.011 | 0.002 | 22.3 | 3.40E-08 | -0.003 | 0.019 | 0.889 | |
| HDL cholesterol | | rs12921195 | A | C | 0.140 | -0.017 | 0.003 | 27.0 | 3.20E-09 | -0.001 | 0.027 | 0.973 | |
| HDL cholesterol | | rs12926854 | G | A | 0.270 | 0.012 | 0.002 | 23.9 | 9.50E-09 | -0.002 | 0.021 | 0.925 | |
| HDL cholesterol | | rs12928099 | A | C | 0.296 | 0.021 | 0.002 | 77.3 | 3.70E-25 | 0.004 | 0.020 | 0.853 | |
| HDL cholesterol | | rs12986742 | C | T | 0.476 | -0.011 | 0.002 | 22.6 | 3.00E-08 | -0.002 | 0.018 | 0.911 | |
| HDL cholesterol | | rs12998038 | T | C | 0.259 | 0.013 | 0.002 | 27.0 | 1.60E-09 | -0.019 | 0.019 | 0.322 | |
| HDL cholesterol | | rs13066793 | G | A | 0.090 | 0.022 | 0.003 | 32.3 | 3.30E-11 | -0.052 | 0.032 | 0.104 | |
| HDL cholesterol | | rs13087167 | C | G | 0.633 | 0.017 | 0.002 | 52.6 | 2.90E-17 | 0.001 | 0.018 | 0.947 | |
| HDL cholesterol | | rs13097947 | C | T | 0.649 | 0.016 | 0.002 | 46.9 | 3.80E-15 | -0.019 | 0.020 | 0.341 | |
| HDL cholesterol | | rs13107325 | T | C | 0.075 | -0.080 | 0.004 | 361.9 | 7.00E-109 | 0.080 | 0.075 | 0.290 | |
| HDL cholesterol | | rs13111599 | G | A | 0.737 | 0.013 | 0.002 | 25.6 | 3.50E-09 | 0.036 | 0.020 | 0.081 | |
| HDL cholesterol | | rs13137144 | A | G | 0.461 | 0.016 | 0.002 | 54.1 | 1.50E-17 | 0.040 | 0.018 | 0.026 | |
| HDL cholesterol | | rs13144151 | G | A | 0.850 | 0.018 | 0.003 | 32.9 | 3.80E-11 | -0.007 | 0.025 | 0.782 | |
| HDL cholesterol | | rs13235365 | T | C | 0.274 | 0.026 | 0.002 | 107.1 | 2.10E-33 | -0.032 | 0.021 | 0.119 | |
| HDL cholesterol | | rs13269725 | G | A | 0.078 | -0.026 | 0.004 | 39.4 | 1.90E-13 | -0.019 | 0.047 | 0.690 | |
| HDL cholesterol | | rs13379043 | C | T | 0.280 | 0.020 | 0.002 | 64.4 | 4.70E-20 | -0.021 | 0.022 | 0.341 | |
| HDL cholesterol | | rs13389219 | T | C | 0.393 | 0.028 | 0.002 | 148.2 | 4.50E-46 | -0.003 | 0.019 | 0.862 | |
| HDL cholesterol | | rs13402475 | G | C | 0.815 | -0.025 | 0.002 | 75.6 | 1.90E-23 | -0.016 | 0.019 | 0.406 | |
| HDL cholesterol | | rs1349852 | C | A | 0.475 | 0.011 | 0.002 | 25.3 | 5.80E-09 | 0.007 | 0.018 | 0.674 | |
| HDL cholesterol | | rs1383732 | G | A | 0.150 | -0.015 | 0.003 | 23.8 | 1.40E-08 | -0.050 | 0.030 | 0.100 | |
| HDL cholesterol | | rs1395221 | T | G | 0.398 | -0.011 | 0.002 | 24.2 | 1.00E-08 | 0.027 | 0.018 | 0.129 | |
| HDL cholesterol | | rs140064750 | C | T | 0.025 | -0.044 | 0.006 | 37.6 | 3.70E-12 | 0.064 | 0.055 | 0.248 | |
| HDL cholesterol | | rs140164052 | A | G | 0.032 | -0.042 | 0.005 | 43.3 | 1.20E-14 | -0.053 | 0.050 | 0.285 | |
| HDL cholesterol | | rs141062196 | A | G | 0.194 | -0.019 | 0.002 | 45.0 | 5.20E-15 | -0.026 | 0.026 | 0.307 | |
| HDL cholesterol | | rs1411432 | C | A | 0.186 | -0.014 | 0.002 | 23.9 | 1.30E-08 | 0.067 | 0.023 | 0.004 | |
| HDL cholesterol | | rs1412234 | C | T | 0.327 | -0.012 | 0.002 | 25.6 | 3.90E-09 | -0.038 | 0.018 | 0.034 | |
| HDL cholesterol | | rs141440048 | T | C | 0.018 | 0.044 | 0.008 | 26.8 | 7.80E-09 | 0.141 | 0.193 | 0.466 | |
| HDL cholesterol | | rs141469619 | G | A | 0.010 | -0.203 | 0.010 | 327.4 | 8.70E-89 | 0.043 | 0.144 | 0.763 | |
| HDL cholesterol | | rs142288236 | T | C | 0.015 | -0.078 | 0.008 | 70.6 | 1.60E-22 | 0.086 | 0.053 | 0.104 | |
| HDL cholesterol | | rs1431659 | G | A | 0.728 | 0.013 | 0.002 | 26.2 | 2.30E-09 | 0.002 | 0.021 | 0.939 | |
| HDL cholesterol | | rs144033177 | C | A | 0.016 | -0.056 | 0.008 | 38.9 | 1.40E-12 | -0.101 | 0.071 | 0.150 | |
| HDL cholesterol | | rs144311893 | T | C | 0.022 | 0.081 | 0.007 | 114.4 | 1.10E-33 | 0.091 | 0.103 | 0.378 | |
| HDL cholesterol | | rs1446585 | G | A | 0.244 | 0.017 | 0.002 | 42.0 | 1.10E-14 | -0.027 | 0.018 | 0.139 | |
| HDL cholesterol | | rs145947882 | C | A | 0.026 | -0.165 | 0.006 | 558.2 | 4.70E-161 | 0.027 | 0.051 | 0.597 | |
| HDL cholesterol | | rs1471251 | T | A | 0.398 | -0.019 | 0.002 | 72.6 | 3.60E-23 | 0.017 | 0.018 | 0.358 | |
| HDL cholesterol | | rs147627829 | A | G | 0.044 | -0.054 | 0.005 | 96.9 | 2.50E-30 | 0.161 | 0.059 | 0.006 | |
| HDL cholesterol | | rs147772065 | C | G | 0.035 | 0.029 | 0.005 | 22.5 | 4.10E-08 | -0.021 | 0.057 | 0.713 | |
| HDL cholesterol | | rs150224153 | T | C | 0.030 | -0.093 | 0.006 | 203.3 | 3.10E-59 | -0.050 | 0.033 | 0.131 | |
| HDL cholesterol | | rs150237291 | C | T | 0.022 | 0.045 | 0.007 | 35.4 | 5.10E-12 | 0.003 | 0.052 | 0.951 | |
| HDL cholesterol | | rs150844304 | C | A | 0.026 | -0.091 | 0.006 | 167.2 | 1.40E-51 | 0.117 | 0.124 | 0.344 | |
| HDL cholesterol | | rs150861794 | T | C | 0.018 | -0.045 | 0.008 | 29.0 | 4.00E-09 | -0.064 | 0.102 | 0.529 | |
| HDL cholesterol | | rs1534696 | A | C | 0.541 | 0.017 | 0.002 | 55.7 | 2.90E-18 | 0.010 | 0.018 | 0.571 | |
| HDL cholesterol | | rs1601934 | A | G | 0.687 | -0.101 | 0.002 | 1779.1 | 1.00E-200 | 0.000 | 0.018 | 0.998 | |
| HDL cholesterol | | rs16928809 | A | G | 0.093 | -0.026 | 0.003 | 47.1 | 1.70E-15 | -0.004 | 0.034 | 0.902 | |
| HDL cholesterol | | rs17124112 | A | C | 0.080 | -0.021 | 0.004 | 25.7 | 3.80E-09 | -0.029 | 0.037 | 0.421 | |
| HDL cholesterol | | rs17138358 | C | G | 0.398 | -0.027 | 0.002 | 143.6 | 2.20E-44 | 0.014 | 0.019 | 0.453 | |
| HDL cholesterol | | rs17309930 | A | C | 0.205 | -0.022 | 0.002 | 63.1 | 1.50E-20 | -0.030 | 0.021 | 0.144 | |
| HDL cholesterol | | rs17326656 | T | G | 0.239 | -0.022 | 0.002 | 73.6 | 1.50E-23 | -0.036 | 0.024 | 0.127 | |
| HDL cholesterol | | rs174566 | G | A | 0.350 | -0.056 | 0.002 | 581.9 | 1.20E-174 | 0.003 | 0.018 | 0.877 | |
| HDL cholesterol | | rs1760940 | C | A | 0.247 | 0.012 | 0.002 | 22.3 | 3.90E-08 | -0.002 | 0.021 | 0.909 | |
| HDL cholesterol | | rs17713879 | A | G | 0.365 | 0.014 | 0.002 | 36.1 | 2.00E-12 | -0.002 | 0.018 | 0.898 | |
| HDL cholesterol | | rs1771582 | G | T | 0.557 | 0.013 | 0.002 | 33.4 | 6.00E-11 | 0.016 | 0.019 | 0.389 | |
| HDL cholesterol | | rs183906992 | C | T | 0.043 | 0.029 | 0.005 | 28.7 | 5.50E-10 | -0.003 | 0.045 | 0.950 | |
| HDL cholesterol | | rs1862205 | A | G | 0.405 | 0.011 | 0.002 | 24.9 | 5.80E-09 | 0.020 | 0.018 | 0.257 | |
| HDL cholesterol | | rs188502504 | C | T | 0.036 | -0.032 | 0.005 | 28.4 | 5.60E-10 | 0.032 | 0.035 | 0.359 | |
| HDL cholesterol | | rs1955512 | A | G | 0.576 | 0.011 | 0.002 | 23.9 | 2.40E-08 | 0.015 | 0.018 | 0.409 | |
| HDL cholesterol | | rs1970811 | C | T | 0.458 | -0.012 | 0.002 | 27.0 | 1.40E-09 | 0.021 | 0.018 | 0.244 | |
| HDL cholesterol | | rs201441 | G | T | 0.581 | -0.011 | 0.002 | 23.7 | 1.40E-08 | -0.028 | 0.018 | 0.118 | |
| HDL cholesterol | | rs2066714 | C | T | 0.129 | 0.047 | 0.003 | 196.2 | 3.40E-60 | -0.010 | 0.030 | 0.746 | |
| HDL cholesterol | | rs2068888 | A | G | 0.450 | 0.019 | 0.002 | 73.6 | 1.30E-23 | -0.007 | 0.018 | 0.697 | |
| HDL cholesterol | | rs2098368 | T | C | 0.546 | -0.012 | 0.002 | 27.0 | 1.80E-09 | 0.030 | 0.018 | 0.102 | |
| HDL cholesterol | | rs2098918 | T | C | 0.455 | 0.012 | 0.002 | 28.2 | 5.80E-10 | -0.043 | 0.018 | 0.018 | |
| HDL cholesterol | | rs2111216 | G | A | 0.594 | 0.021 | 0.002 | 88.7 | 5.00E-28 | 0.006 | 0.018 | 0.744 | |
| HDL cholesterol | | rs2155220 | T | C | 0.438 | -0.011 | 0.002 | 22.0 | 3.80E-08 | -0.014 | 0.018 | 0.428 | |
| HDL cholesterol | | rs2159607 | T | G | 0.811 | -0.024 | 0.002 | 71.1 | 7.20E-23 | 0.028 | 0.030 | 0.351 | |
| HDL cholesterol | | rs2196808 | C | T | 0.732 | 0.013 | 0.002 | 26.2 | 2.50E-09 | 0.026 | 0.021 | 0.206 | |
| HDL cholesterol | | rs2236464 | C | T | 0.212 | -0.016 | 0.002 | 32.8 | 3.70E-11 | 0.006 | 0.025 | 0.825 | |
| HDL cholesterol | | rs2237035 | T | G | 0.386 | 0.014 | 0.002 | 37.1 | 1.20E-12 | -0.032 | 0.019 | 0.081 | |
| HDL cholesterol | | rs2247355 | T | C | 0.183 | 0.021 | 0.002 | 51.3 | 5.10E-17 | -0.008 | 0.020 | 0.684 | |
| HDL cholesterol | | rs2256609 | G | A | 0.189 | -0.033 | 0.002 | 134.1 | 2.20E-41 | -0.002 | 0.019 | 0.920 | |
| HDL cholesterol | | rs2268840 | C | T | 0.228 | 0.017 | 0.002 | 42.7 | 2.20E-14 | -0.024 | 0.021 | 0.254 | |
| HDL cholesterol | | rs2271308 | C | T | 0.733 | 0.028 | 0.002 | 122.9 | 3.00E-38 | 0.009 | 0.020 | 0.662 | |
| HDL cholesterol | | rs2290866 | T | C | 0.253 | -0.012 | 0.002 | 21.7 | 4.60E-08 | 0.011 | 0.019 | 0.572 | |
| HDL cholesterol | | rs2297409 | A | G | 0.195 | -0.033 | 0.002 | 141.0 | 1.00E-43 | 0.007 | 0.027 | 0.789 | |
| HDL cholesterol | | rs2298214 | A | C | 0.577 | -0.012 | 0.002 | 30.4 | 1.60E-10 | 0.006 | 0.018 | 0.718 | |
| HDL cholesterol | | rs2298624 | T | C | 0.133 | 0.030 | 0.003 | 83.8 | 1.10E-26 | -0.017 | 0.023 | 0.458 | |
| HDL cholesterol | | rs2298632 | T | C | 0.498 | 0.014 | 0.002 | 42.0 | 9.70E-14 | 0.005 | 0.018 | 0.786 | |
| HDL cholesterol | | rs2302263 | T | C | 0.089 | -0.036 | 0.003 | 86.7 | 1.60E-27 | -0.056 | 0.026 | 0.034 | |
| HDL cholesterol | | rs2307111 | C | T | 0.395 | 0.019 | 0.002 | 69.7 | 2.10E-22 | 0.003 | 0.018 | 0.855 | |
| HDL cholesterol | | rs2339234 | A | G | 0.683 | -0.012 | 0.002 | 24.8 | 7.30E-09 | -0.012 | 0.019 | 0.532 | |
| HDL cholesterol | | rs235314 | T | C | 0.532 | -0.018 | 0.002 | 63.3 | 2.40E-20 | 0.004 | 0.018 | 0.804 | |
| HDL cholesterol | | rs2362541 | G | T | 0.508 | -0.011 | 0.002 | 23.8 | 1.10E-08 | 0.026 | 0.018 | 0.146 | |
| HDL cholesterol | | rs2364723 | C | G | 0.319 | 0.012 | 0.002 | 25.7 | 3.40E-09 | 0.005 | 0.019 | 0.809 | |
| HDL cholesterol | | rs2417125 | G | A | 0.284 | -0.013 | 0.002 | 28.4 | 5.20E-10 | 0.027 | 0.021 | 0.201 | |
| HDL cholesterol | | rs2435307 | T | C | 0.486 | 0.016 | 0.002 | 53.5 | 1.50E-17 | -0.013 | 0.018 | 0.450 | |
| HDL cholesterol | | rs2498786 | G | C | 0.616 | -0.025 | 0.002 | 124.1 | 2.60E-38 | 0.012 | 0.018 | 0.509 | |
| HDL cholesterol | | rs2516331 | A | C | 0.376 | 0.013 | 0.002 | 30.5 | 1.10E-10 | -0.030 | 0.020 | 0.131 | |
| HDL cholesterol | | rs2520096 | G | A | 0.270 | 0.015 | 0.002 | 34.5 | 7.70E-12 | 0.032 | 0.018 | 0.078 | |
| HDL cholesterol | | rs254562 | G | A | 0.409 | -0.011 | 0.002 | 25.7 | 3.40E-09 | 0.020 | 0.018 | 0.266 | |
| HDL cholesterol | | rs2586116 | G | C | 0.259 | -0.016 | 0.002 | 41.0 | 7.10E-14 | -0.041 | 0.021 | 0.046 | |
| HDL cholesterol | | rs2642438 | G | A | 0.703 | 0.028 | 0.002 | 129.1 | 2.10E-40 | -0.008 | 0.019 | 0.697 | |
| HDL cholesterol | | rs2645979 | A | G | 0.357 | 0.011 | 0.002 | 24.2 | 8.70E-09 | -0.010 | 0.018 | 0.574 | |
| HDL cholesterol | | rs267738 | G | T | 0.219 | 0.021 | 0.002 | 63.7 | 9.90E-21 | 0.019 | 0.022 | 0.390 | |
| HDL cholesterol | | rs2723065 | G | A | 0.377 | 0.015 | 0.002 | 42.5 | 2.40E-14 | -0.002 | 0.019 | 0.912 | |
| HDL cholesterol | | rs2726111 | G | A | 0.667 | -0.015 | 0.002 | 39.3 | 2.60E-13 | -0.001 | 0.018 | 0.950 | |
| HDL cholesterol | | rs2740488 | C | A | 0.265 | -0.069 | 0.002 | 743.6 | 1.00E-200 | 0.014 | 0.022 | 0.528 | |
| HDL cholesterol | | rs2750411 | G | T | 0.492 | -0.011 | 0.002 | 23.7 | 1.40E-08 | 0.010 | 0.018 | 0.555 | |
| HDL cholesterol | | rs2792751 | C | T | 0.725 | -0.036 | 0.002 | 210.0 | 3.20E-64 | -0.017 | 0.019 | 0.373 | |
| HDL cholesterol | | rs2800710 | C | T | 0.518 | -0.020 | 0.002 | 82.9 | 1.80E-26 | 0.092 | 0.018 | 0.000 | |
| HDL cholesterol | | rs2804894 | A | G | 0.735 | 0.017 | 0.002 | 47.2 | 2.30E-15 | 0.010 | 0.021 | 0.631 | |
| HDL cholesterol | | rs2814982 | T | C | 0.102 | -0.028 | 0.003 | 56.5 | 1.60E-18 | 0.047 | 0.024 | 0.054 | |
| HDL cholesterol | | rs28362901 | A | C | 0.088 | -0.024 | 0.003 | 37.1 | 1.20E-12 | -0.030 | 0.028 | 0.275 | |
| HDL cholesterol | | rs28510484 | C | G | 0.170 | -0.015 | 0.003 | 27.3 | 1.20E-09 | -0.001 | 0.023 | 0.980 | |
| HDL cholesterol | | rs286965 | C | T | 0.632 | -0.013 | 0.002 | 33.8 | 1.10E-11 | 0.007 | 0.018 | 0.694 | |
| HDL cholesterol | | rs2910949 | G | T | 0.355 | 0.013 | 0.002 | 33.2 | 2.00E-11 | -0.016 | 0.020 | 0.422 | |
| HDL cholesterol | | rs2925979 | C | T | 0.700 | 0.037 | 0.002 | 236.2 | 1.60E-73 | -0.013 | 0.019 | 0.490 | |
| HDL cholesterol | | rs2943645 | T | C | 0.647 | -0.043 | 0.002 | 349.1 | 5.90E-106 | 0.034 | 0.018 | 0.060 | |
| HDL cholesterol | | rs2963468 | G | A | 0.235 | -0.020 | 0.002 | 56.2 | 3.60E-18 | 0.003 | 0.026 | 0.900 | |
| HDL cholesterol | | rs2965169 | C | A | 0.389 | 0.012 | 0.002 | 28.0 | 6.30E-10 | 0.015 | 0.019 | 0.414 | |
| HDL cholesterol | | rs3027167 | T | C | 0.682 | -0.012 | 0.002 | 27.1 | 1.40E-09 | 0.003 | 0.021 | 0.884 | |
| HDL cholesterol | | rs308 | G | T | 0.021 | 0.127 | 0.007 | 261.5 | 1.10E-79 | 0.080 | 0.051 | 0.118 | |
| HDL cholesterol | | rs3184504 | C | T | 0.517 | 0.027 | 0.002 | 142.1 | 3.90E-44 | 0.034 | 0.018 | 0.057 | |
| HDL cholesterol | | rs32578 | A | G | 0.309 | 0.013 | 0.002 | 30.6 | 1.10E-10 | -0.012 | 0.018 | 0.505 | |
| HDL cholesterol | | rs330089 | C | T | 0.098 | 0.020 | 0.003 | 29.9 | 1.40E-10 | 0.026 | 0.036 | 0.467 | |
| HDL cholesterol | | rs34045894 | A | G | 0.156 | -0.017 | 0.003 | 31.8 | 4.60E-11 | -0.014 | 0.023 | 0.546 | |
| HDL cholesterol | | rs34138141 | T | G | 0.281 | -0.018 | 0.002 | 50.1 | 1.60E-16 | -0.024 | 0.021 | 0.262 | |
| HDL cholesterol | | rs343 | A | C | 0.083 | 0.134 | 0.003 | 1104.3 | 1.00E-200 | 0.011 | 0.036 | 0.768 | |
| HDL cholesterol | | rs34940374 | A | G | 0.183 | -0.017 | 0.002 | 35.1 | 5.30E-12 | 0.035 | 0.020 | 0.086 | |
| HDL cholesterol | | rs35493868 | G | C | 0.202 | 0.037 | 0.002 | 180.6 | 3.00E-55 | -0.007 | 0.023 | 0.766 | |
| HDL cholesterol | | rs35980001 | G | GC | 0.787 | -0.119 | 0.002 | 1923.2 | 1.00E-200 | -0.018 | 0.021 | 0.375 | |
| HDL cholesterol | | rs36057735 | G | C | 0.199 | -0.030 | 0.002 | 117.6 | 1.00E-36 | 0.024 | 0.025 | 0.326 | |
| HDL cholesterol | | rs367070 | G | A | 0.226 | 0.042 | 0.002 | 246.7 | 1.30E-74 | 0.005 | 0.019 | 0.792 | |
| HDL cholesterol | | rs367677 | G | A | 0.240 | 0.017 | 0.002 | 41.0 | 1.20E-13 | -0.019 | 0.020 | 0.328 | |
| HDL cholesterol | | rs3732356 | T | G | 0.934 | -0.030 | 0.004 | 44.7 | 1.00E-14 | 0.028 | 0.035 | 0.416 | |
| HDL cholesterol | | rs3745683 | A | G | 0.075 | -0.055 | 0.004 | 166.6 | 3.20E-51 | -0.013 | 0.031 | 0.661 | |
| HDL cholesterol | | rs3746915 | G | A | 0.581 | 0.011 | 0.002 | 23.3 | 2.10E-08 | -0.001 | 0.018 | 0.974 | |
| HDL cholesterol | | rs3747973 | G | A | 0.593 | 0.014 | 0.002 | 39.1 | 2.70E-13 | -0.002 | 0.018 | 0.910 | |
| HDL cholesterol | | rs3768321 | T | G | 0.197 | -0.045 | 0.002 | 261.3 | 1.50E-79 | 0.007 | 0.024 | 0.780 | |
| HDL cholesterol | | rs3794752 | C | T | 0.281 | 0.013 | 0.002 | 26.9 | 2.00E-09 | -0.019 | 0.020 | 0.321 | |
| HDL cholesterol | | rs3814883 | T | C | 0.482 | -0.015 | 0.002 | 47.2 | 5.90E-16 | 0.053 | 0.018 | 0.003 | |
| HDL cholesterol | | rs3924313 | A | G | 0.322 | -0.024 | 0.002 | 100.9 | 1.60E-31 | -0.011 | 0.020 | 0.600 | |
| HDL cholesterol | | rs4074448 | A | G | 0.589 | 0.015 | 0.002 | 44.8 | 1.30E-14 | 0.000 | 0.018 | 0.997 | |
| HDL cholesterol | | rs41272086 | A | G | 0.106 | -0.057 | 0.003 | 246.0 | 8.50E-75 | 0.033 | 0.029 | 0.260 | |
| HDL cholesterol | | rs42125 | G | A | 0.023 | -0.040 | 0.007 | 29.1 | 1.30E-08 | 0.034 | 0.059 | 0.568 | |
| HDL cholesterol | | rs429358 | C | T | 0.154 | -0.076 | 0.003 | 604.4 | 8.71E-181 | -0.008 | 0.023 | 0.719 | |
| HDL cholesterol | | rs4330777 | A | G | 0.475 | -0.020 | 0.002 | 78.7 | 1.30E-25 | 0.067 | 0.018 | 0.000 | |
| HDL cholesterol | | rs454968 | C | T | 0.645 | 0.011 | 0.002 | 22.5 | 3.20E-08 | -0.050 | 0.018 | 0.006 | |
| HDL cholesterol | | rs4599108 | T | C | 0.488 | 0.014 | 0.002 | 37.7 | 1.20E-12 | 0.014 | 0.018 | 0.450 | |
| HDL cholesterol | | rs460428 | C | T | 0.231 | -0.014 | 0.002 | 29.5 | 2.50E-10 | 0.001 | 0.022 | 0.950 | |
| HDL cholesterol | | rs4614 | G | A | 0.406 | -0.018 | 0.002 | 61.9 | 3.80E-20 | -0.014 | 0.019 | 0.458 | |
| HDL cholesterol | | rs4650994 | A | G | 0.532 | -0.018 | 0.002 | 65.6 | 2.10E-21 | 0.001 | 0.018 | 0.943 | |
| HDL cholesterol | | rs4691379 | T | C | 0.319 | 0.012 | 0.002 | 24.7 | 6.20E-09 | -0.012 | 0.020 | 0.572 | |
| HDL cholesterol | | rs4784709 | A | T | 0.959 | -0.075 | 0.005 | 176.8 | 3.00E-55 | 0.049 | 0.065 | 0.445 | |
| HDL cholesterol | | rs4803773 | G | A | 0.499 | 0.040 | 0.002 | 324.8 | 8.70E-90 | 0.002 | 0.018 | 0.922 | |
| HDL cholesterol | | rs4804101 | T | G | 0.439 | -0.014 | 0.002 | 38.5 | 5.30E-13 | 0.033 | 0.018 | 0.065 | |
| HDL cholesterol | | rs4855582 | T | C | 0.430 | 0.011 | 0.002 | 24.7 | 6.50E-09 | -0.003 | 0.018 | 0.874 | |
| HDL cholesterol | | rs4871603 | T | C | 0.653 | 0.036 | 0.002 | 237.3 | 5.20E-73 | -0.028 | 0.019 | 0.137 | |
| HDL cholesterol | | rs4871624 | G | T | 0.287 | -0.020 | 0.002 | 68.9 | 3.90E-22 | 0.013 | 0.020 | 0.516 | |
| HDL cholesterol | | rs4875043 | C | A | 0.217 | -0.015 | 0.002 | 30.8 | 1.30E-10 | -0.007 | 0.021 | 0.741 | |
| HDL cholesterol | | rs4917675 | C | T | 0.255 | 0.015 | 0.002 | 32.5 | 3.40E-11 | 0.009 | 0.020 | 0.633 | |
| HDL cholesterol | | rs4930352 | T | G | 0.494 | 0.016 | 0.002 | 52.8 | 7.30E-17 | 0.016 | 0.018 | 0.368 | |
| HDL cholesterol | | rs4969141 | T | C | 0.490 | 0.030 | 0.002 | 177.4 | 3.20E-54 | 0.028 | 0.018 | 0.128 | |
| HDL cholesterol | | rs532436 | A | G | 0.185 | 0.023 | 0.002 | 65.2 | 3.80E-21 | 0.100 | 0.022 | 0.000 | |
| HDL cholesterol | | rs549058 | T | G | 0.120 | 0.017 | 0.003 | 25.0 | 5.70E-09 | -0.051 | 0.029 | 0.084 | |
| HDL cholesterol | | rs55781197 | G | A | 0.115 | 0.059 | 0.003 | 283.9 | 4.00E-88 | 0.027 | 0.024 | 0.252 | |
| HDL cholesterol | | rs557933 | C | A | 0.520 | 0.015 | 0.002 | 46.9 | 1.30E-15 | 0.025 | 0.018 | 0.160 | |
| HDL cholesterol | | rs55935382 | A | C | 0.325 | 0.018 | 0.002 | 55.2 | 4.90E-18 | -0.008 | 0.019 | 0.687 | |
| HDL cholesterol | | rs559355 | T | A | 0.157 | -0.035 | 0.003 | 130.7 | 9.00E-41 | 0.022 | 0.021 | 0.289 | |
| HDL cholesterol | | rs564832 | C | T | 0.315 | -0.012 | 0.002 | 23.9 | 1.20E-08 | 0.051 | 0.019 | 0.006 | |
| HDL cholesterol | | rs58123204 | G | A | 0.153 | -0.018 | 0.003 | 35.8 | 2.90E-12 | 0.050 | 0.026 | 0.056 | |
| HDL cholesterol | | rs58298943 | T | C | 0.084 | 0.020 | 0.003 | 25.4 | 3.80E-09 | -0.098 | 0.029 | 0.001 | |
| HDL cholesterol | | rs59104589 | T | C | 0.358 | 0.015 | 0.002 | 41.9 | 3.50E-14 | 0.024 | 0.018 | 0.189 | |
| HDL cholesterol | | rs59781045 | T | C | 0.068 | 0.074 | 0.004 | 281.3 | 4.50E-85 | -0.021 | 0.032 | 0.519 | |
| HDL cholesterol | | rs6018652 | A | G | 0.793 | 0.026 | 0.002 | 89.2 | 4.60E-28 | 0.010 | 0.022 | 0.631 | |
| HDL cholesterol | | rs6059958 | T | C | 0.171 | 0.015 | 0.003 | 24.6 | 9.70E-09 | 0.003 | 0.022 | 0.885 | |
| HDL cholesterol | | rs6066148 | C | G | 0.260 | 0.013 | 0.002 | 27.5 | 1.00E-09 | -0.006 | 0.022 | 0.803 | |
| HDL cholesterol | | rs6073958 | C | T | 0.199 | -0.061 | 0.002 | 478.3 | 4.10E-143 | -0.039 | 0.023 | 0.092 | |
| HDL cholesterol | | rs6075860 | A | G | 0.565 | -0.013 | 0.002 | 32.5 | 3.10E-11 | 0.031 | 0.018 | 0.091 | |
| HDL cholesterol | | rs6123685 | A | G | 0.255 | 0.016 | 0.002 | 39.2 | 3.00E-13 | -0.004 | 0.021 | 0.838 | |
| HDL cholesterol | | rs61352607 | T | G | 0.241 | 0.031 | 0.002 | 138.5 | 4.40E-43 | 0.040 | 0.021 | 0.052 | |
| HDL cholesterol | | rs6142206 | A | G | 0.421 | -0.016 | 0.002 | 51.1 | 8.30E-17 | -0.014 | 0.018 | 0.418 | |
| HDL cholesterol | | rs61435086 | C | T | 0.012 | 0.089 | 0.009 | 78.4 | 3.70E-25 | -0.037 | 0.064 | 0.570 | |
| HDL cholesterol | | rs61596977 | T | C | 0.140 | -0.016 | 0.003 | 25.7 | 3.10E-09 | 0.001 | 0.024 | 0.975 | |
| HDL cholesterol | | rs61748951 | A | C | 0.024 | -0.036 | 0.006 | 24.8 | 1.70E-08 | 0.033 | 0.075 | 0.657 | |
| HDL cholesterol | | rs61805075 | A | G | 0.329 | -0.026 | 0.002 | 117.9 | 6.00E-37 | 0.009 | 0.019 | 0.641 | |
| HDL cholesterol | | rs61884005 | G | C | 0.121 | 0.016 | 0.003 | 23.0 | 2.40E-08 | 0.023 | 0.021 | 0.266 | |
| HDL cholesterol | | rs62102718 | T | A | 0.286 | -0.024 | 0.002 | 92.7 | 3.40E-29 | 0.007 | 0.019 | 0.736 | |
| HDL cholesterol | | rs62117487 | G | A | 0.057 | 0.046 | 0.004 | 91.0 | 1.10E-28 | -0.011 | 0.049 | 0.817 | |
| HDL cholesterol | | rs62246443 | C | T | 0.174 | -0.015 | 0.003 | 25.4 | 3.80E-09 | -0.031 | 0.021 | 0.135 | |
| HDL cholesterol | | rs62271373 | A | T | 0.060 | -0.041 | 0.004 | 75.3 | 2.50E-23 | 0.076 | 0.043 | 0.077 | |
| HDL cholesterol | | rs62331150 | T | G | 0.205 | -0.013 | 0.002 | 22.1 | 4.30E-08 | -0.022 | 0.021 | 0.293 | |
| HDL cholesterol | | rs62428831 | C | T | 0.142 | 0.018 | 0.003 | 31.2 | 1.00E-10 | 0.003 | 0.023 | 0.905 | |
| HDL cholesterol | | rs635769 | C | T | 0.628 | 0.020 | 0.002 | 73.3 | 1.40E-23 | -0.031 | 0.018 | 0.078 | |
| HDL cholesterol | | rs6460894 | C | T | 0.339 | -0.012 | 0.002 | 26.8 | 1.60E-09 | 0.013 | 0.020 | 0.505 | |
| HDL cholesterol | | rs6469605 | T | C | 0.569 | 0.032 | 0.002 | 198.2 | 5.80E-61 | -0.031 | 0.019 | 0.102 | |
| HDL cholesterol | | rs6693842 | C | T | 0.365 | 0.014 | 0.002 | 35.0 | 7.10E-12 | 0.018 | 0.018 | 0.303 | |
| HDL cholesterol | | rs6705285 | T | G | 0.609 | 0.012 | 0.002 | 25.5 | 1.90E-09 | -0.008 | 0.019 | 0.679 | |
| HDL cholesterol | | rs676210 | A | G | 0.205 | 0.059 | 0.002 | 463.4 | 4.50E-140 | -0.021 | 0.020 | 0.283 | |
| HDL cholesterol | | rs6762415 | G | T | 0.535 | -0.011 | 0.002 | 23.9 | 1.20E-08 | -0.009 | 0.018 | 0.604 | |
| HDL cholesterol | | rs6765484 | T | C | 0.473 | 0.023 | 0.002 | 102.2 | 3.90E-32 | -0.037 | 0.018 | 0.037 | |
| HDL cholesterol | | rs680321 | C | T | 0.457 | 0.011 | 0.002 | 23.6 | 1.50E-08 | 0.004 | 0.018 | 0.836 | |
| HDL cholesterol | | rs6824451 | A | G | 0.464 | -0.020 | 0.002 | 80.7 | 1.00E-25 | -0.006 | 0.018 | 0.716 | |
| HDL cholesterol | | rs686030 | A | C | 0.859 | 0.050 | 0.003 | 243.3 | 6.50E-74 | -0.012 | 0.027 | 0.653 | |
| HDL cholesterol | | rs689183 | T | G | 0.751 | -0.016 | 0.002 | 37.8 | 7.70E-13 | 0.018 | 0.019 | 0.346 | |
| HDL cholesterol | | rs6934962 | T | C | 0.402 | 0.016 | 0.002 | 50.9 | 7.30E-17 | -0.018 | 0.018 | 0.315 | |
| HDL cholesterol | | rs6939861 | A | G | 0.262 | -0.014 | 0.002 | 31.5 | 9.70E-11 | -0.026 | 0.020 | 0.191 | |
| HDL cholesterol | | rs7036107 | G | A | 0.511 | -0.012 | 0.002 | 30.3 | 2.90E-10 | 0.007 | 0.018 | 0.714 | |
| HDL cholesterol | | rs703966 | A | G | 0.419 | 0.016 | 0.002 | 48.4 | 4.90E-16 | -0.036 | 0.018 | 0.046 | |
| HDL cholesterol | | rs7158166 | C | T | 0.594 | 0.014 | 0.002 | 38.7 | 5.10E-13 | 0.020 | 0.018 | 0.261 | |
| HDL cholesterol | | rs71603401 | G | A | 0.137 | -0.015 | 0.003 | 22.8 | 3.20E-08 | 0.080 | 0.033 | 0.014 | |
| HDL cholesterol | | rs71647892 | C | T | 0.012 | -0.063 | 0.009 | 37.5 | 5.20E-13 | 0.023 | 0.141 | 0.872 | |
| HDL cholesterol | | rs7170463 | G | A | 0.311 | 0.019 | 0.002 | 62.0 | 4.20E-20 | 0.008 | 0.018 | 0.665 | |
| HDL cholesterol | | rs7186799 | C | A | 0.437 | -0.022 | 0.002 | 96.5 | 5.20E-31 | 0.058 | 0.018 | 0.001 | |
| HDL cholesterol | | rs7218647 | A | G | 0.559 | 0.011 | 0.002 | 23.8 | 1.40E-08 | 0.004 | 0.018 | 0.818 | |
| HDL cholesterol | | rs7251640 | C | T | 0.194 | 0.014 | 0.002 | 25.4 | 5.10E-09 | -0.010 | 0.021 | 0.653 | |
| HDL cholesterol | | rs72647336 | A | G | 0.057 | -0.044 | 0.004 | 83.8 | 2.60E-23 | -0.015 | 0.047 | 0.747 | |
| HDL cholesterol | | rs7281183 | A | G | 0.737 | -0.013 | 0.002 | 25.3 | 6.30E-09 | 0.012 | 0.019 | 0.549 | |
| HDL cholesterol | | rs72926946 | A | C | 0.296 | -0.021 | 0.002 | 71.9 | 3.10E-23 | -0.006 | 0.020 | 0.749 | |
| HDL cholesterol | | rs72964564 | C | A | 0.250 | -0.013 | 0.002 | 24.0 | 1.20E-08 | 0.010 | 0.023 | 0.664 | |
| HDL cholesterol | | rs7305678 | G | T | 0.866 | -0.017 | 0.003 | 25.9 | 4.20E-09 | 0.014 | 0.026 | 0.595 | |
| HDL cholesterol | | rs73151974 | T | C | 0.144 | -0.016 | 0.003 | 26.1 | 3.20E-09 | 0.009 | 0.026 | 0.733 | |
| HDL cholesterol | | rs73243877 | G | A | 0.168 | -0.025 | 0.003 | 72.7 | 2.50E-23 | 0.010 | 0.025 | 0.695 | |
| HDL cholesterol | | rs73455693 | A | G | 0.037 | 0.028 | 0.005 | 22.8 | 3.90E-08 | -0.022 | 0.040 | 0.589 | |
| HDL cholesterol | | rs74500135 | C | T | 0.010 | 0.067 | 0.010 | 35.4 | 2.30E-11 | -0.076 | 0.171 | 0.656 | |
| HDL cholesterol | | rs7488780 | C | G | 0.204 | 0.015 | 0.002 | 27.8 | 8.50E-10 | -0.008 | 0.020 | 0.693 | |
| HDL cholesterol | | rs75032664 | G | C | 0.013 | -0.055 | 0.009 | 31.3 | 1.30E-09 | -0.051 | 0.054 | 0.349 | |
| HDL cholesterol | | rs75152587 | T | G | 0.013 | -0.094 | 0.008 | 90.6 | 1.10E-28 | -0.080 | 0.082 | 0.327 | |
| HDL cholesterol | | rs75246752 | C | G | 0.013 | 0.049 | 0.008 | 24.8 | 5.40E-09 | 0.076 | 0.160 | 0.635 | |
| HDL cholesterol | | rs75479205 | G | A | 0.189 | 0.014 | 0.002 | 24.0 | 1.20E-08 | 0.043 | 0.021 | 0.037 | |
| HDL cholesterol | | rs75609851 | A | G | 0.010 | 0.170 | 0.010 | 241.9 | 1.40E-69 | 0.023 | 0.079 | 0.768 | |
| HDL cholesterol | | rs75662196 | C | G | 0.028 | 0.068 | 0.006 | 101.1 | 6.70E-31 | 0.013 | 0.118 | 0.910 | |
| HDL cholesterol | | rs7583067 | T | C | 0.240 | 0.015 | 0.002 | 31.4 | 6.60E-11 | -0.020 | 0.021 | 0.329 | |
| HDL cholesterol | | rs7622114 | A | C | 0.581 | 0.012 | 0.002 | 26.3 | 2.50E-09 | -0.031 | 0.018 | 0.082 | |
| HDL cholesterol | | rs76247316 | C | T | 0.480 | -0.011 | 0.002 | 26.1 | 2.40E-09 | -0.021 | 0.018 | 0.243 | |
| HDL cholesterol | | rs76428106 | C | T | 0.013 | -0.062 | 0.009 | 40.3 | 1.20E-12 | 0.072 | 0.083 | 0.384 | |
| HDL cholesterol | | rs76602912 | C | T | 0.024 | -0.043 | 0.006 | 35.4 | 4.00E-12 | -0.043 | 0.059 | 0.460 | |
| HDL cholesterol | | rs7665587 | C | T | 0.422 | 0.014 | 0.002 | 38.6 | 4.70E-13 | 0.011 | 0.018 | 0.546 | |
| HDL cholesterol | | rs76962725 | A | G | 0.037 | -0.028 | 0.005 | 22.4 | 4.60E-08 | 0.044 | 0.068 | 0.518 | |
| HDL cholesterol | | rs771481 | A | T | 0.184 | 0.029 | 0.002 | 100.7 | 9.10E-32 | 0.028 | 0.023 | 0.220 | |
| HDL cholesterol | | rs7725218 | A | G | 0.338 | -0.012 | 0.002 | 27.1 | 1.20E-09 | 0.044 | 0.018 | 0.016 | |
| HDL cholesterol | | rs77320712 | T | G | 0.235 | -0.013 | 0.002 | 23.6 | 1.90E-08 | 0.020 | 0.022 | 0.349 | |
| HDL cholesterol | | rs77605964 | A | G | 0.227 | 0.017 | 0.002 | 41.2 | 7.50E-14 | -0.019 | 0.023 | 0.411 | |
| HDL cholesterol | | rs77767539 | A | G | 0.014 | 0.045 | 0.008 | 23.3 | 3.60E-08 | 0.151 | 0.124 | 0.226 | |
| HDL cholesterol | | rs7794796 | T | C | 0.332 | -0.017 | 0.002 | 51.1 | 1.20E-16 | -0.001 | 0.018 | 0.971 | |
| HDL cholesterol | | rs77960347 | G | A | 0.013 | 0.291 | 0.008 | 902.8 | 1.00E-200 | -0.017 | 0.101 | 0.870 | |
| HDL cholesterol | | rs78058190 | A | G | 0.050 | -0.078 | 0.005 | 237.7 | 1.80E-57 | 0.032 | 0.032 | 0.318 | |
| HDL cholesterol | | rs7817574 | C | T | 0.185 | 0.033 | 0.002 | 134.3 | 3.50E-42 | -0.012 | 0.020 | 0.574 | |
| HDL cholesterol | | rs7826177 | C | T | 0.635 | 0.011 | 0.002 | 23.6 | 1.40E-08 | -0.005 | 0.018 | 0.796 | |
| HDL cholesterol | | rs7853377 | G | A | 0.216 | 0.015 | 0.002 | 31.8 | 4.70E-11 | 0.014 | 0.021 | 0.507 | |
| HDL cholesterol | | rs79153732 | T | C | 0.017 | -0.094 | 0.007 | 121.9 | 4.20E-38 | -0.073 | 0.111 | 0.511 | |
| HDL cholesterol | | rs7924036 | T | G | 0.503 | 0.014 | 0.002 | 38.8 | 3.60E-13 | -0.012 | 0.018 | 0.507 | |
| HDL cholesterol | | rs79600951 | G | C | 0.092 | -0.107 | 0.003 | 771.6 | 1.00E-200 | 0.013 | 0.030 | 0.663 | |
| HDL cholesterol | | rs79634051 | C | G | 0.028 | 0.039 | 0.006 | 34.0 | 9.00E-12 | -0.085 | 0.048 | 0.075 | |
| HDL cholesterol | | rs80005209 | G | T | 0.030 | -0.144 | 0.006 | 483.3 | 2.50E-144 | -0.054 | 0.064 | 0.401 | |
| HDL cholesterol | | rs8007841 | C | T | 0.657 | -0.013 | 0.002 | 30.2 | 1.80E-10 | -0.003 | 0.018 | 0.883 | |
| HDL cholesterol | | rs8014289 | G | A | 0.562 | 0.015 | 0.002 | 45.4 | 4.60E-15 | -0.034 | 0.019 | 0.069 | |
| HDL cholesterol | | rs8081548 | A | T | 0.659 | 0.018 | 0.002 | 61.9 | 6.90E-20 | 0.004 | 0.019 | 0.817 | |
| HDL cholesterol | | rs8086351 | G | C | 0.824 | 0.084 | 0.003 | 826.8 | 1.00E-200 | 0.003 | 0.023 | 0.883 | |
| HDL cholesterol | | rs830620 | T | C | 0.415 | 0.015 | 0.002 | 43.9 | 9.60E-15 | 0.042 | 0.018 | 0.018 | |
| HDL cholesterol | | rs880674 | C | T | 0.142 | 0.015 | 0.003 | 22.1 | 4.40E-08 | -0.008 | 0.025 | 0.764 | |
| HDL cholesterol | | rs907866 | A | G | 0.445 | -0.018 | 0.002 | 67.0 | 1.40E-21 | -0.011 | 0.018 | 0.535 | |
| HDL cholesterol | | rs921919 | A | G | 0.669 | -0.042 | 0.002 | 310.7 | 1.40E-90 | 0.001 | 0.018 | 0.956 | |
| HDL cholesterol | | rs9327468 | A | C | 0.755 | -0.015 | 0.002 | 33.1 | 1.90E-11 | -0.019 | 0.025 | 0.443 | |
| HDL cholesterol | | rs9347737 | G | A | 0.428 | -0.013 | 0.002 | 35.9 | 4.40E-12 | -0.030 | 0.018 | 0.091 | |
| HDL cholesterol | | rs9465693 | A | C | 0.305 | -0.012 | 0.002 | 25.1 | 6.20E-09 | -0.008 | 0.019 | 0.684 | |
| HDL cholesterol | | rs9604045 | T | G | 0.254 | 0.018 | 0.002 | 46.9 | 2.00E-14 | -0.046 | 0.024 | 0.051 | |
| HDL cholesterol | | rs9622830 | G | C | 0.354 | -0.016 | 0.002 | 49.6 | 2.60E-16 | -0.001 | 0.018 | 0.977 | |
| HDL cholesterol | | rs964184 | C | G | 0.866 | 0.105 | 0.003 | 1042.6 | 1.00E-200 | -0.048 | 0.025 | 0.055 | |
| HDL cholesterol | | rs9647335 | T | A | 0.192 | 0.028 | 0.002 | 96.6 | 2.40E-30 | -0.024 | 0.027 | 0.370 | |
| HDL cholesterol | | rs968050 | T | C | 0.482 | 0.014 | 0.002 | 37.6 | 8.10E-13 | 0.026 | 0.018 | 0.145 | |
| HDL cholesterol | | rs983663 | G | A | 0.252 | -0.014 | 0.002 | 31.4 | 7.70E-11 | 0.012 | 0.020 | 0.541 | |
| HDL cholesterol | | rs9904004 | G | A | 0.061 | -0.030 | 0.004 | 42.2 | 4.10E-14 | -0.006 | 0.033 | 0.867 | |
| HDL cholesterol | | rs9916613 | A | T | 0.356 | -0.013 | 0.002 | 32.8 | 2.30E-11 | -0.034 | 0.019 | 0.065 | |
| HDL cholesterol | | rs9933509 | C | T | 0.414 | -0.014 | 0.002 | 38.5 | 2.40E-13 | -0.013 | 0.018 | 0.450 | |
| HDL cholesterol | | rs998584 | A | C | 0.483 | -0.034 | 0.002 | 236.0 | 1.10E-71 | 0.023 | 0.018 | 0.188 | |
| HDL cholesterol | | rs9987289 | G | A | 0.909 | 0.087 | 0.003 | 511.2 | 9.10E-155 | -0.005 | 0.026 | 0.860 | |
| HDL cholesterol | | rs9989419 | G | A | 0.606 | 0.144 | 0.002 | 4026.8 | 1.00E-200 | -0.036 | 0.018 | 0.049 | |
| LDL cholesterol | | rs1010759 | A | G | 0.140 | -0.023 | 0.003 | 56.8 | 1.50E-14 | -0.024 | 0.030 | 0.424 | |
| LDL cholesterol | | rs1016988 | C | T | 0.191 | -0.017 | 0.003 | 41.4 | 4.80E-11 | -0.001 | 0.021 | 0.961 | |
| LDL cholesterol | | rs10231941 | C | T | 0.178 | 0.020 | 0.003 | 50.8 | 3.30E-13 | 0.012 | 0.022 | 0.582 | |
| LDL cholesterol | | rs10448340 | G | T | 0.320 | -0.015 | 0.002 | 43.5 | 1.70E-11 | 0.005 | 0.019 | 0.780 | |
| LDL cholesterol | | rs10832963 | G | T | 0.744 | 0.017 | 0.002 | 50.2 | 5.10E-13 | -0.008 | 0.018 | 0.666 | |
| LDL cholesterol | | rs10910476 | T | C | 0.555 | 0.012 | 0.002 | 33.4 | 3.70E-09 | 0.014 | 0.018 | 0.429 | |
| LDL cholesterol | | rs11014204 | T | C | 0.280 | 0.014 | 0.002 | 33.8 | 3.20E-09 | -0.014 | 0.021 | 0.523 | |
| LDL cholesterol | | rs11065385 | G | A | 0.691 | -0.024 | 0.002 | 112.9 | 2.00E-27 | -0.040 | 0.018 | 0.032 | |
| LDL cholesterol | | rs11099097 | T | C | 0.291 | -0.018 | 0.002 | 60.2 | 2.80E-15 | 0.000 | 0.019 | 0.987 | |
| LDL cholesterol | | rs11226108 | C | G | 0.192 | -0.016 | 0.003 | 35.0 | 1.60E-09 | -0.007 | 0.023 | 0.770 | |
| LDL cholesterol | | rs113177823 | A | G | 0.054 | -0.041 | 0.005 | 76.5 | 1.00E-18 | 0.012 | 0.037 | 0.746 | |
| LDL cholesterol | | rs114165349 | C | G | 0.023 | 0.056 | 0.007 | 63.1 | 6.20E-16 | -0.071 | 0.047 | 0.128 | |
| LDL cholesterol | | rs115458560 | C | T | 0.019 | -0.050 | 0.008 | 40.7 | 6.40E-11 | -0.065 | 0.167 | 0.700 | |
| LDL cholesterol | | rs11568318 | A | C | 0.066 | 0.026 | 0.004 | 36.0 | 7.80E-10 | -0.063 | 0.051 | 0.217 | |
| LDL cholesterol | | rs11591147 | T | G | 0.017 | -0.348 | 0.008 | 1843.8 | 1.00E-200 | 0.113 | 0.047 | 0.016 | |
| LDL cholesterol | | rs11601507 | A | C | 0.069 | 0.032 | 0.004 | 59.1 | 1.80E-15 | -0.025 | 0.033 | 0.445 | |
| LDL cholesterol | | rs11621792 | T | C | 0.453 | 0.019 | 0.002 | 80.1 | 1.10E-19 | -0.027 | 0.018 | 0.138 | |
| LDL cholesterol | | rs116734477 | T | C | 0.041 | -0.047 | 0.005 | 77.3 | 3.20E-19 | -0.015 | 0.051 | 0.772 | |
| LDL cholesterol | | rs117139027 | A | G | 0.018 | -0.057 | 0.008 | 49.7 | 5.90E-13 | -0.251 | 0.154 | 0.104 | |
| LDL cholesterol | | rs117733303 | G | A | 0.018 | 0.084 | 0.008 | 112.0 | 2.40E-27 | -0.057 | 0.084 | 0.498 | |
| LDL cholesterol | | rs118039278 | A | G | 0.079 | 0.084 | 0.004 | 445.8 | 1.80E-102 | -0.040 | 0.042 | 0.340 | |
| LDL cholesterol | | rs1183851 | C | T | 0.396 | 0.024 | 0.002 | 125.2 | 4.50E-30 | -0.006 | 0.018 | 0.722 | |
| LDL cholesterol | | rs12078100 | G | C | 0.623 | 0.013 | 0.002 | 35.6 | 1.00E-09 | 0.015 | 0.020 | 0.456 | |
| LDL cholesterol | | rs12162782 | G | T | 0.344 | 0.013 | 0.002 | 33.4 | 3.50E-09 | -0.001 | 0.018 | 0.969 | |
| LDL cholesterol | | rs12208357 | T | C | 0.070 | 0.057 | 0.004 | 186.6 | 5.40E-44 | 0.012 | 0.038 | 0.758 | |
| LDL cholesterol | | rs12246352 | G | A | 0.104 | 0.026 | 0.003 | 54.3 | 6.00E-14 | -0.006 | 0.037 | 0.871 | |
| LDL cholesterol | | rs1229984 | C | T | 0.973 | 0.053 | 0.006 | 64.5 | 1.00E-16 | -0.202 | 0.120 | 0.093 | |
| LDL cholesterol | | rs12445804 | A | G | 0.075 | 0.023 | 0.004 | 32.3 | 9.50E-09 | 0.065 | 0.028 | 0.021 | |
| LDL cholesterol | | rs12471768 | C | T | 0.704 | 0.014 | 0.002 | 34.0 | 2.50E-09 | -0.019 | 0.021 | 0.368 | |
| LDL cholesterol | | rs1250258 | T | C | 0.737 | 0.014 | 0.002 | 32.4 | 6.00E-09 | -0.035 | 0.022 | 0.109 | |
| LDL cholesterol | | rs1260326 | C | T | 0.604 | -0.035 | 0.002 | 254.2 | 5.10E-60 | -0.009 | 0.018 | 0.633 | |
| LDL cholesterol | | rs12916 | C | T | 0.401 | 0.062 | 0.002 | 817.8 | 1.70E-187 | 0.005 | 0.018 | 0.798 | |
| LDL cholesterol | | rs13020929 | A | G | 0.457 | 0.015 | 0.002 | 46.5 | 3.40E-12 | -0.017 | 0.018 | 0.336 | |
| LDL cholesterol | | rs13076933 | G | T | 0.259 | -0.021 | 0.002 | 74.6 | 1.60E-18 | -0.018 | 0.020 | 0.379 | |
| LDL cholesterol | | rs13107325 | T | C | 0.075 | -0.025 | 0.004 | 37.6 | 3.80E-10 | 0.080 | 0.075 | 0.290 | |
| LDL cholesterol | | rs13108218 | G | A | 0.615 | -0.018 | 0.002 | 66.0 | 1.80E-16 | -0.012 | 0.019 | 0.538 | |
| LDL cholesterol | | rs13121616 | G | A | 0.694 | 0.013 | 0.002 | 30.9 | 1.30E-08 | 0.001 | 0.020 | 0.977 | |
| LDL cholesterol | | rs143020224 | G | C | 0.119 | -0.169 | 0.003 | 2644.9 | 1.00E-200 | 0.028 | 0.029 | 0.331 | |
| LDL cholesterol | | rs145730801 | C | T | 0.044 | 0.036 | 0.005 | 48.4 | 3.30E-12 | 0.083 | 0.133 | 0.535 | |
| LDL cholesterol | | rs146433259 | T | C | 0.012 | -0.057 | 0.010 | 33.2 | 4.10E-08 | 0.056 | 0.129 | 0.665 | |
| LDL cholesterol | | rs146534110 | T | G | 0.013 | 0.068 | 0.009 | 53.5 | 7.80E-14 | -0.054 | 0.119 | 0.652 | |
| LDL cholesterol | | rs148150904 | TTAAAG | T | 0.190 | 0.016 | 0.003 | 33.9 | 2.50E-09 | 0.011 | 0.020 | 0.569 | |
| LDL cholesterol | | rs150474434 | A | G | 0.101 | -0.035 | 0.003 | 97.0 | 1.30E-23 | -0.035 | 0.033 | 0.281 | |
| LDL cholesterol | | rs1551891 | A | G | 0.088 | -0.174 | 0.004 | 2144.5 | 1.00E-200 | 0.010 | 0.034 | 0.766 | |
| LDL cholesterol | | rs1556562 | T | G | 0.790 | 0.019 | 0.002 | 52.9 | 2.10E-14 | -0.016 | 0.021 | 0.442 | |
| LDL cholesterol | | rs17050272 | A | G | 0.409 | -0.021 | 0.002 | 90.1 | 2.50E-22 | -0.002 | 0.018 | 0.894 | |
| LDL cholesterol | | rs174564 | G | A | 0.349 | -0.032 | 0.002 | 203.7 | 4.50E-48 | 0.000 | 0.018 | 0.987 | |
| LDL cholesterol | | rs17476364 | C | T | 0.108 | -0.022 | 0.003 | 39.9 | 1.20E-10 | 0.035 | 0.037 | 0.342 | |
| LDL cholesterol | | rs17569873 | T | C | 0.201 | 0.017 | 0.003 | 41.5 | 4.80E-11 | -0.024 | 0.026 | 0.345 | |
| LDL cholesterol | | rs1801689 | C | A | 0.031 | 0.062 | 0.006 | 99.9 | 1.80E-24 | 0.118 | 0.089 | 0.181 | |
| LDL cholesterol | | rs183130 | T | C | 0.324 | -0.033 | 0.002 | 209.6 | 2.10E-49 | -0.031 | 0.020 | 0.112 | |
| LDL cholesterol | | rs1883711 | C | G | 0.031 | 0.103 | 0.006 | 283.3 | 7.50E-64 | 0.031 | 0.036 | 0.390 | |
| LDL cholesterol | | rs200046586 | C | CA | 0.981 | 0.309 | 0.008 | 1568.0 | 1.00E-200 | -0.019 | 0.070 | 0.793 | |
| LDL cholesterol | | rs2043085 | C | T | 0.612 | -0.017 | 0.002 | 60.6 | 1.90E-15 | 0.002 | 0.018 | 0.893 | |
| LDL cholesterol | | rs2066714 | C | T | 0.129 | 0.021 | 0.003 | 43.7 | 1.40E-11 | -0.010 | 0.030 | 0.746 | |
| LDL cholesterol | | rs2068888 | A | G | 0.451 | -0.019 | 0.002 | 80.5 | 4.60E-20 | -0.007 | 0.018 | 0.697 | |
| LDL cholesterol | | rs2073547 | G | A | 0.184 | 0.036 | 0.003 | 167.3 | 2.30E-40 | 0.028 | 0.019 | 0.135 | |
| LDL cholesterol | | rs2160994 | C | T | 0.647 | 0.018 | 0.002 | 67.1 | 6.30E-17 | -0.053 | 0.019 | 0.005 | |
| LDL cholesterol | | rs2238162 | T | C | 0.523 | -0.017 | 0.002 | 59.9 | 2.80E-15 | -0.012 | 0.018 | 0.484 | |
| LDL cholesterol | | rs2250802 | A | G | 0.724 | -0.018 | 0.002 | 58.2 | 6.10E-15 | -0.017 | 0.019 | 0.364 | |
| LDL cholesterol | | rs2256814 | A | G | 0.198 | 0.015 | 0.003 | 32.6 | 6.10E-09 | 0.013 | 0.022 | 0.557 | |
| LDL cholesterol | | rs2287622 | G | A | 0.603 | -0.021 | 0.002 | 94.5 | 2.90E-23 | -0.027 | 0.018 | 0.117 | |
| LDL cholesterol | | rs2391825 | A | G | 0.279 | -0.013 | 0.002 | 29.9 | 2.60E-08 | -0.038 | 0.021 | 0.072 | |
| LDL cholesterol | | rs2519093 | T | C | 0.185 | 0.056 | 0.003 | 412.1 | 1.40E-95 | 0.102 | 0.022 | 0.000 | |
| LDL cholesterol | | rs2611867 | G | A | 0.514 | -0.027 | 0.002 | 155.6 | 4.70E-37 | 0.014 | 0.018 | 0.443 | |
| LDL cholesterol | | rs2618566 | T | G | 0.660 | -0.025 | 0.002 | 122.8 | 9.60E-30 | -0.008 | 0.019 | 0.664 | |
| LDL cholesterol | | rs2642438 | G | A | 0.703 | 0.025 | 0.002 | 118.1 | 8.00E-29 | -0.008 | 0.019 | 0.697 | |
| LDL cholesterol | | rs2737265 | G | A | 0.280 | -0.020 | 0.002 | 73.1 | 2.50E-18 | 0.028 | 0.019 | 0.142 | |
| LDL cholesterol | | rs2738447 | C | A | 0.593 | 0.042 | 0.002 | 380.5 | 2.90E-89 | -0.013 | 0.018 | 0.481 | |
| LDL cholesterol | | rs2740488 | C | A | 0.265 | -0.025 | 0.002 | 109.5 | 1.30E-26 | 0.014 | 0.022 | 0.528 | |
| LDL cholesterol | | rs2745353 | T | C | 0.518 | 0.013 | 0.002 | 35.5 | 1.10E-09 | 0.092 | 0.018 | 0.000 | |
| LDL cholesterol | | rs2820226 | A | G | 0.452 | -0.013 | 0.002 | 36.1 | 8.60E-10 | 0.039 | 0.018 | 0.029 | |
| LDL cholesterol | | rs28406917 | T | C | 0.428 | 0.012 | 0.002 | 30.6 | 1.80E-08 | -0.008 | 0.018 | 0.639 | |
| LDL cholesterol | | rs28601761 | G | C | 0.419 | -0.062 | 0.002 | 826.2 | 2.90E-185 | -0.018 | 0.018 | 0.324 | |
| LDL cholesterol | | rs28615248 | C | T | 0.196 | 0.018 | 0.003 | 47.2 | 2.70E-12 | 0.023 | 0.021 | 0.287 | |
| LDL cholesterol | | rs28631087 | C | T | 0.213 | -0.016 | 0.003 | 38.7 | 2.00E-10 | 0.018 | 0.024 | 0.462 | |
| LDL cholesterol | | rs28814720 | G | A | 0.520 | 0.012 | 0.002 | 32.6 | 1.50E-08 | 0.015 | 0.018 | 0.406 | |
| LDL cholesterol | | rs3104412 | G | A | 0.452 | -0.019 | 0.002 | 78.0 | 1.80E-19 | 0.013 | 0.019 | 0.499 | |
| LDL cholesterol | | rs3127580 | T | C | 0.155 | 0.036 | 0.003 | 147.3 | 2.20E-35 | -0.005 | 0.026 | 0.838 | |
| LDL cholesterol | | rs34042070 | G | C | 0.188 | 0.049 | 0.003 | 316.8 | 2.90E-73 | 0.023 | 0.022 | 0.301 | |
| LDL cholesterol | | rs34265667 | A | G | 0.034 | -0.032 | 0.006 | 29.7 | 2.60E-08 | -0.036 | 0.070 | 0.605 | |
| LDL cholesterol | | rs34568880 | T | C | 0.013 | 0.056 | 0.009 | 36.4 | 6.80E-10 | 0.072 | 0.103 | 0.483 | |
| LDL cholesterol | | rs35511051 | A | C | 0.210 | -0.022 | 0.003 | 69.4 | 1.10E-17 | 0.014 | 0.021 | 0.521 | |
| LDL cholesterol | | rs35882350 | G | A | 0.261 | 0.014 | 0.002 | 33.2 | 4.00E-09 | 0.027 | 0.020 | 0.169 | |
| LDL cholesterol | | rs35980001 | G | GC | 0.787 | -0.022 | 0.003 | 73.5 | 3.80E-18 | -0.018 | 0.021 | 0.375 | |
| LDL cholesterol | | rs3732359 | A | G | 0.780 | -0.017 | 0.003 | 45.5 | 5.70E-12 | -0.009 | 0.019 | 0.655 | |
| LDL cholesterol | | rs3780181 | G | A | 0.068 | -0.028 | 0.004 | 43.2 | 2.60E-11 | -0.037 | 0.038 | 0.342 | |
| LDL cholesterol | | rs3822855 | T | G | 0.402 | 0.018 | 0.002 | 67.4 | 4.50E-17 | -0.019 | 0.018 | 0.288 | |
| LDL cholesterol | | rs3823376 | T | C | 0.502 | 0.017 | 0.002 | 65.8 | 1.00E-16 | 0.006 | 0.018 | 0.758 | |
| LDL cholesterol | | rs4148826 | C | T | 0.180 | -0.016 | 0.003 | 32.5 | 6.70E-09 | 0.040 | 0.022 | 0.068 | |
| LDL cholesterol | | rs4263041 | G | A | 0.283 | -0.070 | 0.003 | 877.7 | 4.90E-171 | 0.013 | 0.019 | 0.514 | |
| LDL cholesterol | | rs4307732 | A | G | 0.106 | 0.045 | 0.003 | 167.7 | 9.80E-40 | 0.013 | 0.024 | 0.577 | |
| LDL cholesterol | | rs438568 | G | A | 0.609 | 0.012 | 0.002 | 32.7 | 5.50E-09 | -0.016 | 0.019 | 0.391 | |
| LDL cholesterol | | rs440677 | A | G | 0.623 | -0.016 | 0.002 | 52.5 | 1.50E-13 | -0.013 | 0.018 | 0.466 | |
| LDL cholesterol | | rs4666384 | G | A | 0.679 | -0.017 | 0.002 | 53.1 | 1.10E-13 | -0.001 | 0.020 | 0.966 | |
| LDL cholesterol | | rs472495 | T | G | 0.649 | 0.043 | 0.002 | 364.1 | 7.30E-85 | -0.019 | 0.018 | 0.275 | |
| LDL cholesterol | | rs4738684 | G | A | 0.665 | -0.032 | 0.002 | 195.5 | 3.00E-46 | 0.013 | 0.018 | 0.486 | |
| LDL cholesterol | | rs4930163 | A | G | 0.159 | 0.017 | 0.003 | 36.0 | 9.20E-10 | 0.036 | 0.025 | 0.149 | |
| LDL cholesterol | | rs4954192 | T | C | 0.373 | 0.015 | 0.002 | 44.3 | 6.70E-12 | -0.031 | 0.018 | 0.086 | |
| LDL cholesterol | | rs4970834 | T | C | 0.187 | -0.105 | 0.003 | 1488.2 | 1.00E-200 | 0.005 | 0.022 | 0.813 | |
| LDL cholesterol | | rs556107 | T | C | 0.523 | 0.035 | 0.002 | 272.1 | 1.20E-63 | 0.028 | 0.018 | 0.117 | |
| LDL cholesterol | | rs55637835 | T | C | 0.121 | -0.019 | 0.003 | 32.8 | 8.40E-09 | 0.066 | 0.030 | 0.025 | |
| LDL cholesterol | | rs55714927 | T | C | 0.190 | -0.026 | 0.003 | 94.9 | 2.40E-23 | -0.045 | 0.020 | 0.025 | |
| LDL cholesterol | | rs55831924 | T | C | 0.361 | 0.016 | 0.002 | 50.0 | 6.90E-13 | 0.037 | 0.019 | 0.053 | |
| LDL cholesterol | | rs56130071 | C | G | 0.217 | 0.033 | 0.003 | 164.9 | 4.70E-39 | 0.004 | 0.022 | 0.836 | |
| LDL cholesterol | | rs56236159 | G | T | 0.131 | 0.018 | 0.003 | 31.6 | 8.80E-09 | 0.020 | 0.019 | 0.305 | |
| LDL cholesterol | | rs5843957 | TG | T | 0.571 | 0.014 | 0.002 | 40.6 | 8.10E-11 | -0.011 | 0.019 | 0.539 | |
| LDL cholesterol | | rs597808 | G | A | 0.516 | 0.027 | 0.002 | 161.7 | 2.10E-38 | 0.031 | 0.018 | 0.077 | |
| LDL cholesterol | | rs6031587 | T | C | 0.072 | -0.026 | 0.004 | 38.7 | 5.70E-10 | 0.032 | 0.034 | 0.344 | |
| LDL cholesterol | | rs60612724 | G | A | 0.039 | 0.032 | 0.005 | 34.3 | 2.30E-09 | -0.019 | 0.050 | 0.706 | |
| LDL cholesterol | | rs6074012 | C | T | 0.524 | 0.012 | 0.002 | 29.8 | 2.40E-08 | -0.019 | 0.018 | 0.278 | |
| LDL cholesterol | | rs61003864 | C | T | 0.187 | 0.016 | 0.003 | 32.6 | 5.60E-09 | 0.000 | 0.021 | 0.995 | |
| LDL cholesterol | | rs61754230 | T | C | 0.020 | 0.043 | 0.007 | 31.6 | 9.10E-09 | -0.131 | 0.112 | 0.244 | |
| LDL cholesterol | | rs61988556 | C | T | 0.086 | -0.022 | 0.004 | 34.9 | 1.70E-09 | 0.026 | 0.033 | 0.430 | |
| LDL cholesterol | | rs62033400 | G | A | 0.395 | -0.014 | 0.002 | 43.8 | 1.40E-11 | -0.021 | 0.018 | 0.232 | |
| LDL cholesterol | | rs6475606 | T | C | 0.484 | -0.020 | 0.002 | 90.4 | 2.30E-22 | -0.080 | 0.018 | 0.000 | |
| LDL cholesterol | | rs6495122 | C | A | 0.591 | 0.014 | 0.002 | 44.1 | 1.20E-11 | 0.003 | 0.018 | 0.845 | |
| LDL cholesterol | | rs6544713 | C | T | 0.677 | -0.054 | 0.002 | 556.7 | 5.00E-129 | 0.006 | 0.021 | 0.787 | |
| LDL cholesterol | | rs6560499 | A | G | 0.576 | -0.012 | 0.002 | 32.0 | 8.90E-09 | 0.030 | 0.018 | 0.105 | |
| LDL cholesterol | | rs6602912 | G | T | 0.285 | 0.022 | 0.002 | 88.5 | 7.20E-22 | 0.017 | 0.020 | 0.401 | |
| LDL cholesterol | | rs6667939 | T | C | 0.719 | 0.015 | 0.002 | 41.7 | 5.00E-11 | -0.001 | 0.019 | 0.946 | |
| LDL cholesterol | | rs6680227 | A | G | 0.035 | -0.075 | 0.006 | 167.0 | 4.30E-40 | 0.014 | 0.040 | 0.732 | |
| LDL cholesterol | | rs6709904 | G | A | 0.113 | -0.043 | 0.003 | 166.2 | 1.10E-39 | 0.040 | 0.029 | 0.164 | |
| LDL cholesterol | | rs6732741 | A | T | 0.119 | -0.028 | 0.003 | 73.1 | 2.90E-18 | -0.004 | 0.027 | 0.898 | |
| LDL cholesterol | | rs6874202 | C | T | 0.634 | 0.032 | 0.002 | 213.7 | 1.60E-50 | 0.039 | 0.019 | 0.039 | |
| LDL cholesterol | | rs7108486 | C | T | 0.024 | -0.039 | 0.007 | 30.4 | 2.40E-08 | -0.007 | 0.060 | 0.903 | |
| LDL cholesterol | | rs71311871 | G | A | 0.083 | -0.028 | 0.004 | 53.8 | 7.50E-14 | 0.062 | 0.032 | 0.057 | |
| LDL cholesterol | | rs7202323 | G | T | 0.230 | -0.026 | 0.002 | 101.7 | 6.50E-25 | -0.017 | 0.022 | 0.452 | |
| LDL cholesterol | | rs7241918 | T | G | 0.824 | 0.016 | 0.003 | 31.7 | 1.00E-08 | 0.005 | 0.023 | 0.828 | |
| LDL cholesterol | | rs72631343 | G | C | 0.129 | -0.029 | 0.003 | 84.9 | 4.60E-21 | 0.025 | 0.025 | 0.327 | |
| LDL cholesterol | | rs72911393 | T | C | 0.148 | -0.018 | 0.003 | 37.0 | 5.30E-10 | -0.026 | 0.030 | 0.387 | |
| LDL cholesterol | | rs7562734 | C | G | 0.322 | -0.020 | 0.002 | 77.9 | 1.80E-19 | 0.017 | 0.018 | 0.344 | |
| LDL cholesterol | | rs7569317 | C | T | 0.531 | 0.018 | 0.002 | 70.2 | 8.90E-18 | 0.009 | 0.018 | 0.618 | |
| LDL cholesterol | | rs76468627 | T | C | 0.076 | -0.022 | 0.004 | 29.2 | 3.70E-08 | -0.007 | 0.031 | 0.821 | |
| LDL cholesterol | | rs7707394 | A | G | 0.357 | 0.040 | 0.002 | 331.3 | 2.40E-77 | -0.015 | 0.018 | 0.409 | |
| LDL cholesterol | | rs7734476 | A | G | 0.550 | 0.019 | 0.002 | 76.8 | 3.30E-19 | 0.027 | 0.018 | 0.122 | |
| LDL cholesterol | | rs7746081 | A | G | 0.304 | -0.023 | 0.002 | 102.8 | 4.00E-25 | 0.037 | 0.018 | 0.044 | |
| LDL cholesterol | | rs77542162 | G | A | 0.022 | 0.128 | 0.007 | 320.1 | 2.10E-74 | -0.128 | 0.105 | 0.223 | |
| LDL cholesterol | | rs7776054 | G | A | 0.261 | -0.016 | 0.002 | 44.8 | 8.20E-12 | -0.006 | 0.019 | 0.732 | |
| LDL cholesterol | | rs77960347 | G | A | 0.013 | 0.071 | 0.009 | 57.9 | 7.70E-15 | -0.017 | 0.101 | 0.870 | |
| LDL cholesterol | | rs78508096 | A | G | 0.226 | 0.018 | 0.002 | 48.2 | 1.30E-12 | -0.008 | 0.019 | 0.698 | |
| LDL cholesterol | | rs79220007 | C | T | 0.076 | -0.057 | 0.004 | 203.8 | 2.00E-48 | -0.080 | 0.046 | 0.086 | |
| LDL cholesterol | | rs79828839 | T | C | 0.199 | 0.014 | 0.003 | 29.5 | 2.90E-08 | 0.014 | 0.023 | 0.537 | |
| LDL cholesterol | | rs8107974 | T | A | 0.076 | -0.105 | 0.004 | 682.0 | 2.70E-158 | -0.034 | 0.036 | 0.346 | |
| LDL cholesterol | | rs869412 | C | T | 0.226 | -0.014 | 0.003 | 31.2 | 1.30E-08 | 0.008 | 0.020 | 0.684 | |
| LDL cholesterol | | rs880315 | C | T | 0.340 | -0.015 | 0.002 | 45.8 | 5.30E-12 | 0.025 | 0.018 | 0.157 | |
| LDL cholesterol | | rs9289196 | C | T | 0.174 | 0.017 | 0.003 | 38.2 | 3.00E-10 | 0.001 | 0.025 | 0.975 | |
| LDL cholesterol | | rs934197 | A | G | 0.335 | 0.083 | 0.002 | 1365.0 | 1.00E-200 | -0.004 | 0.020 | 0.842 | |
| LDL cholesterol | | rs9471968 | G | A | 0.546 | -0.012 | 0.002 | 29.5 | 2.90E-08 | 0.017 | 0.018 | 0.330 | |
| LDL cholesterol | | rs9496567 | A | G | 0.243 | -0.017 | 0.002 | 49.3 | 7.80E-13 | 0.010 | 0.022 | 0.637 | |
| LDL cholesterol | | rs960596 | T | C | 0.339 | 0.014 | 0.002 | 36.0 | 1.10E-09 | 0.032 | 0.018 | 0.081 | |
| LDL cholesterol | | rs964184 | C | G | 0.866 | -0.058 | 0.003 | 338.4 | 8.20E-79 | -0.048 | 0.025 | 0.055 | |
| LDL cholesterol | | rs9832727 | G | C | 0.340 | -0.015 | 0.002 | 43.1 | 2.10E-11 | -0.045 | 0.019 | 0.015 | |
| LDL cholesterol | | rs9834932 | G | A | 0.089 | -0.032 | 0.004 | 74.0 | 1.50E-18 | -0.004 | 0.032 | 0.890 | |
| LDL cholesterol | | rs9884390 | C | T | 0.234 | 0.025 | 0.002 | 99.9 | 5.90E-24 | -0.015 | 0.021 | 0.480 | |
| LDL cholesterol | | rs9894946 | G | A | 0.841 | -0.017 | 0.003 | 35.4 | 2.30E-09 | -0.025 | 0.024 | 0.299 | |
| LDL cholesterol | | rs9929977 | A | T | 0.370 | 0.017 | 0.002 | 57.5 | 1.00E-14 | -0.017 | 0.018 | 0.339 | |
| LDL cholesterol | | rs9987289 | G | A | 0.909 | 0.045 | 0.004 | 150.4 | 5.10E-36 | -0.005 | 0.026 | 0.860 | |
| Triglycerides | | rs1009360 | C | T | 0.419 | -0.018 | 0.002 | 73.3 | 3.40E-20 | -0.015 | 0.018 | 0.394 | |
| Triglycerides | | rs1009590 | C | G | 0.915 | 0.021 | 0.004 | 29.8 | 5.70E-09 | -0.027 | 0.033 | 0.415 | |
| Triglycerides | | rs10152471 | A | G | 0.389 | -0.014 | 0.002 | 38.3 | 4.00E-11 | 0.024 | 0.018 | 0.182 | |
| Triglycerides | | rs10210970 | T | C | 0.131 | 0.023 | 0.003 | 54.5 | 2.00E-15 | 0.062 | 0.023 | 0.006 | |
| Triglycerides | | rs10242866 | T | C | 0.399 | 0.016 | 0.002 | 52.7 | 7.00E-15 | 0.014 | 0.019 | 0.471 | |
| Triglycerides | | rs10277582 | T | C | 0.119 | -0.017 | 0.003 | 27.8 | 1.70E-08 | 0.042 | 0.028 | 0.141 | |
| Triglycerides | | rs1037117 | A | G | 0.255 | 0.017 | 0.002 | 49.7 | 5.70E-14 | 0.017 | 0.019 | 0.391 | |
| Triglycerides | | rs10405944 | C | T | 0.483 | -0.013 | 0.002 | 38.4 | 6.20E-11 | -0.032 | 0.018 | 0.074 | |
| Triglycerides | | rs1043897 | T | G | 0.416 | -0.015 | 0.002 | 46.1 | 3.40E-13 | -0.003 | 0.018 | 0.863 | |
| Triglycerides | | rs1044808 | C | G | 0.081 | -0.025 | 0.004 | 40.3 | 8.30E-12 | -0.033 | 0.038 | 0.384 | |
| Triglycerides | | rs1045241 | T | C | 0.271 | -0.021 | 0.002 | 74.7 | 3.20E-20 | 0.039 | 0.019 | 0.038 | |
| Triglycerides | | rs10513688 | A | G | 0.097 | 0.025 | 0.003 | 47.6 | 1.40E-13 | 0.024 | 0.030 | 0.415 | |
| Triglycerides | | rs10642257 | A | AAGGCC | 0.839 | 0.037 | 0.003 | 163.4 | 1.70E-42 | -0.038 | 0.021 | 0.079 | |
| Triglycerides | | rs10750766 | A | C | 0.710 | 0.019 | 0.002 | 68.3 | 7.80E-19 | 0.006 | 0.020 | 0.771 | |
| Triglycerides | | rs10773000 | T | G | 0.332 | -0.015 | 0.002 | 43.6 | 1.70E-12 | -0.044 | 0.018 | 0.016 | |
| Triglycerides | | rs10773049 | C | T | 0.395 | -0.029 | 0.002 | 177.9 | 4.10E-46 | -0.017 | 0.018 | 0.337 | |
| Triglycerides | | rs10775406 | G | A | 0.760 | 0.021 | 0.002 | 68.7 | 6.60E-19 | 0.049 | 0.020 | 0.014 | |
| Triglycerides | | rs1077835 | G | A | 0.220 | 0.047 | 0.002 | 340.4 | 2.20E-86 | 0.017 | 0.020 | 0.395 | |
| Triglycerides | | rs10797119 | C | T | 0.537 | 0.016 | 0.002 | 54.1 | 4.60E-15 | 0.004 | 0.018 | 0.810 | |
| Triglycerides | | rs10811662 | A | G | 0.173 | -0.015 | 0.003 | 30.0 | 4.60E-09 | 0.008 | 0.025 | 0.747 | |
| Triglycerides | | rs10883026 | T | C | 0.522 | -0.014 | 0.002 | 46.0 | 5.50E-13 | -0.005 | 0.018 | 0.786 | |
| Triglycerides | | rs10899490 | T | C | 0.161 | -0.017 | 0.003 | 34.5 | 2.70E-10 | -0.011 | 0.022 | 0.630 | |
| Triglycerides | | rs11000468 | T | C | 0.255 | -0.015 | 0.002 | 37.0 | 1.50E-10 | -0.003 | 0.018 | 0.862 | |
| Triglycerides | | rs11030107 | G | A | 0.261 | 0.016 | 0.002 | 43.8 | 1.20E-12 | -0.033 | 0.020 | 0.101 | |
| Triglycerides | | rs11078597 | C | T | 0.187 | 0.019 | 0.003 | 49.2 | 5.70E-14 | -0.034 | 0.022 | 0.130 | |
| Triglycerides | | rs11100083 | C | T | 0.226 | -0.016 | 0.002 | 39.7 | 1.50E-11 | -0.015 | 0.022 | 0.508 | |
| Triglycerides | | rs11118310 | T | A | 0.593 | 0.019 | 0.002 | 79.4 | 1.10E-21 | 0.047 | 0.018 | 0.008 | |
| Triglycerides | | rs11122450 | G | T | 0.612 | -0.048 | 0.002 | 486.7 | 1.30E-123 | -0.024 | 0.018 | 0.170 | |
| Triglycerides | | rs11185542 | C | G | 0.728 | -0.013 | 0.002 | 27.9 | 1.60E-08 | -0.002 | 0.019 | 0.904 | |
| Triglycerides | | rs11187019 | G | A | 0.551 | -0.012 | 0.002 | 29.6 | 6.40E-09 | -0.004 | 0.018 | 0.836 | |
| Triglycerides | | rs11206374 | A | G | 0.225 | 0.025 | 0.002 | 96.3 | 6.10E-26 | 0.002 | 0.022 | 0.942 | |
| Triglycerides | | rs112108602 | A | AG | 0.910 | 0.027 | 0.003 | 52.1 | 1.10E-14 | 0.027 | 0.038 | 0.484 | |
| Triglycerides | | rs112381903 | T | A | 0.067 | 0.023 | 0.004 | 29.7 | 7.50E-09 | -0.042 | 0.047 | 0.372 | |
| Triglycerides | | rs11240358 | A | G | 0.394 | 0.014 | 0.002 | 38.8 | 2.30E-11 | -0.047 | 0.018 | 0.009 | |
| Triglycerides | | rs1133400 | G | A | 0.220 | 0.014 | 0.002 | 28.6 | 9.80E-09 | -0.054 | 0.023 | 0.020 | |
| Triglycerides | | rs114165349 | C | G | 0.023 | 0.082 | 0.007 | 133.6 | 6.30E-35 | -0.071 | 0.047 | 0.128 | |
| Triglycerides | | rs11429307 | G | GT | 0.809 | -0.047 | 0.003 | 295.4 | 2.70E-75 | -0.064 | 0.026 | 0.012 | |
| Triglycerides | | rs11600815 | A | G | 0.052 | -0.032 | 0.005 | 45.0 | 2.10E-12 | -0.014 | 0.032 | 0.658 | |
| Triglycerides | | rs11637681 | G | A | 0.276 | 0.013 | 0.002 | 27.9 | 2.00E-08 | 0.021 | 0.022 | 0.337 | |
| Triglycerides | | rs11664106 | T | A | 0.374 | -0.013 | 0.002 | 32.7 | 2.40E-09 | -0.029 | 0.019 | 0.131 | |
| Triglycerides | | rs116843064 | A | G | 0.019 | -0.227 | 0.007 | 859.4 | 1.00E-200 | -0.056 | 0.055 | 0.303 | |
| Triglycerides | | rs117233107 | A | G | 0.015 | -0.073 | 0.009 | 70.7 | 1.80E-17 | -0.095 | 0.075 | 0.206 | |
| Triglycerides | | rs117287238 | A | G | 0.028 | -0.039 | 0.006 | 37.6 | 1.40E-10 | -0.067 | 0.057 | 0.243 | |
| Triglycerides | | rs117291242 | T | C | 0.037 | 0.030 | 0.005 | 27.5 | 1.70E-08 | -0.006 | 0.039 | 0.889 | |
| Triglycerides | | rs117316645 | A | G | 0.042 | 0.028 | 0.005 | 27.5 | 2.10E-08 | -0.069 | 0.046 | 0.134 | |
| Triglycerides | | rs117431393 | G | A | 0.035 | 0.031 | 0.005 | 29.4 | 6.50E-09 | 0.007 | 0.036 | 0.856 | |
| Triglycerides | | rs11746801 | A | G | 0.637 | -0.012 | 0.002 | 31.2 | 2.70E-09 | 0.006 | 0.018 | 0.734 | |
| Triglycerides | | rs11904650 | G | A | 0.021 | 0.041 | 0.007 | 30.6 | 3.00E-09 | -0.062 | 0.073 | 0.390 | |
| Triglycerides | | rs12185242 | C | A | 0.455 | 0.018 | 0.002 | 67.2 | 1.80E-18 | -0.012 | 0.018 | 0.509 | |
| Triglycerides | | rs12424054 | A | G | 0.232 | 0.019 | 0.002 | 57.5 | 4.50E-16 | -0.016 | 0.023 | 0.482 | |
| Triglycerides | | rs12440800 | T | A | 0.255 | 0.016 | 0.002 | 43.5 | 2.20E-12 | 0.000 | 0.022 | 0.991 | |
| Triglycerides | | rs12446515 | T | C | 0.323 | -0.033 | 0.002 | 215.5 | 3.50E-55 | -0.031 | 0.020 | 0.111 | |
| Triglycerides | | rs12475332 | G | T | 0.261 | -0.014 | 0.002 | 33.3 | 5.20E-10 | 0.014 | 0.021 | 0.527 | |
| Triglycerides | | rs12504746 | T | C | 0.193 | -0.015 | 0.003 | 32.0 | 1.40E-09 | -0.005 | 0.025 | 0.855 | |
| Triglycerides | | rs12530679 | G | A | 0.485 | -0.012 | 0.002 | 32.4 | 1.80E-09 | -0.023 | 0.018 | 0.198 | |
| Triglycerides | | rs12669911 | C | A | 0.614 | -0.012 | 0.002 | 29.4 | 7.90E-09 | -0.010 | 0.019 | 0.616 | |
| Triglycerides | | rs12880341 | C | T | 0.159 | 0.021 | 0.003 | 51.9 | 1.80E-14 | -0.035 | 0.026 | 0.171 | |
| Triglycerides | | rs12902047 | C | A | 0.314 | -0.013 | 0.002 | 31.7 | 1.70E-09 | 0.011 | 0.019 | 0.555 | |
| Triglycerides | | rs1292065 | G | C | 0.709 | -0.014 | 0.002 | 35.3 | 1.90E-10 | -0.047 | 0.019 | 0.013 | |
| Triglycerides | | rs12926107 | G | A | 0.455 | 0.013 | 0.002 | 34.9 | 2.70E-10 | -0.019 | 0.019 | 0.328 | |
| Triglycerides | | rs12928099 | A | C | 0.296 | -0.028 | 0.002 | 146.5 | 2.90E-38 | 0.004 | 0.020 | 0.853 | |
| Triglycerides | | rs12948505 | T | C | 0.194 | 0.014 | 0.003 | 26.6 | 3.20E-08 | -0.027 | 0.026 | 0.299 | |
| Triglycerides | | rs13066793 | G | A | 0.090 | -0.022 | 0.003 | 36.5 | 1.00E-10 | -0.052 | 0.032 | 0.104 | |
| Triglycerides | | rs13107325 | T | C | 0.075 | 0.030 | 0.004 | 55.3 | 1.70E-15 | 0.080 | 0.075 | 0.290 | |
| Triglycerides | | rs13108218 | G | A | 0.615 | -0.031 | 0.002 | 194.6 | 9.70E-50 | -0.012 | 0.019 | 0.538 | |
| Triglycerides | | rs13118477 | A | G | 0.393 | 0.015 | 0.002 | 47.3 | 1.90E-13 | -0.008 | 0.018 | 0.655 | |
| Triglycerides | | rs13264304 | G | C | 0.151 | 0.019 | 0.003 | 40.4 | 8.00E-12 | 0.000 | 0.021 | 0.996 | |
| Triglycerides | | rs13269725 | G | A | 0.078 | 0.035 | 0.004 | 78.0 | 2.00E-21 | -0.019 | 0.047 | 0.690 | |
| Triglycerides | | rs13354321 | C | T | 0.410 | -0.015 | 0.002 | 50.8 | 1.90E-14 | 0.012 | 0.018 | 0.482 | |
| Triglycerides | | rs13389219 | T | C | 0.393 | -0.038 | 0.002 | 297.9 | 6.60E-77 | -0.003 | 0.019 | 0.862 | |
| Triglycerides | | rs1340819 | C | A | 0.345 | -0.012 | 0.002 | 29.5 | 6.30E-09 | -0.008 | 0.019 | 0.667 | |
| Triglycerides | | rs134551 | T | C | 0.335 | -0.012 | 0.002 | 26.7 | 3.40E-08 | 0.035 | 0.020 | 0.079 | |
| Triglycerides | | rs1347188 | G | A | 0.246 | 0.014 | 0.002 | 31.6 | 1.90E-09 | -0.041 | 0.020 | 0.042 | |
| Triglycerides | | rs138191773 | A | G | 0.017 | -0.047 | 0.008 | 32.8 | 2.60E-09 | -0.288 | 0.141 | 0.042 | |
| Triglycerides | | rs139974673 | C | T | 0.026 | 0.143 | 0.006 | 459.9 | 2.20E-116 | 0.100 | 0.122 | 0.413 | |
| Triglycerides | | rs140107293 | G | A | 0.155 | -0.023 | 0.003 | 59.4 | 1.60E-16 | -0.021 | 0.025 | 0.409 | |
| Triglycerides | | rs140288 | A | G | 0.567 | -0.013 | 0.002 | 38.1 | 4.00E-11 | -0.016 | 0.018 | 0.371 | |
| Triglycerides | | rs1420384 | T | G | 0.667 | -0.013 | 0.002 | 32.1 | 1.20E-09 | 0.012 | 0.019 | 0.515 | |
| Triglycerides | | rs143076454 | A | G | 0.019 | 0.040 | 0.007 | 26.3 | 3.90E-08 | 0.053 | 0.075 | 0.482 | |
| Triglycerides | | rs145947882 | C | A | 0.026 | 0.137 | 0.006 | 420.9 | 3.40E-103 | 0.027 | 0.051 | 0.597 | |
| Triglycerides | | rs1473886 | T | G | 0.478 | -0.018 | 0.002 | 72.3 | 6.90E-20 | -0.012 | 0.018 | 0.512 | |
| Triglycerides | | rs148827772 | G | A | 0.023 | 0.047 | 0.007 | 42.1 | 5.70E-11 | 0.066 | 0.059 | 0.263 | |
| Triglycerides | | rs149142833 | T | C | 0.156 | 0.017 | 0.003 | 33.4 | 9.10E-10 | -0.036 | 0.024 | 0.135 | |
| Triglycerides | | rs149778057 | C | A | 0.328 | -0.016 | 0.002 | 48.3 | 1.50E-12 | -0.008 | 0.018 | 0.640 | |
| Triglycerides | | rs150419156 | A | G | 0.015 | -0.051 | 0.008 | 33.9 | 2.40E-09 | -0.050 | 0.117 | 0.672 | |
| Triglycerides | | rs150423652 | T | G | 0.007 | 0.286 | 0.012 | 478.3 | 1.40E-119 | 0.328 | 0.195 | 0.093 | |
| Triglycerides | | rs150460588 | C | T | 0.044 | 0.033 | 0.005 | 40.0 | 1.60E-11 | 0.032 | 0.050 | 0.530 | |
| Triglycerides | | rs150555490 | T | C | 0.057 | -0.038 | 0.004 | 69.9 | 2.50E-19 | -0.072 | 0.046 | 0.120 | |
| Triglycerides | | rs150564454 | A | G | 0.012 | -0.102 | 0.010 | 104.3 | 4.40E-26 | -0.019 | 0.190 | 0.920 | |
| Triglycerides | | rs151235402 | T | C | 0.016 | 0.052 | 0.008 | 37.4 | 1.50E-10 | -0.092 | 0.070 | 0.189 | |
| Triglycerides | | rs1544980 | C | T | 0.199 | 0.024 | 0.002 | 80.0 | 7.60E-22 | 0.005 | 0.023 | 0.834 | |
| Triglycerides | | rs1567353 | G | C | 0.308 | 0.015 | 0.002 | 41.3 | 7.40E-12 | 0.014 | 0.019 | 0.445 | |
| Triglycerides | | rs17184382 | C | A | 0.425 | -0.022 | 0.002 | 103.7 | 1.10E-27 | 0.018 | 0.019 | 0.326 | |
| Triglycerides | | rs17326656 | T | G | 0.238 | 0.017 | 0.002 | 48.7 | 7.80E-14 | -0.036 | 0.024 | 0.127 | |
| Triglycerides | | rs174566 | G | A | 0.350 | 0.049 | 0.002 | 472.4 | 2.80E-120 | 0.003 | 0.018 | 0.877 | |
| Triglycerides | | rs17585887 | C | T | 0.591 | -0.029 | 0.002 | 174.3 | 1.30E-45 | -0.036 | 0.018 | 0.040 | |
| Triglycerides | | rs1760801 | A | G | 0.296 | -0.020 | 0.002 | 75.7 | 1.50E-20 | -0.008 | 0.018 | 0.674 | |
| Triglycerides | | rs1799831 | T | C | 0.156 | 0.025 | 0.003 | 69.8 | 3.70E-19 | 0.026 | 0.022 | 0.245 | |
| Triglycerides | | rs1801689 | C | A | 0.031 | -0.066 | 0.006 | 114.2 | 2.40E-30 | 0.118 | 0.089 | 0.181 | |
| Triglycerides | | rs1835346 | G | A | 0.024 | -0.039 | 0.007 | 31.6 | 2.20E-09 | 0.015 | 0.071 | 0.836 | |
| Triglycerides | | rs186696265 | T | C | 0.015 | -0.104 | 0.008 | 139.0 | 2.40E-36 | 0.017 | 0.070 | 0.809 | |
| Triglycerides | | rs188247550 | T | C | 0.013 | -0.134 | 0.009 | 206.0 | 5.90E-49 | -0.089 | 0.040 | 0.028 | |
| Triglycerides | | rs193735 | A | G | 0.037 | 0.033 | 0.005 | 33.9 | 4.90E-10 | 0.031 | 0.035 | 0.378 | |
| Triglycerides | | rs1938566 | T | C | 0.835 | -0.021 | 0.003 | 54.8 | 2.00E-15 | 0.001 | 0.021 | 0.982 | |
| Triglycerides | | rs2043085 | C | T | 0.612 | -0.031 | 0.002 | 198.8 | 1.90E-51 | 0.002 | 0.018 | 0.893 | |
| Triglycerides | | rs2068888 | A | G | 0.451 | -0.032 | 0.002 | 221.2 | 3.20E-57 | -0.007 | 0.018 | 0.697 | |
| Triglycerides | | rs2070341 | T | C | 0.603 | 0.011 | 0.002 | 27.0 | 2.80E-08 | 0.007 | 0.018 | 0.718 | |
| Triglycerides | | rs2071887 | A | T | 0.345 | 0.016 | 0.002 | 53.0 | 7.30E-15 | 0.002 | 0.018 | 0.897 | |
| Triglycerides | | rs2081194 | C | G | 0.601 | -0.021 | 0.002 | 97.0 | 6.30E-25 | -0.031 | 0.019 | 0.103 | |
| Triglycerides | | rs2081687 | C | T | 0.663 | -0.026 | 0.002 | 134.6 | 7.60E-36 | 0.007 | 0.018 | 0.705 | |
| Triglycerides | | rs2092203 | T | C | 0.481 | 0.014 | 0.002 | 41.2 | 6.80E-12 | -0.018 | 0.018 | 0.309 | |
| Triglycerides | | rs2131311 | G | A | 0.715 | -0.012 | 0.002 | 27.7 | 2.50E-08 | -0.044 | 0.020 | 0.025 | |
| Triglycerides | | rs2131919 | G | A | 0.164 | 0.017 | 0.003 | 36.5 | 1.00E-10 | -0.003 | 0.023 | 0.900 | |
| Triglycerides | | rs213494 | T | C | 0.648 | 0.016 | 0.002 | 49.0 | 5.50E-14 | -0.050 | 0.020 | 0.010 | |
| Triglycerides | | rs2137557 | C | T | 0.646 | 0.012 | 0.002 | 27.9 | 1.60E-08 | -0.013 | 0.019 | 0.496 | |
| Triglycerides | | rs2187114 | A | G | 0.101 | -0.019 | 0.003 | 27.5 | 2.10E-08 | -0.005 | 0.027 | 0.848 | |
| Triglycerides | | rs2237029 | A | G | 0.601 | -0.014 | 0.002 | 41.1 | 8.30E-12 | 0.000 | 0.018 | 0.987 | |
| Triglycerides | | rs2240466 | A | G | 0.123 | -0.123 | 0.003 | 1437.9 | 1.00E-200 | -0.015 | 0.026 | 0.575 | |
| Triglycerides | | rs2240533 | C | T | 0.310 | -0.013 | 0.002 | 31.8 | 1.60E-09 | 0.014 | 0.019 | 0.484 | |
| Triglycerides | | rs2244278 | A | C | 0.121 | -0.027 | 0.003 | 68.1 | 1.20E-18 | 0.008 | 0.031 | 0.800 | |
| Triglycerides | | rs2267373 | T | C | 0.581 | 0.022 | 0.002 | 99.9 | 1.40E-26 | 0.033 | 0.018 | 0.065 | |
| Triglycerides | | rs2302263 | T | C | 0.089 | 0.044 | 0.003 | 135.9 | 8.00E-36 | -0.056 | 0.026 | 0.034 | |
| Triglycerides | | rs2304969 | T | G | 0.145 | -0.016 | 0.003 | 28.5 | 1.50E-08 | -0.013 | 0.020 | 0.499 | |
| Triglycerides | | rs2305746 | G | A | 0.933 | 0.028 | 0.004 | 43.8 | 1.20E-12 | 0.017 | 0.036 | 0.647 | |
| Triglycerides | | rs2382825 | T | C | 0.623 | -0.013 | 0.002 | 37.6 | 4.80E-11 | -0.021 | 0.020 | 0.284 | |
| Triglycerides | | rs2407278 | G | A | 0.030 | -0.034 | 0.006 | 29.7 | 5.40E-09 | -0.046 | 0.040 | 0.247 | |
| Triglycerides | | rs2487294 | T | G | 0.723 | 0.018 | 0.002 | 59.2 | 1.60E-16 | -0.018 | 0.019 | 0.350 | |
| Triglycerides | | rs2519093 | T | C | 0.185 | -0.021 | 0.003 | 59.4 | 1.50E-16 | 0.102 | 0.022 | 0.000 | |
| Triglycerides | | rs2604568 | A | T | 0.664 | 0.012 | 0.002 | 26.9 | 2.80E-08 | -0.020 | 0.020 | 0.319 | |
| Triglycerides | | rs2699805 | A | G | 0.400 | -0.020 | 0.002 | 85.8 | 5.10E-23 | 0.007 | 0.018 | 0.702 | |
| Triglycerides | | rs275184 | G | T | 0.162 | -0.017 | 0.003 | 36.1 | 2.90E-10 | 0.040 | 0.022 | 0.073 | |
| Triglycerides | | rs2773469 | G | A | 0.733 | -0.019 | 0.002 | 61.4 | 5.70E-17 | 0.007 | 0.020 | 0.725 | |
| Triglycerides | | rs278981 | C | T | 0.758 | 0.013 | 0.002 | 25.6 | 3.90E-08 | -0.012 | 0.021 | 0.575 | |
| Triglycerides | | rs2812208 | C | G | 0.021 | -0.048 | 0.007 | 42.3 | 3.50E-12 | -0.104 | 0.060 | 0.086 | |
| Triglycerides | | rs28383314 | C | T | 0.624 | 0.038 | 0.002 | 297.4 | 1.50E-76 | 0.046 | 0.021 | 0.029 | |
| Triglycerides | | rs28439112 | A | T | 0.256 | 0.013 | 0.002 | 26.6 | 2.70E-08 | 0.040 | 0.022 | 0.071 | |
| Triglycerides | | rs28577186 | A | G | 0.665 | -0.016 | 0.002 | 52.5 | 1.20E-14 | 0.021 | 0.018 | 0.248 | |
| Triglycerides | | rs28752924 | G | T | 0.243 | 0.024 | 0.003 | 96.6 | 1.10E-18 | 0.044 | 0.029 | 0.132 | |
| Triglycerides | | rs2925979 | C | T | 0.700 | -0.032 | 0.002 | 192.3 | 8.50E-50 | -0.013 | 0.019 | 0.490 | |
| Triglycerides | | rs2937124 | T | C | 0.363 | -0.018 | 0.002 | 68.0 | 8.10E-18 | -0.008 | 0.018 | 0.639 | |
| Triglycerides | | rs2943645 | T | C | 0.647 | 0.040 | 0.002 | 327.4 | 2.90E-84 | 0.034 | 0.018 | 0.060 | |
| Triglycerides | | rs2983896 | A | G | 0.215 | 0.014 | 0.002 | 28.3 | 1.20E-08 | 0.004 | 0.023 | 0.876 | |
| Triglycerides | | rs308 | G | T | 0.021 | -0.159 | 0.007 | 452.6 | 2.60E-115 | 0.080 | 0.051 | 0.118 | |
| Triglycerides | | rs3103310 | G | A | 0.242 | 0.020 | 0.002 | 66.3 | 1.10E-17 | -0.023 | 0.024 | 0.341 | |
| Triglycerides | | rs320369 | G | A | 0.683 | -0.013 | 0.002 | 30.0 | 5.20E-09 | 0.023 | 0.018 | 0.213 | |
| Triglycerides | | rs325485 | G | A | 0.603 | -0.012 | 0.002 | 29.1 | 8.60E-09 | -0.035 | 0.018 | 0.056 | |
| Triglycerides | | rs326222 | C | T | 0.698 | 0.025 | 0.002 | 118.3 | 1.40E-31 | -0.021 | 0.019 | 0.265 | |
| Triglycerides | | rs343 | A | C | 0.083 | -0.141 | 0.004 | 1350.0 | 1.00E-200 | 0.011 | 0.036 | 0.768 | |
| Triglycerides | | rs34389637 | C | CT | 0.935 | 0.025 | 0.004 | 34.3 | 3.80E-10 | -0.063 | 0.032 | 0.049 | |
| Triglycerides | | rs34672664 | T | TG | 0.930 | 0.022 | 0.004 | 26.9 | 3.10E-08 | 0.049 | 0.036 | 0.181 | |
| Triglycerides | | rs34682685 | A | G | 0.104 | 0.034 | 0.003 | 93.8 | 5.80E-25 | -0.030 | 0.038 | 0.421 | |
| Triglycerides | | rs35763453 | C | T | 0.059 | 0.028 | 0.004 | 39.4 | 6.20E-11 | -0.039 | 0.047 | 0.405 | |
| Triglycerides | | rs36043408 | A | G | 0.502 | -0.013 | 0.002 | 36.2 | 1.10E-10 | 0.035 | 0.018 | 0.045 | |
| Triglycerides | | rs3731696 | G | A | 0.121 | 0.022 | 0.003 | 45.0 | 5.10E-13 | 0.058 | 0.027 | 0.032 | |
| Triglycerides | | rs3758413 | C | T | 0.418 | 0.011 | 0.002 | 26.5 | 3.50E-08 | 0.001 | 0.018 | 0.959 | |
| Triglycerides | | rs3775228 | T | C | 0.400 | 0.034 | 0.002 | 242.2 | 5.80E-62 | 0.013 | 0.018 | 0.487 | |
| Triglycerides | | rs3808477 | T | C | 0.279 | -0.013 | 0.002 | 31.8 | 1.20E-09 | 0.028 | 0.019 | 0.139 | |
| Triglycerides | | rs3814883 | T | C | 0.482 | 0.015 | 0.002 | 48.9 | 8.70E-14 | 0.053 | 0.018 | 0.003 | |
| Triglycerides | | rs3820897 | C | T | 0.820 | 0.020 | 0.003 | 50.7 | 3.50E-14 | -0.017 | 0.019 | 0.361 | |
| Triglycerides | | rs3860846 | T | C | 0.275 | 0.030 | 0.002 | 155.3 | 4.00E-40 | 0.011 | 0.021 | 0.580 | |
| Triglycerides | | rs394872 | T | C | 0.536 | 0.011 | 0.002 | 27.4 | 2.40E-08 | -0.003 | 0.018 | 0.876 | |
| Triglycerides | | rs3974807 | T | C | 0.189 | 0.016 | 0.003 | 34.6 | 2.80E-10 | -0.005 | 0.026 | 0.843 | |
| Triglycerides | | rs4128205 | C | A | 0.509 | 0.012 | 0.002 | 29.2 | 8.10E-09 | 0.032 | 0.018 | 0.072 | |
| Triglycerides | | rs4134963 | T | C | 0.190 | -0.019 | 0.003 | 48.8 | 9.00E-14 | -0.008 | 0.022 | 0.724 | |
| Triglycerides | | rs41785 | A | C | 0.417 | -0.015 | 0.002 | 48.6 | 8.20E-14 | -0.014 | 0.018 | 0.436 | |
| Triglycerides | | rs4253750 | C | T | 0.214 | 0.018 | 0.002 | 46.9 | 3.20E-13 | 0.029 | 0.024 | 0.216 | |
| Triglycerides | | rs4471666 | G | T | 0.069 | -0.022 | 0.004 | 28.8 | 1.50E-08 | -0.003 | 0.032 | 0.937 | |
| Triglycerides | | rs4665972 | C | T | 0.605 | -0.100 | 0.002 | 2129.4 | 1.00E-200 | -0.006 | 0.018 | 0.753 | |
| Triglycerides | | rs4675812 | A | G | 0.588 | -0.014 | 0.002 | 43.3 | 1.50E-12 | 0.008 | 0.018 | 0.675 | |
| Triglycerides | | rs4731701 | T | C | 0.493 | -0.033 | 0.002 | 234.1 | 2.70E-60 | 0.021 | 0.018 | 0.228 | |
| Triglycerides | | rs4760254 | C | G | 0.239 | -0.028 | 0.002 | 127.1 | 1.00E-33 | 0.041 | 0.021 | 0.050 | |
| Triglycerides | | rs4761234 | C | T | 0.484 | -0.014 | 0.002 | 43.3 | 2.00E-12 | 0.013 | 0.018 | 0.463 | |
| Triglycerides | | rs4765148 | T | G | 0.313 | -0.025 | 0.002 | 120.0 | 9.70E-32 | -0.013 | 0.019 | 0.500 | |
| Triglycerides | | rs480823 | C | T | 0.079 | 0.156 | 0.004 | 1561.6 | 1.00E-200 | 0.009 | 0.024 | 0.714 | |
| Triglycerides | | rs483082 | T | G | 0.235 | 0.086 | 0.002 | 1182.9 | 1.00E-200 | -0.007 | 0.021 | 0.724 | |
| Triglycerides | | rs483808 | T | C | 0.702 | -0.014 | 0.002 | 34.5 | 4.00E-10 | -0.032 | 0.018 | 0.076 | |
| Triglycerides | | rs4841580 | C | T | 0.435 | -0.025 | 0.002 | 130.6 | 1.40E-34 | 0.021 | 0.022 | 0.346 | |
| Triglycerides | | rs4969179 | G | T | 0.604 | -0.018 | 0.002 | 66.5 | 2.80E-18 | 0.040 | 0.019 | 0.038 | |
| Triglycerides | | rs4976033 | G | A | 0.402 | 0.018 | 0.002 | 67.1 | 4.30E-18 | 0.022 | 0.018 | 0.224 | |
| Triglycerides | | rs499293 | A | G | 0.658 | -0.012 | 0.002 | 27.6 | 1.70E-08 | -0.023 | 0.018 | 0.209 | |
| Triglycerides | | rs55646464 | T | G | 0.300 | 0.012 | 0.002 | 27.5 | 1.90E-08 | -0.006 | 0.018 | 0.753 | |
| Triglycerides | | rs55966194 | G | C | 0.282 | -0.018 | 0.002 | 56.9 | 7.10E-16 | -0.004 | 0.021 | 0.854 | |
| Triglycerides | | rs56397607 | G | A | 0.183 | 0.017 | 0.003 | 40.2 | 1.10E-11 | -0.010 | 0.024 | 0.687 | |
| Triglycerides | | rs56902258 | A | T | 0.196 | -0.015 | 0.003 | 31.9 | 1.70E-09 | 0.003 | 0.022 | 0.893 | |
| Triglycerides | | rs57996145 | G | GT | 0.833 | -0.029 | 0.003 | 104.8 | 1.50E-27 | -0.020 | 0.026 | 0.434 | |
| Triglycerides | | rs581080 | C | G | 0.819 | 0.018 | 0.003 | 40.2 | 1.10E-11 | -0.017 | 0.025 | 0.509 | |
| Triglycerides | | rs58542926 | T | C | 0.075 | -0.103 | 0.004 | 649.3 | 5.30E-164 | -0.025 | 0.036 | 0.480 | |
| Triglycerides | | rs6028716 | A | G | 0.258 | -0.013 | 0.002 | 27.8 | 2.00E-08 | 0.020 | 0.023 | 0.390 | |
| Triglycerides | | rs6073958 | C | T | 0.199 | 0.056 | 0.002 | 436.0 | 1.60E-110 | -0.039 | 0.023 | 0.092 | |
| Triglycerides | | rs60856912 | T | G | 0.163 | 0.025 | 0.003 | 74.2 | 4.80E-20 | -0.020 | 0.021 | 0.343 | |
| Triglycerides | | rs61729990 | A | C | 0.018 | -0.056 | 0.008 | 48.3 | 1.10E-13 | 0.014 | 0.104 | 0.893 | |
| Triglycerides | | rs61830291 | C | A | 0.096 | 0.029 | 0.003 | 63.1 | 1.80E-17 | 0.031 | 0.028 | 0.268 | |
| Triglycerides | | rs61905078 | C | A | 0.074 | 0.200 | 0.004 | 2423.6 | 1.00E-200 | 0.003 | 0.029 | 0.918 | |
| Triglycerides | | rs61993685 | C | T | 0.076 | -0.023 | 0.004 | 34.1 | 3.90E-10 | 0.031 | 0.032 | 0.334 | |
| Triglycerides | | rs62102718 | T | A | 0.286 | 0.020 | 0.002 | 73.8 | 3.90E-20 | 0.007 | 0.019 | 0.736 | |
| Triglycerides | | rs62117489 | A | C | 0.056 | -0.043 | 0.004 | 85.9 | 3.60E-23 | -0.012 | 0.049 | 0.811 | |
| Triglycerides | | rs62128802 | T | C | 0.183 | -0.016 | 0.003 | 33.3 | 7.40E-10 | -0.015 | 0.023 | 0.515 | |
| Triglycerides | | rs62135012 | A | G | 0.358 | -0.012 | 0.002 | 28.0 | 1.50E-08 | -0.005 | 0.018 | 0.776 | |
| Triglycerides | | rs62271373 | A | T | 0.060 | 0.042 | 0.004 | 87.6 | 7.80E-23 | 0.076 | 0.043 | 0.077 | |
| Triglycerides | | rs62274099 | T | C | 0.424 | 0.012 | 0.002 | 31.5 | 2.30E-09 | -0.018 | 0.018 | 0.309 | |
| Triglycerides | | rs62397245 | G | C | 0.222 | 0.015 | 0.002 | 34.3 | 3.90E-10 | 0.031 | 0.020 | 0.130 | |
| Triglycerides | | rs62427982 | T | C | 0.322 | -0.013 | 0.002 | 34.2 | 4.20E-10 | 0.015 | 0.018 | 0.413 | |
| Triglycerides | | rs62473520 | C | T | 0.078 | -0.021 | 0.004 | 28.2 | 2.80E-08 | 0.026 | 0.035 | 0.457 | |
| Triglycerides | | rs6432622 | G | A | 0.490 | -0.011 | 0.002 | 26.1 | 3.80E-08 | -0.013 | 0.018 | 0.449 | |
| Triglycerides | | rs6506033 | T | C | 0.073 | -0.023 | 0.004 | 31.1 | 2.50E-09 | 0.024 | 0.029 | 0.409 | |
| Triglycerides | | rs6517522 | C | T | 0.498 | -0.013 | 0.002 | 36.7 | 9.40E-11 | 0.010 | 0.018 | 0.598 | |
| Triglycerides | | rs6532798 | T | C | 0.697 | 0.014 | 0.002 | 35.4 | 1.80E-10 | -0.017 | 0.019 | 0.372 | |
| Triglycerides | | rs6562773 | G | A | 0.548 | -0.012 | 0.002 | 31.8 | 2.00E-09 | -0.020 | 0.018 | 0.244 | |
| Triglycerides | | rs6572807 | G | A | 0.267 | 0.012 | 0.002 | 26.9 | 2.90E-08 | 0.015 | 0.020 | 0.462 | |
| Triglycerides | | rs676210 | A | G | 0.205 | -0.074 | 0.002 | 779.1 | 7.10E-198 | -0.021 | 0.020 | 0.283 | |
| Triglycerides | | rs6792725 | G | A | 0.692 | -0.015 | 0.002 | 43.7 | 6.20E-12 | 0.008 | 0.018 | 0.648 | |
| Triglycerides | | rs67981690 | G | A | 0.130 | 0.030 | 0.003 | 89.3 | 8.30E-24 | 0.047 | 0.023 | 0.038 | |
| Triglycerides | | rs6800707 | G | C | 0.811 | 0.030 | 0.003 | 121.0 | 5.30E-32 | 0.031 | 0.030 | 0.304 | |
| Triglycerides | | rs6805924 | T | G | 0.431 | 0.011 | 0.002 | 26.0 | 4.50E-08 | -0.017 | 0.018 | 0.333 | |
| Triglycerides | | rs684773 | C | A | 0.767 | 0.029 | 0.002 | 133.7 | 2.50E-35 | 0.012 | 0.025 | 0.625 | |
| Triglycerides | | rs6882076 | C | T | 0.634 | 0.033 | 0.002 | 224.3 | 4.90E-58 | 0.039 | 0.019 | 0.036 | |
| Triglycerides | | rs696825 | T | C | 0.253 | -0.020 | 0.002 | 68.6 | 7.50E-19 | 0.000 | 0.019 | 0.990 | |
| Triglycerides | | rs698927 | C | A | 0.184 | -0.018 | 0.003 | 44.3 | 9.20E-13 | 0.028 | 0.023 | 0.220 | |
| Triglycerides | | rs6999569 | G | A | 0.471 | -0.086 | 0.002 | 1634.7 | 1.00E-200 | -0.023 | 0.018 | 0.184 | |
| Triglycerides | | rs7000494 | C | G | 0.030 | 0.137 | 0.006 | 479.7 | 8.00E-121 | -0.054 | 0.064 | 0.401 | |
| Triglycerides | | rs7077812 | C | T | 0.195 | 0.014 | 0.003 | 28.0 | 1.40E-08 | -0.023 | 0.022 | 0.304 | |
| Triglycerides | | rs7134375 | A | C | 0.431 | -0.017 | 0.002 | 63.6 | 1.20E-17 | -0.011 | 0.018 | 0.535 | |
| Triglycerides | | rs7135509 | C | T | 0.293 | -0.012 | 0.002 | 26.9 | 4.10E-08 | -0.006 | 0.019 | 0.738 | |
| Triglycerides | | rs71368855 | T | C | 0.115 | 0.026 | 0.003 | 61.1 | 7.80E-17 | -0.010 | 0.035 | 0.779 | |
| Triglycerides | | rs7140110 | C | T | 0.298 | 0.028 | 0.002 | 148.1 | 1.20E-38 | 0.028 | 0.020 | 0.157 | |
| Triglycerides | | rs71538127 | G | C | 0.122 | 0.018 | 0.003 | 29.4 | 6.20E-09 | 0.065 | 0.030 | 0.029 | |
| Triglycerides | | rs71603401 | G | A | 0.137 | 0.026 | 0.003 | 73.2 | 1.10E-19 | 0.080 | 0.033 | 0.014 | |
| Triglycerides | | rs7215055 | G | A | 0.063 | 0.039 | 0.004 | 78.8 | 2.30E-21 | -0.010 | 0.033 | 0.752 | |
| Triglycerides | | rs7239575 | C | T | 0.490 | -0.016 | 0.002 | 57.0 | 6.80E-16 | -0.014 | 0.018 | 0.436 | |
| Triglycerides | | rs7244 | A | G | 0.174 | 0.015 | 0.003 | 29.3 | 6.40E-09 | 0.012 | 0.023 | 0.618 | |
| Triglycerides | | rs72555385 | G | A | 0.049 | 0.065 | 0.005 | 175.7 | 1.70E-45 | 0.094 | 0.059 | 0.113 | |
| Triglycerides | | rs72603744 | C | A | 0.316 | -0.013 | 0.002 | 29.9 | 4.60E-09 | 0.006 | 0.021 | 0.759 | |
| Triglycerides | | rs72644085 | C | T | 0.146 | -0.020 | 0.003 | 42.3 | 3.20E-12 | -0.021 | 0.026 | 0.422 | |
| Triglycerides | | rs7274718 | A | G | 0.599 | 0.016 | 0.002 | 53.9 | 3.80E-15 | -0.018 | 0.018 | 0.323 | |
| Triglycerides | | rs72784786 | A | G | 0.061 | 0.026 | 0.004 | 34.9 | 2.60E-10 | 0.048 | 0.035 | 0.171 | |
| Triglycerides | | rs72801474 | A | G | 0.092 | -0.031 | 0.003 | 69.8 | 3.60E-19 | -0.033 | 0.038 | 0.385 | |
| Triglycerides | | rs729761 | G | T | 0.712 | 0.018 | 0.002 | 57.2 | 9.50E-16 | 0.040 | 0.019 | 0.036 | |
| Triglycerides | | rs73025562 | A | G | 0.246 | 0.014 | 0.002 | 31.4 | 2.00E-09 | 0.019 | 0.019 | 0.316 | |
| Triglycerides | | rs7308584 | A | G | 0.184 | 0.015 | 0.003 | 29.9 | 5.30E-09 | 0.021 | 0.020 | 0.308 | |
| Triglycerides | | rs73238173 | G | C | 0.130 | -0.017 | 0.003 | 27.5 | 1.90E-08 | -0.001 | 0.025 | 0.962 | |
| Triglycerides | | rs7400002 | G | A | 0.231 | 0.014 | 0.002 | 30.5 | 3.60E-09 | 0.007 | 0.020 | 0.730 | |
| Triglycerides | | rs74090351 | A | G | 0.068 | -0.025 | 0.004 | 35.0 | 2.10E-10 | -0.022 | 0.033 | 0.502 | |
| Triglycerides | | rs742036 | A | G | 0.375 | -0.014 | 0.002 | 42.7 | 2.50E-12 | 0.029 | 0.019 | 0.125 | |
| Triglycerides | | rs7424120 | T | C | 0.602 | -0.012 | 0.002 | 32.3 | 1.20E-09 | -0.013 | 0.018 | 0.473 | |
| Triglycerides | | rs75268115 | G | A | 0.085 | -0.021 | 0.004 | 29.4 | 6.80E-09 | 0.045 | 0.030 | 0.136 | |
| Triglycerides | | rs75609851 | A | G | 0.010 | -0.199 | 0.010 | 362.9 | 1.60E-87 | 0.023 | 0.079 | 0.768 | |
| Triglycerides | | rs75634664 | G | C | 0.060 | 0.036 | 0.004 | 65.1 | 6.40E-17 | 0.000 | 0.032 | 0.995 | |
| Triglycerides | | rs75721796 | A | G | 0.214 | 0.021 | 0.002 | 65.8 | 1.20E-17 | 0.023 | 0.019 | 0.226 | |
| Triglycerides | | rs75942983 | T | A | 0.084 | -0.020 | 0.004 | 27.4 | 2.50E-08 | -0.068 | 0.041 | 0.100 | |
| Triglycerides | | rs77009508 | G | A | 0.074 | 0.045 | 0.004 | 122.5 | 1.80E-32 | 0.012 | 0.036 | 0.734 | |
| Triglycerides | | rs7704653 | G | A | 0.723 | 0.016 | 0.002 | 43.8 | 2.30E-12 | -0.011 | 0.021 | 0.594 | |
| Triglycerides | | rs7714361 | C | A | 0.234 | 0.014 | 0.002 | 30.5 | 4.40E-09 | -0.023 | 0.020 | 0.246 | |
| Triglycerides | | rs7735249 | G | C | 0.113 | 0.027 | 0.003 | 63.7 | 1.90E-17 | 0.034 | 0.027 | 0.205 | |
| Triglycerides | | rs7786339 | T | C | 0.169 | 0.016 | 0.003 | 33.0 | 7.60E-10 | 0.015 | 0.023 | 0.509 | |
| Triglycerides | | rs78058190 | A | G | 0.050 | 0.082 | 0.005 | 281.6 | 1.70E-57 | 0.032 | 0.032 | 0.318 | |
| Triglycerides | | rs7847285 | C | T | 0.589 | -0.012 | 0.002 | 28.8 | 1.00E-08 | -0.006 | 0.018 | 0.741 | |
| Triglycerides | | rs78484485 | A | G | 0.054 | -0.076 | 0.004 | 259.5 | 1.20E-66 | -0.003 | 0.030 | 0.915 | |
| Triglycerides | | rs78588343 | A | G | 0.176 | -0.016 | 0.003 | 30.9 | 2.50E-09 | 0.037 | 0.021 | 0.078 | |
| Triglycerides | | rs7861679 | T | C | 0.697 | 0.012 | 0.002 | 27.7 | 1.80E-08 | 0.029 | 0.020 | 0.134 | |
| Triglycerides | | rs79153732 | T | C | 0.017 | 0.077 | 0.008 | 90.2 | 2.00E-24 | -0.073 | 0.111 | 0.511 | |
| Triglycerides | | rs79287178 | A | G | 0.031 | 0.050 | 0.006 | 66.8 | 7.20E-17 | 0.046 | 0.045 | 0.304 | |
| Triglycerides | | rs79357714 | G | A | 0.046 | -0.029 | 0.005 | 32.2 | 1.20E-09 | 0.038 | 0.043 | 0.382 | |
| Triglycerides | | rs7947951 | G | A | 0.689 | 0.019 | 0.002 | 70.9 | 1.40E-19 | 0.003 | 0.018 | 0.865 | |
| Triglycerides | | rs80276949 | A | G | 0.023 | 0.046 | 0.007 | 40.9 | 8.00E-12 | 0.181 | 0.080 | 0.024 | |
| Triglycerides | | rs8102873 | T | C | 0.585 | 0.012 | 0.002 | 32.6 | 9.40E-10 | -0.037 | 0.018 | 0.038 | |
| Triglycerides | | rs8126001 | T | C | 0.490 | -0.016 | 0.002 | 59.1 | 2.40E-16 | -0.015 | 0.018 | 0.390 | |
| Triglycerides | | rs852388 | C | G | 0.211 | 0.016 | 0.002 | 36.3 | 1.60E-10 | 0.000 | 0.027 | 0.996 | |
| Triglycerides | | rs867939 | A | G | 0.576 | -0.014 | 0.002 | 39.9 | 1.80E-11 | 0.005 | 0.018 | 0.796 | |
| Triglycerides | | rs880315 | C | T | 0.340 | -0.012 | 0.002 | 27.4 | 2.20E-08 | 0.025 | 0.018 | 0.157 | |
| Triglycerides | | rs921971 | C | T | 0.266 | 0.016 | 0.002 | 42.2 | 4.00E-12 | -0.050 | 0.022 | 0.026 | |
| Triglycerides | | rs9373056 | T | C | 0.320 | -0.012 | 0.002 | 29.0 | 7.20E-09 | -0.023 | 0.020 | 0.250 | |
| Triglycerides | | rs9376511 | G | A | 0.203 | -0.015 | 0.002 | 34.3 | 3.20E-10 | -0.025 | 0.022 | 0.254 | |
| Triglycerides | | rs9425589 | A | G | 0.567 | -0.014 | 0.002 | 41.0 | 6.20E-12 | -0.006 | 0.018 | 0.720 | |
| Triglycerides | | rs9436661 | G | T | 0.353 | -0.078 | 0.002 | 1220.4 | 1.00E-200 | -0.010 | 0.020 | 0.606 | |
| Triglycerides | | rs9480889 | G | C | 0.783 | 0.016 | 0.002 | 39.8 | 1.30E-11 | 0.015 | 0.025 | 0.562 | |
| Triglycerides | | rs954244 | G | C | 0.255 | 0.015 | 0.002 | 39.3 | 1.60E-11 | -0.004 | 0.022 | 0.873 | |
| Triglycerides | | rs9561643 | C | A | 0.315 | 0.017 | 0.002 | 53.2 | 6.50E-15 | 0.004 | 0.018 | 0.830 | |
| Triglycerides | | rs9584870 | C | T | 0.366 | -0.012 | 0.002 | 31.3 | 4.30E-09 | 0.010 | 0.019 | 0.590 | |
| Triglycerides | | rs970069 | T | C | 0.212 | 0.016 | 0.002 | 38.8 | 2.60E-11 | 0.033 | 0.022 | 0.134 | |
| Triglycerides | | rs9831084 | C | T | 0.462 | -0.012 | 0.002 | 30.8 | 2.90E-09 | -0.013 | 0.018 | 0.464 | |
| Triglycerides | | rs9859117 | C | G | 0.203 | 0.015 | 0.002 | 31.2 | 2.40E-09 | 0.001 | 0.020 | 0.953 | |
| Triglycerides | | rs9889402 | A | G | 0.728 | 0.012 | 0.002 | 26.3 | 3.60E-08 | 0.018 | 0.021 | 0.382 | |
| Triglycerides | | rs9902027 | T | C | 0.774 | -0.015 | 0.002 | 35.4 | 2.10E-10 | 0.021 | 0.020 | 0.309 | |
| Triglycerides | | rs998584 | A | C | 0.483 | 0.040 | 0.002 | 354.8 | 2.20E-90 | 0.023 | 0.018 | 0.188 | |

| **Table S3 Mendelian randomization analysis on the causal effect of the gut microbiome on endometriosis.** | | | | | | | | |
| --- | --- | --- | --- | --- | --- | --- | --- | --- |
| **Exposure** | **Method** | **N snp** | **Beta** | **SE** | **P value** | **Egger intercept** | **P for pleiotropy** | **P for heterogeneity** |
| **Anaerotruncus** | Inverse variance weighted | 13 | 0.225 | 0.101 | 0.025 |  |  | 0.317 |
|  | MR Egger | 13 | -0.166 | 0.281 | 0.566 | 0.028 | 0.166 | 0.405 |
|  | Simple mode | 13 | 0.192 | 0.176 | 0.295 |  |  |  |
|  | Weighted median | 13 | 0.219 | 0.124 | 0.079 |  |  |  |
|  | Weighted mode | 13 | 0.183 | 0.150 | 0.248 |  |  |  |
|  | MR-PRESSO | 13 | 0.225 | 0.101 | 0.045 |  |  | 0.377 |
| **Eubacteriumruminantiumgroup** | Inverse variance weighted | 18 | -0.127 | 0.052 | 0.015 |  |  | 0.754 |
|  | MR Egger | 18 | -0.131 | 0.175 | 0.466 | 0.0004 | 0.983 | 0.692 |
|  | Simple mode | 18 | -0.038 | 0.132 | 0.779 |  |  |  |
|  | Weighted median | 18 | -0.075 | 0.072 | 0.297 |  |  |  |
|  | Weighted mode | 18 | -0.043 | 0.112 | 0.706 |  |  |  |
|  | MR-PRESSO | 18 | -0.127 | 0.045 | 0.012 |  |  | 0.770 |
| **Olsenella** | Inverse variance weighted | 10 | 0.104 | 0.050 | 0.036 |  |  | 0.598 |
|  | MR Egger | 10 | 0.028 | 0.159 | 0.863 | 0.011 | 0.629 | 0.524 |
|  | Simple mode | 10 | 0.116 | 0.110 | 0.320 |  |  |  |
|  | Weighted median | 10 | 0.100 | 0.063 | 0.112 |  |  |  |
|  | Weighted mode | 10 | 0.037 | 0.100 | 0.719 |  |  |  |
|  | MR-PRESSO | 10 | 0.104 | 0.045 | 0.046 |  |  | 0.596 |
| **Oscillospira** | Inverse variance weighted | 8 | 0.195 | 0.092 | 0.035 |  |  | 0.859 |
|  | MR Egger | 8 | -0.042 | 0.390 | 0.918 | 0.023 | 0.555 | 0.824 |
|  | Simple mode | 8 | 0.129 | 0.196 | 0.531 |  |  |  |
|  | Weighted median | 8 | 0.154 | 0.122 | 0.206 |  |  |  |
|  | Weighted mode | 8 | 0.114 | 0.188 | 0.563 |  |  |  |
|  | MR-PRESSO | 8 | 0.195 | 0.063 | 0.018 |  |  | 0.863 |

| **Table S4 Mendelian randomization analysis on the causal effect of endometriosis on gut microbiome.** | | | | | | | | |
| --- | --- | --- | --- | --- | --- | --- | --- | --- |
| **Outcome** | **Method** | **N snp** | **Beta** | **SE** | **P value** | **Egger intercept** | **P for pleiotropy** | **P for heterogeneity** |
| **Anaerotruncus** | Inverse variance weighted | 8 | -0.061 | 0.030 | 0.041 |  |  | 0.519 |
|  | MR Egger | 8 | -0.143 | 0.109 | 0.238 | 0.013 | 0.465 | 0.473 |
|  | Simple mode | 8 | -0.039 | 0.066 | 0.573 |  |  |  |
|  | Weighted median | 8 | -0.043 | 0.041 | 0.304 |  |  |  |
|  | Weighted mode | 8 | -0.035 | 0.068 | 0.623 |  |  |  |
|  | MR-PRESSO | 8 | -0.061 | 0.028 | 0.067 |  |  | 0.509 |
| **Eubacteriumruminantiumgroup** | Inverse variance weighted | 8 | -0.007 | 0.044 | 0.883 |  |  | 0.720 |
|  | MR Egger | 8 | 0.236 | 0.162 | 0.196 | -0.038 | 0.171 | 0.911 |
|  | Simple mode | 8 | 0.051 | 0.087 | 0.572 |  |  |  |
|  | Weighted median | 8 | 0.038 | 0.055 | 0.492 |  |  |  |
|  | Weighted mode | 8 | 0.053 | 0.074 | 0.499 |  |  |  |
|  | MR-PRESSO | 8 | -0.007 | 0.035 | 0.859 |  |  | 0.737 |
| **Olsenella** | Inverse variance weighted | 8 | 0.078 | 0.063 | 0.215 |  |  | 0.404 |
|  | MR Egger | 8 | -0.104 | 0.236 | 0.675 | 0.029 | 0.452 | 0.365 |
|  | Simple mode | 8 | -0.026 | 0.142 | 0.862 |  |  |  |
|  | Weighted median | 8 | 0.028 | 0.084 | 0.742 |  |  |  |
|  | Weighted mode | 8 | -0.023 | 0.124 | 0.858 |  |  |  |
|  | MR-PRESSO | 8 | 0.078 | 0.063 | 0.255 |  |  | 0.434 |
| **Oscillospira** | Inverse variance weighted | 8 | 0.021 | 0.038 | 0.589 |  |  | 0.436 |
|  | MR Egger | 8 | -0.021 | 0.149 | 0.890 | 0.007 | 0.779 | 0.337 |
|  | Simple mode | 8 | 0.005 | 0.078 | 0.953 |  |  |  |
|  | Weighted median | 8 | 0.001 | 0.049 | 0.990 |  |  |  |
|  | Weighted mode | 8 | 0.0002 | 0.067 | 0.998 |  |  |  |
|  | MR-PRESSO | 8 | 0.021 | 0.038 | 0.604 |  |  | 0.477 |
